# Regiodivergent and stereoselective hydroxyazidation of alkenes by biocatalytic cascades

**DOI:** 10.1016/j.isci.2021.102883

**Published:** 2021-07-17

**Authors:** Jing-Fei Wu, Nan-Wei Wan, Ying-Na Li, Qing-Ping Wang, Bao-Dong Cui, Wen-Yong Han, Yong-Zheng Chen

**Affiliations:** 1 Key Laboratory of Biocatalysis & Chiral Drug Synthesis of Guizhou Province, Generic Drug Research Center of Guizhou Province, Green Pharmaceuticals Engineering Research Center of Guizhou Province, School of Pharmacy, Zunyi Medical University, Zunyi, 563000, China; 2 Key Laboratory of Basic Pharmacology of Ministry of Education and Joint International Research Laboratory of Ethnomedicine of Ministry of Education, Zunyi Medical University, , Zunyi, 563000, China

**Keywords:** Biosynthesis, Biocatalysis, Bioengineering

## Abstract

Asymmetric functionalization of alkenes allows the direct synthesis of a wide range of chiral compounds. Vicinal hydroxyazidation of alkenes provides a desirable path to 1,2-azidoalcohols; however, existing methods are limited by the control of stereoselectivity and regioselectivity. Herein, we describe a dual-enzyme cascade strategy for regiodivergent and stereoselective hydroxyazidation of alkenes, affording various enantiomerically pure 1,2-azidoalcohols. The biocatalytic cascade process is designed by combining styrene monooxygenase-catalyzed asymmetric epoxidation of alkenes and halohydrin dehalogenase-catalyzed regioselective ring opening of epoxides with azide. Additionally, a one-pot chemo-enzymatic route to chiral *β*-hydroxytriazoles from alkenes is developed via combining the biocatalytic cascades and Cu-catalyzed azide-alkyne cycloaddition.

## Highlights

• A dual-enzyme cascade is developed for asymmetric hydroxyazidation of alkenes
• Regiodivergent and stereoselective hydroxyazidation of alkenes is achieved
• Various enantiomerically pure 1,2-azidoalcohols are synthesized from alkenes
• Chiral *β*-hydroxytriazoles are prepared from alkenes by a chemo-enzymatic approach

## Graphical abstract

**Figure:**
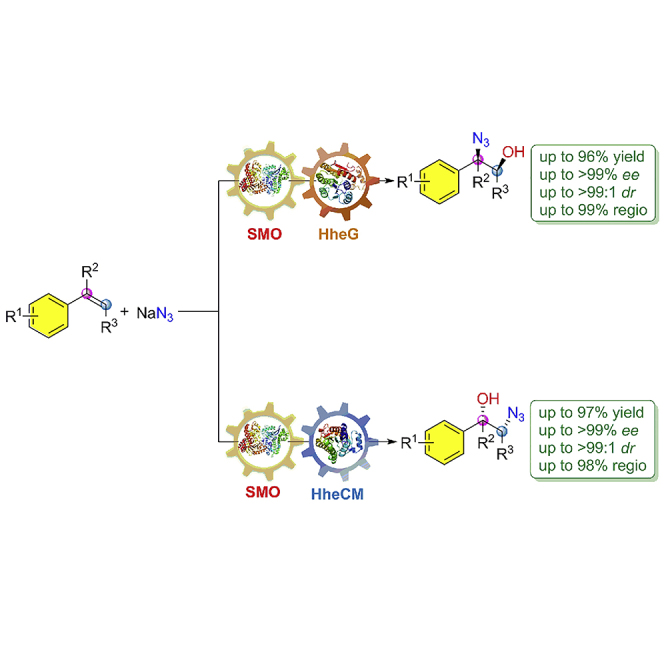

## Introduction

The 1,2-azidoalcohols are useful building blocks for the synthesis of various pharmaceuticals, biologically active molecules, natural products and synthetic materials (Bräse et al., 2005; Chiba et al., 2009; Meldal and Tornøe, 2008; Sletten and Bertozzi, 2011). Traditional synthetic methods to 1,2-azidoalcohols include the ring opening of corresponding epoxides (Larrow et al., 1996), substitution of vicinal halohydrins (Ohkuma et al., 2007), and reduction of *α*-azido carbonyl compounds (Patonay et al., 2011). However, these methods are restricted by some drawbacks such as the use of prefunctionalized starting materials. Direct difunctionalization of alkenes has emerged as a powerful strategy in organic synthesis, which has been successfully applied in the conversion of olefins into more structurally diverse 1,2-difunctionalized compounds (Fu et al., 2017; Ge et al., 2020; Koike and Akita, 2018; McDonald et al., 2011; Sauer and Lin, 2018; Yin et al., 2016). Vicinal hydroxyazidation of alkenes offers a simpler and more convenient approach for preparing 1,2-azidoalcohols. So far, several effective approaches have been developed for the synthesis of 1,2-azidoalcohols through direct hydroxyazidation of alkenes (Prasad et al., 2015; Sakurada et al., 2000). However, stoichiometric or excess oxidants must be used, and these methods are restricted by the specified O-sources. Therefore, it is urgent to develop greener and more efficient strategies for vicinal hydroxyazidation of alkenes.

**Figure 1:**
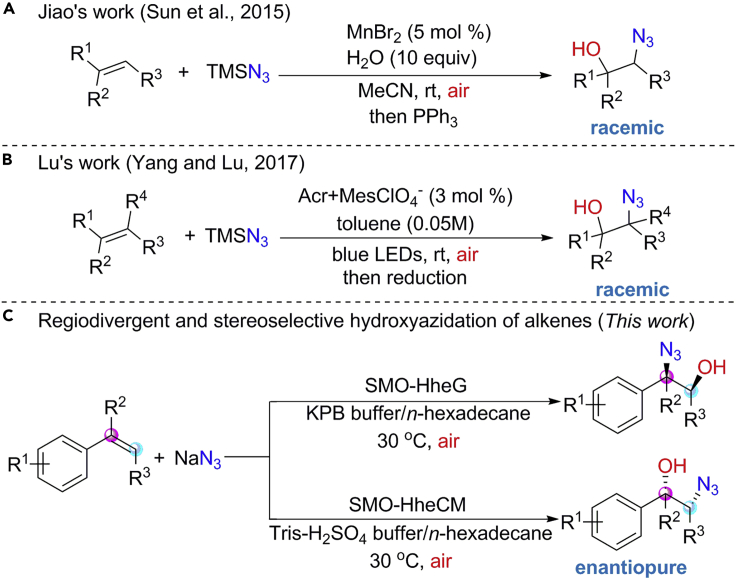
Direct synthesis of 1,2-azidoalcohols through vicinal hydroxyazidation of alkenes

Molecular oxygen (O_2_) is regarded as an ideal oxidant in terms of green and sustainable chemistry due to its inexpensive and environmentally benign nature (Shi et al., 2012). Thus, the replacement of chemical oxidants with O_2_ or the more advantageous air for the hydroxyazidation of alkenes is a highly desired task. Recently, Jiao and coworkers have developed a convenient Mn-catalyzed aerobic oxidative hydroxyazidation of olefins for the synthesis of 1,2-azidoalcohols using air as oxidant and TMSN_3_ as N_3_ source (Figure 1A) (Sun et al., 2015). In addition, Lu and Yang in 2017 also reported a facile visible-light-promoted aerobic hydroxyazidation of alkenes to afford 1,2-azidoalcohols using air and TMSN_3_ as the terminal oxidant and N_3_ source, respectively (Figure 1B) (Yang and Lu, 2017). These elegant strategies feature mild conditions and broad substrate scope, providing efficient approaches to 1,2-azidoalcohols from alkenes. However, there are several important issues that need to be addressed: (i) control of regioselectivity for regiodivergent synthesis of 1,2-azidoalcohols is difficult; (ii) enantioselective hydroxyazidation of alkenes to afford enantiopure 1,2-azidoalcohols still remains challenging.

**Figure 2:**
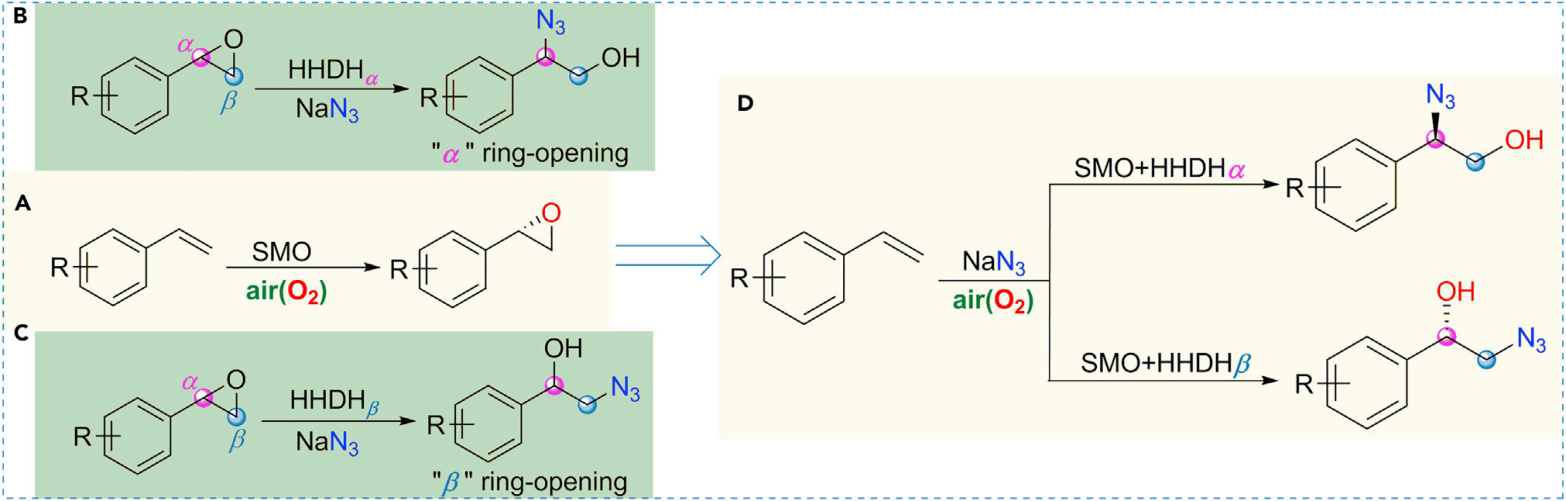
Design of biocatalytic cascades for regioselective and stereoselective hydroxyazidation of alkenes HHDH_*α*_: ring opening of styrene oxides with azide at C_*α*_-position; HHDH_*β*_: ring opening of styrene oxides with azide at C_*β*_-position.

Biocatalysis is an environmentally attractive and sustainable synthetic technology, which has been integrated into mainstream organic synthesis, particularly for the synthesis of chiral molecules (Devine et al., 2018; Reetz, 2013; Sheldon and Pereira, 2017; Sheldon and Woodley, 2018; Sun et al., 2016). Enzymes are likely to be compatible with each other and thus can be applied on “one-pot” sequential organic transformations without isolating intermediates (France et al., 2017; Ricca et al., 2011; Schrittwieser et al., 2018). Over the past years, many non-natural biocatalytic cascades have been developed by combining multiple enzymatic transformations, synthesizing diverse valuable compounds from simple precursors (Both et al., 2016; Chen et al., 2018; Corrado et al., 2019; Mutti et al., 2015; Wu et al., 2014, 2016, 2017; Zhang et al., 2015; Zhou et al., 2016). Styrene monooxygenases (SMOs) are valuable enzymes, which have been used to catalyze the asymmetric epoxidation of styrenes with air as an oxidant to afford styrene oxides in high optical purity (Figure 2A) (Corrado et al., 2018; Heine et al., 2017, 2018; Panke et al., 2000). By combining SMO with two regioselective epoxide hydrolases, Li and coworkers have developed a cascade biocatalysis for dihydroxylation of olefins, affording stereocomplementary chiral diols in high chemical and optical purity (Wu et al., 2014). Halohydrin dehalogenase (HHDH) is another synthetically attractive enzyme with catalytic promiscuity, which performs in the dehalogenation of vicinal halohydrins with the production of epoxides (Haak et al., 2008; Schallmey and Schallmey, 2016; van Hylckama Vlieg et al., 2001) and the formation of *β*-substituted alcohols via ring opening of epoxides in the presence of several anionic nucleophiles such as azide (Calderini et al., 2019; de Jong et al., 2005; Hasnaoui-Dijoux et al., 2008; Koopmeiners et al., 2017). HHDHs also have been used to construct biocatalytic cascades for the synthesis of enantiopure 1,2-azidoalcohols and 1,2-hydroxynitriles from *α*-chloroketones (Schrittwieser et al., 2009). For a long time, HHDHs have been considered to catalyze ring opening of styrene oxides with azide in favor of *β*-regioselectivity (Figure 2B) (Lutje Spelberg et al., 2001; Molinaro et al., 2010), while we recently identified the HheG, a HHDH from *Ilumatobacter coccineus* with high *α*-regioselectivity (Figure 2C) (An et al., 2019). In this context, we herein develop a practical one-pot biocatalytic cascade strategy for regiodivergent and stereoselective hydroxyazidation of alkenes by combining SMO and two regiocomplementary HHDHs (Figure 2D), affording various enantiopure 1,2-azidoalcohols (Figure 1C).

## Results and discussion

**Figure 3:**
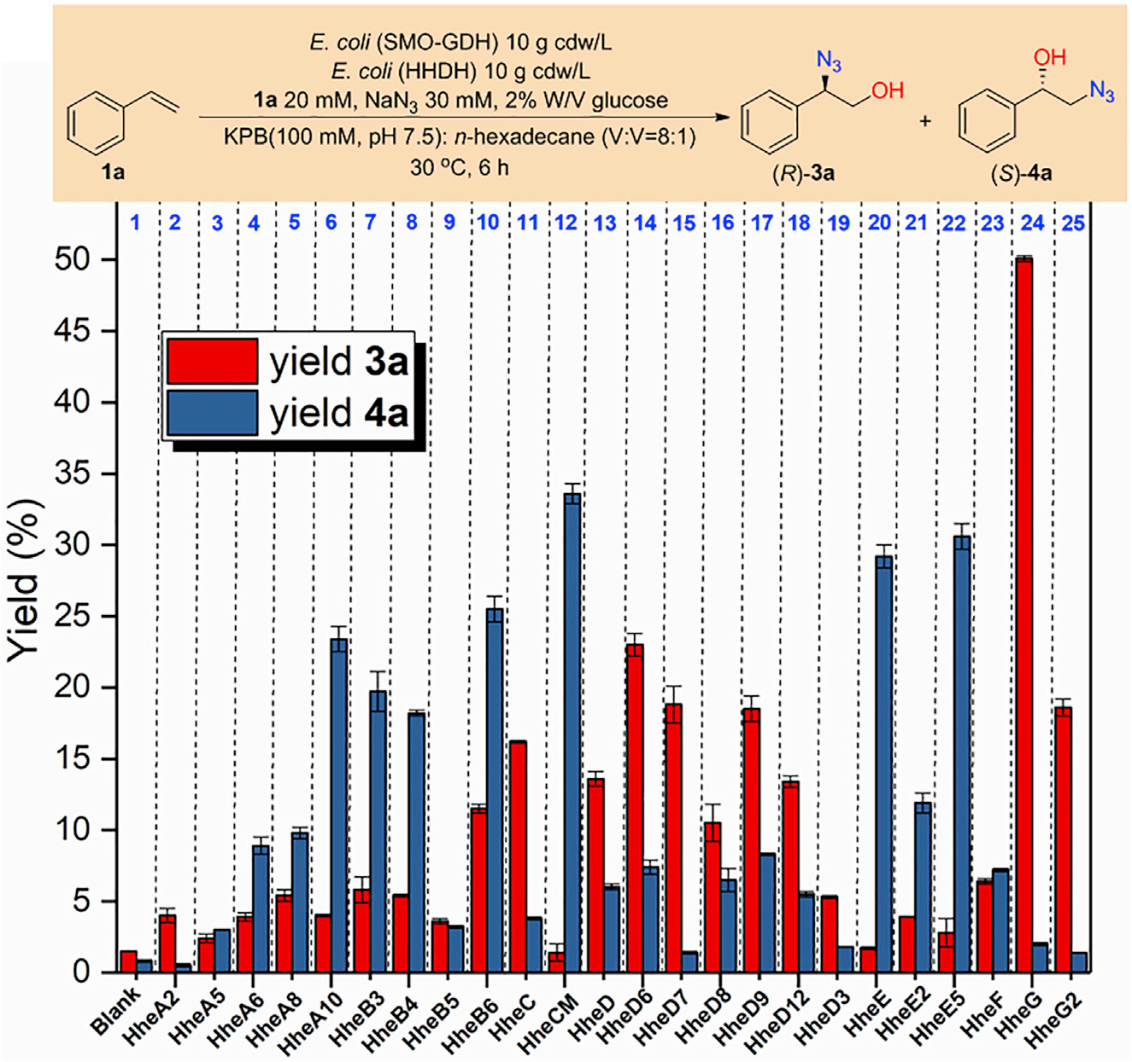
Screening of biocatalytic cascades for stereoselective and regioselective hydroxyazidation of **1a** Reactions were carried out in a biphasic system containing 4 mL of K_2_HPO_4_-KH_2_PO_4_ buffer (KPB, 100 mM, pH 7.5), 0.5 mL *n*-hexadecane, 20 mM **1a**, 30 mM NaN_3_, 2% W/V glucose, and resting cells *E. coli* (SMO-GDH) (10 g cdw/L) and *E. coli* (HHDH) (10 g cdw/L) at 30°C, 250 rpm for 6 hr. Blank: the reaction was carried out in the absence of *E. coli* (HHDH). Yield is the analytical yield of the formation of 1,2-azidoalcohol product, determined by chiral HPLC analysis.

We initially constructed a recombinant *Escherichia coli* (SMO-GDH) strain for co-expression of a styrene monooxygenase and a glucose dehydrogenase. Whole-cell bioconversion of 20 mM styrene (**1a**) with *E. coli* (SMO-GDH) in a biphasic system showed the specific activity of 12.7 U (g cdw)^−1^ and produced (*S*)-styrene oxide (**2a**) with 91% yield and 99% (enantiomeric excess (*ee*) after 4 hr (Figure S1). We then screened biocatalytic cascades with the model reaction of transformation of **1a** into 2-azido-2-phenylethan-1-ol (**3a**) and 2-azido-1-phenylethan-1-ol (**4a**) (Figure 3). By combining with SMO, more than twenty HHDHs were evaluated for asymmetric hydroxyazidation of **1a** in one-pot cascade process, and the results are summarized in Figure 3 (see Table S1 for details). As the SMO-catalyzed asymmetric epoxidation step is highly *S*-enantioselective, both (*R*)-**3a** and (*S*)-**4a** are produced in high *ee* in these cascades. The control reaction (Figure 3, column 1) in the absence of HHDH indicates that spontaneous formation of **3a** and **4a** is observed, while the yields (<2%) and regioselectivity (**3a**:**4a** = 65:35) are really low. Notably, the SMO-HheG cascade (Figure 3, column 24) generates (*R*)-**3a** in relatively good yield, as well as excellent *α*-regioselectivity (**3a**:**4a** = 96:4). Interestingly, the *β*-regioselectivity in the production of (*S*)-**4a** (**3a**:**4a** = 60:40) is not high in the SMO-HheC cascade (Figure 3, column 11), although the HheC exhibits good *β*-regioselectivity in the azide-mediated kinetic resolution of epoxides (Lutje Spelberg et al., 2001). To our delight, when we tried to construct the SMO-HheCM cascade (Figure 3, column 12) by using an *S*-enantioselective variant HheCM (P84V/F86P/T134A/N176A) mutated from HheC (Guo et al., 2015), (*S*)-**4a** was produced in relatively good yield and high *β*-regioselectivity (**3a**:**4a** = 4:96). Therefore, the HheG and HheCM were chosen as two regiocomplementary HHDHs for combining with SMO, constructing biocatalytic cascades SMO-HheG (BC_*α*_) and SMO-HheCM (BC_*β*_) for synthesizing (*R*)-**3a** and (*S*)-**4a** through asymmetric hydroxyazidation of **1a**, respectively. Subsequently, systematical investigation of the reaction conditions of BC_*α*_ and BC_*β*_ was carried out based on the model reaction (see Tables S2–S7 for details). Under the optimized conditions, (*R*)-**3a** was formed in 90% yield and >99% *ee* by BC_*α*_, and (*S*)-**4a** was produced in 96% yield and >99% *ee* in the case of BC_*β*_ (Figure S2).

**Figure 4:**
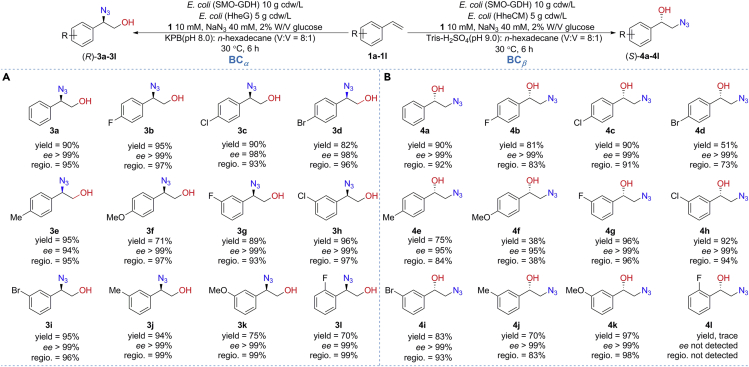
Asymmetric hydroxyazidation of styrenes **1a-1L** catalyzed by BC_*α*_ (A) and BC_*β*_ (B). Reactions were conducted in a biphasic system (48 mL aqueous buffer and 6 mL *n*-hexadecane) containing 10 mM styrenes, 40 mM NaN_3_, 2% W/V glucose, and resting cells *E. coli* (SMO-GDH) (10 g cdw/L) and *E. coli* (HHDH) (5 g cdw/L) at 30°C, 250 rpm for 6 hr. Yield is the isolated yield of the formation of 1,2-azidoalcohol product, obtained by silica gel chromatography. The *ee* and regioselectivity were determined by chiral HPLC.

With the optimum conditions in hand, we next explored the scope of the two biocatalytic cascades for asymmetric hydroxyazidation of alkenes. A series of styrenes **1a-1l** bearing electron-withdrawing groups (R = F, Cl, Br) or electron-donating groups (R = Me, OMe) were tested, and the results are summarized in Figure 4. In the case of BC_*α*_ (Figure 4A), all styrenes perform the transformation to produce the desired enantiopure 1,2-azidoalcohols **3a-3l** in high stereoselectivity and regioselectivity. Halo substituents on the phenyl ring are well tolerated (**3b**-**3d**, **3g**-**3i**, and **3l**). The fluorine group on *ortho-*, *meta-*, and *para-* positions of styrene **1a** with different steric hindrance is also tolerated, yielding the corresponding 1,2-azidoalcohols with 70%, 89%, and 95% yields, respectively. As expected, a broad scope was also found in the case of BC_*β*_ (Figure 4B). Styrenes **1a-1k** are smoothly transformed into the enantiopure 1,2-azidoalcohols **4a-4k**. The resulting low yields of 1,2-azidoalcohols **4d** and **4f** are caused by the poor regioselectivity of HheCM. In addition, conversion of the *ortho-*fluorine-substituted styrene **1l** to the 1,2-azidoalcohol **4l** is unsuccessful because the sterically hindered epoxide intermediate is not tolerated by HheCM. In general, both BC_*α*_ and BC_*β*_-catalyzed asymmetric hydroxyazidations of alkenes are basically completed after reaction for 6 h, affording the corresponding 1,2-azidoalcohols in high yields. The formed enantiopure epoxides in the first step are rapidly converted into the corresponding chiral 1,2-azidoalcohols by the subsequent azide-mediated regioselective ring-opening reaction. Therefore, the by-product vicinal diols generated from the epoxide intermediates by water activation are trace in the biocatalytic cascades. It is noteworthy that all the tested styrenes are converted into the corresponding chiral 1,2-azidoalcohols (except for **4l**) in excellent optical purity.

**Figure 5:**
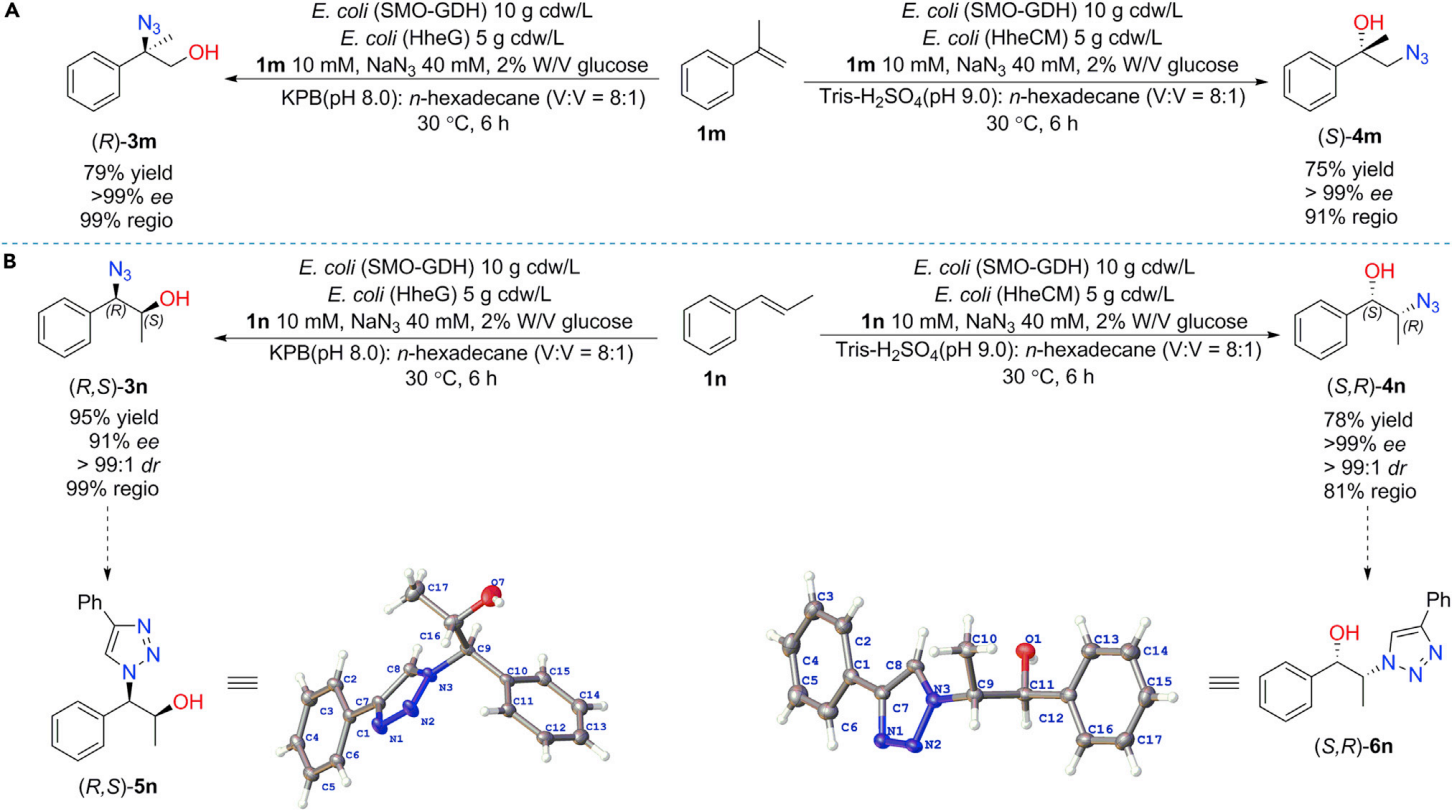
Asymmetric hydroxyazidation of *α*-methylstyrene **1m** (A) and *trans*-*β*-methylstyrene **1n** (B) catalyzed by BC_*α*_ and BC_*β*_ Reactions were conducted in a biphasic system (48 mL aqueous buffer and 6 mL *n*-hexadecane) containing 10 mM alkenes, 40 mM NaN_3_, 2% W/V glucose, and resting cells *E. coli* (SMO-GDH) (10 g cdw/L) and *E. coli* (HHDH) (5 g cdw/L) at 30°C, 250 rpm for 6 hr. Yield is the isolated yield of the formation of 1,2-azidoalcohol product, obtained by silica gel chromatography. The *ee*, *dr*, and regioselectivity were determined by chiral HPLC. The configurations of (*R*,*S*)-**3n** and (*S*,*R*)-**4n** were determined by further derivatization and single-crystal diffraction of (*R*,*S*)-**5n** and (*S*,*R*)-**6n**, respectively.

Subsequently, we turned our attention to the more sterically hindered substrates, *α*-methylstyrene **1m** and *trans*-*β*-methylstyrene **1n**. Gratifyingly, both BC_*α*_ and BC_*β*_ are able to catalyze the conversion of **1m** into the chiral corresponding 1,2-azidoalcohols (*R*)-**3m** and (*S*)-**4m** in good yields and excellent *ee* (Figure 5A). These results reveal that *α*-methyl substitution of **1a** does not influence the stereoselectivity and regioselectivity of the biocatalytic cascades. To our delight, enantiopure 1,2-azidoalcohols (*R,S*)*-***3n** and (*S,R*)*-***4n** that contain two chiral centers are also smoothly synthesized from **1n** in 95% and 78% yields catalyzed by BC_*α*_ and BC_*β*_, respectively (Figure 5B). More importantly, both BC_*α*_ and BC_*β*_ exhibit good stereoselectivity as well as diastereoselectivity in asymmetric hydroxyazidation of **1n**, yielding (*R,S*)*-***3n** in 91% *ee*, >99:1 *dr* and (*S,R*)*-***4n** in >99% *ee* and >99:1 *dr*, respectively. Absolute configurations of (*R,S*)*-***3n** and (*S,R*)*-***4n** were determined by single-crystal X-ray diffraction analysis of the corresponding derivatives (*R,S*)*-***5n** and (*S,R*)*-***6n**. These results clearly demonstrate that the more challenging internal styrene is also well tolerated, highlighting the applicability of these biocatalytic cascades.

**Figure 6:**
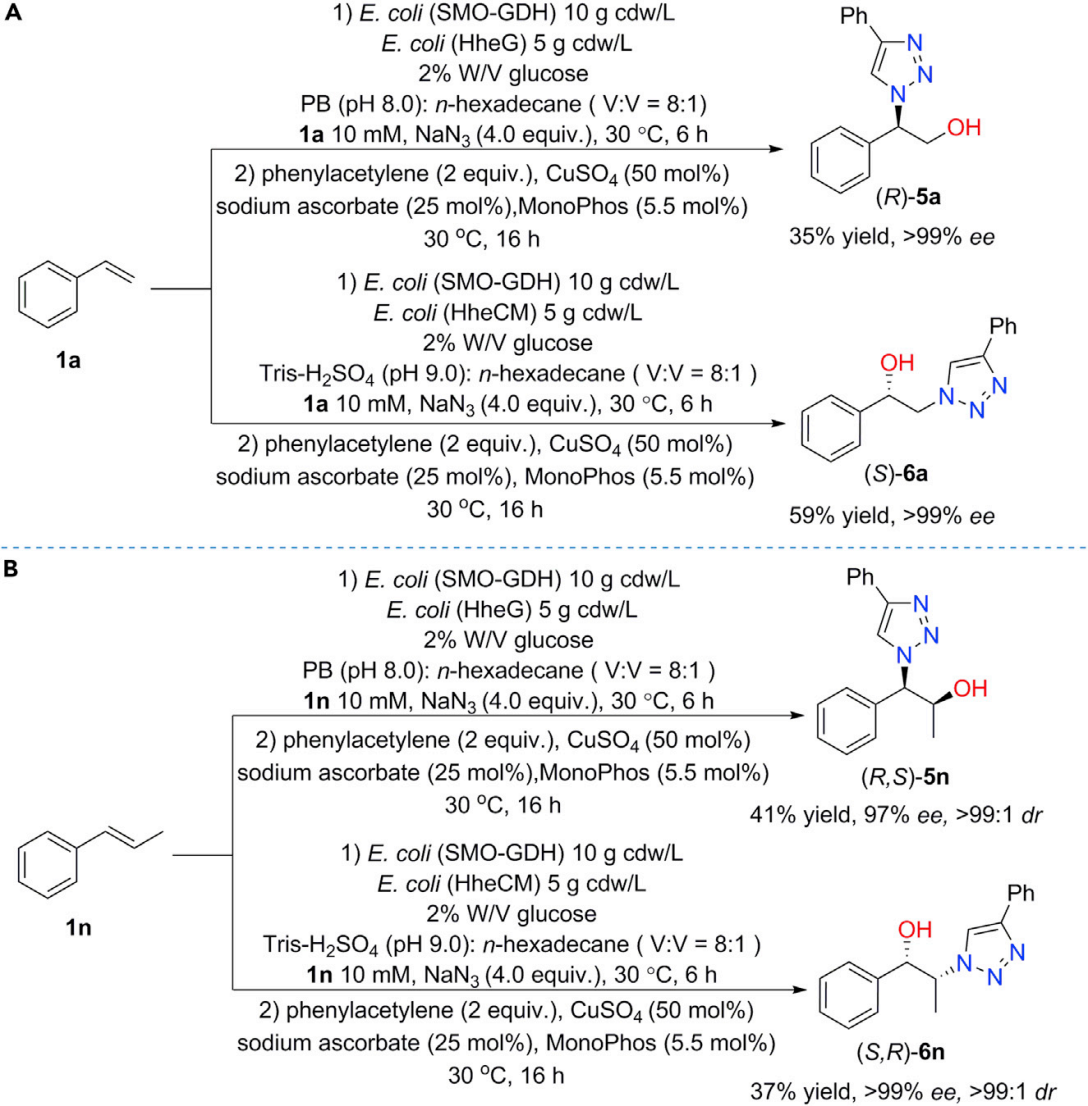
Chemo-enzymatic synthesis of chiral *β*-hydroxytriazoles from alkenes **1a** (A) and **1n** (B) by combining biocatalytic cascade and Cu(I)-catalyzed click reaction. Reactions were conducted in a biphasic system (48 mL aqueous buffer and 6 mL *n*-hexadecane) containing 10 mM alkenes, 40 mM NaN_3_, 2% W/V glucose, and resting cells *E. coli* (SMO-GDH) (10 g cdw/L) and *E. coli* (HHDH) (5 g cdw/L) at 30°C, 250 rpm for 6 hr; then 2 equiv. phenylacetylene, 50 mol% CuSO_4_, 25 mol% sodium ascorbate and 5.5 mol% MonoPhos were added for reaction for another 16 hr (total 22 hr). Yields were the isolated yield of the formation of *β*-hydroxytriazole product, obtained by silica gel chromatography. The *ee* and *dr* were determined by chiral HPLC.

The combination of chemocatalysis and biocatalysis for multistep syntheses shows many advantages such as environmental benefits and high selectivity (Huang et al., 2020; Rudroff et al., 2018). The Cu-catalyzed alkyne-azide cycloaddition “click reaction” occurs in aqueous condition (Tiwari et al., 2016), which is suitable for combining with biocatalysis due to the compatibility of reaction systems. Several chemo-enzymatic systems have been developed for the synthesis of chiral *β*-hydroxytriazoles from epoxides or *α*-haloketones (Campbell-Verduyn et al., 2010; Szymanski et al., 2010). Here, we tried to synthesize chiral *β*-hydroxytriazoles from styrenes through a one-pot chemo-enzymatic system. In this system, after asymmetric hydroxyazidation of styrenes by biocatalytic cascades, a subsequent step is carried out via Cu(I)-catalyzed [2 + 3]-dipolar cycloaddition of the enantiopure 1,2-azidoalcohol with phenylacetylene. To demonstrate the concept, transformations of styrenes **1a** and **1n** to the corresponding chiral *β*-hydroxytriazoles were investigated. As shown in Figure 6A, chiral *β*-hydroxytriazoles (*R*)-**5a** and (*S*)-**6a** are smoothly prepared from **1a** in >99% *ee*. In addition, *trans*-*β*-methylstyrene **1n** is also converted into corresponding *β*-hydroxytriazoles (*R,S*)-**5n** and (*S,R*)-**6n**, both of which are formed in excellent *ee* and *dr* (Figure 6B). To the best of our knowledge, it is the first report of preparing enantiopure *β*-hydroxytriazoles from alkenes.

**Figure 7:**
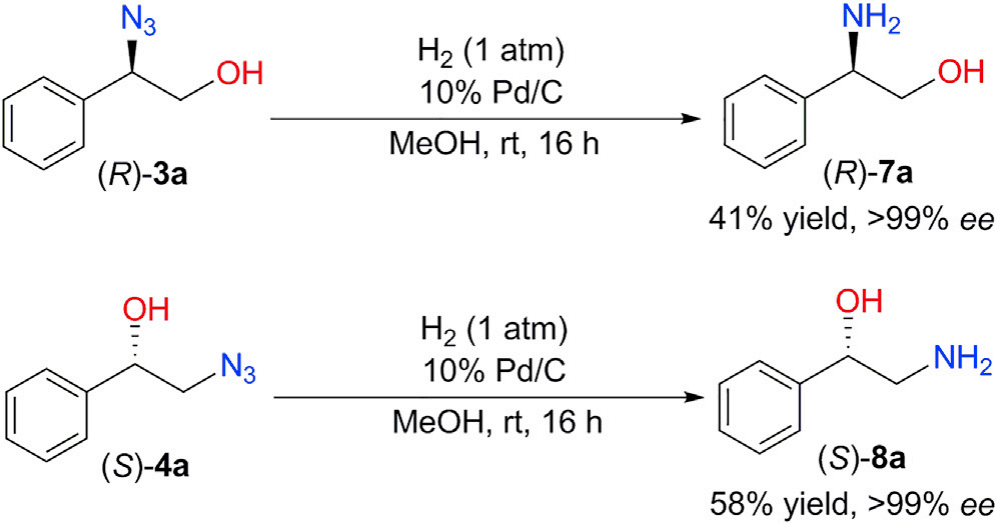
Transformations of chiral 1,2-azidoalcohols (*R*)-**3a** and (*S*)-**4a** to enantiopure 1,2-amino alcohols Reaction condition: (*R*)-**3a** or (*S*)-**4a** (0.92 mmol), 10% Pd/C (70 mg) in MeOH (2 mL) under H_2_ (1 atm) at room temperature, 16 hr. Yield is the isolated yield of the formation of 1,2-amino alcohol product, obtained by silica gel chromatography. The *ee* value was determined by chiral HPLC.

Since enantiopure 1,2-azidoalcohols are synthesized by the biocatalytic cascades, a variety of chiral molecules could be prepared by further transformations. For example, chiral 1,2-amino alcohols are important precursors of many chiral drugs (Legnani and Morandi, 2016; Perricos and Wenzl, 2017) and serve as important chiral ligands and auxiliaries in asymmetric synthesis (Ager et al., 1996). Herein, facile synthesis of chiral 1,2-amino alcohols (*R*)-**7a** (41%, >99% *ee*) and (*S*)**-8a** (58%, >99% *ee*) was achieved via a simple reduction reaction of (*R*)-**3a** and (*S*)-**4a**, respectively (Figure 7). In addition, many other useful chiral heterocyclic scaffolds could be obtained according to previous studies of transformations of 1,2-azidoalcohols (Ariza et al., 2001; Chakraborty et al., 2005; Ittah et al., 1978; Li et al., 2009; Sahasrabudhe et al., 2003; Sun et al., 2015; Wu et al., 2019; Yang and Lu, 2017). These representative transformations clearly demonstrate the versatilities of these enantiopure 1,2-azidoalcohols.

### Conclusions

In summary, we have developed an efficient method for regiodivergent and stereoselective hydroxyazidation of alkenes by two novel biocatalytic cascades, providing a direct and green approach to various enantiopure 1,2-azidoalcohols. The reaction is featured by its high regioselectivity, excellent stereoselectivity, good efficiency, broad substrate scope, easy operation, and mild conditions. We also demonstrated that direct preparation of chiral *β*-hydroxytriazoles from alkenes is feasible by a one-pot chemo-enzymatic synthesis. We anticipate that this biocatalytic cascade strategy could impact the development of asymmetric difunctionalization of alkenes.

## STAR★Methods

### Key resources table

**Table:** 

REAGENT or RESOURCE	SOURCE	IDENTIFIER
**Bacterial and virus strains**		

*Escherichia coli* BL21 (DE3) Competent Cells	Sangon Biotech	Cat#B528414
Recombinant *E. coli* (SMO-GDH) strain (see Table S8)	This paper	N/A
Recombinant *E. coli* (HHDH) strains (see Table S8)	This paper	N/A

**Chemicals, peptides, and recombinant proteins**		

Styrene (**1a**)	TCI	Cat#10289A
1-fluoro-4-vinylbenzene (**1b**)	Innochem	Cat#A31520
1-chloro-4-vinylbenzene (**1c**)	Acros	Cat#110090100
1-bromo-4-vinylbenzene (**1d**)	Adamas	Cat#35574D
1-methyl-4-vinylbenzene (**1e**)	TCI	Cat#71373F
1-methoxy-4-vinylbenzene (**1f**)	Ark Pharm	Cat#AK-46470
1-fluoro-3-vinylbenzene (**1g**)	TCI	Cat#F0409
1-chloro-3-vinylbenzene (**1h**)	aladdin	Cat#C140515
1-bromo-3-vinylbenzene (**1i**)	Innochem	Cat#A76415
1-methyl-3-vinylbenzene (**1j**)	aladdin	Cat#M158347
1-methoxy-3-vinylbenzene (**1k**)	Sigma-Aldrich	Cat#5630
1-fluoro-2-vinylbenzene (**1l**)	aladdin	Cat#F121723
prop-1-en-2-ylbenzene (**1m**)	Alfa aesar	Cat#L03609
(E)-prop-1-en-1-ylbenzene (**1n**)	Acros	Cat#150150010

**Deposited data**		

Crystallographic data of (*R*,*S*)-**5n**	This paper	CCDC: 2074402
Crystallographic data of (*S*,*R*)-**6n**	This paper	CCDC: 2074403

### Resource availability

#### Lead contact

Further information and requests for resources should be directed to and will be fulfilled by the lead contact, Nan-Wei Wan (nanweiwan@zmu.edu.cn).

#### Materials availability

All other data supporting the findings of this study are available within the article and the supplemental information or from the lead contact upon reasonable request.

#### Data and code availability

Crystallographic data (see Tables S9 and S10) of (*R*,*S*)-**5n** (CCDC: 2074402) and (*S*,*R*)-**6n** (CCDC: 2074403) can be obtained free of charge from The Cambridge Crystallographic Data Center (http://www.ccdc.cam.ac.uk/structures). All other data are available from the authors upon reasonable request.

### Method details

#### General experimental information and materials

^1^H-NMR (400 MHz) and ^13^C-NMR (100 MHz) were recorded on Agilent Technologies 400 MR. Chemical shifts were reported in parts per million (ppm) with respect to the residual solvent peak. Signal shapes and splitting patterns were expressed as follows: s = singlet, d = doublet, t = triplet, m = multiplet, dd = doublet of doublets, br. s = broad single. High-resolution mass spectra (HRMS) were recorded by EI, ESI or FI ionization sources. Column chromatography was performed on silica gel (200-400 mesh). Melting points were uncorrected.

Isopropyl-*β*-D-thiogalactopyranoside (IPTG), ampicillin (Amp), streptomycin sulfate (Sm), and kanamycin sulfate (Kan) were purchased from Solarbio (Beijing, China). Unless otherwise noted, all the other reagents and solvents were obtained from commercial suppliers and used without further purification.

Chiral HPLC analysis was performed on Shimadzu LC-20A, equipped with Chiralcel OD-H chiral column (4.6 mmΦ × 250 mmL, particle size 5 μm), Chiralcel OJ-H chiral column (4.6 mmΦ × 250 mmL, particle size 5 μm), Chiralpak AD-H chiral column (4.6 mmΦ × 250 mmL, particle size 5 μm), Chiralpak AS-H chiral column (4.6 mmΦ × 250 mmL, particle size 5 μm), Chiralpak IH chiral column (4.6 mmΦ × 250 mmL, particle size 5 μm), or Chiralpak AS-3 chiral column (4.6 mmΦ × 250 mmL, particle size 3 μm).

#### Safety concerning statements

Organic azides are potentially explosive substances that can decompose with the slight input of energy from external sources. We always keep in mind the following equation when preparing and utilizing organic azides. The equation takes into account all nitrogen atoms in the organic azide, not just those of azido group. We should be careful when handling the organic azides and sodium azide. In addition, we have never experienced a safety problem with these experiments.

$\frac{n\left(\text{C}\right)+n\left(\text{O}\right)}{n\left(\text{N}\right)}\geq 3$, **n** sigifies the number of atoms

#### Enzymes preparation

The *E. coli* (SMO-GDH) strain was constructed by co-expression of styrene monooxygenase (SMO) and glucose dehydrogenase (GDH) using two plasmids pETDuet-1 (Amp^R^) and pCDFDuet-1(Sm^R^). The recombinant plasmids pETDuet-styA-styB contained two subunit genes styA (*Nco*I/*Hind*III) and styB (*Nde*I/*Xho*I) of SMO, and the pCDFDuet-GDH contained GDH gene (*Nde*I/*Xho*I). The two plasmids were transformed into *E. coli* BL21(DE3), and screened on LB plate containing 100 μg/mL Amp and 50 μg/mL Sm. All the recombinant *E. coli* (HHDH) strains were constructed using pET-28b(+) (Kan^R^). The HHDH genes were inserted into the plasmid to construct recombinant plasmids pET-28-HHDH, and followed by transformation into *E. coli* BL21(DE3). All the enzyme genes were synthesized after codon optimization (see Table S8).

Cultivation was carried out using TB medium containing the corresponding resistance (100 μg/mL Amp and 50 μg/mL Sm for *E. coli* (SMO-GDH), 50 μg/mL Kan for *E. coli* (HHDH). After growing at 37°C to an OD_600_ of 0.6–0.8, IPTG was added to the final concentration of 0.2–0.5 mM. The culture was incubated at 25°C with another 12 hr for enyzme expression. Expression analysis of twenty-four recombinant *E. coli* (HHDH) strains was analyzed by SDS-PAGE gels (see Figure S3). The recombinant *E. coli* cells that containing recombinant enzymes were harvested by centrifugation at 8,000 × *g* at 4°C for 5 min. The freshly prepared *E. coli* cells were resuspended for biotransformation with reaction buffer.

#### Chemical synthesis of racemic 1,2-azidoalcohols

##### Synthesis of racemic 1,2-azidoalcohols **3a-3n**

Racemic 1,2-azidoalcohols **3a-3n** were synthesized from alkenes by two reaction steps (Bernasconi et al., 2004; Wang et al., 2016). Step 1: To a 100 mL round bottomed flask, 15 mL CH_2_Cl_2_ containing 3.0 mmol alkene and NaHCO_3_ (1.5 g in 15 mL H_2_O) was added. Then 2 mL CH_2_Cl_2_ containing 3.3 mmol 3-chloroperbenzoic acid (*m*-CPBA) was cautiously added to this solution (ice bath). The reaction mixture was stirred at room temperature for 3 hr. After washing with aqueous Na_2_SO_3_ (1.95 g in 10 mL) for 20 min, the aqueous phase was then extracted with CH_2_Cl_2_ (3 × 15 mL). Afterward, the organic phase was dried over anhydrous Na_2_SO_4_, evaporated at reduced pressure and the resulting mixture was purified by flash chromatography to afford epoxides. Step 2: To a 500 mL round bottomed flask, 89.6 mL ethanol and 22.4 mL dd H_2_O were added. Then epoxides (17.4 mmol), NaN_3_ (34.8 mmol) and NH_4_Cl (34.8 mmol) were added to this solution. The reaction mixture was stirred and refluxed at 60°C for 12 hr. Ethyl acetate (3 × 110 mL) was used to extract the reaction mixture, and the organic phases were combined and washed with saturated NaCl solution (2 × 200 mL). Afterward the organic phase was dried over anhydrous Na_2_SO_4_, evaporated at reduced pressure and the resulting mixture was purified by flash chromatography to afford racemic 1,2-azidoalcohols **3a-3n.**

##### Synthesis of racemic 1,2-azidoalcohols **4a-4n**

Racemic 1,2-azidoalcohols **4a-4l** and **4n** were synthesized from *α-*bromoketones by two reaction steps (Rocha et al., 2015). Step 1: To a 50 mL round bottomed flask, 5 mL DMSO containing 5 mmol *α*-bromoacetophenones was added. Then 15 mmol NaN_3_ (0.99 g) was added to this solution for reaction at room temperature, and the reaction was monitored by TLC (about 20 min). The mixture was poured into 15 mL water and extracted with ethyl acetate (3 × 20 mL). The organic phases were combined, dried over anhydrous Na_2_SO_4_ and evaporated at reduced pressure to afford crude *α*-azido ketones. Step 2: To a 50 mL round bottomed flask, 5 mL methanol and crude *α*-azido ketones were added. Then 6 mmol NaBH_4_ (1.2 eq. to *α*-bromoacetophenones) was added gradually with stirring and cooling (ice bath). The mixture was stirred at ice bath and monitored by TLC (about 30 min). Afterward the reaction was quenched with 25 mL saturated NH_4_Cl solution. Ethyl acetate (3 × 30 mL) was used to extract the mixture, and the organic phases were combined, dried over anhydrous Na_2_SO_4_, evaporated at reduced pressure. The resulting mixture was purified by flash chromatography to afford racemic 1,2-azidoalcohols **4a-4l** and **4n**. The racemic **4m** was obtained accompanying with the racemic **3m**.

#### Biocatalytic synthesis of chiral 1,2-azidoalcohols from alkenes

General procedure for BC_*α*_-catalyzed synthesis of chiral 1,2-azidoalcohols **3a-3n** from alkenes **1a-1n:** To a 250 mL round bottomed flask, 6 mL *n*-hexadecane and 48 mL KPB (100 mM, pH 8.0) containing resting cells *E. coli* (SMO-GDH) (10 g cdw/L), *E. coli* (HheG) (5 g cdw/L) and 2% W/V glucose were added. To this solution, alkenes **1a-1n** was added to a final concentration of 10 mM using DSMO as cosolvent. Then NaN_3_ (2.16 mmol) was added to a final concentration of 40 mM, and the mixture was stirred at 30°C for 6 h. The reaction mixture was then extracted with ethyl acetate (3 × 55 mL), and the organic phases were separated by centrifugation (7000 rpm × 2 min), combined, dried over anhydrous Na_2_SO_4_, and evaporated at reduced pressure. The resulting mixture was purified by flash chromatography (ethyl acetate: petroleum ether = 1:10) to afford chiral 1,2-azidoalcohols **3a-3n**.

**(*R*)-2-azido-2-phenylethan-1-ol (3a)**(Wang et al., 2016)

 Light yellow liquid, 79.3 mg, 90% yield, 95.2% *ee*; [*α*]^25^ = -163.75 (*c* = 0.74, MeOH); The *ee* was determined by HPLC (Chiralpak AD-H, Hexane/*i*-PrOH = 95/5, flow rate 0.5 mL/min, λ = 210 nm, t_(*R*)-**3a**_ = 23.8 min, t_(*S*)-**3a**_ = 25.7 min). ^1^H NMR (400 MHz, CDCl_3_) *δ* 7.44 – 7.31 (m, 5H), 4.67 (dd, *J* = 7.1, 5.7 Hz, 1H), 3.74 (t, *J* = 5.6 Hz, 2H), 2.70 (br. s, 1H). ^13^C NMR (100 MHz, CDCl_3_) *δ* 136.3, 129.0, 128.8, 127.2, 67.9, 66.5. HRMS (ESI): calcd. for C_8_H_9_N_3_ONa [M + Na]^+^ 186.0638; found 186.0638.

**(*R*)-2-azido-2-(4-fluorophenyl)ethan-1-ol (3b)**(Wang et al., 2016)

 Light yellow liquid, 92.9 mg, 95% yield, 99.2% *ee*; [*α*]^25^ = -125.36 (*c* = 0.41, MeOH); The *ee* was determined by HPLC (Chiralpak AD-H, Hexane/*i*-PrOH = 95/5, flow rate 0.5 mL/min, λ = 210 nm, t_(*R*)-**3b**_ = 23.7 min, t_(*S*)-**3b**_ = 27.5 min). ^1^H NMR (400 MHz, CDCl_3_) *δ* 7.35 – 7.27 (m, 2H), 7.12 – 7.04 (m, 2H), 4.66 (dd, *J* = 7.3, 5.4 Hz, 1H), 3.77 – 3.66 (m, 2H), 2.23 (br. s, 1H). ^13^C NMR (100 MHz, CDCl_3_) *δ* 162.9 (d, *J* = 247.6 Hz, 1C), 132.3 (d, *J* = 3.2 Hz, 1C), 129.0 (d, *J* = 8.3 Hz, 1C), 116.0 (d, *J* = 21.6 Hz, 1C). 67.2, 66.5. HRMS (ESI): calcd. for C_8_H_8_FN_3_ONa [M + Na]^+^ 204.0544; found 204.0544.

**(*R*)-2-azido-2-(4-chlorophenyl)ethan-1-ol (3c)**(Wang et al., 2016)

 White solid, 96.0 mg, 90% yield, 97.5% *ee*; [*α*]^25^ = -246.69 (*c* = 0.16, MeOH); mp 76.4-78.1 ºC; The *ee* was determined by HPLC (Chiralpak AD-H, Hexane/*i*-PrOH = 95/5, flow rate 0.5 mL/min, λ = 210 nm, t_(*R*)-**3c**_ = 24.9 min, t_(*S*)-**3c**_ = 19.7 min). ^1^H NMR (400 MHz, CDCl_3_) *δ* 7.35 – 7.31 (m, 2H), 7.25 – 7.20 (m, 2H), 4.60 (dd, *J* = 7.7, 5.0 Hz, 1H), 3.75 – 3.60 (m, 2H), 2.31 (br. s, 1H). ^13^C NMR (100 MHz, CDCl_3_) *δ* 135.0, 134.8, 129.3, 128.7, 67.2, 66.5. HRMS (ESI): calcd. for C_8_H_8_ClN_3_ONa [M + Na]^+^ 220.0246; found 220.0246.

**(*R*)-2-azido-2-(4-bromophenyl)ethan-1-ol (3d)**(Wang et al., 2016)

 White solid, 107.2 mg, 82% yield, 98.2% *ee*; [*α*]^25^ = -105.51 (*c* = 0.36, MeOH); mp 95.9-97.5 ºC; The *ee* was determined by HPLC (Chiralpak AD-H, Hexane/*i-*PrOH = 95/5, flow rate 0.5 mL/min, λ = 210 nm, t_(*R*)-**3d**_ = 26.4 min, t_(*S*)-**3d**_ = 32.0 min). ^1^H NMR (400 MHz, CDCl_3_) *δ* 7.59 – 7.48 (m, 2H), 7.23 – 7.18 (m, 2H), 4.63 (dd, *J* = 7.7, 4.9 Hz, 1H), 3.77 – 3.67 (m, 2H), 2.16 (br. s, 1H). ^13^C NMR (100 MHz, CDCl_3_) *δ* 135.5, 132.2, 128.9, 122.8, 67.2, 66.4. HRMS (ESI): calcd. for C_8_H_8_BrN_3_ONa [M + Na]^+^ 263.9740; found 263.9739.

**(*R*)-2-azido-2-(*p*-tolyl)ethan-1-ol (3e)**(Wang et al., 2016)

 Light yellow liquid, 90.9 mg, 95% yield, 94.3% *ee*; [*α*]^25^ = -163.29 (*c* =0.42, MeOH); The *ee* was determined by HPLC (Chiralpak AD-H, Hexane/*i*-PrOH = 95/5, flow rate 0.5 mL/min, λ = 210 nm, t_(*R*)-**3e**_ = 23.6 min, t_(*S*)-**3e**_ = 29.3 min). ^1^H NMR (400 MHz, CDCl_3_) *δ* 7.22 (s, 4H), 4.70 – 4.57 (m, 1H), 3.77 – 3.68 (m, 2H), 2.36 (s, 3H), 2.20 (br. s, 1H). ^13^C NMR (100 MHz, CDCl_3_) *δ* 138.7, 133.3, 129.7, 127.2, 67.8, 66.5, 21.3. HRMS (ESI): calcd. for C_9_H_11_N_3_ONa [M + Na]^+^ 200.0795; found 200.0796.

**(*R*)-2-azido-2-(4-methoxyphenyl)ethan-1-ol (3f)**(Wang et al., 2016)

 White solid, 74.1 mg, 71% yield, 99.4% *ee*; [*α*]^25^ = -119.51 (*c* =0.25, MeOH); mp 104.5 – 106.2 ºC; The *ee* was determined by HPLC (Chiralpak AD-H, Hexane/*i*-PrOH = 95/5, flow rate 0.5 mL/min, λ = 210 nm, t_(*R*)-**3f**_ = 34.9 min, t_(*S*)-**3f**_ = 42.6 min). ^1^H NMR (400 MHz, CDCl_3_) *δ* 7.36 – 7.30 (m, 2H), 7.02 – 6.96 (m, 2H), 4.70 (dd, *J* = 7.3, 5.7 Hz, 1H), 3.89 (s, 3H), 3.81 – 3.76 (m, 2H), 2.09 (br. s, 1H). ^13^C NMR (100 MHz, CDCl_3_) *δ* 159.9, 128.6, 128.3, 114.4, 67.5, 66.5, 55.4. HRMS (EI): calcd. for C_9_H_11_N_3_O_2_ [M]^+^ 193.0846; found 193.0845.

**(*R*)-2-azido-2-(3-fluorophenyl)ethan-1-ol (3g)**

 Light yellow liquid, 87.1 mg, 89% yield, 99.9% *ee*; [*α*]^25^ = -124.82 (*c* =0.63, MeOH); The *ee* was determined by HPLC (Chiralpak AS-3, Hexane/*i*-PrOH = 95/5, flow rate 0.5 mL/min, λ=210nm, t_(*R*)-**3g**_ = 27.8 min, t_(*S*)-**3g**_ = 25.1 min). ^1^H NMR (400 MHz, CDCl_3_) *δ* 7.40 – 7.32 (m, 1H), 7.14 – 7.00 (m, 3H), 4.67 (dd, *J* = 7.9, 4.7 Hz, 1H), 3.85 – 3.65 (m, 2H), 2.23 (br. s, 1H). ^13^C NMR (100 MHz, CDCl_3_) *δ* 163.1 (d, *J* = 247.2 Hz, 1C), 139.0 (d, *J* = 7.0 Hz, 1C), 130.7 (d, *J* = 8.3 Hz, 1C), 122.9 (d, *J* = 3.0 Hz, 1C), 115.8 (d, *J* = 21.1 Hz, 1C), 114.3 (d, *J* = 22.3 Hz, 1C), 67.3 (d, *J* = 1.9 Hz, 1C), 66.5. HRMS (ESI): calcd. for C_8_H_8_FN_3_ONa [M + Na]^+^ 204.0542; found 204.0542.

**(*R*)-2-azido-2-(3-chlorophenyl)ethan-1-ol (3h)**

 Light yellow liquid, 102.4 mg, 96% yield, 99.9% *ee*; [*α*]^25^ = -122.92 (*c* = 0.51, MeOH); The *ee* was determined by HPLC (Chiralpak AS-3, Hexane/*i*-PrOH = 95/5, flow rate 0.5 mL/min, λ = 210 nm, t_(*R*)-**3h**_ = 29.4 min, t_(*S*)-**3h**_ = 25.9 min). ^1^H NMR (400 MHz, CDCl_3_) *δ* 7.37 – 7.28 (m, 3H), 7.22 (s, 1H), 4.64 (dd, *J* = 7.9, 4.7 Hz, 1H), 3.77 – 3.67 (m, 2H), 2.43 (br. s, 1H). ^13^C NMR (100 MHz, CDCl_3_) *δ* 138.5, 135.0, 130.3, 129.0, 127.4, 125.4, 67.2, 66.5. HRMS (FI): calcd. for C_8_H_8_N_3_OCl [M]^+^ 197.0350; found 197.0354.

**(*R*)-2-azido-2-(3-bromophenyl)ethan-1-ol (3i)**(Wang et al., 2016)

 Yellow liquid, 124.2 mg, 95% yield, 99.9% *ee*; [*α*]^25^ = -122.49 (*c* = 0.72, MeOH); The *ee* was determined by HPLC (Chiralpak OD-H, Hexane/*i*-PrOH = 95/5, flow rate 0.5 mL/min, λ = 210 nm, t_(*R*)-**3i**_ = 26.5 min, t_(*S*)-**3i**_ = 29.1 min). ^1^H NMR (400 MHz, CDCl_3_) *δ* 7.49 – 7.42 (m, 2H), 7.26 – 7.21 (m, 2H), 4.60 (dd, *J* = 7.9, 4.7 Hz, 1H), 3.75 – 3.61 (m, 2H), 2.75 (br. s, 1H). ^13^C NMR (100 MHz, CDCl_3_) *δ* 138.7, 131.8, 130.5, 130.2, 125.8, 123.0, 67.0, 66.3. HRMS (ESI): calcd. for C_8_H_9_BrN_3_O [M + H]^+^ 241.9924; found 241.9929.

**(*R*)-2-azido-2-(*m*-tolyl)ethan-1-ol (3j)**

 Yellow liquid, 90.0 mg, 94% yield, 99.5% *ee*; [*α*]^25^ = -155.69 (*c* = 0.50, MeOH); The *ee* was determined by HPLC (Chiralpak OJ-H, Hexane/*i*-PrOH = 95/5, flow rate 0.5 mL/min, λ = 210 nm, t_(*R*)-**3j**_ = 21.2 min, t_(*S*)-**3j**_ = 23.5 min). ^1^H NMR (400 MHz, DMSO-*d*_*6*_) *δ* 7.28 – 7.23 (m, 1H), 7.16 – 7.10 (m, 3H), 5.34 – 5.30 (m, 1H), 4.66 (dd, *J* = 8.2, 4.5 Hz, 1H), 3.69 – 3.55 (m, 2H), 2.30 (s, 3H). ^13^C NMR (100 MHz, DMSO-*d*_*6*_) *δ* 137.7, 137.2, 128.7, 128.4, 127.7, 124.2, 66.9, 65.6, 21.0. HRMS (FI): calcd. for C_9_H_11_N_3_O [M]^+^ 177.0897; found 177.0903.

**(*R*)-2-azido-2-(3-methoxyphenyl)ethan-1-ol (3k)**(Wang et al., 2016)

 Yellow liquid, 78.3 mg, 75% yield, 99.9% *ee*; [*α*]^25^ = -109.85 (*c* = 0.57, MeOH); The *ee* was determined by HPLC (Chiralpak OD-H, Hexane/*i*-PrOH = 95/5, flow rate 0.5 mL/min, λ = 210 nm, t_(*R*)-**3k**_ = 31.9 min, t_(*S*)-**3k**_ = 34.8 min). ^1^H NMR (400 MHz, CDCl_3_) *δ* 7.34 – 7.25 (m, 1H), 6.95 – 6.84 (m, 3H), 4.63 (dd, *J* = 7.6, 5.3 Hz, 1H), 3.81 (s, 3H), 3.76 – 3.70 (m, 2H), 2.53 (br. s, 1H). ^13^C NMR (100 MHz, CDCl_3_) *δ* 160.0, 137.9, 130.0, 119.4, 114.0, 112.9, 67.8, 66.4, 55.3. HRMS (ESI): calcd. for C_9_H_11_N_3_O_2_Na [M + Na]^+^ 216.0743; found 216.0741.

**(*R*)-2-azido-2-(2-fluorophenyl)ethanol (3l)**

 Yellow liquid, 68.5 mg, 70% yield, 98.5% *ee*; [*α*]^25^ = -101.99 (*c* = 0.87 MeOH); The *ee* was determined by HPLC (Chiralpak AS-3, Hexane/*i*-PrOH = 95/5, flow rate 0.5 mL/min, λ = 210 nm, t_(*R*)-**3l**_ = 25.9 min, t_(*S*)-**3l**_ = 23.3 min). ^1^H NMR (400 MHz, CDCl_3_) *δ* 7.44 – 7.38 (m, 1H), 7.36 – 7.28 (m, 1H), 7.21 – 7.15 (m, 1H), 7.12 – 7.05 (m, 1H), 5.02 (dd, *J* = 8.1, 4.2 Hz, 1H), 3.85 – 3.69 (m, 2H), 2.76 (br. s, 1H). ^13^C NMR (100 MHz, CDCl_3_) *δ* 160.1 (d, *J* = 247.2 Hz, 1C), 130.2 (d, *J* = 8.3 Hz, 1C), 128.5 (d, *J* = 3.6 Hz, 1C), 124.7 (d, *J* = 3.7 Hz, 1C), 123.6 (d, *J* = 13.8 Hz, 1C), 115.8 (d, *J* = 21.7 Hz, 1C), 65.3 (d, *J* = 1.4 Hz, 1C), 61.4 (d, *J* = 1.8 Hz, 1C). HRMS (FI): calcd. for C_8_H_8_FN_3_O [M]^+^ 181.0646; found 181.0652.

**(*R*)-2-azido-2-phenylpropan-1-ol (3m)**(Yukio et al., 2003)

 Yellow liquid, 75.6 mg, 79% yield, 99.9% *ee*; [*α*]^25^ = -49.10 (*c* = 0.58, MeOH); The *ee* was determined by HPLC (Chiralpak AD-H, Hexane/*i*-PrOH = 95/5, flow rate 0.5 mL/min, λ = 210 nm, t_(*R*)-**3m**_ = 24.6 min, t_(*S*)-**3m**_ = 25.6 min). ^1^H NMR (400 MHz, CDCl_3_) *δ* 7.48 – 7.29 (m, 5H), 3.71 (d, *J* = 11.5 Hz, 1H), 3.63 (d, *J* = 11.5 Hz, 1H), 1.93 (br. s, 1H), 1.74 (s, 3H).^13^C NMR (100 MHz, CDCl_3_) *δ* 140.9, 128.9, 128.1, 126.1, 70.7, 68.0, 21.5. HRMS (FI): calcd. for C_9_H_11_N_3_O [M]^+^ 177.0897; found 177.0900.

**(1*R*,2*S*)-1-azido-1-phenylpropan-2-ol (3n)**(Sayyed and Sudalai, 2004)

 Yellow liquid, 90.9 mg, 95% yield, > 99:1 *dr*, 91.3% *ee*; [*α*]^25^ = -123.23 (*c* = 0.68, MeOH); The *dr* and *ee* were determined by HPLC (Chiralpak OD-H, Hexane/*i*-PrOH = 95/5, flow rate 0.5 mL/min, λ = 210 nm, t_(1*R,*2*S*)-**3n**_ = 20.1min, t_(1*S,*2*R*)-**3n**_ = 22.1 min). ^1^H NMR (400 MHz, CDCl_3_) *δ* 7.44 -7.32 (m, 5H), 4.47 (d, *J* = 5.6 Hz, 1H), 4.00 – 3.92 (m, 1H), 2.05 (br. s, 1H), 1.17 (d, *J* = 6.3 Hz, 3H)).^13^C NMR (100 MHz, CDCl_3_) *δ* 136.3, 128.9, 128.7, 127.9, 71.5, 70.6, 18.6. HRMS (FI): calcd. for C_9_H_11_N_3_O [M]^+^ 177.0897; found 177.0904.

General procedure for BC_*β*_-catalyzed synthesis of chiral 1,2-azidoalcohols **4a-4n** from alkenes **1a-1n**: To a 250 mL round bottomed flask, 6 mL *n*-hexadecane and 48 mL Tris-H_2_SO_4_ (100 mM, pH 9.0) containing resting cells *E. coli* (SMO-GDH) (10 g cdw/L), *E. coli* (HheCM) (5 g cdw/L) and 2% W/V glucose were added. To this solution alkenes **1a-1n** was added to a final concentration of 10 mM using DSMO as cosolvent. Then NaN_3_ (2.16 mmol) was added to a final concentration of 40 mM, and the mixture was stirred at 30 ^o^C for 6 h. The reaction mixture was then extracted with ethyl acetate (3 × 55 mL), and the organic phases were separated by centrifugation (7000 rpm × 2 min), combined, dried over anhydrous Na_2_SO_4_, and evaporated at reduced pressure. The resulting mixture was purified by flash chromatography (ethyl acetate: petroleum ether = 1:30) to afford chiral 1,2-azidoalcohols **4a-4n**.

**(*S*)-2-azido-1-phenylethan-1-ol (4a)**(Tae Cho et al., 2002)

 Light yellow liquid, 79.3 mg, 90% yield, 99.9% *ee*; [*α*]^25^ = +65.76 (*c* = 0.93, MeOH); The *ee* was determined by HPLC (Chiralpak OD-H, Hexane/*i*-PrOH = 95/5, flow rate 0.5 mL/min, λ = 210 nm, t_(*S*)-**4a**_ = 37.3 min, t_(*R*)-**4a**_ = 32.7 min). ^1^H NMR (400 MHz, CDCl_3_) *δ* 7.43 – 7.30 (m, 5H), 4.85 (dd, *J* = 8.0, 4.0 Hz, 1H), 3.51 – 3.38 (m, 2H), 2.69 (br. s, 1H). ^13^C NMR (100 MHz, CDCl_3_) *δ* 140.7, 128.8, 128.4, 126.0, 73.5, 58.1. HRMS (FI): calcd. for C_8_H_9_N_3_O [M]^+^ 163.0740; found 163.0740.

**(*S*)-2-azido-1-(4-fluorophenyl)ethan-1-ol (4b)**(Tae Cho et al., 2002)

 Light yellow liquid, 79.2 mg, 81% yield, 99.6% *ee*; [*α*]^25^ = +43.16 (*c* = 0.72, MeOH); The *ee* was determined by HPLC (Chiralpak OD-H, Hexane/ *i*-PrOH = 95/5, flow rate 0.5 mL/min, λ = 210 nm, t_(*S*)-**4b**_ = 26.8 min, t_(*R*)-**4b**_ = 23.4 min). ^1^H NMR (400 MHz, CDCl_3_) *δ* 7.41 – 7.26 (m, 2H), 7.12 – 6.97 (m, 2H), 4.82 (dd, *J* = 7.8, 4.4 Hz, 1H), 3.51 – 3.32 (m, 2H), 2.91 (br. s, 1H). ^13^C NMR (100 MHz, CDCl_3_) *δ* 162.6 (d, *J* = 246.6 Hz, 1C), 136.4 (d, *J* = 3.1 Hz, 1C), 127.7 (d, *J* = 8.2 Hz, 1C), 115.6 (d, *J* = 21.5 Hz, 1C), 72.8, 58.1. HRMS (FI): calcd. for C_8_H_8_FN_3_O [M]^+^ 181.0646; found 181.0644.

**(*S*)-2-azido-1-(4-chlorophenyl)ethan-1-ol (4c)**(Tae Cho et al., 2002)

 White solid, 96.2 mg, 90% yield, 99.0% *ee*; [*α*]^25^ = +106.56 (*c* = 0.24, MeOH); mp 46.2-47.8°C. The *ee* was determined by HPLC (Chiralpak OD-H, Hexane/*i*-PrOH = 95/5, flow rate 0.5 mL/min, λ = 210 nm, t_(*S*)-**4c**_ = 31.9 min, t_(*R*)-**4c**_ = 26.4 min).^1^H NMR (400 MHz, CDCl_3_) *δ* 7.38 – 7.33 (m, 2H), 7.33 – 7.27 (m, 2H), 4.85 (dd, *J* = 7.1, 4.7 Hz, 1H), 3.47 – 3.40 (m, 2H), 2.71 (br. s, 1H). ^13^C NMR (100 MHz, CDCl_3_) *δ* 139.1, 134.2, 128.9, 127.4, 72.8, 58.0. HRMS (FI): calcd. for C_8_H_8_ClN_3_O [M]^+^ 197.0350; found 197.0359.

**(*S*)-2-azido-1-(4-bromophenyl)ethan-1-ol (4d)**(Hoff et al., 2020)

 White solid, 66.7 mg, 51% yield, 99.7% *ee*; [*α*]^25^ = +57.95 (*c* = 0.22, MeOH); mp 65.4-67.1°C. The *ee* was determined by HPLC (Chiralpak OD-H, Hexane/*i*-PrOH = 95/5, flow rate 0.5 mL/min, λ = 210 nm, t_(*S*)-**4d**_ = 33.7 min, t_(*R*)-**4d**_ = 29.3 min).^1^H NMR (400 MHz, CDCl_3_) *δ* 7.61 – 7.53 (m, 2H), 7.37 – 7.28 (m, 2H), 4.94 – 4.88 (m, 1H), 3.53 – 3.47 (m, 2H), 2.56 (br. s, 1H). ^13^C NMR (100 MHz, CDCl_3_) *δ* 139.6, 131.9, 127.8, 122.4, 72.9, 58.1. HRMS (ESI): calcd. for C_8_H_8_BrN_3_ONa [M + Na]^+^ 263.9743; found 263.9749.

**(*S*)-2-azido-1-(*p*-tolyl)ethan-1-ol (4e)**(Tae Cho et al., 2002)

 Yellow liquid, 71.8 mg, 75% yield, 94.6% *ee*; [*α*]^25^ = +39.22 (*c* = 0.19, MeOH); The *ee* was determined by HPLC (Chiralpak OD-H, Hexane/*i*-PrOH = 95/5, flow rate 0.5 mL/min, λ = 210 nm, t_(*S*)-**4e**_ = 33.7 min, t_(*R*)-**4e**_ = 27.8 min). ^1^H NMR (400 MHz, CDCl_3_) *δ* 7.28 – 7.22 (m, 2H), 7.22 – 7.16 (m, 2H), 4.84 (dd, *J* = 8.2, 3.9 Hz, 1H), 3.52 – 3.38 (m, 2H), 2.35 (s, 3H), 1.67 (br. s, 1H). ^13^C NMR (100 MHz, CDCl_3_) *δ* 138.3, 137.7, 129.5, 126.0, 73.4, 58.2, 21.3. HRMS (FI): calcd. for C_9_H_11_N_3_O [M]^+^ 177.0897; found 177.0898.

**(*S*)-2-azido-1-(4-methoxyphenyl)ethan-1-ol (4f)**(Ankati et al., 2008)

 Yellow liquid, 39.6 mg, 38% yield, 94.6% *ee*; [*α*]^25^ = +57.78 (*c* = 0.71, MeOH); The *ee* was determined by HPLC (Chiralpak AD-H, Hexane/*i*-PrOH = 95/5, flow rate 0.5 mL/min, λ = 210 nm, t_(*S*)-**4f**_ = 39.2 min, t_(*R*)-**4f**_ = 40.7 min). ^1^H NMR (400 MHz, CDCl_3_) *δ* 7.33 – 7.27 (m, 2H), 6.96 – 6.89 (m, 2H), 4.86 – 4.78 (m, 1H), 3.83 (s, 3H), 3.52 – 3.35 (m, 2H), 2.72 (br. s, 1H). ^13^C NMR (100 MHz, CDCl_3_) *δ* 159.6, 132.9, 127.3, 114.1, 73.0, 58.0, 55.4. HRMS (EI): calcd. for C_9_H_11_N_3_O_2_ [M]^+^ 193.0846; found 193.0846.

**(*S*)-2-azido-1-(3-fluorophenyl) ethanol (4g)**

 Yellow liquid, 93.9 mg, 96% yield, 99.9% *ee*; [*α*]^25^ = +55.46 (*c* = 0.79, MeOH); The *ee* was determined by HPLC (Chiralpak OD-H, Hexane/*i*-PrOH = 95/5, flow rate 0.5 mL/min, λ = 210 nm, t_(*S*)-**4g**_ = 30.4 min, t_(*R*)-**4g**_= 26.5 min). ^1^H NMR (400 MHz, DMSO-*d*_6_) *δ* 7.43 – 7.33 (m, 1H), 7.32 – 7.20 (m, 2H), 7.15 – 7.03 (m, 1H), 5.98 (d, *J* = 4.6 Hz, 1H), 4.89 – 4.81 (m, 1H), 3.37 (d, *J* = 5.7 Hz, 2H). ^13^C NMR (100 MHz, DMSO-*d*_6_) *δ* 162.3 (d, *J* = 243.4 Hz, 1C), 145.8 (d, *J* = 6.8 Hz, 1C), 130.1 (d, *J* = 8.1 Hz, 1C), 122.1 (d, *J* = 2.8 Hz, 1C), 114.1 (d, *J* = 21.0 Hz, 1C), 112.9 (d, *J* = 21.9 Hz, 1C), 71.5 (d, *J* = 1.9 Hz, 1C), 57.0. HRMS (ESI): calcd. for C_8_H_8_FN_3_ONa [M + Na]^+^ 204.0544; found 204.0549.

**(*S*)-2-azido-1-(3-chlorophenyl)ethan-1-ol (4h)**(Tae Cho et al., 2002)

 Yellow liquid, 98.2 mg, 92% yield, 99.9% *ee*; [*α*]^25^ = +80.95 (*c* = 0.72, MeOH); The *ee* was determined by HPLC (Chiralpak OD-H, Hexane/*i*-PrOH = 95/5, flow rate 0.5 mL/min, λ = 210 nm, t_(*S*)-**4h**_ = 34.5 min, t_(*R*)-**4h**_ = 28.4 min). ^1^H NMR (400 MHz, CDCl_3_) *δ* 7.36 (s, 1H), 7.31 – 7.27 (m, 2H), 7.25 – 7.19 (m, 1H), 4.81 (dd, *J* = 6.8, 5.1 Hz, 1H), 3.44 – 3.40 (m, 2H), 2.64 (br. s, 1H). ^13^C NMR (100 MHz, CDCl_3_) *δ* 142.6, 134.7, 130.1, 128.5, 126.2, 124.1, 72.8, 57.9. HRMS (FI): calcd. for C_8_H_8_ClN_3_O [M]^+^ 197.0350; found 197.0357.

**(*S*)-2-azido-1-(3-bromophenyl)ethan-1-ol (4i)**

 Yellow liquid, 108.5 mg, 83% yield, 99.9% *ee*; [*α*]^25^ = +66.24 (*c* = 0.35, MeOH); The *ee* was determined by HPLC (Chiralpak OD-H, Hexane/*i*-PrOH = 95/5, flow rate 0.5 mL/min, λ = 210 nm, t_(*S*)-**4i**_ = 40.6 min, t_(*R*)-**4i**_ = 30.8 min). ^1^H NMR (400 MHz, CDCl_3_) *δ* 7.52 (s, 1H), 7.47–7.40 (m, 1H), 7.30–7.18 (m, 2H), 4.81 (dd, *J* = 6.8, 5.1 Hz, 1H), 3.45–3.33 (m, 2H), 2.59 (br. s, 1H). ^13^C NMR (100 MHz, CDCl_3_) *δ* 142.9, 131.5, 130.4, 129.2, 124.6, 122.9, 72.8, 58.0. HRMS (FI): calcd. for C_8_H_8_BrN_3_O [M]^+^ 240.9845; found 240.9843.

**(*S*)-2-azido-1-(*m*-tolyl)ethan-1-ol (4j)**

 Yellow liquid, 67.0 mg, 70% yield, 99.9% *ee*; [*α*]^25^ = +69.64 (*c* = 0.32, MeOH); The *ee* was determined by HPLC (Chiralpak OJ-H, Hexane/*i*-PrOH = 95/5, flow rate 0.5 mL/min, λ = 210 nm, t_(*S*)-**4j**_ = 37.5 min, t_(*R*)-**4j**_ = 28.9 min). ^1^H NMR (400 MHz, CDCl_3_) *δ* 7.33–7.27 (m, 1H), 7.21–7.15 (m, 3H), 4.82 (dd, *J* = 8.2, 3.7 Hz, 1H), 3.51–3.37 (m, 2H), 2.94 (br. s, 1H), 2.40 (s, 3H). ^13^C NMR (100 MHz, CDCl_3_) *δ* 140.6, 138.4, 129.1, 128.6, 126.6, 123.0, 73.4, 58.0, 21.4. HRMS (FI): calcd. for C_9_H_11_N_3_O [M]^+^ 177.0897; found 177.0894.

**(*S*)-2-azido-1-(3-methoxyphenyl)ethan-1-ol (4k)** (Ankati et al., 2008)

 Yellow liquid, 101.2 mg, 97% yield, 99.9% *ee*; [*α*]^25^ = +58.27 (*c* = 0.38, MeOH); The *ee* was determined by HPLC (Chiralpak OD-H, Hexane/*i*-PrOH = 95/5, flow rate 0.5 mL/min, λ = 210 nm, t_(*S*)-**4k**_ = 37.8 min, t_(*R*)-**4k**_ = 29.9 min). ^1^H NMR (400 MHz, CDCl_3_) *δ* 7.30–7.21 (m, 1H), 6.93–6.87 (m, 2H), 6.86–6.79 (m, 1H), 4.81 (dd, *J* = 8.0, 4.0 Hz, 1H), 3.78 (s, 3H), 3.46–3.37 (m, 2H), 2.51 (br. s, 1H).^13^C NMR (100 MHz, CDCl_3_) *δ* 160.0, 142.4, 129.9, 118.3, 113.9, 111.6, 73.5, 58.1, 55.4. HRMS (FI): calcd. for C_9_H_11_N_3_O_2_ [M]^+^ 193.0846; found 193.0850.

**(*S*)-2-azido-1-(2-fluorophenyl)ethan-1-ol (4L)**

Trace.

**(*S*)-1-azido-2-phenylpropan-2-ol (4m)**

 Yellow liquid, 71.8 mg, 75% yield, 99.7% *ee*; [*α*]^25^ = +46.07 (*c* = 0.42, MeOH); The *ee* was determined by HPLC (Chiralpak AS-H, Hexane/*i*-PrOH = 95/5, flow rate 0.5 mL/min, λ = 210 nm, t_(*S*)-**4m**_ = 18.9 min, t_(*R*)-**4m**_ = 20.6 min). ^1^H NMR (400 MHz, CDCl_3_) *δ* 7.50–7.42 (m, 2H), 7.42–7.35 (m, 2H), 7.34–7.27 (m, 1H), 3.61 (d, *J* = 12.3 Hz, 1H), 3.45 (d, *J* = 12.3 Hz, 1H), 2.39 (br. s, 1H), 1.60 (s, 3H). ^13^C NMR (100 MHz, CDCl_3_) *δ* 144.7, 128.6, 127.6, 124.9, 74.7, 62.2, 27.2. HRMS (FI): calcd. for C_9_H_11_N_3_O [M]^+^ 177.0897; found 177.0900.

**(1*S*,2*R*)-2-azido-1-phenylpropan-1-ol (4n)**

 Yellow liquid, 74.6 mg, 78% yield, > 99:1 *dr*, 99.9% *ee*; [*α*]^25^ = +36.14 (*c* = 0.33, MeOH); The *dr* and *ee* were determined by HPLC (Chiralpak OJ-H, Hexane/*i*-PrOH = 95/5, flow rate 0.5 mL/min, λ = 210 nm, t_(1*S,*2*R*)-**4n**_ = 33.8 min, t_(1*R*,2*S*)-**4n**_ = 30.7 min).^1^H NMR (400 MHz, CDCl_3_) *δ* 7.42–7.29 (m, 5H), 4.74 (d, *J* = 4.5 Hz, 1H), 3.77–3.69 (m, 1H), 2.14 (br. s, 1H), 1.19 (d, *J* = 6.7 Hz, 3H). ^13^C NMR (100 MHz, CDCl_3_) *δ* 140.2, 128.6, 128.2, 126.6, 76.5, 62.5, 13.6. HRMS (FI): calcd. for C_9_H_11_N_3_O [M]^+^ 177.0897; found 177.0895.

#### Chemical synthesis of racemic *β*-hydroxytriazoles

Racemic *β*-hydroxytriazoles **5a**, **6a, 5n** and **6n** were synthesized from the corresponding racemic 1,2-azidoalcohols (Campbell-Verduyn et al., 2010). General procedure: To a 10 mL flask, 3 mL water containing CuSO_4_∙5H_2_O (31.1 mg, 0.123 mmol) and sodium ascorbate (24.6 mg, 0.123 mmol) was added. Then MonoPhos (9.8 mg, 0.027 mmol) was added to this solution, and the mixture was stirred at room temperature for 15 min. Afterward the mixture was transferred to a 100 mL round bottomed flask. To this solution, 76.3 mg (0.49 mmol) 1,2-azidoalcohols **3a**, phenylacetylene (111 μL, 0.98 mmol), 9 mL distilled water and 4.0 mL DMSO were added. Then the mixture was stirred at room temperature for 12 hr, and 12 mL cold water was added to the mixture. The precipitate was filtered off, washed with cold water and purified by flash chromatography to afford racemic *β*-hydroxytriazoles **5a**.

#### Chemoenzymatic synthesis of chiral *β*-hydroxytriazoles from alkenes

Chemoenzymatic synthesis of chiral *β*-hydroxytriazoles (*R*)-**5a** and (*R*,*S*)-**5n**: To a 250 mL round bottomed flask, 6 mL *n*-hexadecane and 48 mL KPB (100 mM, pH 8.0) containing resting cells *E. coli* (SMO-GDH) (10 g cdw/L), *E. coli* (HheG) (5 g cdw/L) and 2% W/V glucose were added. To this solution alkene **1a** or **1n** was added to a final concentration of 10 mM using DSMO as cosolvent. Then NaN_3_ (2.16 mmol) was added to a final concentration of 40 mM, and the mixture was stirred at 30°C for 6 hr.

To a 10 mL flask, 1 mL KPB containing CuSO_4_∙5H_2_O (68.2 mg, 0.27 mmol) and sodium ascorbate (28.0 mg, 0.14 mmol) was added. Then MonoPhos (10.9 mg, 0.03 mmol) was added to this solution, and the mixture was stirred at room temperature for 15 min. Afterward the mixture was transferred into the enzymatic reaction mixture (250 mL round bottomed flask) and phenylacetylene (125 μL, 1.1 mmol) was added, then the mixture was stirred at 30°C for another 16 hr.

The reaction mixture was extracted with ethyl acetate (3 × 60 mL), and the organic phases were separated by centrifugation (7000 rpm × 2 min), combined, dried over anhydrous Na_2_SO_4_, evaporated at reduced pressure. The resulting mixture was purified by flash chromatography (ethyl acetate: petroleum ether = 1:3) to afford chiral *β*-hydroxytriazole (*R*)-**5a** or (1*R,*2*S*)-**5n**.

**(*R*)-2-phenyl-2-(4-phenyl-1*H*-1,2,3-triazol-1-yl) ethan-1-ol (5a)**

 Light yellow solid, 50.1 mg, 35% yield, 99.9% *ee*; [*α*]^25^ = −2.75 (*c* = 0.17, CHCl_3_); mp 116.1–117.9°C. The *ee* was determined by HPLC (Chiralpak OD-H, Hexane/*i*-PrOH = 80/20, flow rate 1 mL/min, λ = 210 nm, t_(*R*)-**5a**_ = 13.1 min, t_(*S*)-**5a**_ = 10.8 min). ^1^H NMR (400 MHz, CDCl_3_) *δ* 7.86–7.81 (m, 2H), 7.80 (s, 1H), 7.51–7.44 (m, 5H), 7.44–7.40 (m, 1H), 7.39–7.33 (m, 2H), 5.77 (dd, *J* = 8.4, 3.7 Hz, 1H), 4.73 (dd, *J* = 12.4, 8.4 Hz, 1H), 4.31 (dd, *J* = 12.5, 3.7 Hz, 1H), 3.25 (br. s, 1H). ^13^C NMR (100 MHz, CDCl_3_) *δ* 136.1, 130.2, 129.3, 129.2, 129.0, 128.5, 127.3, 125.8, 120.8, 67.5, 65.3. HRMS (ESI): calcd. for C_16_H_16_N_3_O [M + H]^+^ 266.1288; found 266.1289.

**(1*R*,2*S*)-1-phenyl-1-(4-phenyl-1*H*-1,2,3-triazol-1-yl) propan-2-ol (5n)**

 Colorless solid, 61.8 mg, 41% yield, > 99:1 *dr*, 97.4% *ee*; [*α*]^25^ = +48.94 (*c* = 0.98, CHCl_3_); mp 132.1–133.8°C. The *dr* and *ee* were determined by HPLC (Chiralpak OJ-H, Hexane/*i*-PrOH = 80/20, flow rate 1 mL/min, λ = 210 nm, t_(1*R*,2*S*)-**5n**_ = 13.4 min, t_(1*S*,2*R*)-**5n**_ = 20.0 min). ^1^H NMR (400 MHz, CDCl_3_) *δ* 7.78–7.71 (m, 3H), 7.47–7.42 (m, 2H), 7.41–7.35 (m, 5H), 7.35–7.25 (m, 1H), 5.40–5.36 (m, 1H), 4.91 (dd, *J* = 6.5, 4.3 Hz, 1H), 3.05 (br. s, 1H), 1.24 (d, *J* = 6.4 Hz, 3H). ^13^C NMR (100 MHz, CDCl_3_) *δ* 147.5, 134.9, 130.3, 129.1, 129.0, 128.9, 128.4, 125.7, 120.5, 70.5, 68.7, 19.9. HRMS (ESI): calcd. for C_17_H_18_N_3_O [M + H]^+^ 280.1444; found 280.1446.

Chemoenzymatic synthesis of chiral *β*-hydroxytriazoles (*S*)-**6a** and (*S*,*R*)-**6n**: To a 250 mL round bottomed flask, 6 mL *n*-hexadecane and 48 mL Tris-H_2_SO_4_ (100 mM, pH 9.0) containing resting cells *E. coli* (SMO-GDH) (10 g cdw/L), *E. coli* (HheCM) (5 g cdw/L) and 2% W/V glucose were added. To this solution alkene **1a** or **1n** was added to a final concentration of 10 mM using DSMO as cosolvent. Then NaN_3_ (2.16 mmol) was added to a final concentration of 40 mM, and the mixture was stirred at 30°C for 6 hr.

To a 10 mL flask, 1 mL Tris-H_2_SO_4_ containing CuSO_4_∙5H_2_O (68.2 mg, 0.27 mmol) and sodium ascorbate (28.0 mg, 0.14 mmol) was added. Then MonoPhos (10.9 mg, 0.03 mmol) was added to this solution, and the mixture was stirred at room temperature for 15 min. Afterward, the mixture was transferred into the enzymatic reaction mixture (250 mL round bottomed flask) and phenylacetylene (125 μL, 1.1 mmol) was added, then the mixture was stirred at 30°C for another 16 hr.

The reaction mixture was extracted with ethyl acetate (3 × 60 mL), and the organic phases were separated by centrifugation (7000 rpm × 2 min), combined, dried over anhydrous Na_2_SO_4_, evaporated at reduced pressure. The resulting mixture was purified by flash chromatography (ethyl acetate: petroleum ether = 1:3) to afford chiral *β*-hydroxytriazole (*S*)-**6a** or (1*S,*2*R*)-**6n**.

**(*S*)-1-phenyl-2-(4-phenyl-1*H*-1,2,3-triazol-1-yl) ethan-1-ol (6a)**

 Light yellow solid, 84.5 mg, 59% yield, 99.9% *ee*; [*α*]^25^ = +9.69 (*c* = 0.45, CHCl_3_); mp 156.5–158.4°C. The *ee* was determined by HPLC (Chiralpak OJ-H, Hexane/*i*-PrOH = 80/20, flow rate 1 mL/min, λ = 210 nm, t_(*S*)-**6a**_ = 22.7 min, t_(*R*)-**6a**_ = 25.1 min). ^1^H NMR (400 MHz, CDCl_3_) *δ* 7.80 (s, 1H), 7.69–7.64 (m, 2H), 7.53–7.48 (m, 2H), 7.48–7.42 (m, 2H), 7.42–7.30 (m, 4H), 5.35 (dd, *J* = 9.2, 3.0 Hz, 1H), 4.69 (dd, *J* = 13.9, 3.0 Hz, 1H), 4.42 (dd, *J* = 13.9, 9.2 Hz, 1H), 3.99 (br. s, 1H). ^13^C NMR (100 MHz, CDCl_3_) *δ* 147.2, 140.4, 130.1, 128.9, 128.8, 128.5, 128.2, 126.1, 125.6, 121.5, 72.7, 57.9. HRMS (ESI): calcd. for C_16_H_16_N_3_O [M + H]^+^ 266.1288; found 266.1288.

**(1*S*,2*R*)-1-phenyl-2-(4-phenyl-1*H*-1,2,3-triazol-1-yl) propan-1-ol (6n)**

 Colorless solid, 55.8 mg, 37% yield, > 99:1 *dr*, 99.9% *ee*; [*α*]^25^ = −10.20 (*c* = 0.98, CHCl_3_); mp 139.9–141.7°C. The *dr* and *ee* were determined by HPLC (Chiralpak OJ-H, Hexane/*i*-PrOH = 80/20, flow rate 1 mL/min, λ = 210 nm, t_(1*S*,2*R*)-**6n**_ = 13.2 min, t_(1*R*,2*S*)-**6n**_ = 16.9 min). ^1^H NMR (400 MHz, CDCl_3_) *δ* 7.78–7.71 (m, 3H), 7.47–7.27 (m, 8H), 5.40–5.36 (m, 1H), 4.91 (dd, *J* = 6.6, 4.5 Hz, 1H), 3.12 (br. s, 1H), 1.23 (d, *J* = 6.4 Hz, 3H). ^13^C NMR (100 MHz, CDCl_3_) *δ* 140.1, 130.3, 128.9, 128.6, 128.2, 128.1, 126.1, 125.6, 119.4, 75.4, 62.7, 13.5. HRMS (ESI): calcd. for C_17_H_18_N_3_O [M + H]^+^ 280.1444; found 280.1444.

#### Transformations of chiral 1,2-azidoalcohols

Synthesis of chiral 1,2-amino alcohols from chiral 1,2-azidoalcohols was performed according to previous method(Tae Cho et al., 2002). In a 100 mL round bottomed flask, a mixture of 1,2-azidoalcohols (*R*)-**3a** or (*S*)-**4a** (150 mg, 0.92 mmol) and 10% Pd/C (70 mg) in 5 mL methanol was hydrogenated using hydrogen balloon at room temperature for 16 hr. The mixture was filtered, and the filtrate was concentrated and purified by flash chromatography (dichloromethane: methanol = 3:1) to afford chiral 1,2-amino alcohols (*R*)-**7a** or (*S*)-**8a**. Racemic 1,2-amino alcohols **7a** and **8a** were also prepared from the corresponding racemic 1,2-azidoalcohols(Tae Cho et al., 2002).

**(*R*)-2-amino-2-phenylethan-1-ol (7a)** (Wang et al., 2016)

 White solid, 51.7 mg, 41% yield, 99.9% *ee*; [*α*]^25^ = −25.48 (*c* = 0.85, MeOH); mp 76.1–77.8°C. The *ee* was determined by HPLC (Chiralpak OJ-H, Hexane/*i*-PrOH = 95/5, flow rate 0.5 mL/min, λ = 210 nm, t_(*R*)-**7a**_ = 30.2 min, t_(*S*)-**7a**_ = 27.8 min). ^1^H NMR (400 MHz, CDCl_3_) *δ* 7.45–7.20 (m, 5H), 4.03 (dd, *J* = 8.4, 4.1 Hz, 1H), 3.72 (dd, *J* = 11.0, 4.1 Hz, 1H), 3.55 (dd, *J* = 11.0, 8.4 Hz, 1H), 2.81 (br. s, 3H). ^13^C NMR (100 MHz, CDCl_3_) *δ* 142.4, 128.6, 127.5, 126.6, 67.8, 57.4. HRMS (ESI): calcd. for C_8_H_12_NO [M + H]^+^ 138.0919; found 138.0912.

**(*S*)-2-amino-1-phenylethan-1-ol (8a)** (Tae Cho et al., 2002)

 Light yellow solid, 73.2 mg, 58% yield, 99.9% *ee*; [*α*]^25^ = 38.70 (*c* = 0.46, MeOH); mp 54.4–56.3°C. The *ee* was determined by HPLC (Chiralpak IH, Hexane/*i*-PrOH = 95/5, flow rate 0.5 mL/min, λ = 210 nm, t_(*S*)-**8a**_ = 39.1 min, t_(*R*)-**8a**_ = 41.4 min). ^1^H NMR (400 MHz, CDCl_3_) *δ* 7.38–7.26 (m, 5H), 4.64 (dd, *J* = 7.8, 3.9 Hz, 1H), 2.97 (dd, *J* = 12.8, 4.0 Hz, 1H), 2.81 (dd, *J* = 12.8, 7.8 Hz, 1H), 2.21 (br. s, 3H). ^13^C NMR (100 MHz, CDCl_3_) *δ* 142.7, 128.5, 127.7, 126.0, 74.4, 49.4. HRMS (ESI): calcd. for C_8_H_12_NO [M + H]^+^ 138.0919; found 138.0912.

## Supplemental information

Document S1. Transparent methods, Figures S1–S3, and Tables S1–S10

## Footnotes

Supplemental information can be found online at https://doi.org/10.1016/j.isci.2021.102883.
