# Supplementary material for: Regiodivergent and stereoselective hydroxyazidation of alkenes by biocatalytic cascades
Source: iScience. 2021 Jul 17;24(8):102883. doi: 10.1016/j.isci.2021.102883 (PMC8353479; doi:10.1016/j.isci.2021.102883)
Supplement: Document S1. Transparent methods, Figures S1–S3, and Tables S1–S10 [file mmc1.pdf]

iScience, Volume *24*

Supplemental information

Regiodivergent and stereoselective hydroxyazidation of alkenes by biocatalytic cascades

Jing-Fei Wu, Nan-Wei Wan, Ying-Na Li, Qing-Ping Wang, Bao-Dong Cui, Wen-Yong Han, and Yong-Zheng Chen

Supplemental information

Regiodivergent and stereoselective hydroxyazidation of alkenes by bio-catalytic cascades

Jing-Fei Wu, Nan-Wei Wan, Ying-Na Li, Qing-Ping Wang, Bao-Dong Cui, Wen-Yong Han, and Yong-Zheng Chen

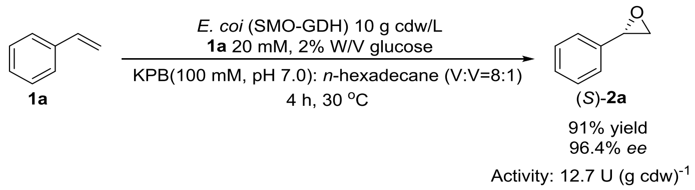

Figure. S1 Asymmetric epxidation of alkene **1a** catalyzed by *E. coli* (SMO-GDH). Yield is the analytic yield of the formation of 1,2-azidoalcohol product. Yield and *ee* were determined by chiral HPLC analysis. Specific activity was determined over the first 30 min. The unit of specific activity was μmol·min^{-1}·g cdw^{-1}.

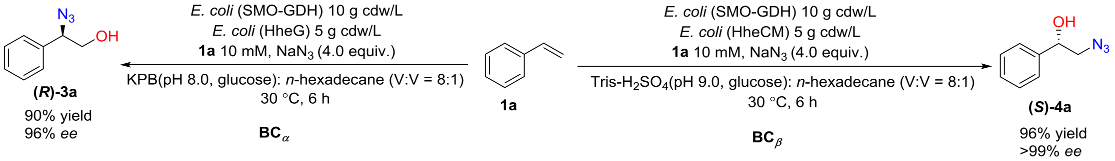

Figure. S2 The optimized conditions for regio- and enantio-selective hydroxyazidation of styrene **1a** by BC$_\alpha$ and BC$_\beta$. Analytic yield and *ee* were determined by chiral HPLC analysis.

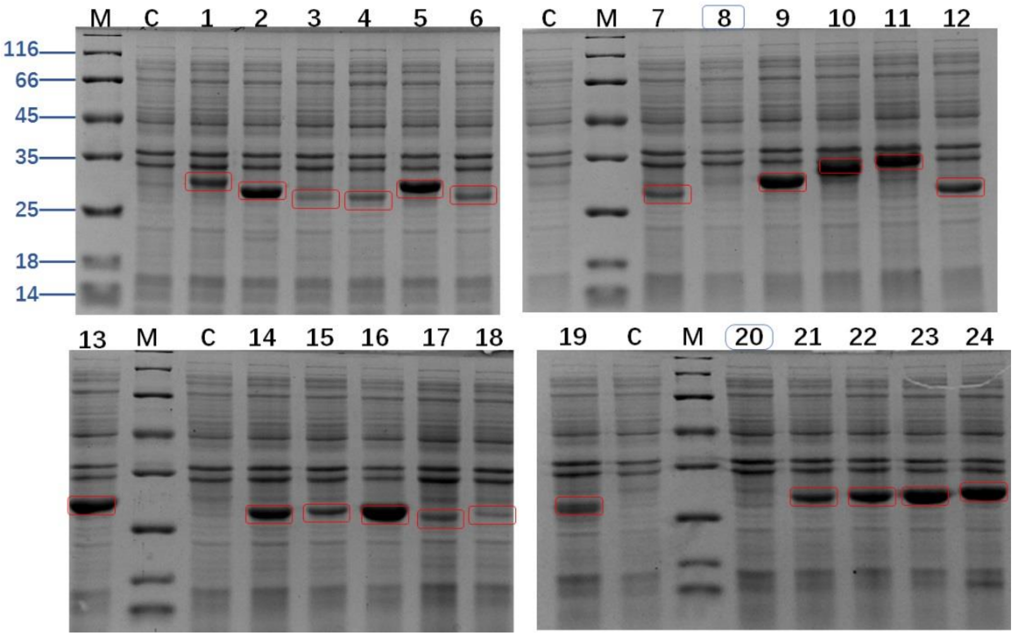

Figure S3. SDS-PAGE analysis of the expression of HHDHs in recombinant *E. coli* strains. Lane M, protein marker. Lane C, the *E. coli* strain containing pET-28b(+) plasmid in the absence of HHDH gene. Lane 1: HheA2. Lane 2: HheA5. Lane 3: HheA6. Lane 4: HheA8. Lane 5: HheA10. Lane 6: HheB3. Lane 7: HheB4. Lane 8: HheB5. Lane 9: HheB6. Lane 10: HheC. Lane 11: HheCM. Lane 12: HheD. Lane 13: HheD6. Lane 14: HheD7. Lane 15: HheD8. Lane 16: HheD9. Lane 17: HheD12. Lane 18: HheD13. Lane 19: HheE. Lane 20: HheE2. Lane 21: HheE5. Lane 22: HheF. Lane 23: HheG. Lane 24: HheG2. Expression of HheB5 (lane 8) and HheE2 (lane 20) was inapparent, and successful expression of the other HHDHs could be observed obviously.

Table S1. Screening of halohydrin dehalogenases for constructing biocatalytic cascades, Related to Figure 3.[a)]

Entry	Catalyst	Yield (%)[b)] (*R*)-**3a**	*ee* (%)[b)] (*R*)-**3a**	Yield (%)[b)] (*S*)-**4a**	*ee* (%)[b)] (*S*)-**4a**	**3a**:**4a** [b)]
1	Blank[c)]	1.5±0.1	99.6	0.8±0.1	97.3	65:35
2	HheA2	4.0±0.5	99.6	0.5±0.1	95.5	89:11
3	HheA5	2.4±0.3	99.7	3.0±0.1	98.0	44:56
4	HheA6	3.9±0.3	99.8	8.9±0.6	99.6	30:70
5	HheA8	5.4±0.4	99.9	9.8±0.4	99.4	36:64
6	HheA10	4.0±0.1	99.7	23.4±0.9	99.7	15:85
7	HheB3	5.8±0.9	99.8	19.7±1.4	99.8	23:77
8	HheB4	5.4±0.1	99.8	18.2±0.2	99.8	23:77
9	HheB5	3.6±0.2	99.7	3.2±0.1	98.8	53:47
10	HheB6	11.5±0.3	99.6	25.5±0.9	99.8	31:69
11	HheC	5.6±0.1	99.9	3.8±0.1	98.3	60:40
12	HheCM	1.4±0.6	98.9	33.6±0.7	99.8	4:96
13	HheD	13.6±0.5	99.9	6.0±0.2	99.5	69:31
14	HheD6	23.0±0.8	99.9	7.4±0.5	99.4	76:24
15	HheD7	18.8±1.3	99.9	1.4±0.1	95.3	93:7
16	HheD8	10.5±1.3	99.9	6.5±0.8	99.8	62:38
17	HheD9	18.5±0.9	99.9	8.3±0.1	99.5	69:31
18	HheD12	13.4±0.4	99.9	5.5±0.2	99.5	71:29
19	HheD13	5.3±0.1	99.9	1.8±0.1	99.0	75:25
20	HheE	1.7±0.1	99.3	29.2±0.8	99.9	6:94
21	HheE2	3.9±0.1	99.7	11.9±0.7	99.6	25:75
22	HheE5	2.8±1.0	99.7	30.6±0.9	99.7	8:92
23	HheF	6.4±0.2	99.9	7.2±0.1	98.9	47:53
24	HheG	50.1±0.2	96.9	2.0±0.1	96.8	96:4
25	HheG2	18.6±0.6	100.0	1.4±0.1	96.3	93:7

[a)]Reactions were carried out in a two-liquid phase system containing 4 mL of K_2HPO_4-KH_2PO_4 buffer (KPB, 100 mM, pH 7.5), 0.5 mL *n*-hexadecane, 20 mM **1a**, 30 mM NaN_3, 2% W/V glucose, resting cells *E. coli* (SMO-GDH) (10 g cdw/L) and *E. coli* (HHDH) (10 g cdw/L) at 30 °C, 250 rpm for 6 h.[b)]Determined by chiral HPLC. Data are mean values of triplicate experiments with error bars indicating standard deviations (n = 3).[c)]Blank reaction was carried out in a two-liquid phase system containing 4 mL of K_2HPO_4-KH_2PO_4 buffer (100 mM, pH 7.5), 0.5 mL *n*-hexadecane, 20 mM **1a**, 30 mM NaN_3, 2% W/V glucose, resting cells *E. coli* (SMO-GDH) (10 g cdw/L) and *E. coli* BL21(DE3) host (10 g cdw/L, in the absence of HHDH) at 30 °C, 250 rpm for 6 h.

Optimization of BC$_\alpha$ for the synthesis of (*R*)-3a.

Table S2. Investigation of reaction buffer (pH) and temperature.[a)]

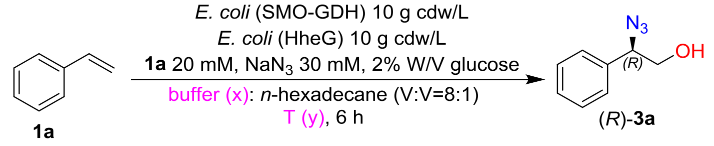

Entry	Buffer[b)]	pH	T	**3a** Yield (%)[c)]	**3a** ee (%)[c)]
1	KPB	7.0	30	53±0.2	95.8
2	KPB	8.0	30	59±1.0	95.2
3	Tris-H_2SO_4	7.0	30	20±0.2	95.8
4	Tris-H_2SO_4	8.0	30	43±0.2	95.9
5	Tris-H_2SO_4	9.0	30	54±0.1	95.6
6	KPB	8.0	25	48±2.7	95.7
7	KPB	8.0	35	56±1.3	94.8
8	KPB	8.0	40	24±1.5	95.7
9	KPB	8.0	45	3±0.9	94.4

a) Reactions were carried out in a two-liquid phase system containing 4 mL of buffer, 0.5 mL *n*-hexadecane, 20 mM **1a**, 30 mM NaN_3, 2% W/V glucose, resting cells *E. coli* (SMO-GDH) (10 g cdw/L) and *E. coli* (HheG) (10 g cdw/L) at 250 rpm for 6 h.
b) KPB buffer (K_2HPO_4-KH_2PO_4,100 mM), Tris-H_2SO_4 buffer (100 mM).
c) Determined by chiral HPLC analysis. Data are mean values of triplicate experiments with error bars indicating standard deviations (n = 3).

Table S3. Investigation of ratio (NaN_3:**1a**) and cell density.[a)]

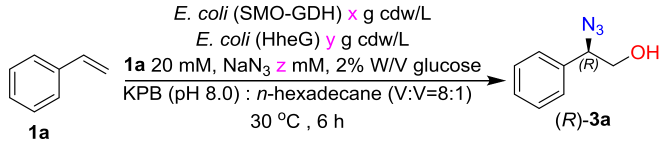

Entry	SMO-GDH	HheG	**1a** (mM)	NaN_3 (mM)	**3a** Yield (%)[b)]	**3a** *ee* (%)[b)]
1	10	10	20	20	44±0.1	95.6
2	10	10	20	30	53±0.2	95.6
3	10	10	20	60	65±0.7	95.7
4	10	10	20	80	73±0.1	95.8
5	5	5	20	80	53±0.8	95.8
6	5	10	20	80	46±0.8	95.8
7	10	5	20	80	76±1.2	95.7
8	20	5	20	80	68±0.5	95.5
9	20	10	20	80	63±1.0	95.7

a)Reactions were carried out in a two-liquid phase system containing 4 mL of KPB buffer (pH 8.0, 100 mM), 0.5 mL *n*-hexadecane, 20 mM **1a**, NaN_3, 2% W/V glucose, resting cells *E. coli* (SMO-GDH) and *E. coli* (HheG) at 30 °C, 250 rpm for 6 h.
b)Determined by chiral HPLC. Data are mean values of triplicate experiments with error bars indicating standard deviations (n = 3).

Table S4. Investigation of substrate concentration and reaction time.[a)]

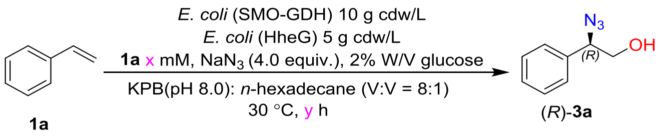

Entry	1a (mM)	6 h		12 h		24 h	
		**3a** Yield (%)[b)]	**3a** *ee* (%)[b)]	**3a** Yield (%)	**3a** *ee* (%)	**3a** Yield (%)	**3a** *ee* (%)
1	20	70±2.1	95.6	71±2.9	95.7	78±3.6	95.8
2	15	77±2.3	95.7	83±2.5	95.8	83±2.7	95.8
3	10	90±0.8	95.7	90±2.6	95.7	93±2.2	95.7

[a)]Reactions were carried out in a two-liquid phase system containing 4 mL of KPB buffer (pH 8.0, 100 mM), 0.5 mL *n*-hexadecane, **1a**, 4.0 equiv. NaN_3, 2% W/V glucose, resting cells *E. coli* (SMO-GDH) (10 g cdw/L) and *E. coli* (HheG) (5 g cdw/L) at 30 °C, 250 rpm for 6 h.
[b)]Determined by chiral HPLC. Data are mean values of triplicate experiments with error bars indicating standard deviations (n = 3).

Optimization of BC$_\beta$ for the synthesis of (*S*)-4a.

Table S5. Investigation of buffer (pH) and temperature.[a)]

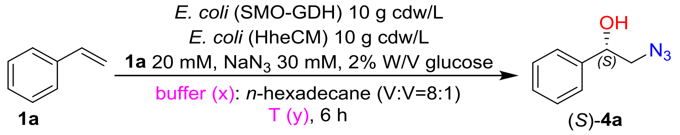

Entry	Buffer[b)]	pH	T	**4a** Yield (%)[c)]	**4a** *ee* (%)[c)]
1	KPB	7.0	30	29±1.1	98.7
2	KPB	8.0	30	35±1.0	99.2
3	Tris-H_2SO_4	7.0	30	24±0.9	97.9
4	Tris-H_2SO_4	8.0	30	30±1.6	99.0
5	Tris-H_2SO_4	9.0	30	43±0.2	99.5
6	Tris-H_2SO_4	9.0	25	36±2.5	99.7
7	Tris-H_2SO_4	9.0	35	28±1.1	98.1
8	Tris-H_2SO_4	9.0	40	23±1.6	98.8
9	Tris-H_2SO_4	9.0	45	13±0.5	98.3

[a)]Reactions were carried out in a two-liquid phase system containing 4 mL of buffer, 0.5 mL *n*-hexadecane, 20 mM **1a**, 30 mM NaN_3, 2% W/V glucose, resting cells *E. coli* (SMO-GDH) (10 g cdw/L) and *E. coli* (HheCM) (10 g cdw/L) at 250 rpm for 6 h.
[b)]KPB buffer (K_2HPO_4-KH_2PO_4,100 mM), Tris-H_2SO_4 buffer (100 mM).
[c)]Determined by chiral HPLC. Data are mean values of triplicate experiments with error bars indicating standard deviations (n = 3).

Table S6. Investigation of ratio (NaN_3:**1a**) and cell density.[a)]

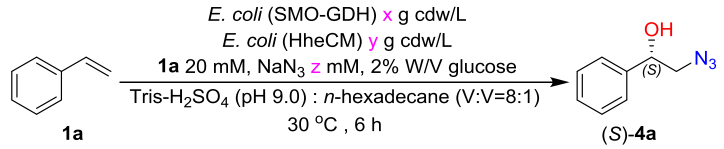

Entry	SMO-GDH	HheCM	**1a** (mM)	NaN_3 (mM)	**4a** Yield (%)[b)]	**4a** *ee* (%)[b)]
1	10	10	20	20	37±1.9	99.7
2	10	10	20	30	41±1.2	99.8
3	10	10	20	60	49±1.5	99.9
4	10	10	20	80	54±1.5	99.9
5	5	5	20	80	46±0.8	99.8
6	5	10	20	80	35±0.8	99.8
7	10	5	20	80	58±1.2	99.7
8	20	5	20	80	60±0.5	99.5
9	20	10	20	80	54±1.0	99.7

[a)]Reactions were carried out in a two-liquid phase system containing 4 mL of Tris-H_2SO_4 buffer buffer (pH 9.0, 100 mM), 0.5 mL *n*-hexadecane, 20 mM **1a**, NaN_3, 2% W/V glucose, resting cells *E. coli* (SMO-GDH) and *E. coli* (HheCM) at 30 °C, 250 rpm for 6 h.
[b)]Determined by chiral HPLC. Data are mean values of triplicate experiments with error bars indicating standard deviations (n = 3).

Table S7. Investigation of substrate concentration and reaction time.[a)]

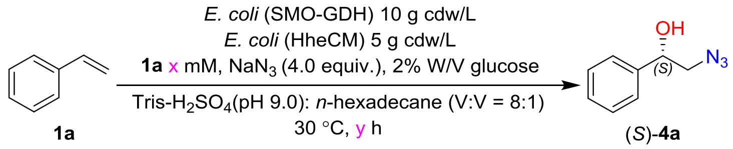

Entry	**1a** (mM)	6 h		12 h		24 h	
		**4a** Yield (%)[b)]	**4a** *ee* (%)[b)]	**4a** Yield (%)	**4a** *ee* (%)	**4a** Yield (%)	**4a** *ee* (%)
1	20	50±1.1	99.5	64±1.0	99.5	65±0.7	99.3
2	15	64±1.2	99.4	79±1.0	99.4	84±1.2	99.4
3	10	96±0.2	99.5	99±1.0	99.5	99±0.4	99.5

[a)]Reactions were carried out in a two-liquid phase system containing 4 mL of Tris-H_2SO_4 buffer buffer (pH 9.0, 100 mM), 0.5 mL *n*-hexadecane, **1a**, 4.0 equiv. NaN_3, 2% W/V glucose, resting cells *E. coli* (SMO-GDH) (10 g cdw/L) and *E. coli* (HheCM) (5 g cdw/L) at 30 °C, 250 rpm for 6 h.
[b)]Determined by chiral HPLC. Data are mean values of triplicate experiments with error bars indicating standard deviations (n = 3).

Table S8. Ecoding genes for the recombinant enezymes expressed in *E. coli* (SMO-GDH) and *E. coli* (HHDH) strains. (Related to STAR Methods)

**Enzymes**	**Source**	**Accession**
SMO	*Pseudomonas* sp. VLB120	AAC23718.1 (styA)
		AAC23719.1 (styB)
GDH	*Bacillus subtilis* QB928	AFQ56330.1
HheA2	*Arthrobacter* sp. AD2	AAK92100
HheA5	*Tistrella mobilis* KA081020-065	WP_014743557
HheA6	*Candidatus Phaeomarinobacter ectocarpi* Ec32	WP_052534782
HheA8	alpha proteobacterium Mf 1.05b.01	WP_051402546
HheA10	*Tsukamurella* sp. 1534	WP_019201195
HheB3	marine metagenome	EBL02020
HheB4	marine metagenome	EBP61646
HheB5	marine metagenome	ECR06649
HheB6	marine metagenome	EDB56284
HheC	*Agrobacterium tumefaciens* AD1	AAK92099
HheCM	mutant of HheC (P84V/F86P/T134A/N176A)	N/A
HheD	*Dechloromonas aromatica* RCB	WP_011285856
HheD6	*Marinobacter nanhaiticus* D15-8W	WP_004579485
HheD7	*Thauera* sp. 27	WP_002926105
HheD8	*Thauera aminoaromatica* S2	WP_004302136
HheD9	*Thauera phenylacetica* B4P	WP_004355811
HheD12	*Pseudomonas pelagia CL-AP6*	WP_022962804
HheD13	*Betaproteobacteria bacterium MOLA814*	WP_023472742
HheE	marine metagenome	ECW41905
HheE2	marine metagenome	EDF62577
HheE5	gamma proteobacterium IMCC3088	WP_009577001
HheF	uncultured bacterium	BAH89601
HheG	*Ilumatobacter coccineus* YM16-304	WP_015443096
HheG2	*Ilumatobacter nonamiensis* YM16-303	WP_040495182

Table S9. Crystal data and structure refinement for (*R*,*S*)-**5n**. (Related to STAR Methods)

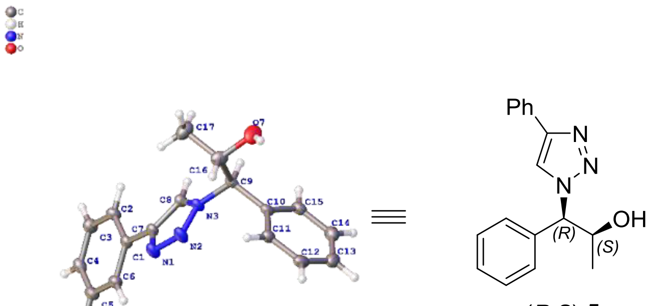

(*R*,*S*)-**5n**

Compound (*R*,*S*)-**5n**	Data
Empirical formula	$C_{17}H_{17}N_3O$
Formula weight	279.33
Temperature/K	100.00(13)
Crystal system	monoclinic
Space group	$P2_1$
a/Å	15.5705(5)
b/Å	5.4950(2)
c/Å	17.8377(7)
α/°	90
β/°	110.219(4)
γ/°	90
Volume/Å^3	1432.15(10)
Z	4
ρ_{calc}g/cm^3	1.296
μ/mm^{-1}	0.660
F(000)	592.0
Crystal size/mm^3	0.14 × 0.1 × 0.08
Radiation	Cu Kα (λ = 1.54184)
2Θ range for data collection/°	5.28 to 147.314
Index ranges	$-19 \leq h \leq 18$, $-6 \leq k \leq 6$, $-21 \leq l \leq 22$
Reflections collected	16405
Independent reflections	5518 [R_{int} = 0.0538, R_{sigma} = 0.0533]
Data/restraints/parameters	5518/1/383
Goodness-of-fit on F^2	1.040
Final R indexes [I>=2σ (I)]	R_1 = 0.0490, wR_2 = 0.1216
Final R indexes [all data]	R_1 = 0.0579, wR_2 = 0.1297
Largest diff. peak/hole / e Å^{-3}	0.19/-0.32
Flack parameter	0.0(3)

Table S10. Crystal data and structure refinement for (*S*,*R*)-**6n**. (Related to STAR Methods)

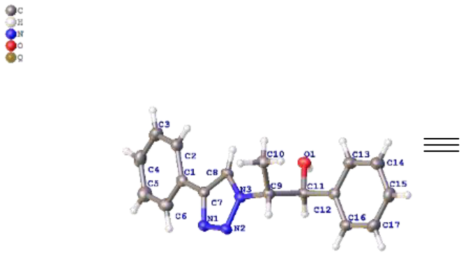

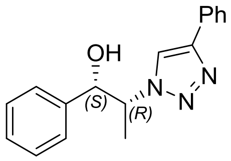

(*S*,*R*)-**6n**

Compound (*S*,*R*)-**6n**	Data
Empirical formula	$C_{17}H_{17}N_3O$
Formula weight	279.33
Temperature/K	100.00(10)
Crystal system	orthorhombic
Space group	$P2_12_12_1$
a/Å	5.8216(2)
b/Å	8.6946(3)
c/Å	29.2365(10)
α/°	90
β/°	90
γ/°	90
Volume/Å^3	1479.85(9)
Z	4
ρ_{calc}g/cm^3	1.254
μ/mm^{-1}	0.639
F(000)	592.0
Crystal size/mm^3	0.13 × 0.12 × 0.11
Radiation	Cu Kα (λ = 1.54184)
2Θ range for data collection/°	6.046 to 146.736
Index ranges	-7 ≤ h ≤ 6, -10 ≤ k ≤ 10, -34 ≤ l ≤ 35
Reflections collected	8699
Independent reflections	2888 [R_{int} = 0.0284, R_{sigma} = 0.0252]
Data/restraints/parameters	2888/0/192
Goodness-of-fit on F^2	1.119
Final R indexes [I>=2σ (I)]	R_1 = 0.0451, wR_2 = 0.1100
Final R indexes [all data]	R_1 = 0.0459, wR_2 = 0.1104
Largest diff. peak/hole / e Å^{-3}	0.22/-0.25
Flack parameter	0.22(13)

HPLC spectra

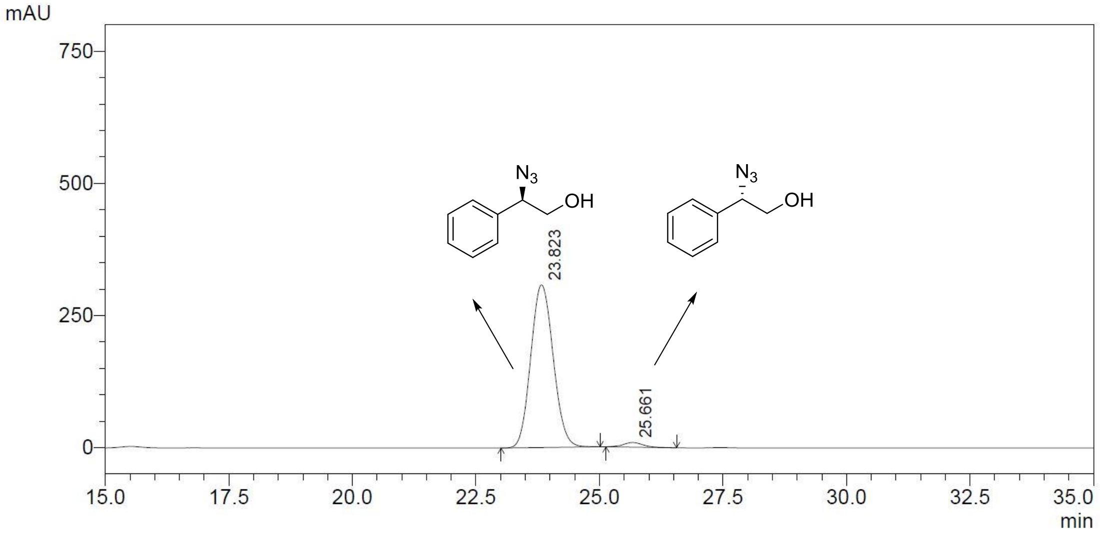

PDA

ID#	Rt. Time	Area	Height	Area %
1	23.823	9725172	307656	97.597
2	25.661	239421	8718	2.403

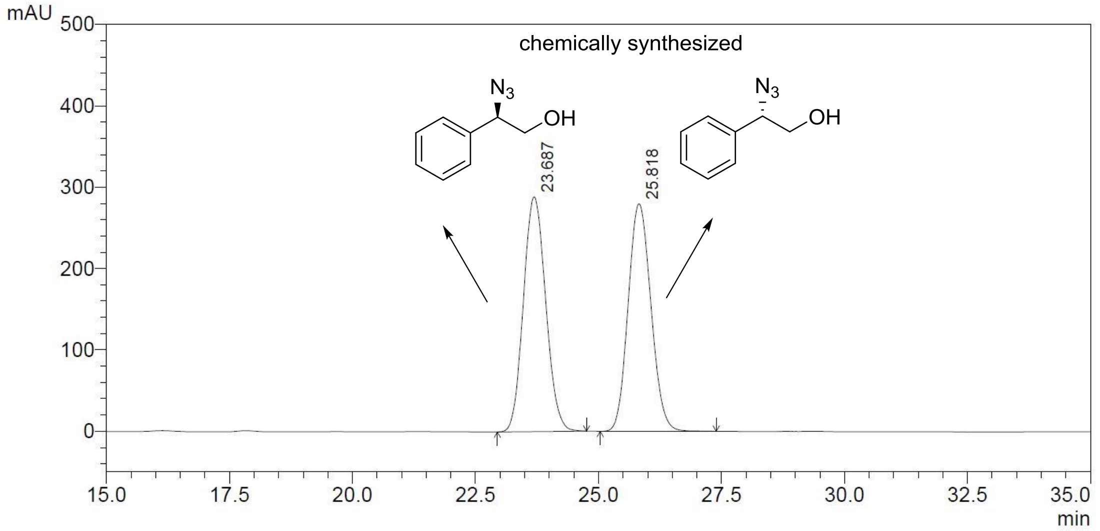

PDA

ID#	Rt. Time	Area	Height	Area %
1	23.687	9101622	288428	50.198
2	25.818	9029917	279752	49.802

Chiral HPLC analysis: Chiralcel AD-H (Hexane/*i*-PrOH = 95/5; 0.5 mL/min; λ = 210 nm; $t_{(R)\text{-}\mathbf{3a}}$ = 23.8 min, $t_{(S)\text{-}\mathbf{3a}}$ = 25.7 min).

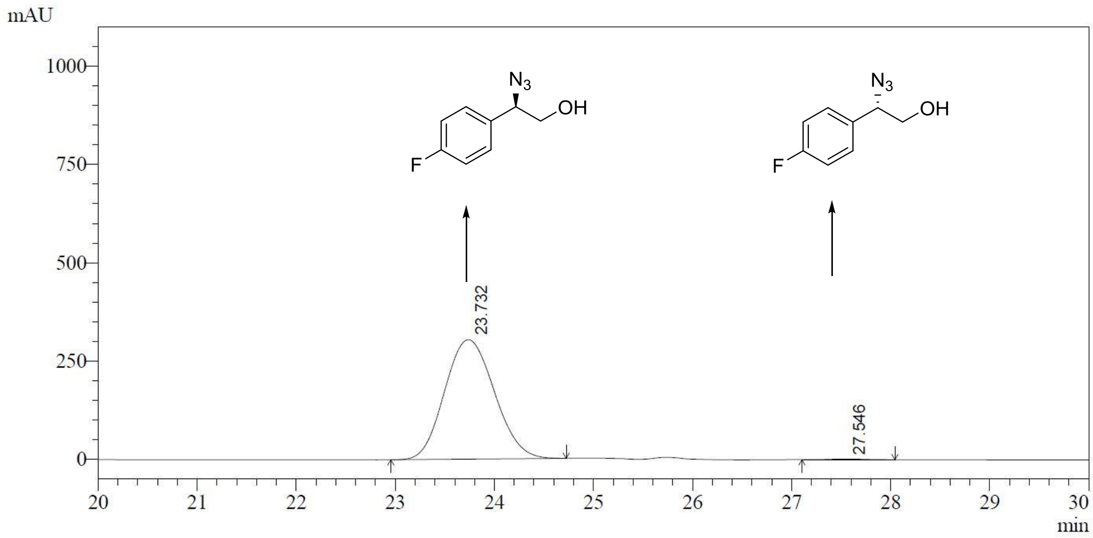

PDA

ID#	Rt. Time	Area	Height	Area %
1	23.732	10603311	303512	99.588
2	27.546	43846	1564	0.412

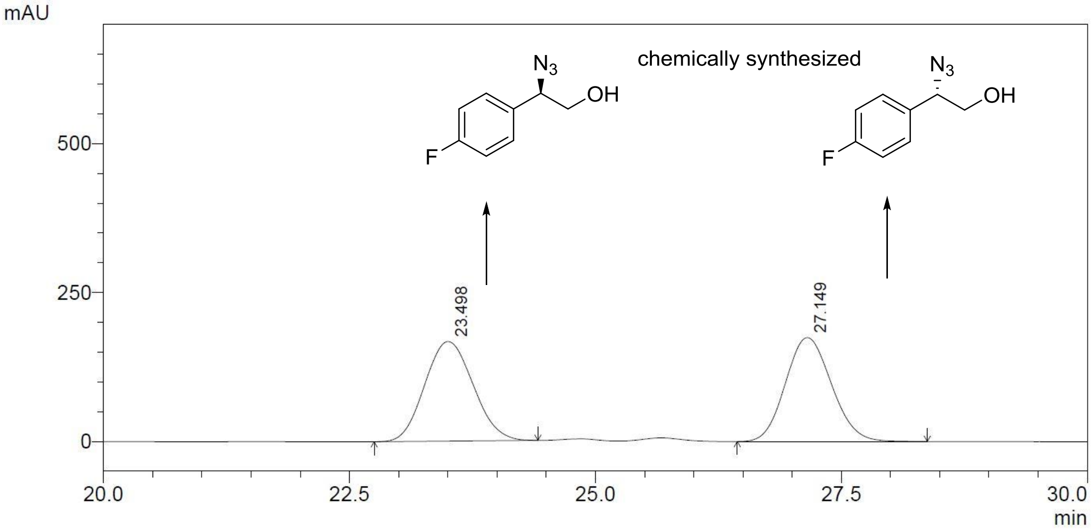

PDA

ID#	Rt. Time	Area	Height	Area %
1	23.498	5723677	167152	50.199
2	27.149	5678319	174408	49.801

Chiral HPLC analysis: Chiralcel AD-H (Hexane/*i*-PrOH = 95/5; 0.5 mL/min; λ = 210 nm; $t_{(R)\text{-}\mathbf{3b}}$ = 23.7 min, $t_{(S)\text{-}\mathbf{3b}}$ = 27.5 min).

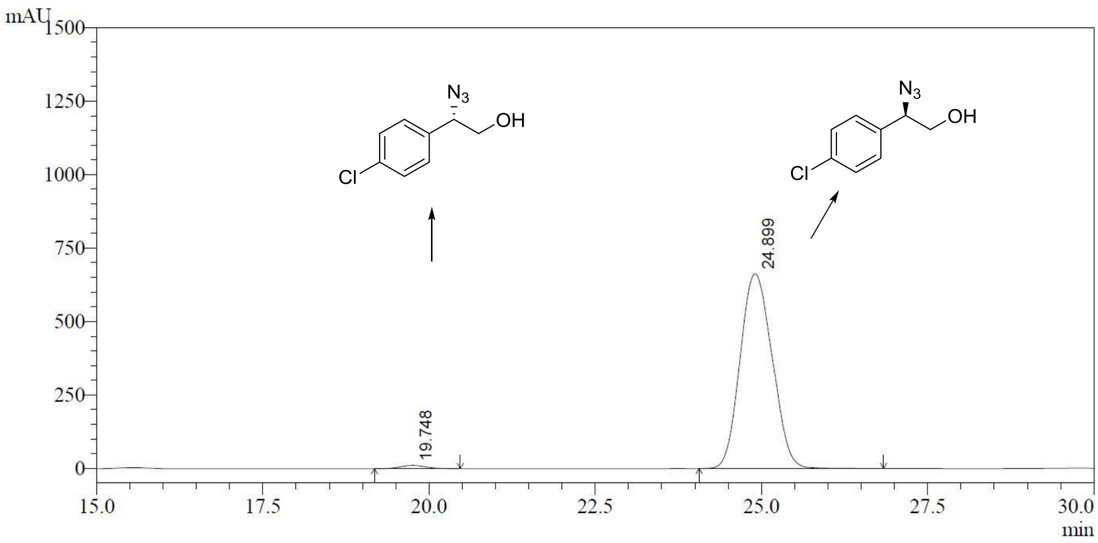

PDA

ID#	Rt. Time	Area	Height	Area %
1	19.748	287381	10960	1.275
2	24.899	22257314	662278	98.725

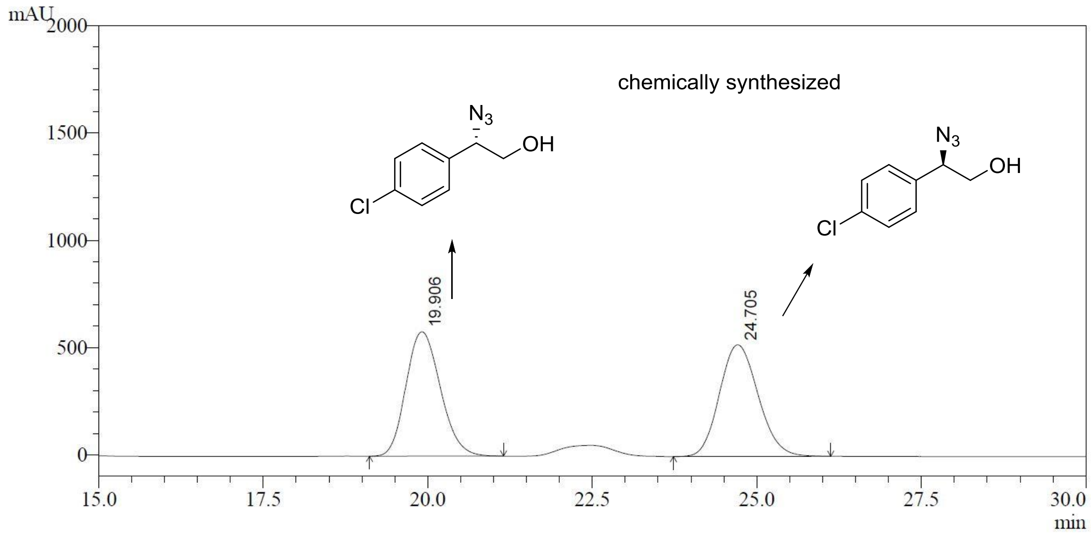

PDA

ID#	Rt. Time	Area	Height	Area %
1	19.906	21141681	579486	49.868
2	24.705	21253243	519659	50.132

Chiral HPLC analysis: Chiralcel AD-H (Hexane/*i*-PrOH = 95/5; 0.5 mL/min; λ = 210 nm; $t_{(R)\text{-}\mathbf{3c}}$ = 24.9 min, $t_{(S)\text{-}\mathbf{3c}}$ = 19.7 min).

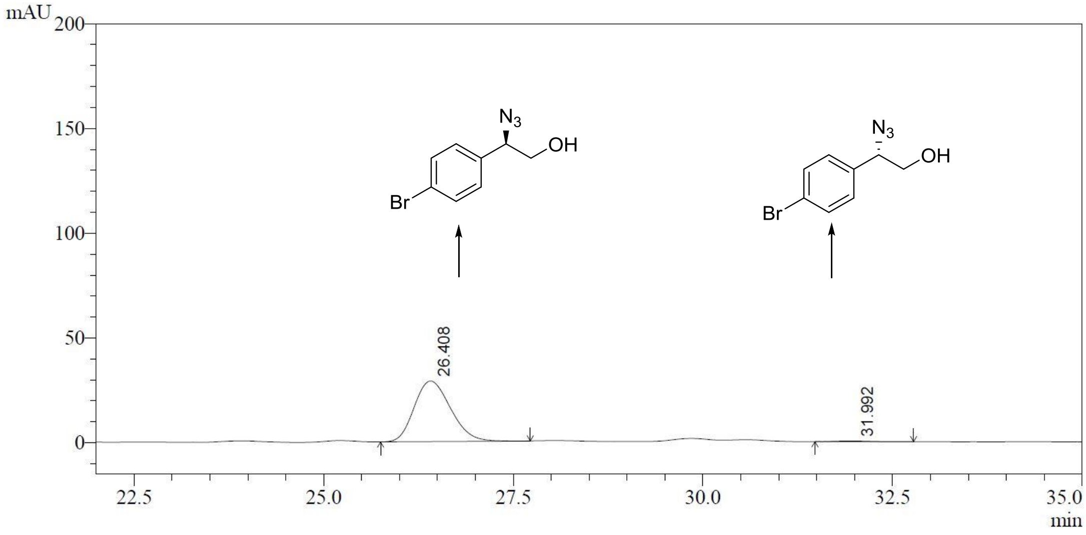

PDA

ID#	Rt. Time	Area	Height	Area %
1	26.408	964094	28921	99.094
2	31.992	8819	288	0.906

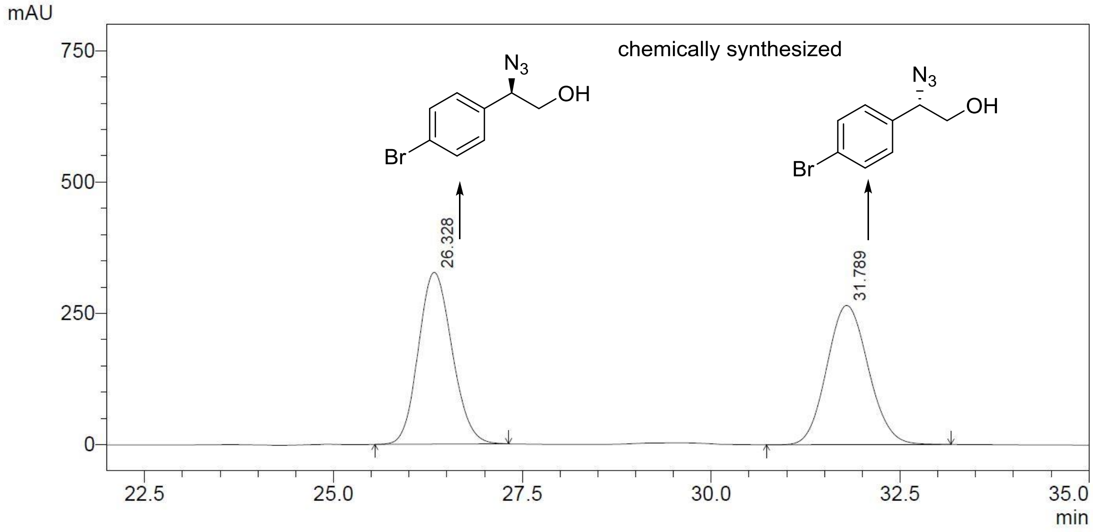

PDA

ID#	Rt. Time	Area	Height	Area %
1	26. 328	10161833	327003	50. 035
2	31. 789	10147723	264924	49. 965

Chiral HPLC analysis: Chiralcel AD-H (Hexane/*i*-PrOH = 95/5; 0.5 mL/min; λ = 210 nm; $t_{(R)\text{-}\mathbf{3d}}$ = 26.4 min, $t_{(S)\text{-}\mathbf{3d}}$ = 32.0 min).

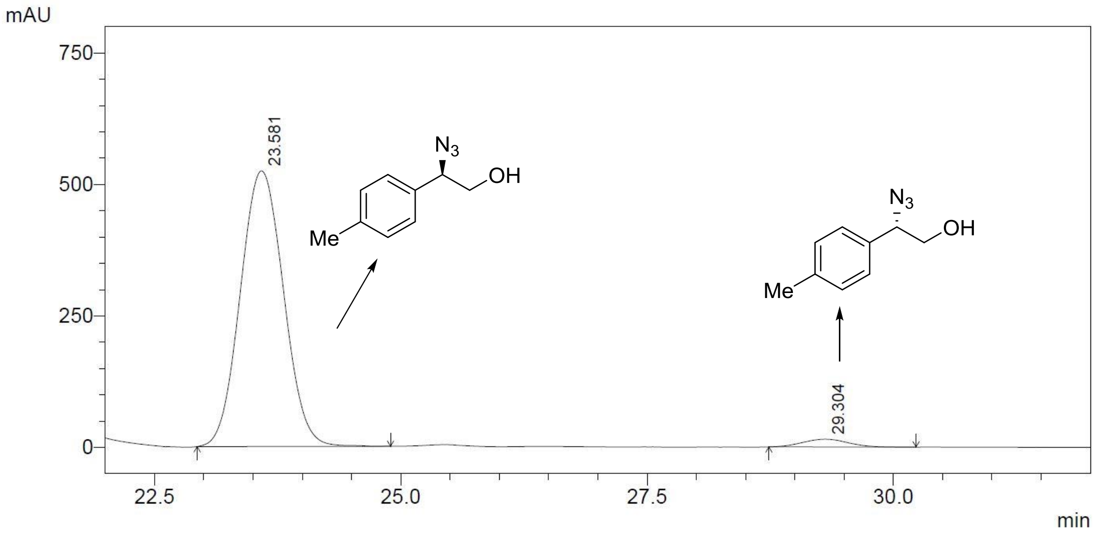

PDA

ID#	Rt. Time	Area	Height	Area %
1	23.581	15963979	523827	97.168
2	29.304	465331	14745	2.832

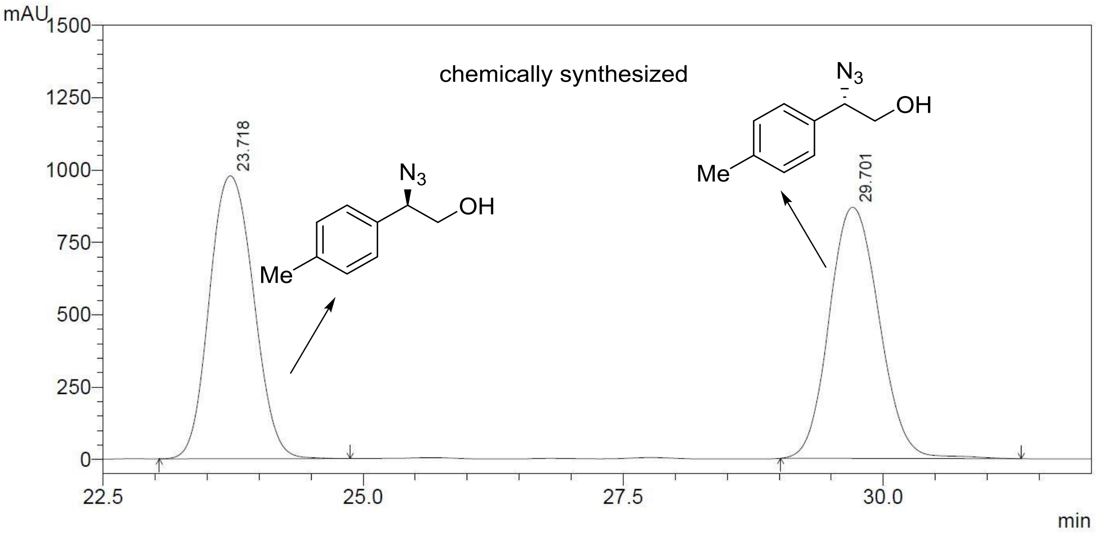

PDA

ID#	Rt. Time	Area	Height	Area %
1	23.718	28854511	976811	49.897
2	29.701	28974119	867646	50.103

Chiral HPLC analysis: Chiralcel AD-H (Hexane/*i*-PrOH = 95/5; 0.5 mL/min; λ = 210 nm; $t_{(R)\text{-}\mathbf{3e}}$ = 23.6 min, $t_{(S)\text{-}\mathbf{3e}}$ = 29.3 min).

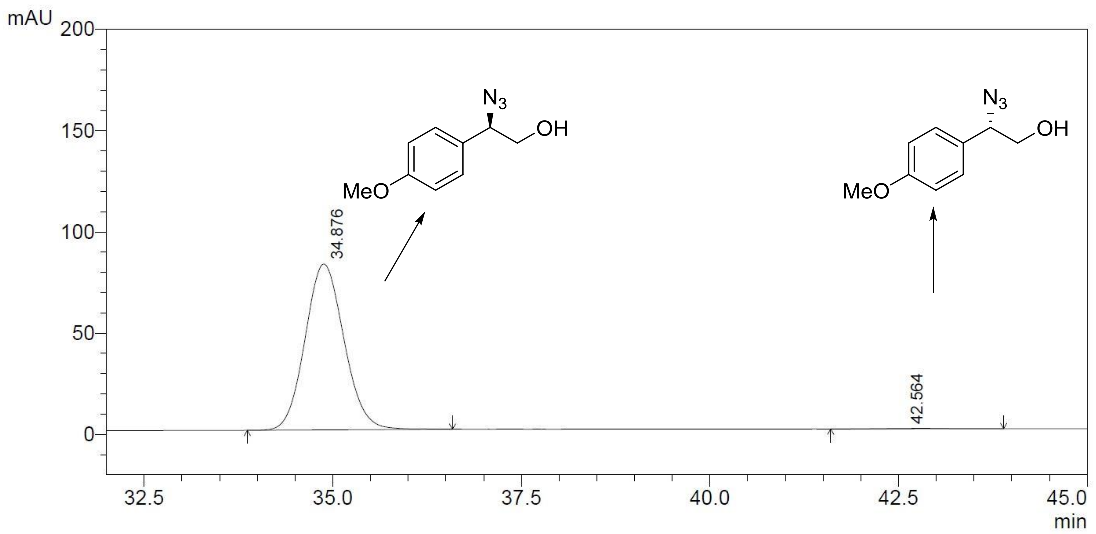

PDA

ID#	Rt. Time	Area	Height	Area %
1	34.876	2944702	81920	99.686
2	42.564	9284	281	0.314

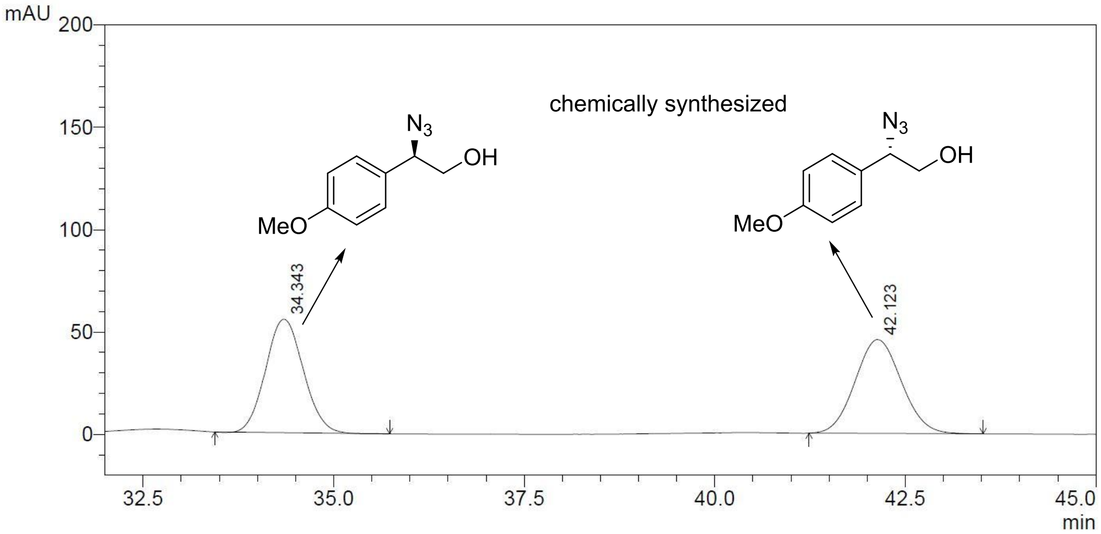

PDA

ID#	Rt. Time	Area	Height	Area %
1	34.343	1909849	55499	49.655
2	42.123	1936425	45826	50.345

Chiral HPLC analysis: Chiralcel AD-H (Hexane/*i*-PrOH = 95/5; 0.5 mL/min; λ = 210 nm; $t_{(R)\text{-}\mathbf{3f}}$ = 34.9 min, $t_{(S)\text{-}\mathbf{3f}}$ = 42.6 min).

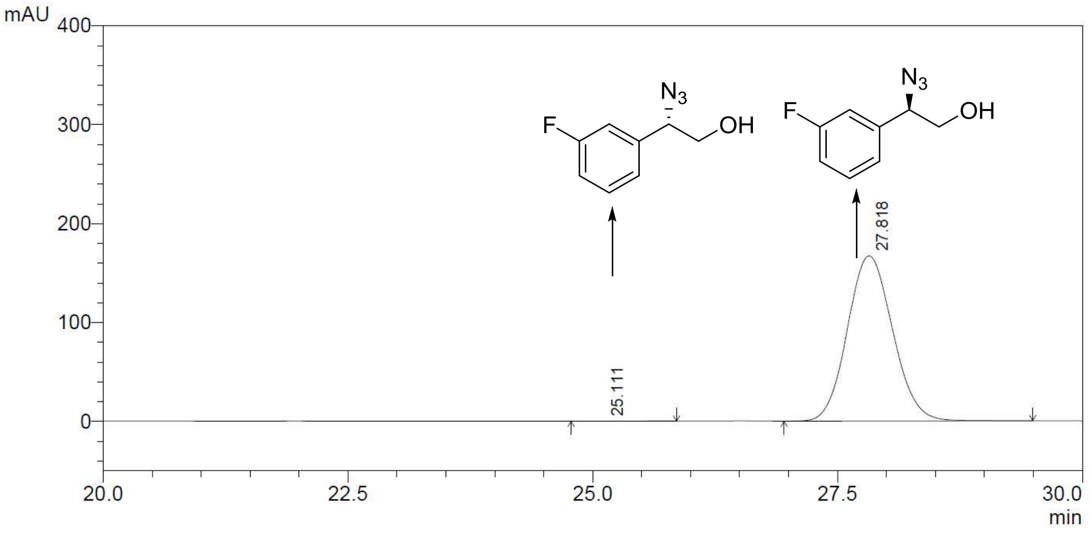

PDA

ID#	Rt. Time	Area	Height	Area %
1	25.111	1986	86	0.037
2	27.818	5363787	166927	99.963

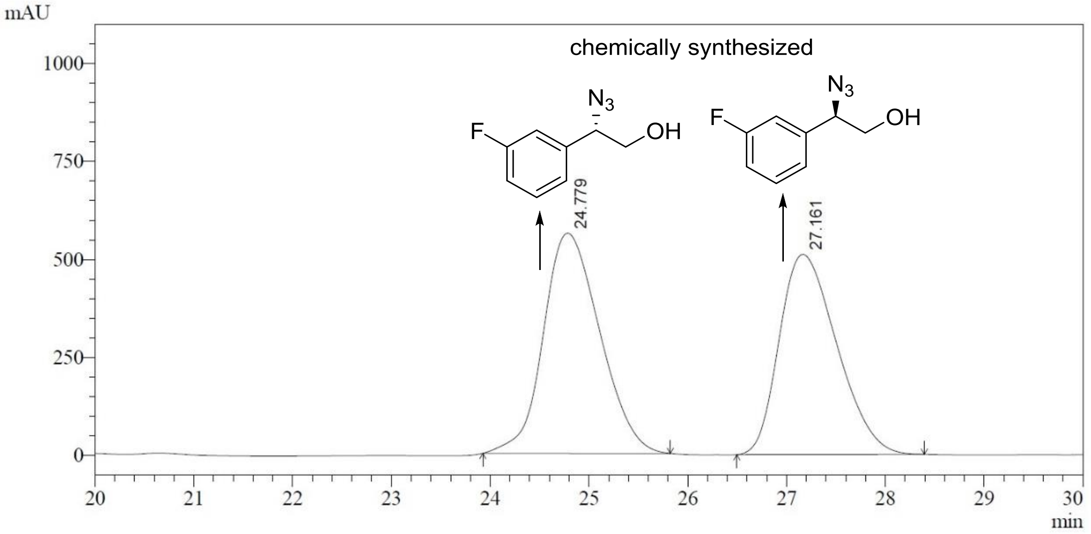

PDA

ID#	Rt. Time	Area	Height	Area %
1	24.779	22339825	561359	51.958
2	27.161	20655975	510417	48.042

Chiral HPLC analysis: Chiralcel AS-3 (Hexane/*i*-PrOH = 95/5; 0.5 mL/min; λ = 210 nm; $t_{(R)\text{-}\mathbf{3g}}$ = 27.8 min, $t_{(S)\text{-}\mathbf{3g}}$ = 25.1 min).

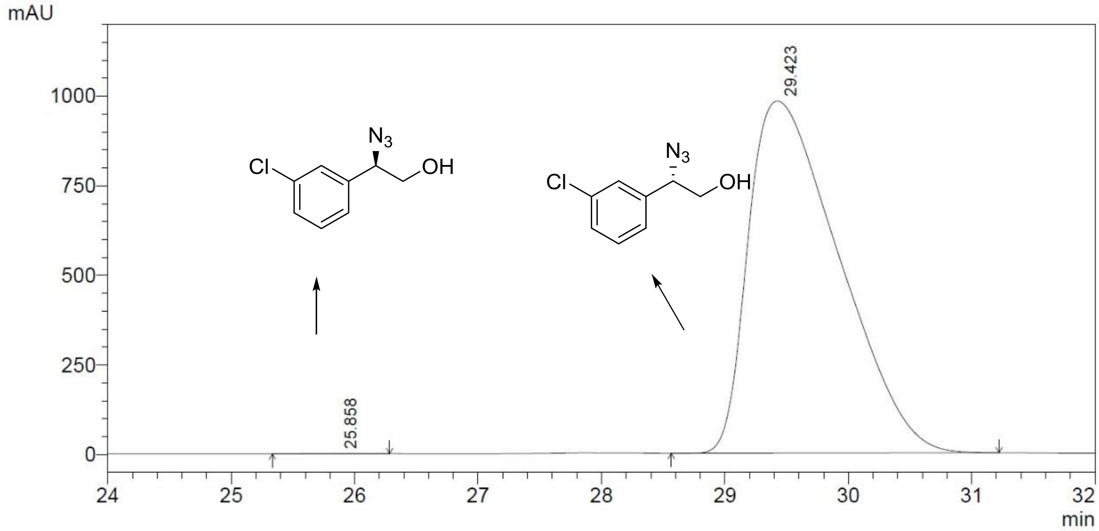

PDA

ID#	Rt. Time	Area	Height	Area %
1	25.858	19654	718	0.039
2	29.423	50251854	982038	99.961

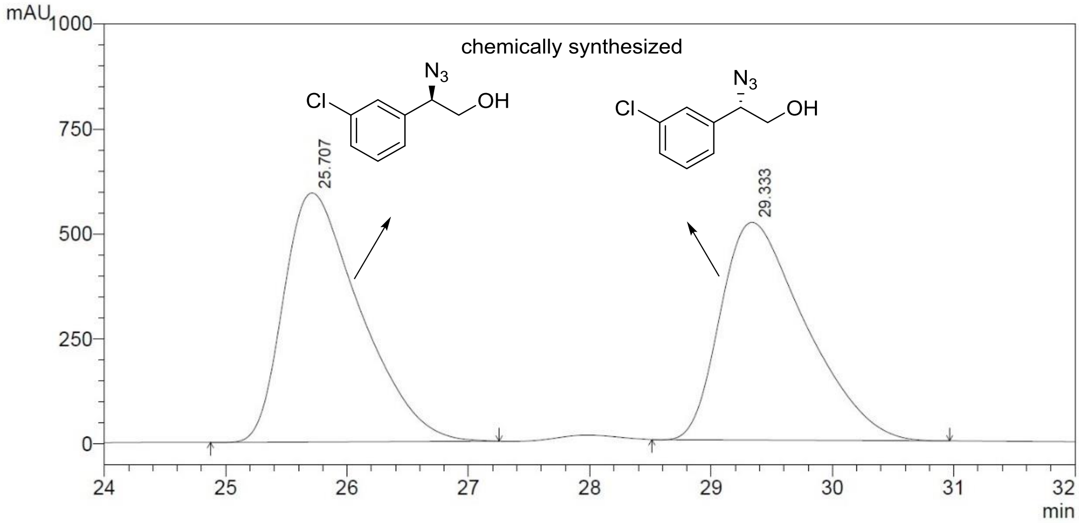

PDA

ID#	Rt. Time	Area	Height	Area %
1	25.707	26205003	592821	50.811
2	29.333	25368356	518299	49.189

Chiral HPLC analysis: Chiralcel AS-3 (Hexane/*i*-PrOH = 95/5; 0.5 mL/min; λ = 210 nm; $t_{(R)\text{-}\mathbf{3h}}$ = 29.4 min, $t_{(S)\text{-}\mathbf{3h}}$ = 25.9 min).

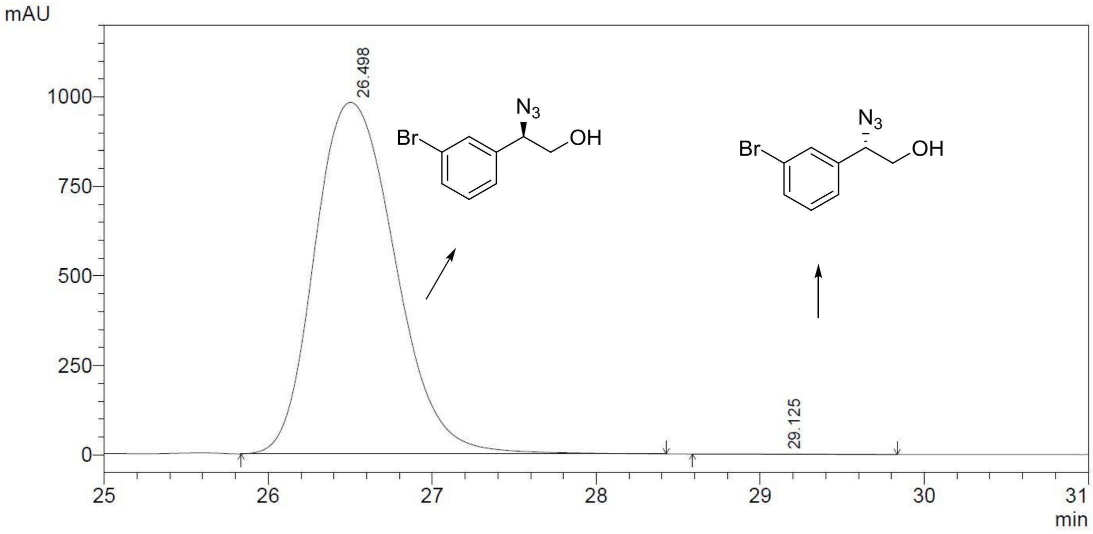

PDA

ID#	Rt. Time	Area	Height	Area %
1	26.498	33536539	981911	99.960
2	29.125	13368	588	0.040

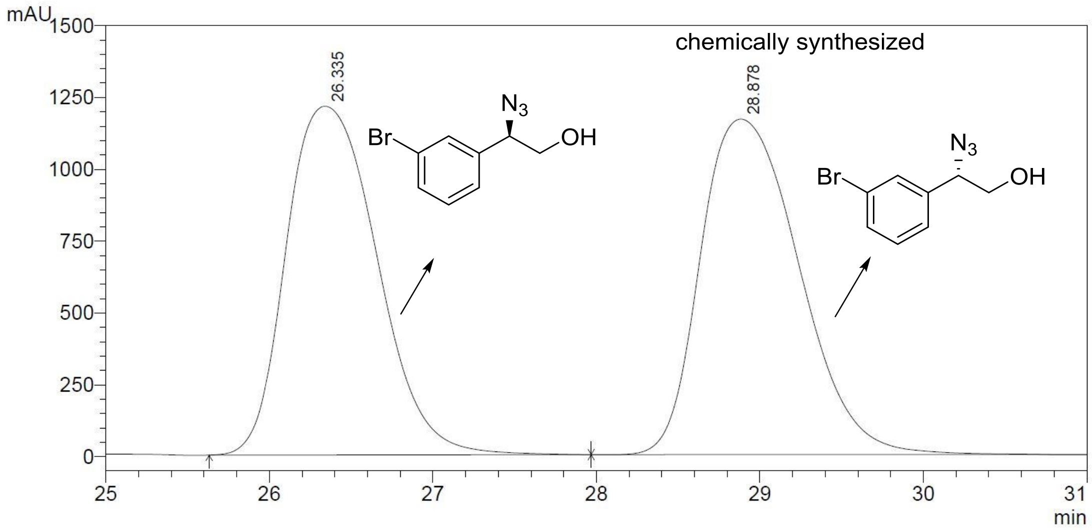

PDA

ID#	Rt. Time	Area	Height	Area %
1	26.335	46936487	1213652	48.953
2	28.878	48944816	1167628	51.047

Chiral HPLC analysis: Chiralcel OD-H (Hexane/*i*-PrOH = 95/5; 0.5 mL/min; λ = 210 nm; $t_{(R)\text{-}\mathbf{3i}}$ = 26.5 min, $t_{(S)\text{-}\mathbf{3i}}$ = 29.1 min).

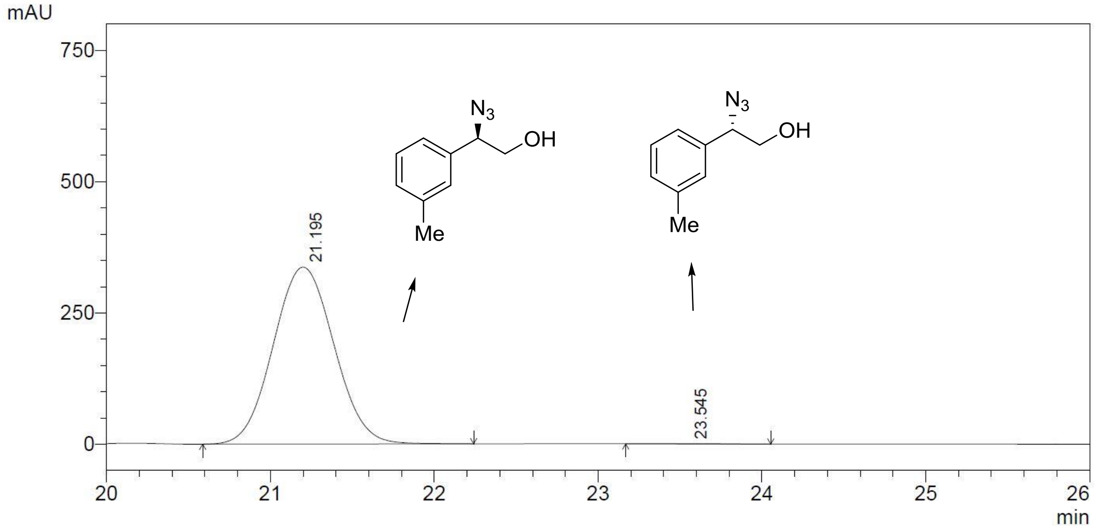

PDA

ID#	Rt. Time	Area	Height	Area %
1	21.195	8694778	336570	99.730
2	23.545	23522	919	0.270

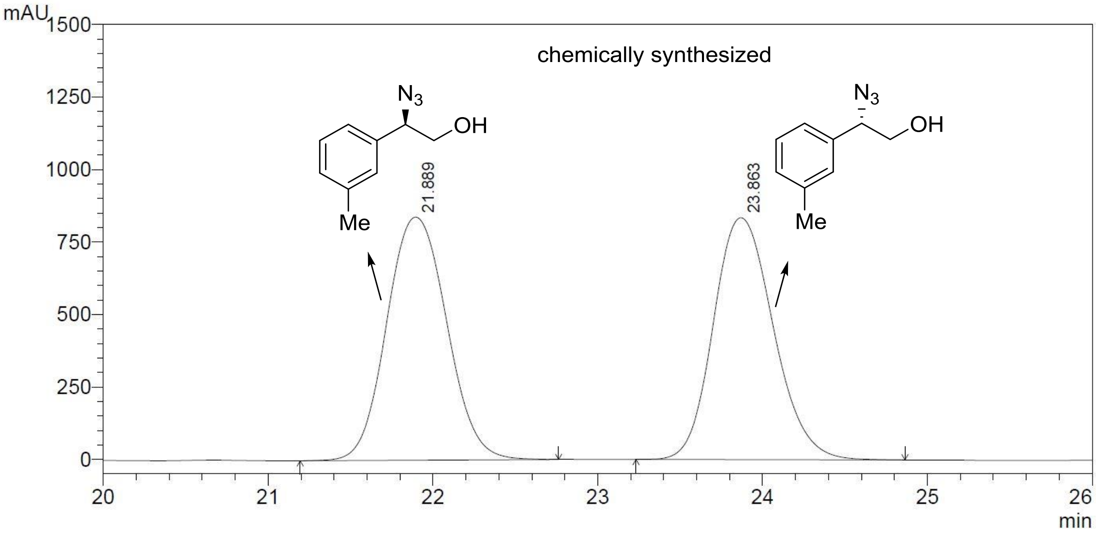

PDA

ID#	Rt. Time	Area	Height	Area %
1	21.889	21226699	837801	50.114
2	23.863	21130388	834213	49.886

Chiral HPLC analysis: Chiralcel OJ-H (Hexane/*i*-PrOH = 95/5; 0.5 mL/min; λ = 210 nm; $t_{(R)\text{-}\mathbf{3j}}$ = 21.2 min, $t_{(S)\text{-}\mathbf{3j}}$ = 23.5 min).

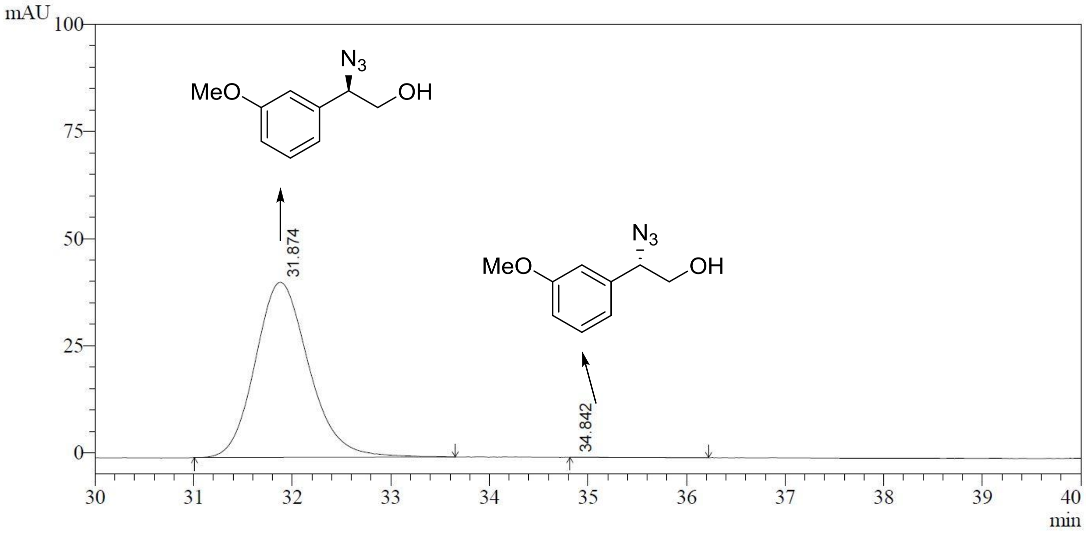

PDA

ID#	Rt. Time	Area	Height	Area %
1	31.874	1543755	40840	99.970
2	34.842	456	35	0.030

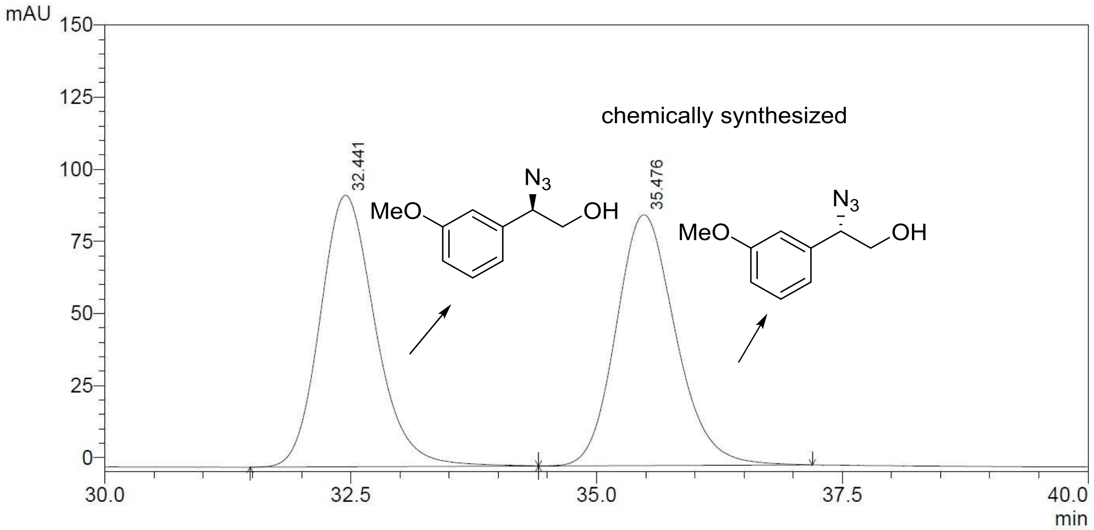

PDA

ID#	Rt. Time	Area	Height	Area %
1	32.441	3684361	94183	50.105
2	35.476	3668960	87054	49.895

Chiral HPLC analysis: Chiralcel OD-H (Hexane/*i*-PrOH = 95/5; 0.5 mL/min; λ = 210 nm; $t_{(R)\text{-}\mathbf{3k}}$ = 31.9 min, $t_{(S)\text{-}\mathbf{3k}}$ = 34.8 min).

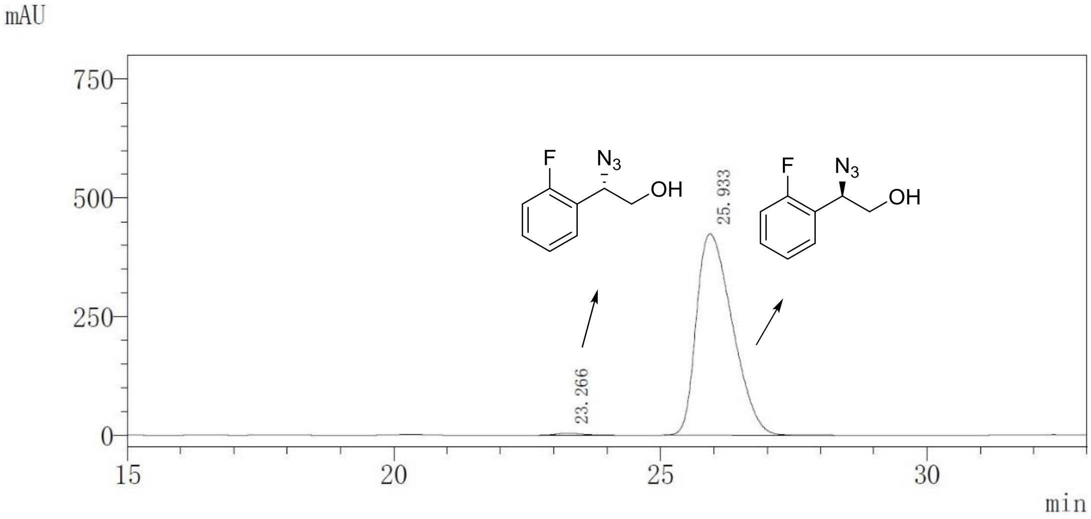

PDA

ID#	Rt. Time	Area	Height	Area%
1	23.266	155156	4411	0.773
2	25.933	19922843	423919	99.227

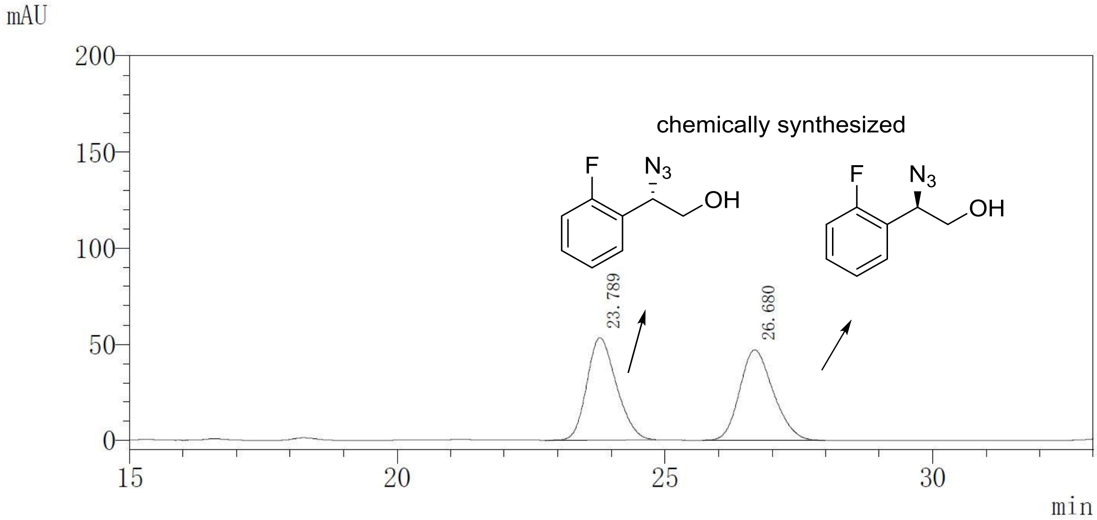

PDA

ID#	Rt. Time	Area	Height	Area%
1	23.789	2034845	53156	49.832
2	26.680	2048550	47067	50.168

Chiral HPLC analysis: Chiralcel AS-3 (Hexane/*i*-PrOH = 95/5; 0.5 mL/min; λ = 210 nm; $t_{(R)\text{-}\mathbf{3l}}$ = 25.9 min, $t_{(S)\text{-}\mathbf{3l}}$ = 23.3 min).

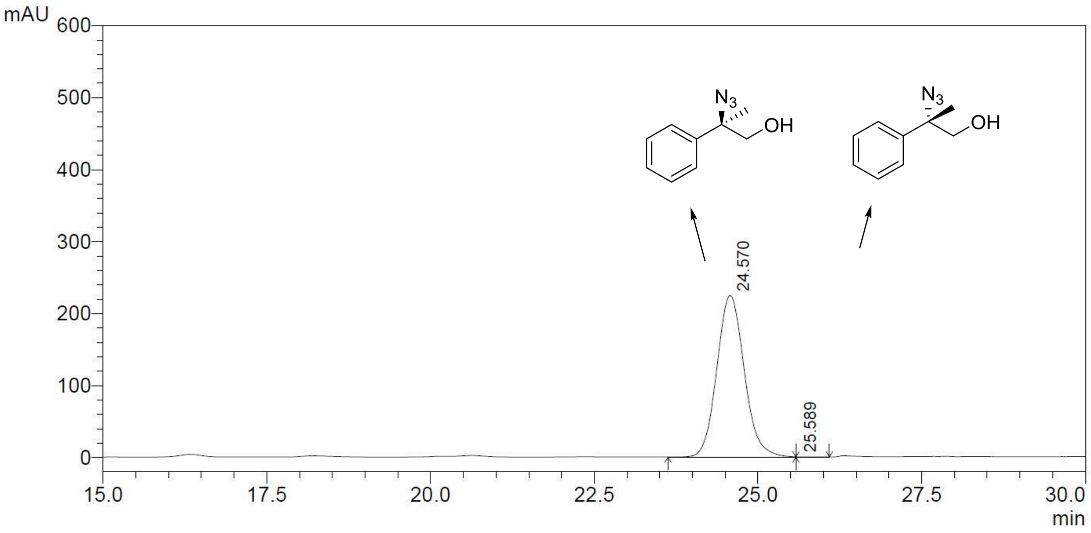

PDA

ID#	Rt. Time	Area	Height	Area %
1	24.570	6551498	224620	99.951
2	25.589	3201	326	0.049

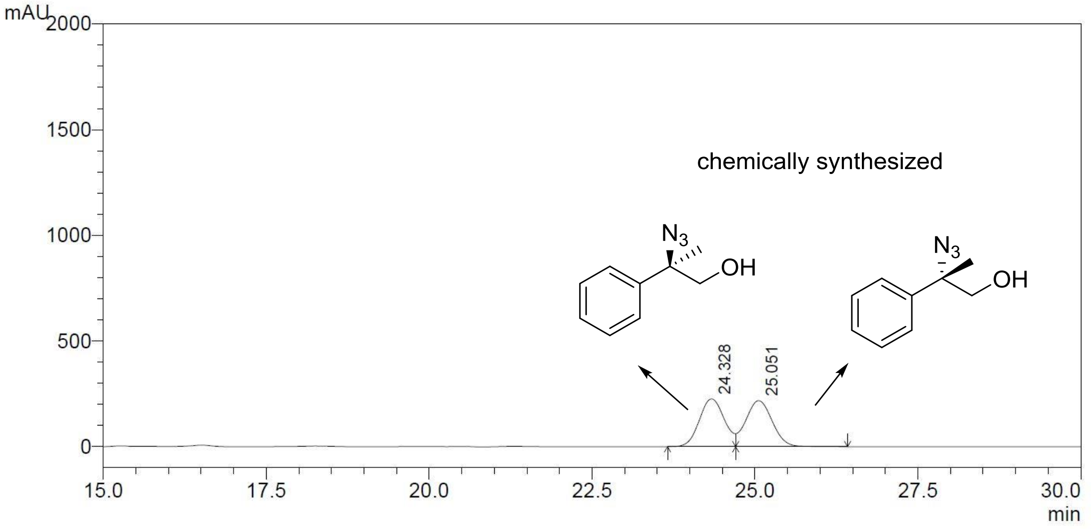

PDA

ID#	Rt. Time	Area	Height	Area %
1	24.328	6052991	226022	49.802
2	25.051	6101225	217539	50.198

Chiral HPLC analysis: Chiralcel AD-H (Hexane/*i*-PrOH = 95/5; 0.5 mL/min; λ = 210 nm; $t_{(R)\text{-}\mathbf{3m}}$ = 24.6 min, $t_{(S)\text{-}\mathbf{3m}}$ = 25.6 min).

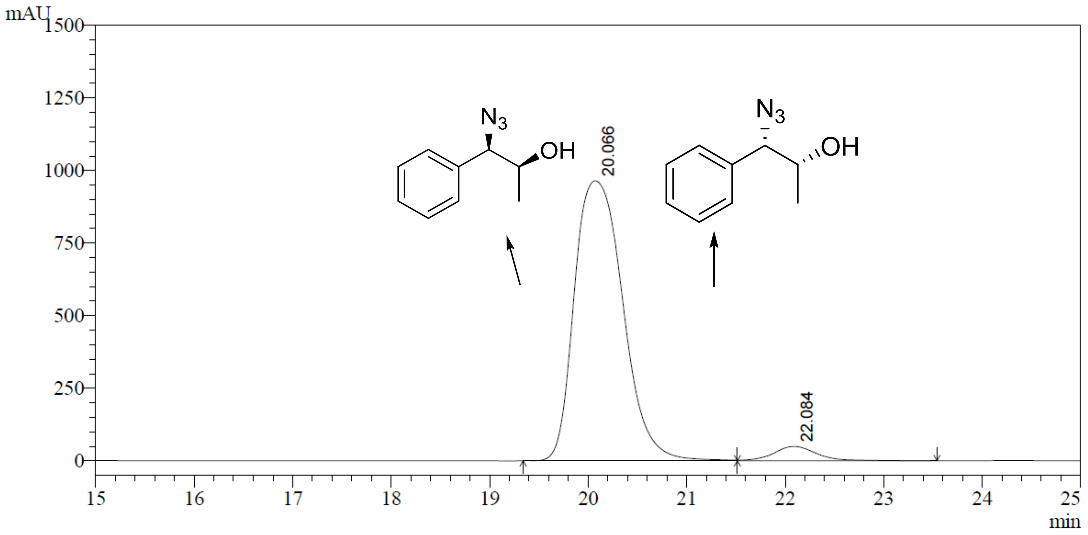

PDA

ID#	Rt. Time	Area	Height	Area %
1	20.066	33062612	963969	95.637
2	22.084	1508194	49313	4.363

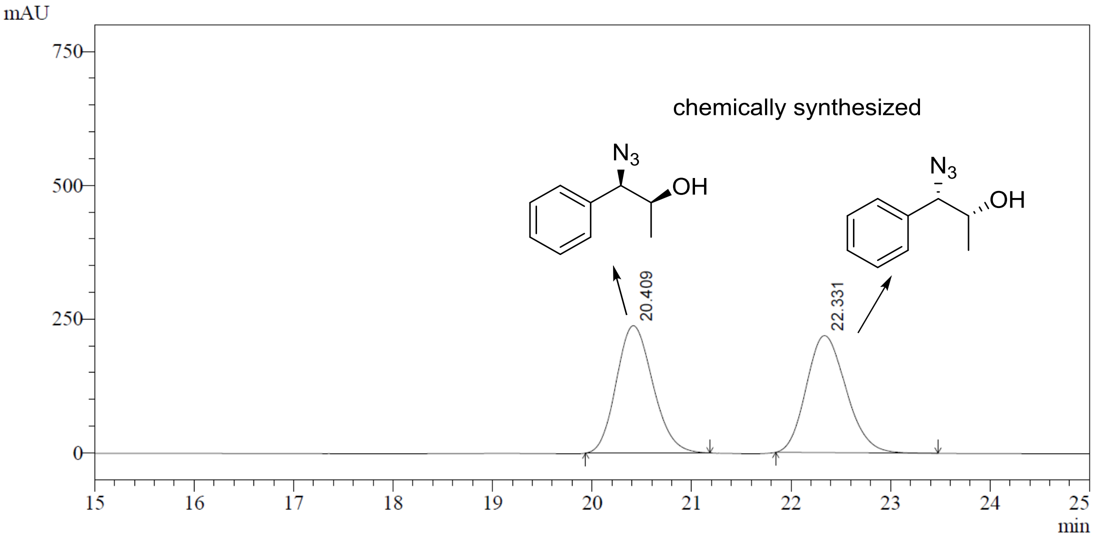

PDA

ID#	Rt. Time	Area	Height	Area %
1	20.409	6080332	238068	49.998
2	22.331	6080921	218747	50.002

Chiral HPLC analysis: Chiralcel OD-H (Hexane/*i*-PrOH = 95/5; 0.5 mL/min; λ = 210 nm; $t_{(1R,2S)\text{-}\mathbf{3n}}$ = 20.1 min, $t_{(1S,2R)\text{-}\mathbf{3n}}$ = 22.1 min).

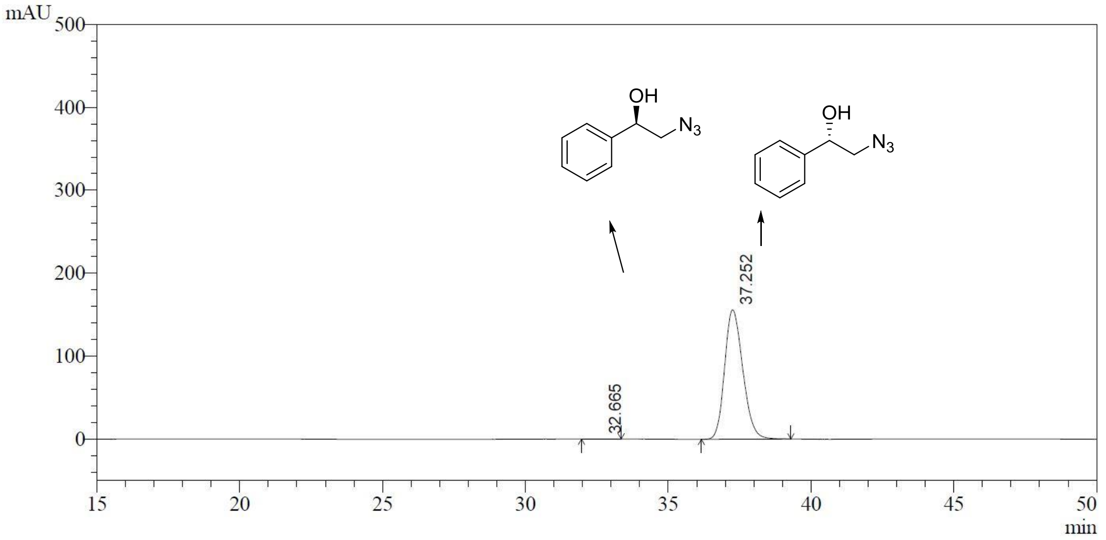

PDA

ID#	Rt. Time	Area	Height	Area %
1	32.665	3952	104	0.057
2	37.252	6922689	156090	99.943

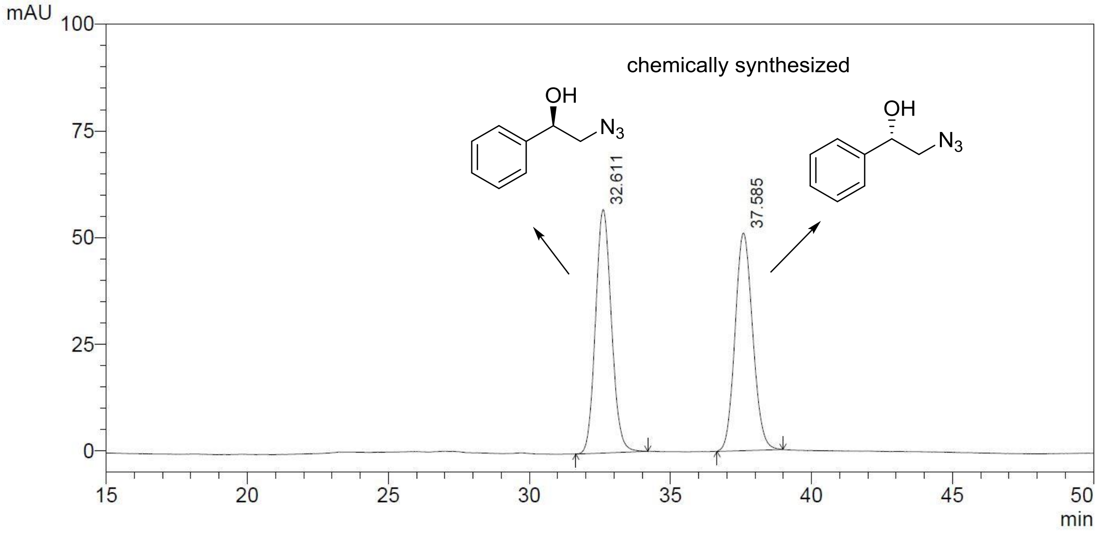

PDA

ID#	Rt. Time	Area	Height	Area %
1	32.611	2238798	57031	50.207
2	37.585	2220312	50981	49.793

Chiral HPLC analysis: Chiralcel OD-H (Hexane/*i*-PrOH = 95/5; 0.5 mL/min; λ = 210 nm; $t_{(S)\textbf{-4a}}$ = 37.3 min, $t_{(R)\textbf{-4a}}$ = 32.7 min).

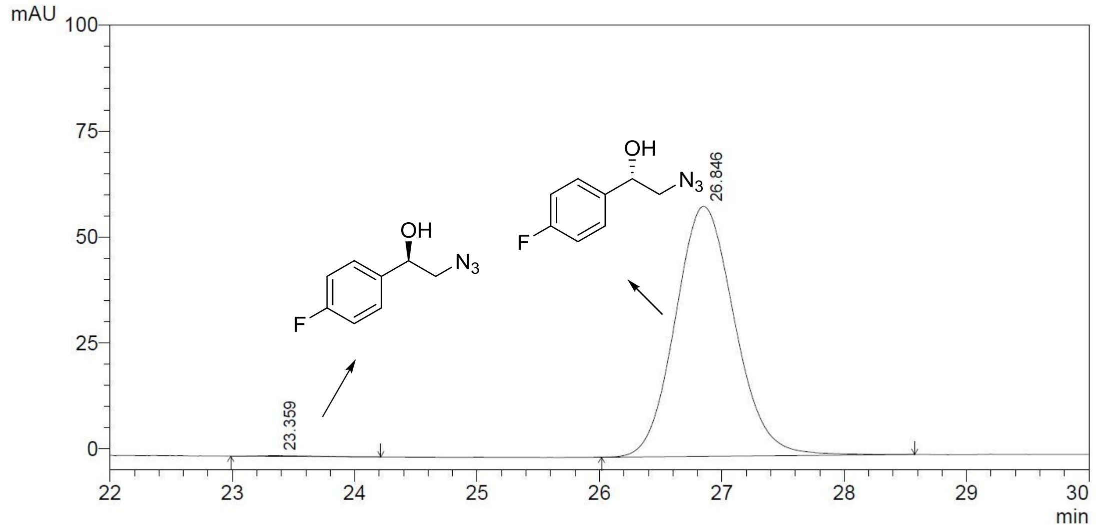

PDA

ID#	Rt. Time	Area	Height	Area %
1	23.359	4452	205	0.223
2	26.846	1994822	58996	99.777

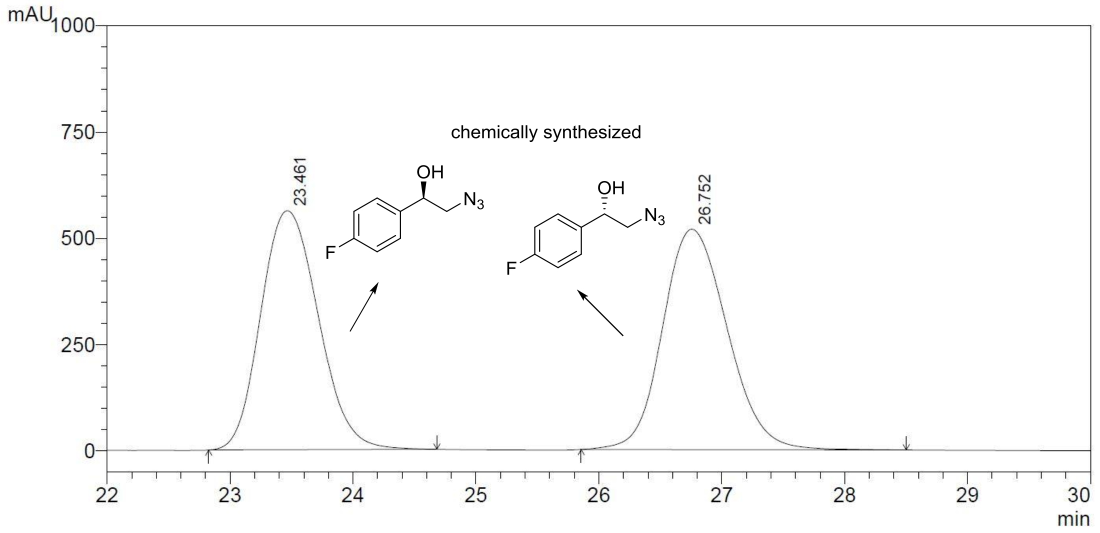

PDA

ID#	Rt. Time	Area	Height	Area %
1	23.461	18340719	562074	49.196
2	26.752	18940462	517961	50.804

Chiral HPLC analysis: Chiralcel OD-H (Hexane/*i*-PrOH = 95/5; 0.5 mL/min; λ = 210 nm; $t_{(S)\text{-}\mathbf{4b}}$ = 26.8 min, $t_{(R)\text{-}\mathbf{4b}}$ = 23.4 min).

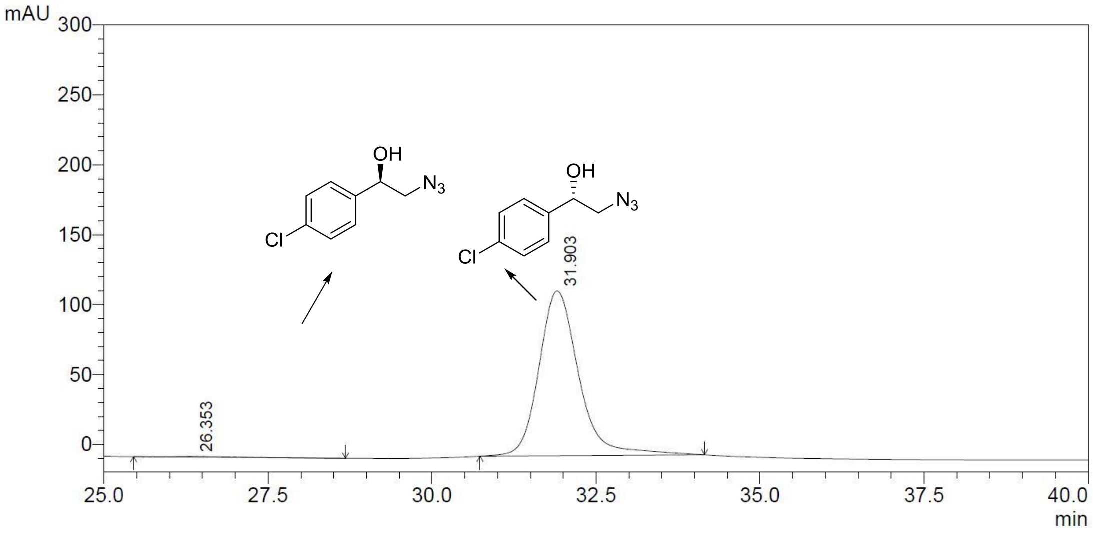

PDA

ID#	Rt. Time	Area	Height	Area %
1	26.353	25176	533	0.502
2	31.903	4987161	117828	99.498

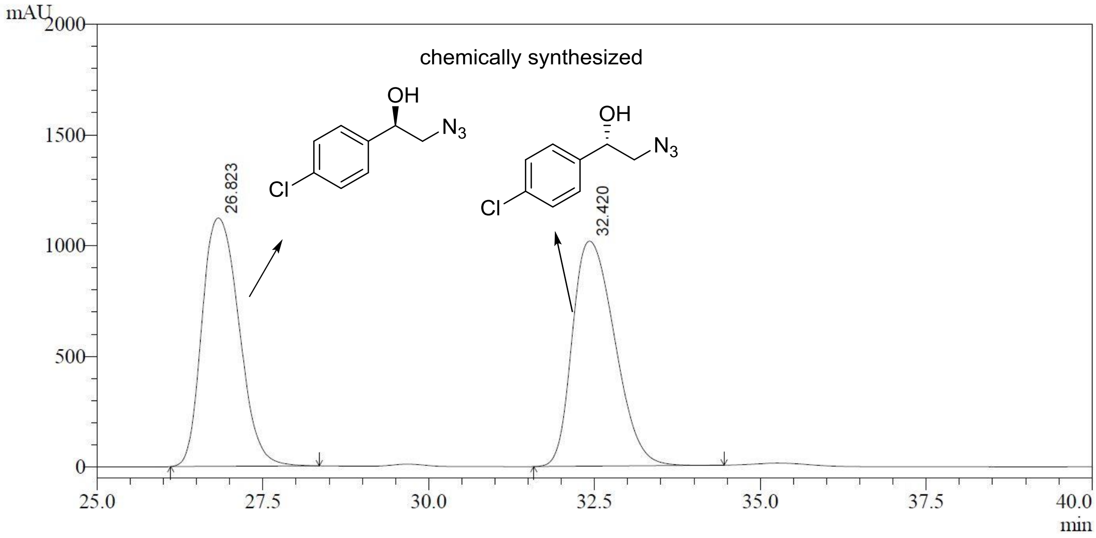

PDA

ID#	Rt. Time	Area	Height	Area %
1	26.823	43578925	1120567	49.121
2	32.420	45138197	1015513	50.879

Chiral HPLC analysis: Chiralcel OD-H (Hexane/*i*-PrOH = 95/5; 0.5 mL/min; λ = 210 nm; $t_{(S)\text{-}\mathbf{4c}}$ = 31.9 min, $t_{(R)\text{-}\mathbf{4c}}$ = 26.4 min).

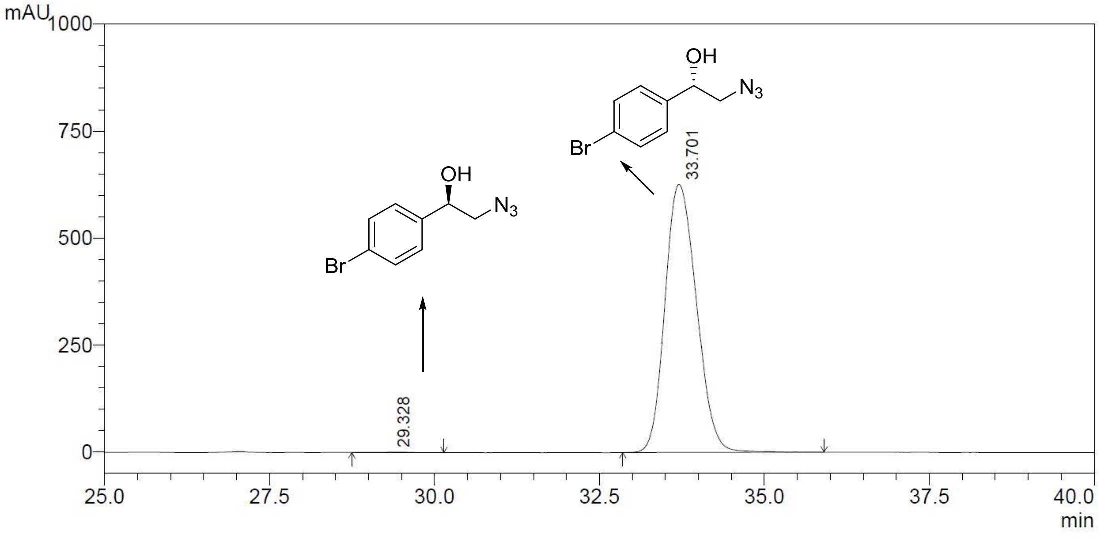

PDA

ID#	Rt. Time	Area	Height	Area %
1	29.328	35645	1140	0.171
2	33.701	20839350	626013	99.829

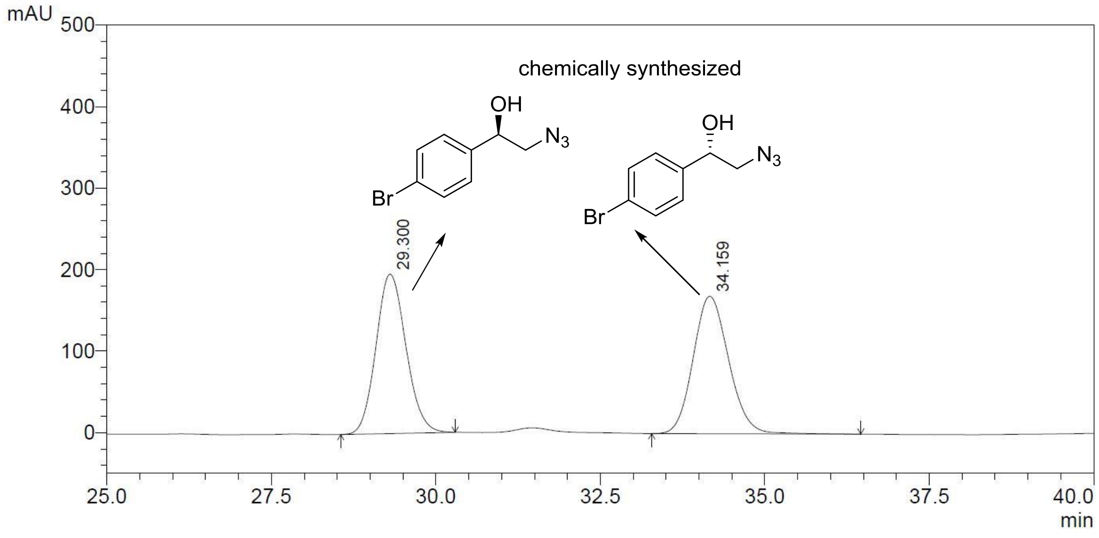

PDA

ID#	Rt. Time	Area	Height	Area %
1	29.300	6205704	195525	49.547
2	34.159	6319107	168470	50.453

Chiral HPLC analysis: Chiralcel OD-H (Hexane/*i*-PrOH = 95/5; 0.5 mL/min; λ = 210 nm; $t_{(S)\text{-}\mathbf{4d}}$ = 33.7 min, $t_{(R)\text{-}\mathbf{4d}}$ = 29.3 min).

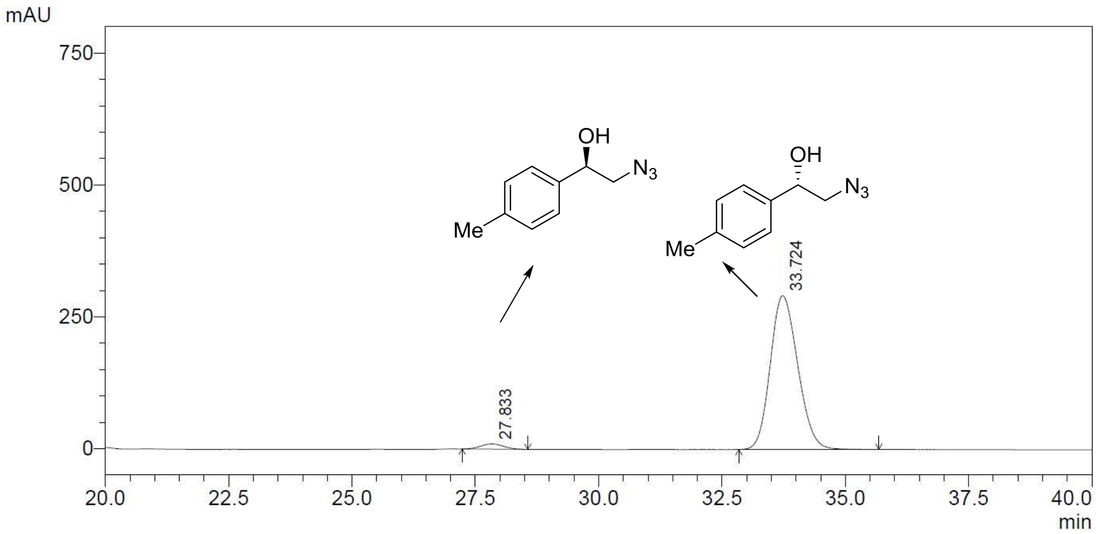

PDA

ID#	Rt. Time	Area	Height	Area %
1	27.833	315834	9862	2.720
2	33.724	11294676	291404	97.280

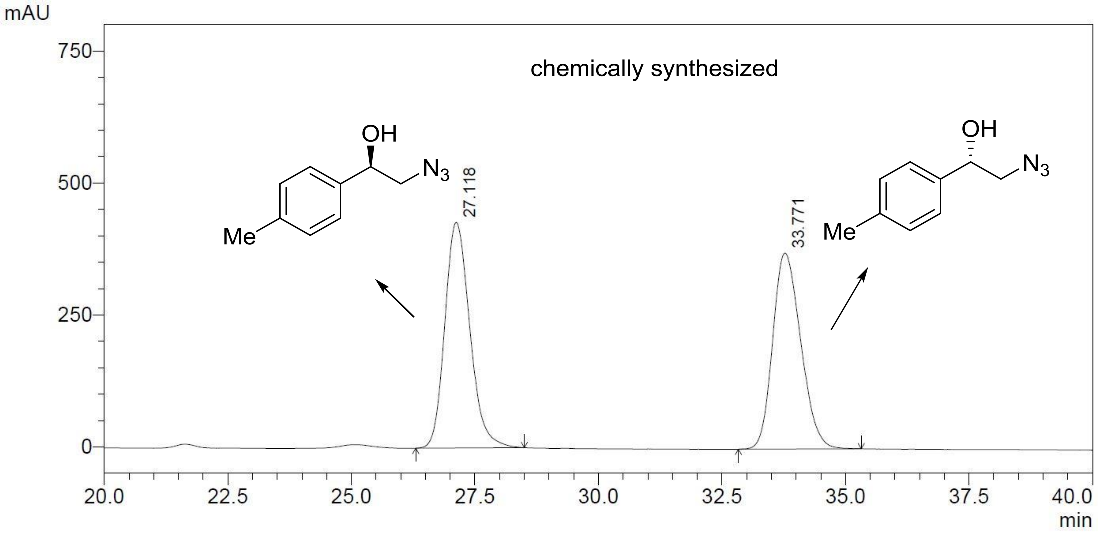

PDA

ID#	Rt. Time	Area	Height	Area %
1	27.118	15062790	427277	50.704
2	33.771	14644339	370896	49.296

Chiral HPLC analysis: Chiralcel OD-H (Hexane/*i*-PrOH = 95/5; 0.5 mL/min; λ = 210 nm; $t_{(S)\text{-}\mathbf{4e}}$ = 33.7 min, $t_{(R)\text{-}\mathbf{4e}}$ = 27.8 min).

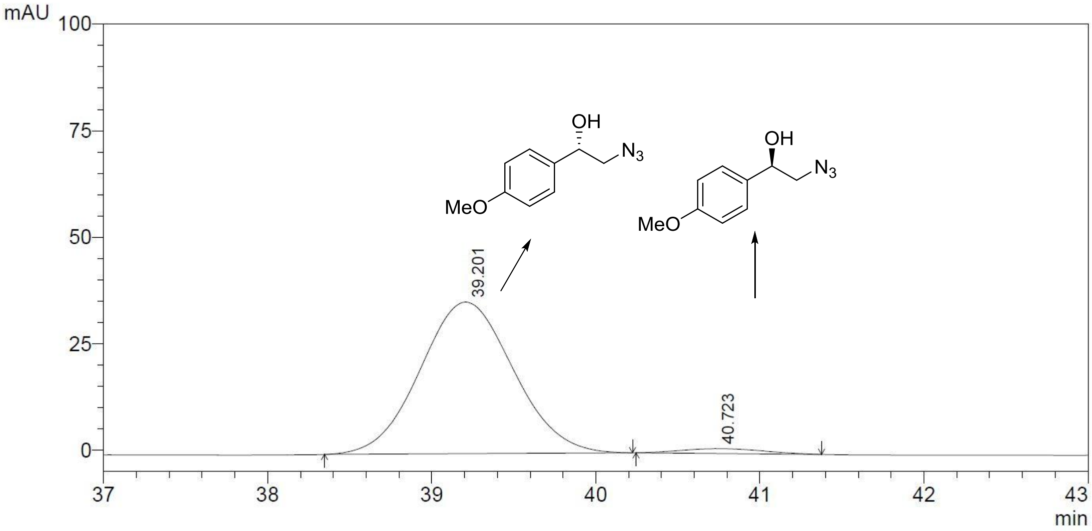

PDA

ID#	Rt. Time	Area	Height	Area %
1	39.201	1406086	35542	97.281
2	40.723	39307	1139	2.719

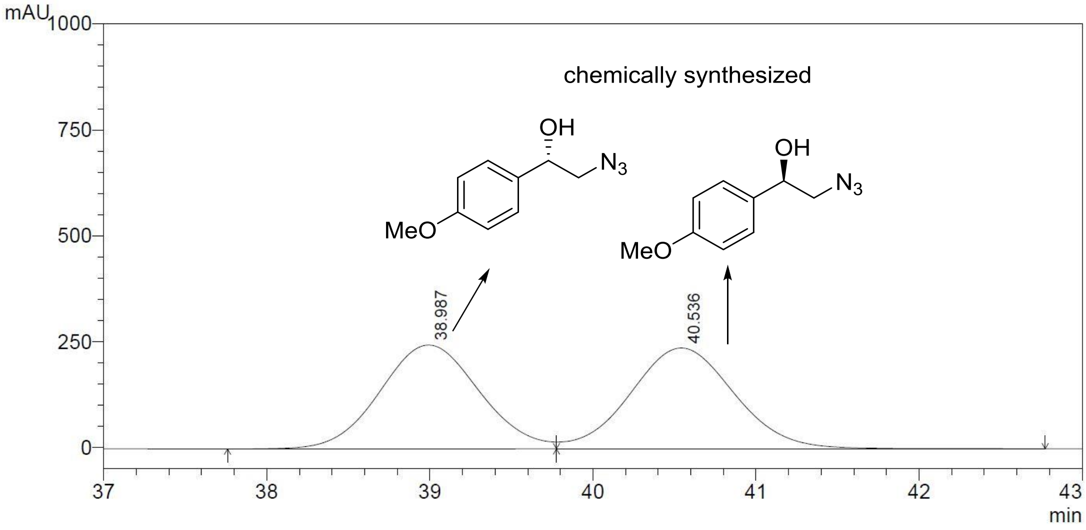

PDA

ID#	Rt. Time	Area	Height	Area %
1	38.987	10427612	245116	49.593
2	40.536	10598966	237900	50.407

Chiral HPLC analysis: Chiralcel AD-H (Hexane/*i*-PrOH = 95/5; 0.5 mL/min; λ = 210 nm; $t_{(S)\text{-}\mathbf{4f}}$ = 39.2 min, $t_{(R)\text{-}\mathbf{4f}}$ = 40.7 min).

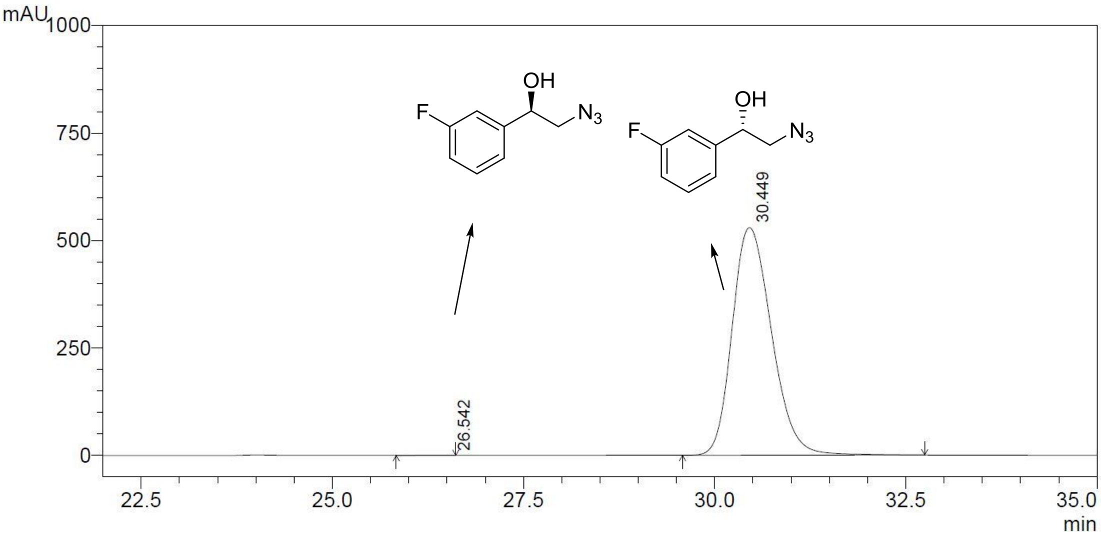

PDA

ID#	Rt. Time	Area	Height	Area %
1	26.542	2482	86	0.013
2	30.449	18977862	529663	99.987

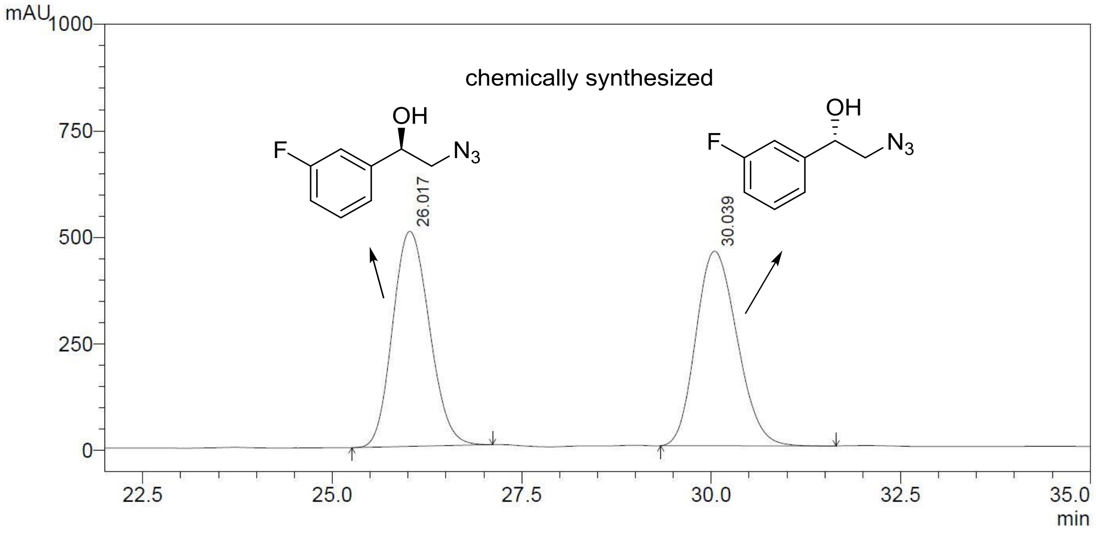

PDA

ID#	Rt. Time	Area	Height	Area %
1	26.017	17028263	504329	49.607
2	30.039	17297759	456433	50.393

Chiral HPLC analysis: Chiralcel OD-H (Hexane/*i*-PrOH = 95/5; 0.5 mL/min; λ = 210 nm; $t_{(S)\text{-}\mathbf{4g}}$ = 30.4 min, $t_{(R)\text{-}\mathbf{4g}}$ = 26.5 min).

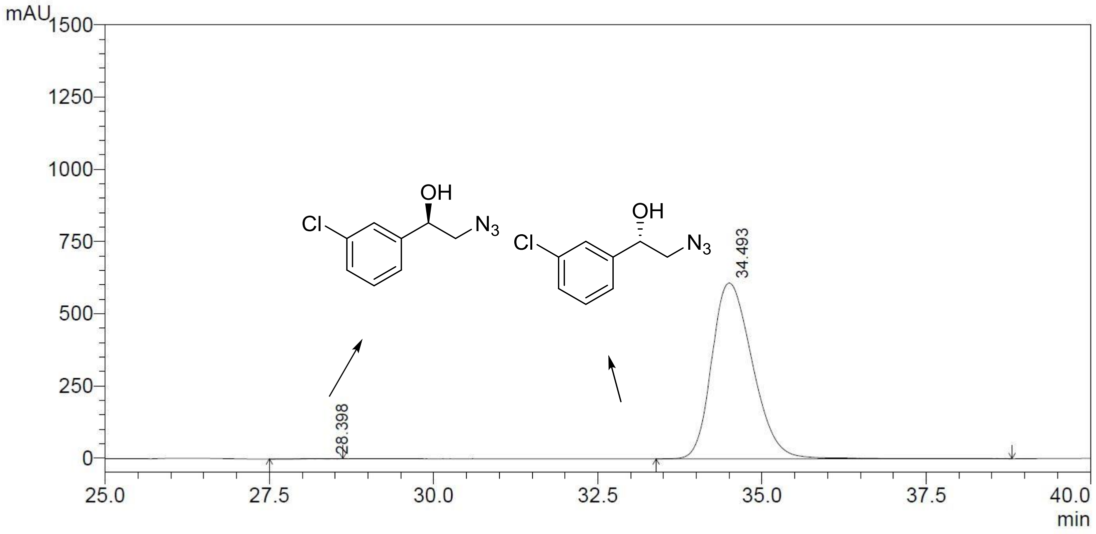

PDA

ID#	Rt. Time	Area	Height	Area %
1	28.398	1655	66	0.006
2	34.493	26222905	608649	99.994

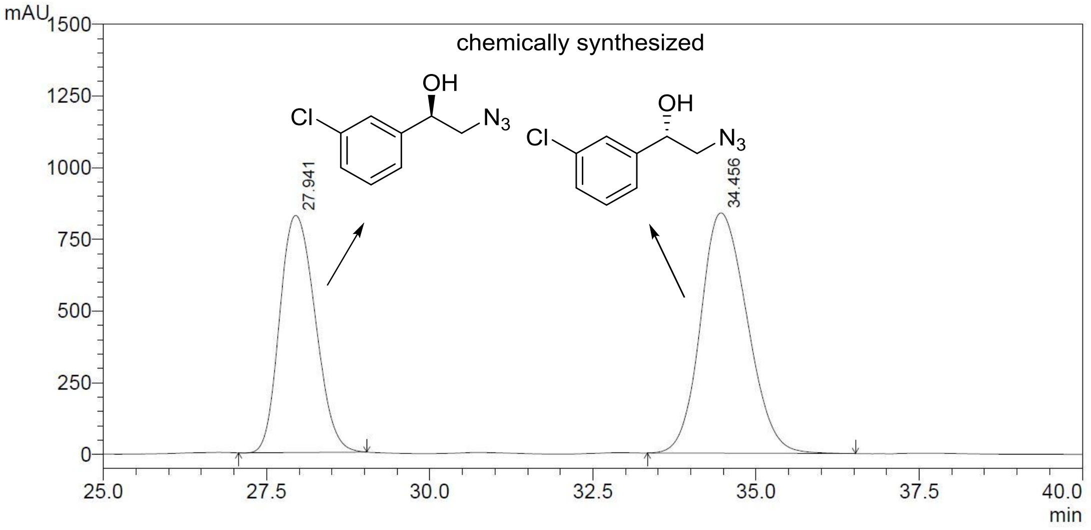

PDA

ID#	Rt. Time	Area	Height	Area %
1	27.941	31424919	826784	43.022
2	34.456	41618267	837678	56.978

Chiral HPLC analysis: Chiralcel OD-H (Hexane/*i*-PrOH = 95/5; 0.5 mL/min; λ = 210 nm; $t_{(S)\text{-}\mathbf{4h}}$ = 34.5 min, $t_{(R)\text{-}\mathbf{4h}}$ = 28.4 min).

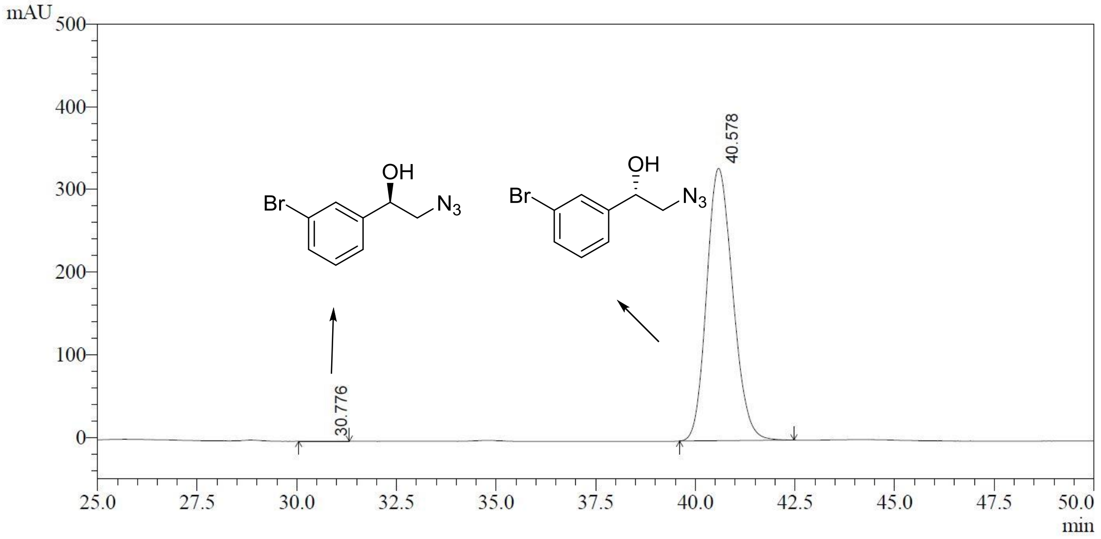

PDA

ID#	Rt. Time	Area	Height	Area %
1	30.776	1168	107	0.008
2	40.578	15065756	329260	99.992

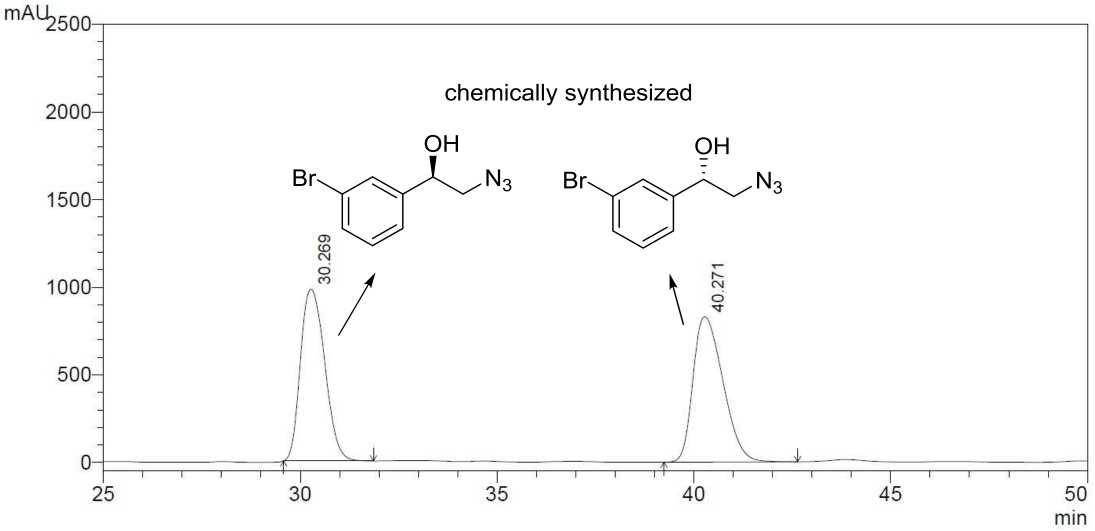

PDA

ID#	Rt. Time	Area	Height	Area %
1	30.269	41960154	979602	48.855
2	40.271	43927024	829501	51.145

Chiral HPLC analysis: Chiralcel OD-H (Hexane/*i*-PrOH = 95/5; 0.5 mL/min; λ = 210 nm; $t_{(S)\text{-}\mathbf{4i}}$ = 40.6 min, $t_{(R)\text{-}\mathbf{4i}}$ = 30.8 min).

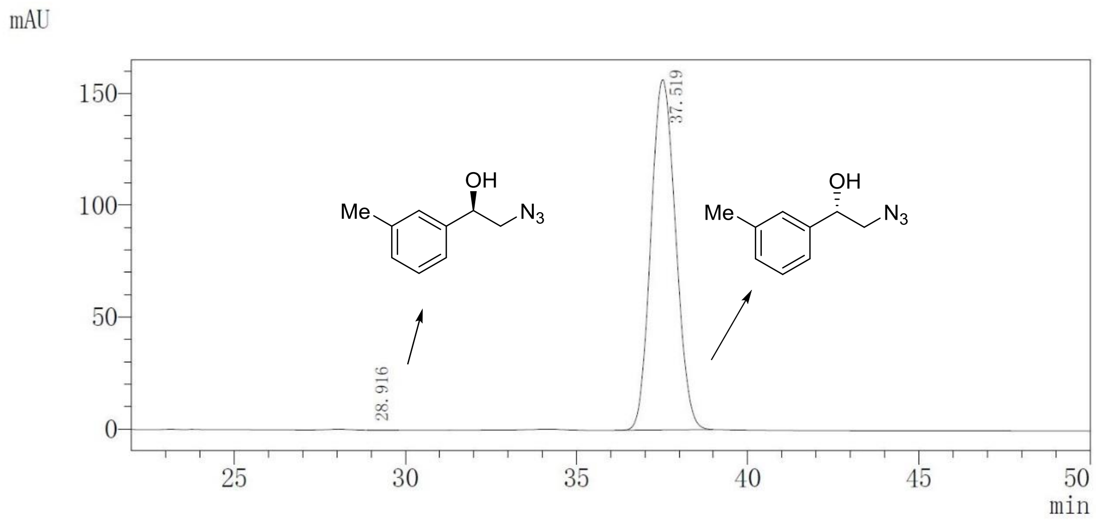

PDA

ID#	Rt. time	Area	Height	Area%
1	28.916	829	2	0.011
2	37.519	7881893	156624	99.989

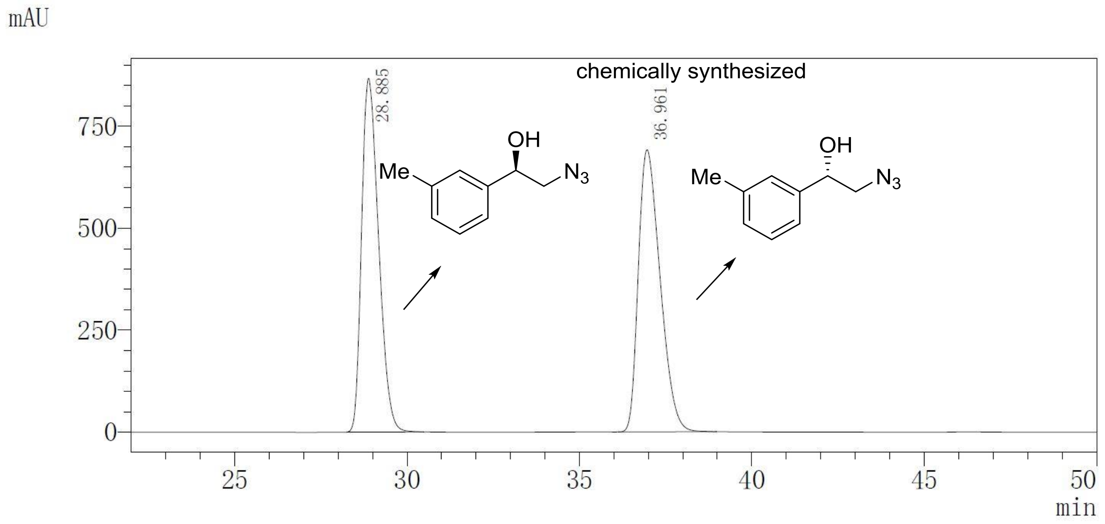

PDA

ID#	Rt. time	Area	Height	Area%
1	28.885	29663966	867177	49.385
2	36.961	30402241	691693	50.615

Chiral HPLC analysis: Chiralcel OJ-H (Hexane/*i*-PrOH = 95/5; 0.5 mL/min; λ = 210 nm; $t_{(S)\text{-}\mathbf{4j}}$ = 37.5 min, $t_{(R)\text{-}\mathbf{4j}}$ = 28.9 min).

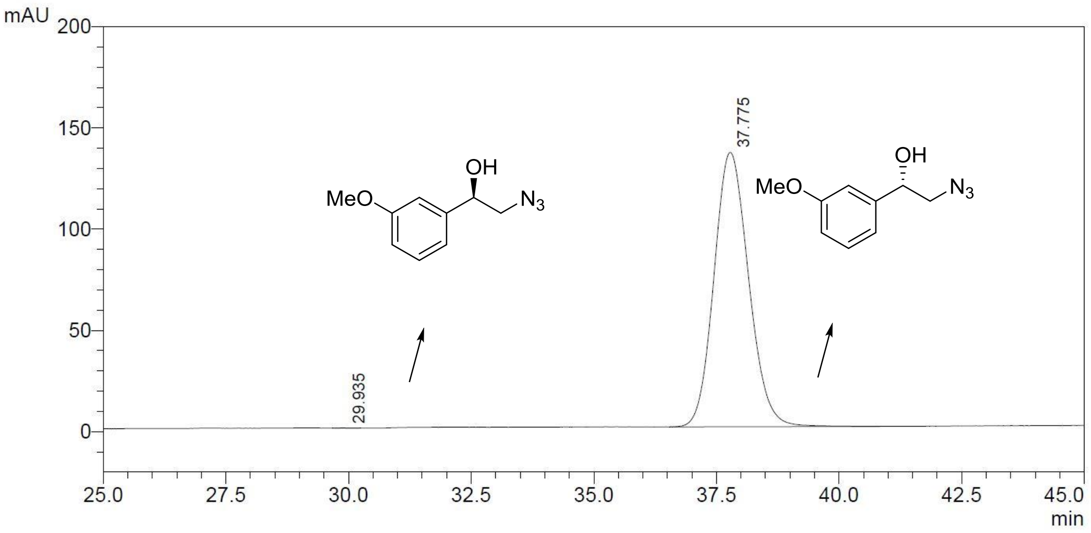

PDA

Peak#	Ret. Time	Area	Height	Area %
1	29.935	624	51	0.009
2	37.775	6662006	135489	99.991

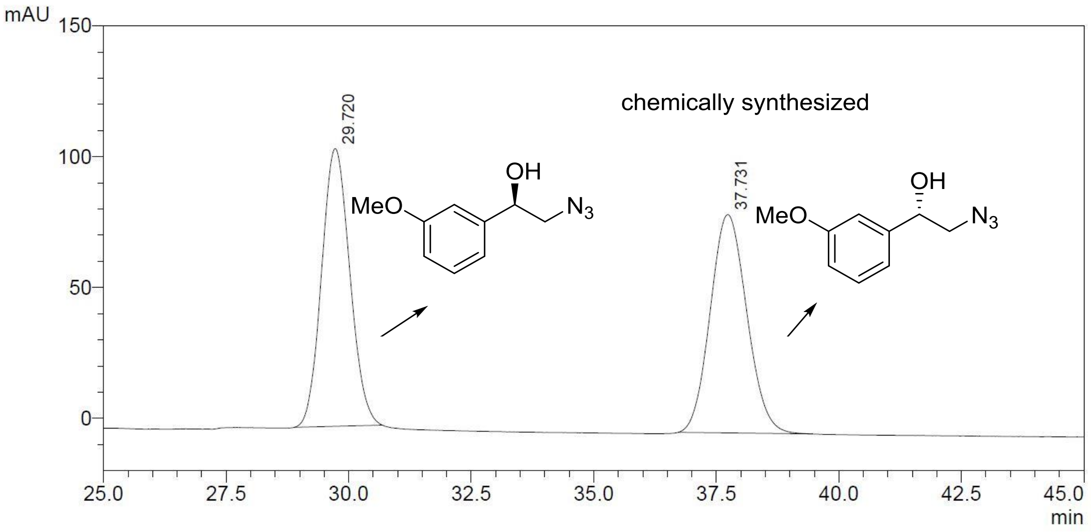

PDA

Peak#	Ret. Time	Area	Height	Area %
1	29.720	4250930	106097	49.536
2	37.731	4330521	83424	50.464

Chiral HPLC analysis: Chiralcel OD-H (Hexane/*i*-PrOH = 95/5; 0.5 mL/min; λ = 210 nm; $t_{(S)\text{-}\mathbf{4k}}$ = 37.8 min, $t_{(R)\text{-}\mathbf{4k}}$ = 29.9 min).

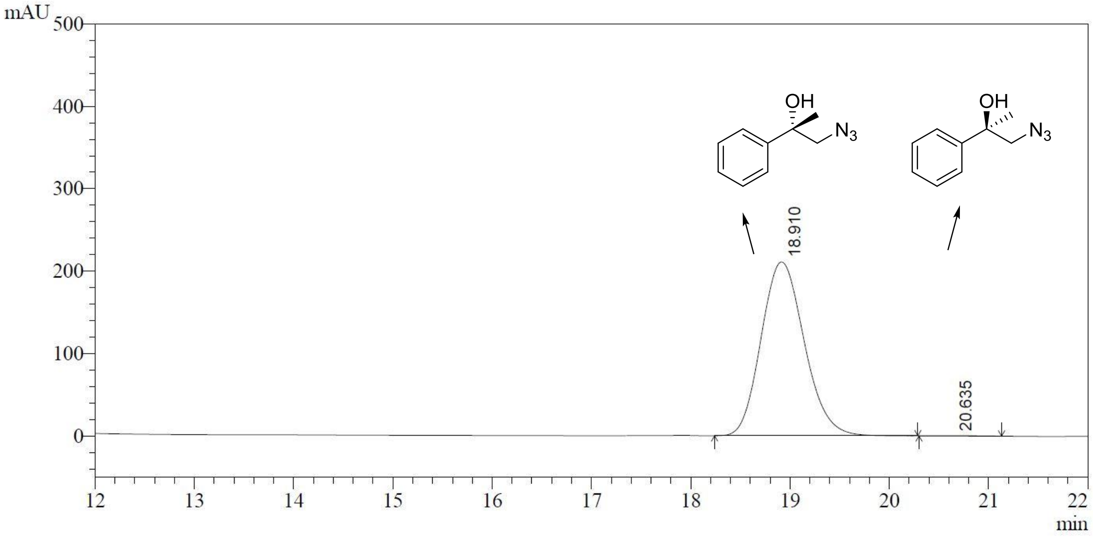

PDA

ID#	Rt. Time	Area	Height	Area %
1	18.910	6369500	210765	99.825
2	20.635	11145	568	0.175

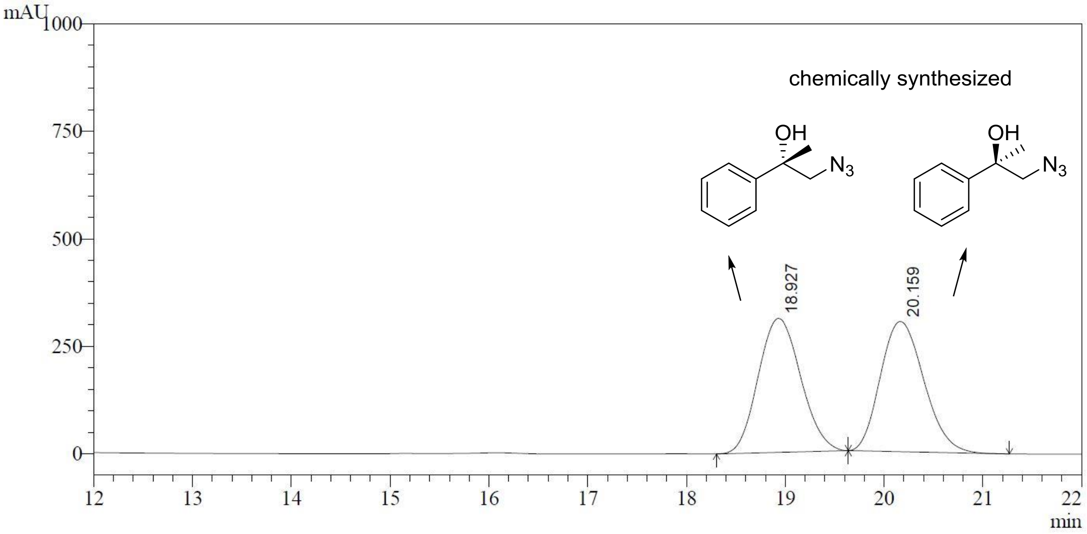

PDA

ID#	Rt. Time	Area	Height	Area %
1	18.927	9311872	311685	50.122
2	20.159	9266538	302668	49.878

Chiral HPLC analysis: Chiralcel AS-H (Hexane/*i*-PrOH = 95/5; 0.5 mL/min; λ = 210 nm; $t_{(S)\text{-}\mathbf{4m}}$ = 18.9 min, $t_{(R)\text{-}\mathbf{4m}}$ = 20.6 min).

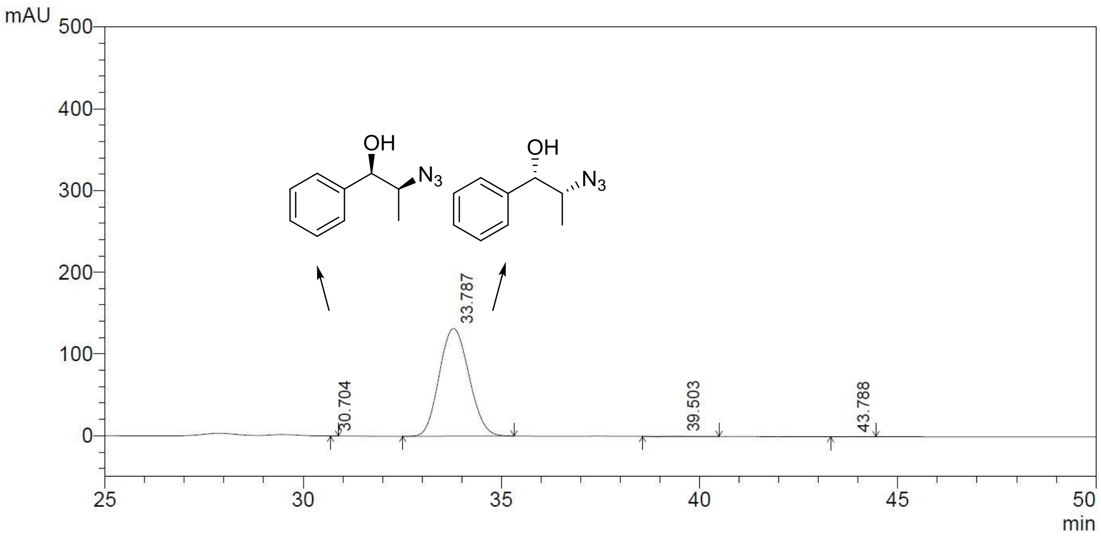

PDA

ID#	Rt. Time	Area	Height	Area %
1	30.704	198	18	0.003
2	33.787	6880866	131701	99.927
3	39.503	4297	112	0.062
4	43.788	500	49	0.007

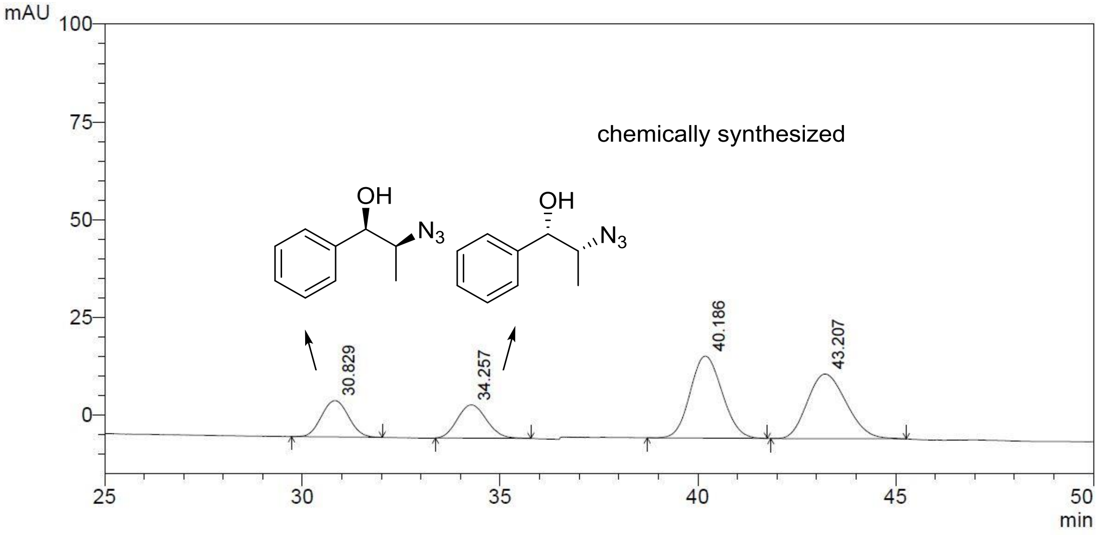

PDA

ID#	Rt. Time	Area	Height	Area %
1	30.829	441133	9315	13.797
2	34.257	436115	8523	13.640
3	40.186	1183822	21025	37.024
4	43.207	1136343	16552	35.539

Chiral HPLC analysis: Chiralcel OJ-H (Hexane/*i*-PrOH = 95/5; 0.5 mL/min; λ = 210 nm; $t_{(1S,2R)\text{-}\mathbf{4n}}$ = 33.8 min, $t_{(1R,2S)\text{-}\mathbf{4n}}$ = 30.7 min).

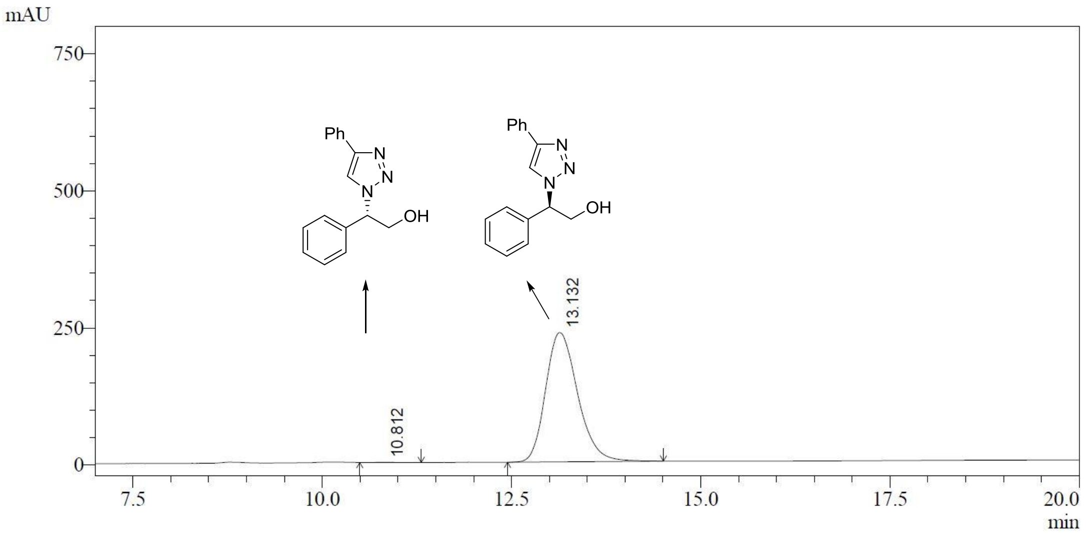

PDA

ID#	Rt. Time	Area	Height	Area %
1	10.812	3059	283	0.044
2	13.132	6940965	235671	99.956

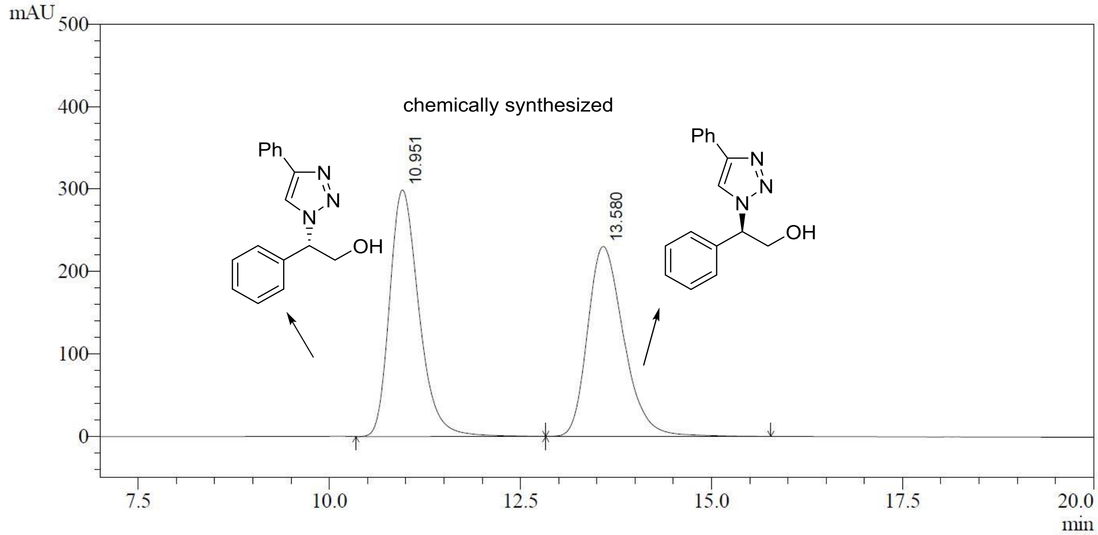

PDA

ID#	Rt. Time	Area	Height	Area %
1	10.951	8002032	298967	51.279
2	13.580	7602819	230363	48.721

Chiral HPLC analysis: Chiralcel OD-H (Hexane/*i*-PrOH = 80/20; 1 mL/min; λ = 210 nm; $t_{(R)\text{-}\mathbf{5a}}$ = 13.1 min, $t_{(S)\text{-}\mathbf{5a}}$ = 10.8 min).

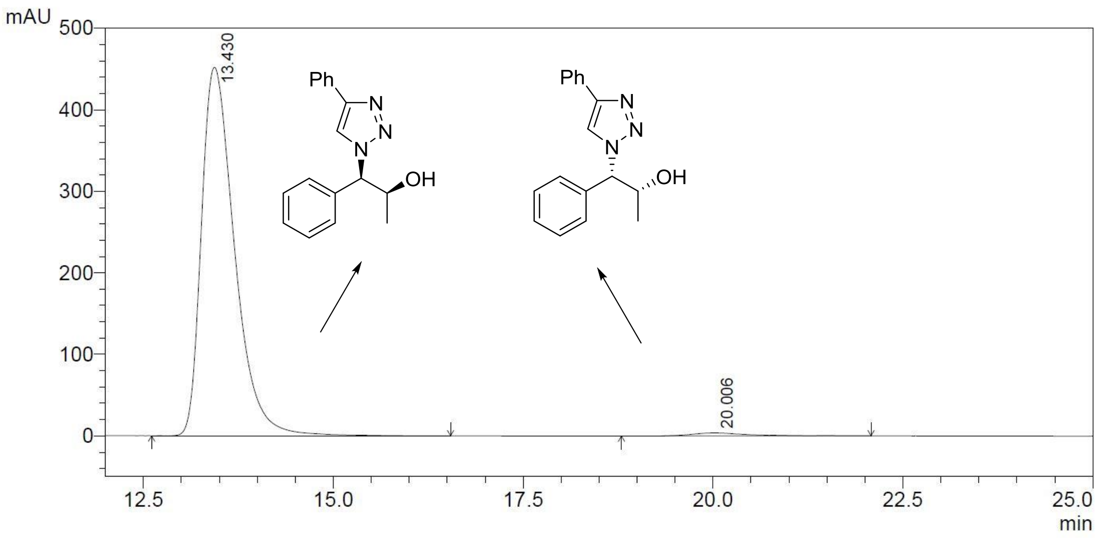

PDA

ID#	Rt. Time	Area	Height	Area %
1	13.430	13712078	451826	98.716
2	20.006	178333	3741	1.284

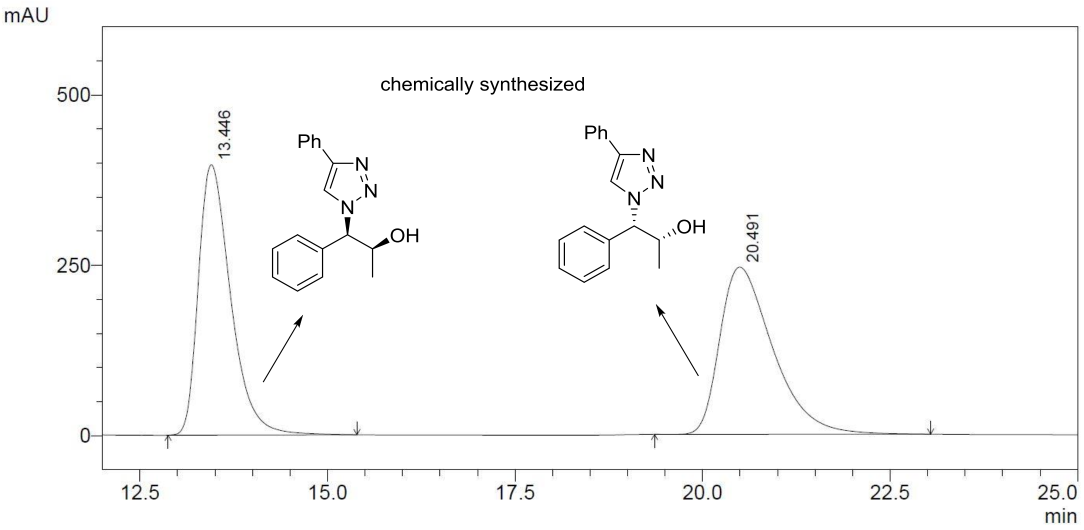

PDA

ID#	Rt. Time	Area	Height	Area %
1	13.446	11895945	396605	49.554
2	20.491	12110060	245261	50.446

Chiral HPLC analysis: Chiralcel OJ-H (Hexane/*i*-PrOH = 80/20; 1 mL/min; λ = 210 nm; $t_{(1R,2S)\text{-}\mathbf{5n}}$ = 13.4 min, $t_{(1S,2R)\text{-}\mathbf{5n}}$ = 20.0 min).

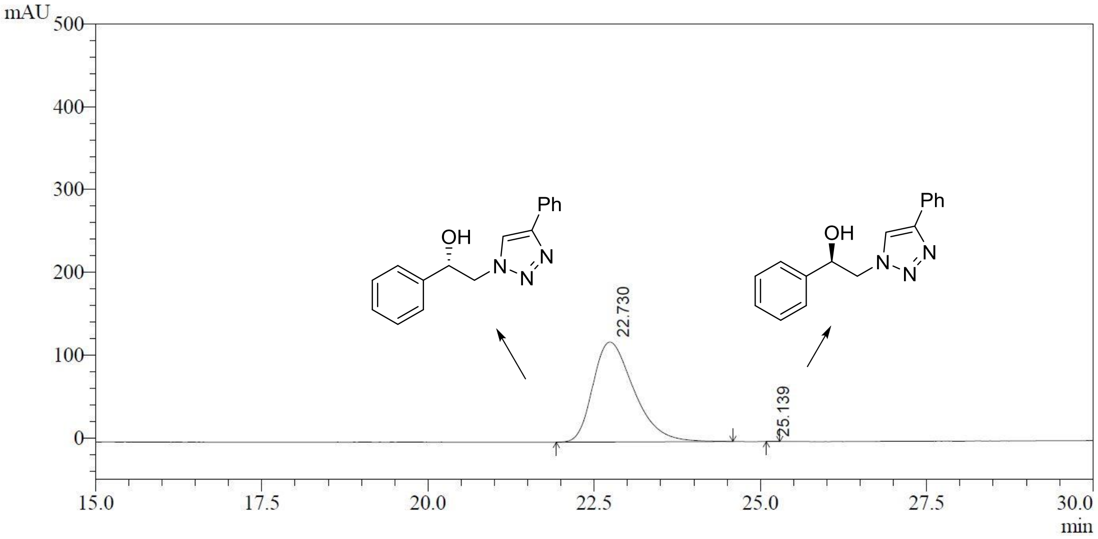

PDA

ID#	Rt. Time	Area	Height	Area %
1	22.730	5175790	120687	99.999
2	25.139	35	42	0.001

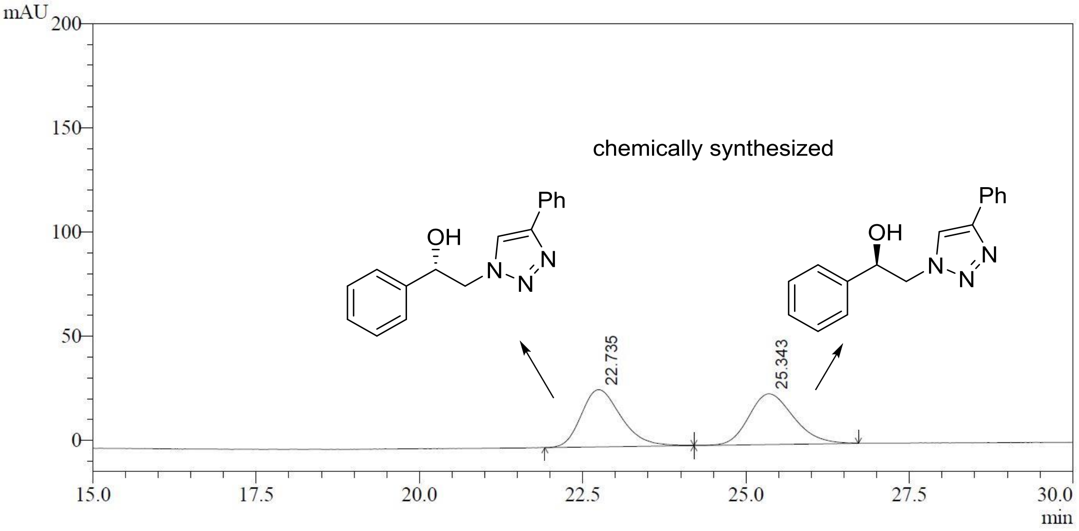

PDA

ID#	Rt. Time	Area	Height	Area %
1	22.735	1171837	27499	50.144
2	25.343	1165120	24335	49.856

Chiral HPLC analysis: Chiralcel OJ-H (Hexane/*i*-PrOH = 80/20; 1 mL/min; λ = 210 nm; $t_{(S)\text{-}\mathbf{6a}}$ = 22.7 min, $t_{(R)\text{-}\mathbf{6a}}$ = 25.1 min).

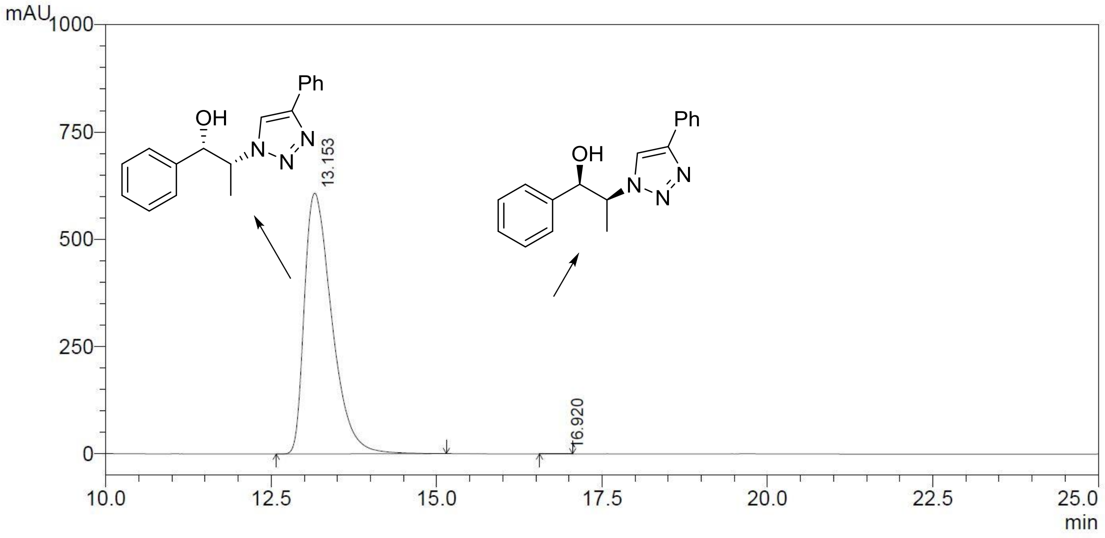

PDA

ID#	Rt. Time	Area	Height	Area %
1	13.153	17550256	607446	99.998
2	16.920	390	151	0.002

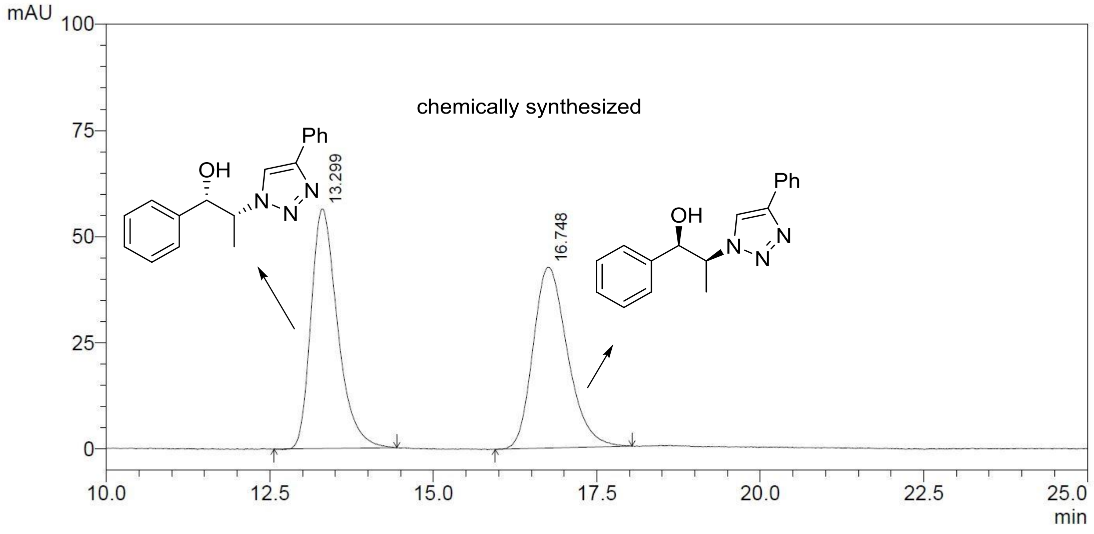

PDA

ID#	Rt. Time	Area	Height	Area %
1	13.299	1592968	56397	50.452
2	16.748	1564439	42563	49.548

Chiral HPLC analysis: Chiralcel OJ-H (Hexane/*i*-PrOH = 80/20; 1 mL/min; λ = 210 nm; $t_{(1S,2R)\text{-}\mathbf{6n}}$ = 13.2 min, $t_{(1R,2S)\text{-}\mathbf{6n}}$ = 16.9 min).

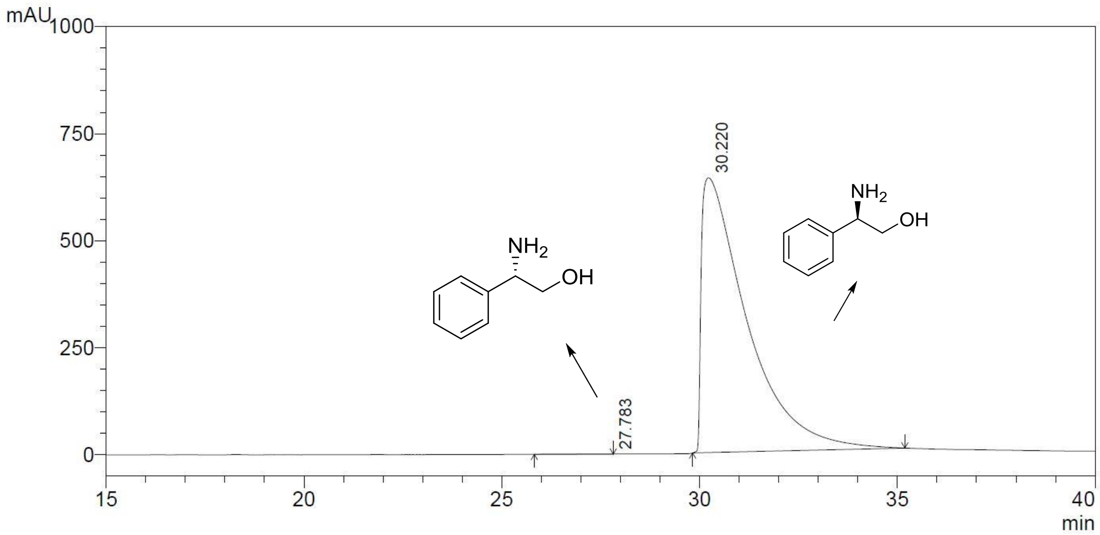

PDA

ID#	Rt. Time	Area	Height	Area %
1	27.783	11871	21	0.023
2	30.220	51364633	641557	99.977

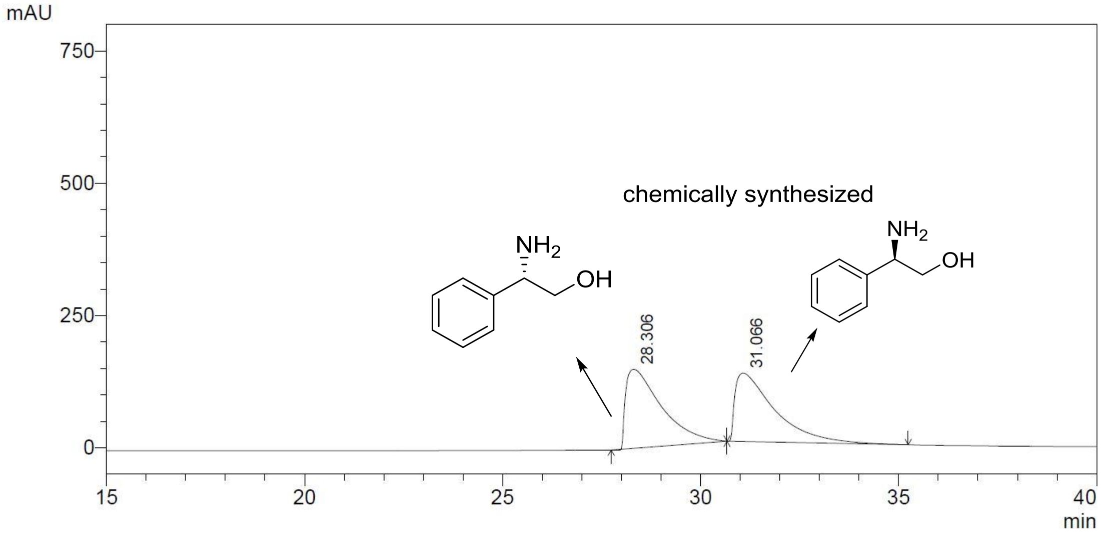

PDA

ID#	Rt. Time	Area	Height	Area %
1	28.306	9378501	149386	51.006
2	31.066	9008460	129160	48.994

Chiral HPLC analysis: Chiralcel OJ-H (Hexane/*i*-PrOH = 95/5; 0.5 mL/min; λ = 210 nm; $t_{(R)\text{-}\mathbf{7a}}$ = 30.2 min, $t_{(S)\text{-}\mathbf{7a}}$ = 27.8 min).

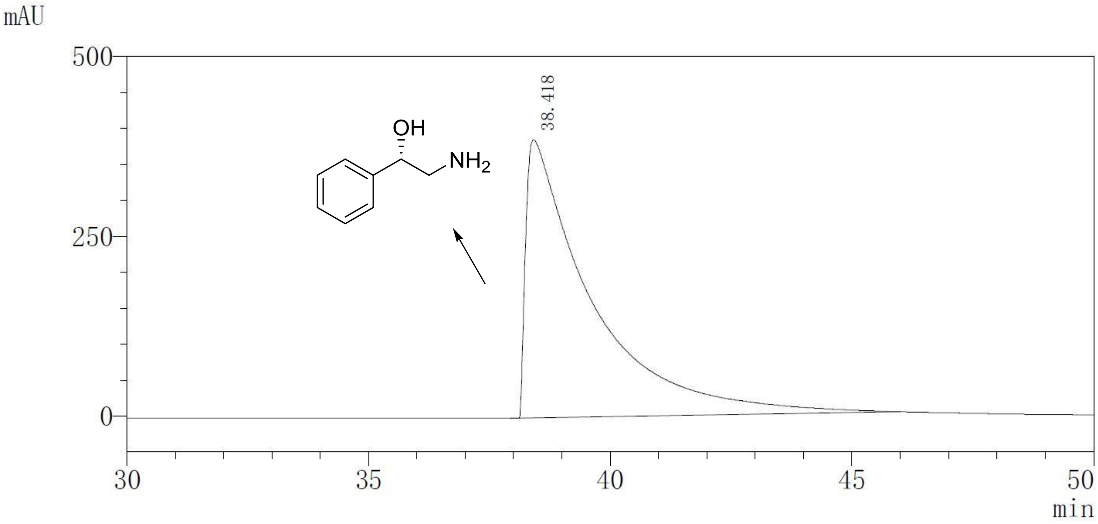

PDA

ID#	Rt. Time	Area	Height	Area%
1	38.418	36691169	386827	100.000

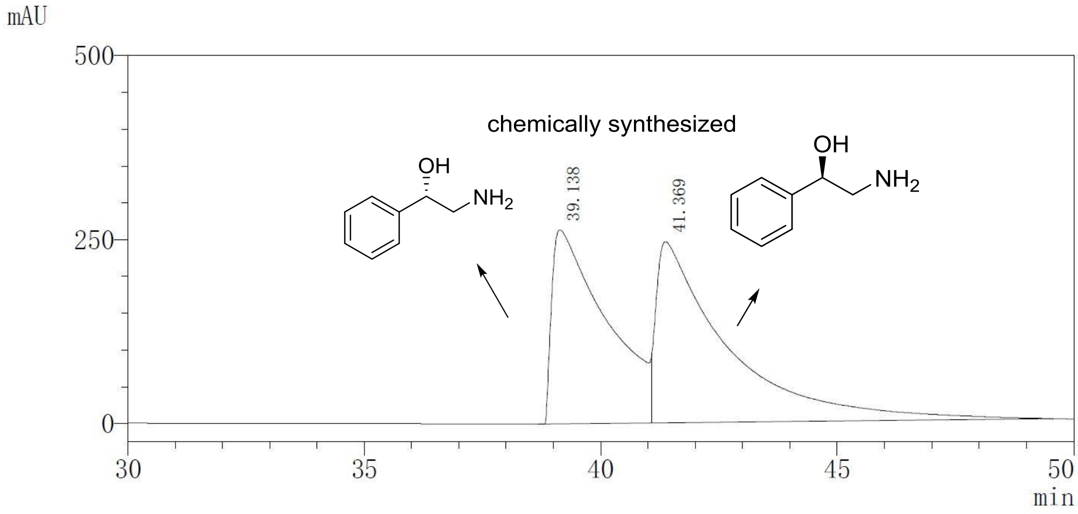

PDA

ID#	Rt. Time	Area	Height	Area%
1	39.138	21078527	263709	44.911
2	41.369	25855197	246042	55.089

Chiral HPLC analysis: Chiralcel IH (Hexane/*i*-PrOH = 95/5; 0.5 mL/min; λ = 210 nm; $t_{(S)\text{-}\mathbf{8a}}$ = 39.1 min, $t_{(R)\text{-}\mathbf{8a}}$ = 41.4 min).

NMR spectra

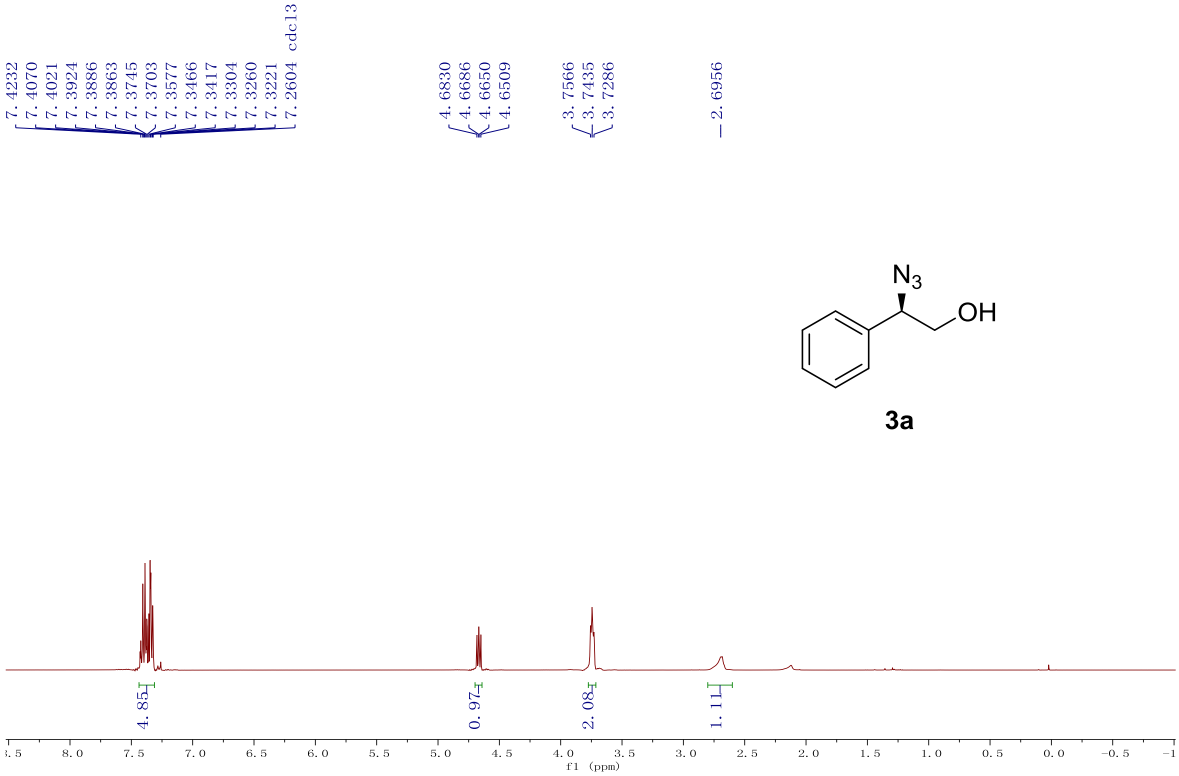
7.4232
7.4070
7.4021
7.3924
7.3886
7.3863
7.3745
7.3703
7.3577
7.3466
7.3417
7.3304
7.3260
7.3221
7.2604 cdcl3
4.6830
4.6686
4.6650
4.6509
3.7566
3.7435
3.7286
2.6956
N3
OH
3a
4.85
0.97
2.08
1.11
f1 (ppm)

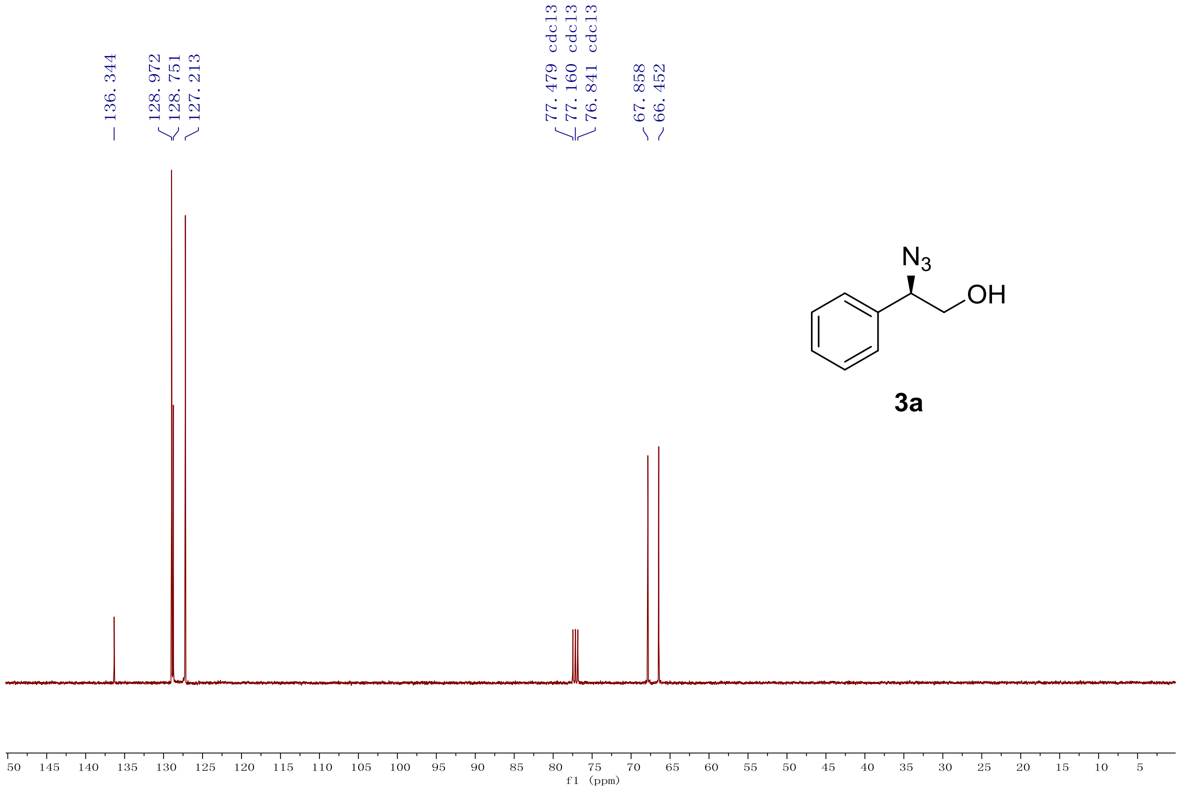
136.344
128.972
128.751
127.213
77.479 cdcl3
77.160 cdcl3
76.841 cdcl3
67.858
66.452
N3
OH
3a
f1 (ppm)

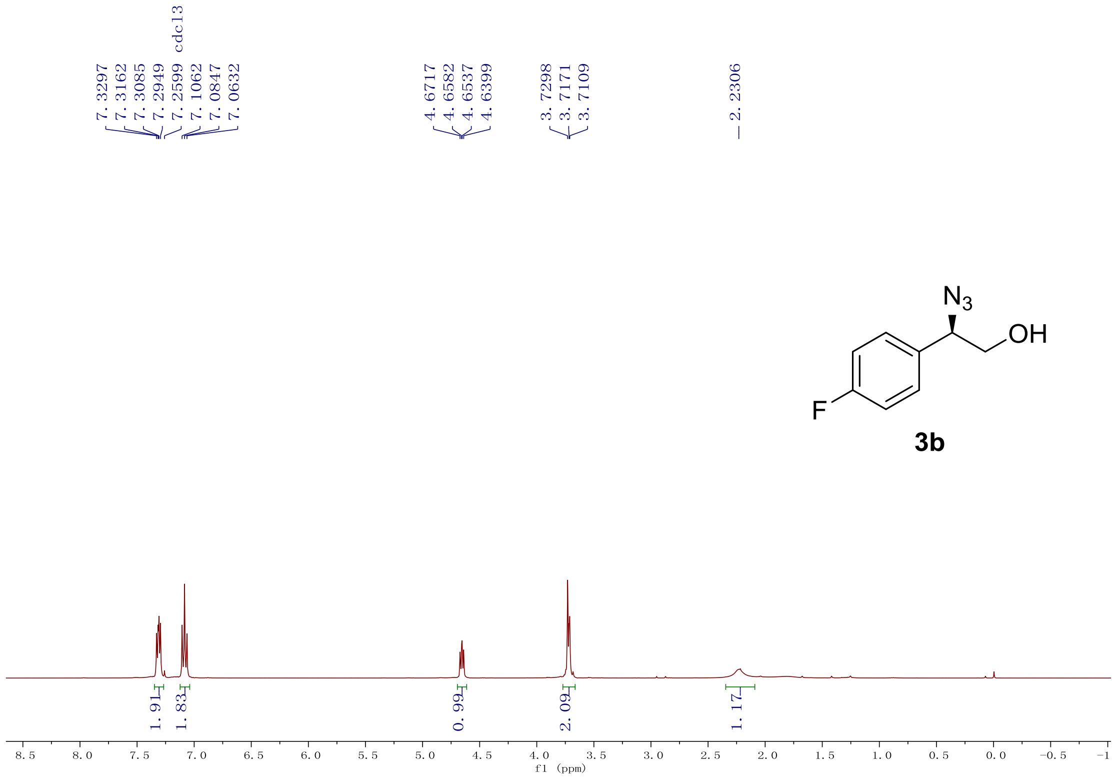
7.3297
7.3162
7.3085
7.2949
7.2599 cdcl3
7.1062
7.0847
7.0632
4.6717
4.6582
4.6537
4.6399
3.7298
3.7171
3.7109
2.2306
N3
OH
F
3b
1.91
1.83
0.99
2.09
1.17
f1 (ppm)

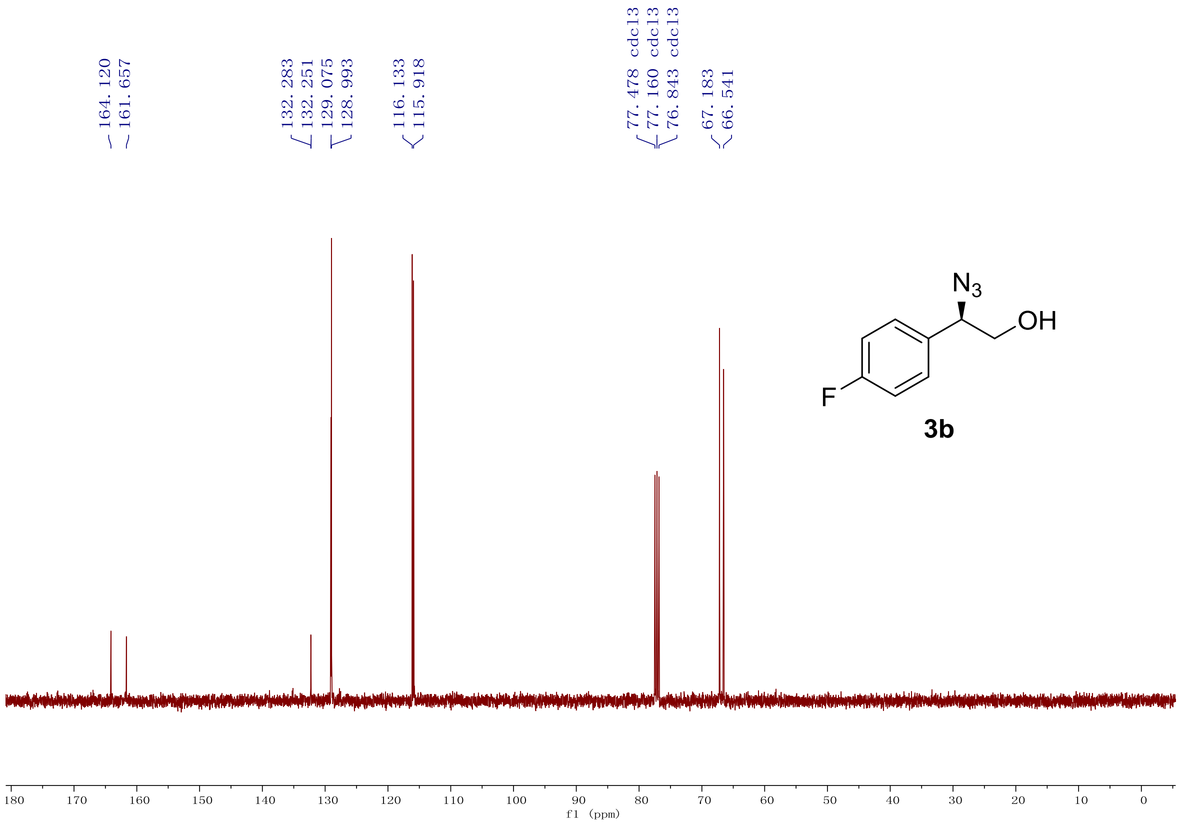
164.120
161.657
132.283
132.251
129.075
128.993
116.133
115.918
77.478 cdcl3
77.160 cdcl3
76.843 cdcl3
67.183
66.541
N3
OH
F
3b
f1 (ppm)

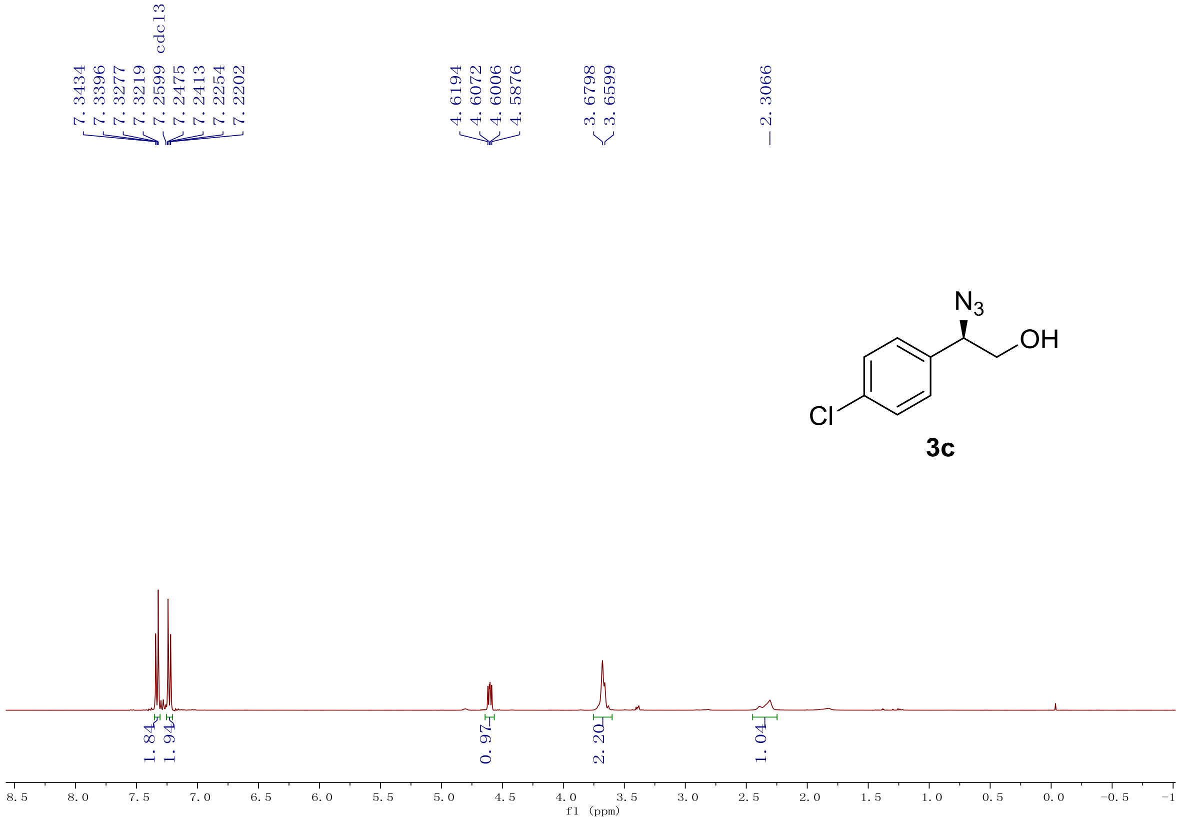
7.3434
7.3396
7.3277
7.3219
7.2599 cdcl3
7.2475
7.2413
7.2254
7.2202
4.6194
4.6072
4.6006
4.5876
3.6798
3.6599
2.3066
N3
OH
Cl
3c
1.84
1.94
0.97
2.20
1.04
f1 (ppm)

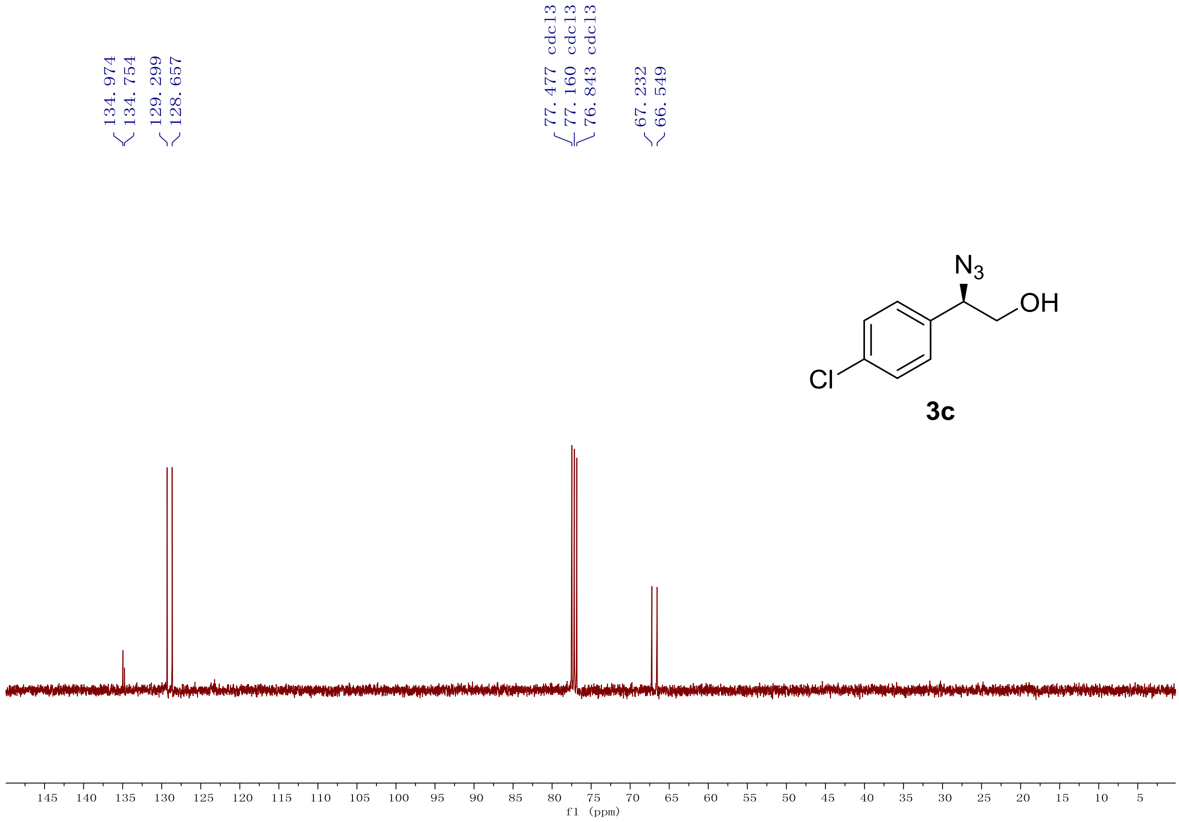
134.974
134.754
129.299
128.657
77.477 cdcl3
77.160 cdcl3
76.843 cdcl3
67.232
66.549
N3
OH
Cl
3c
f1 (ppm)

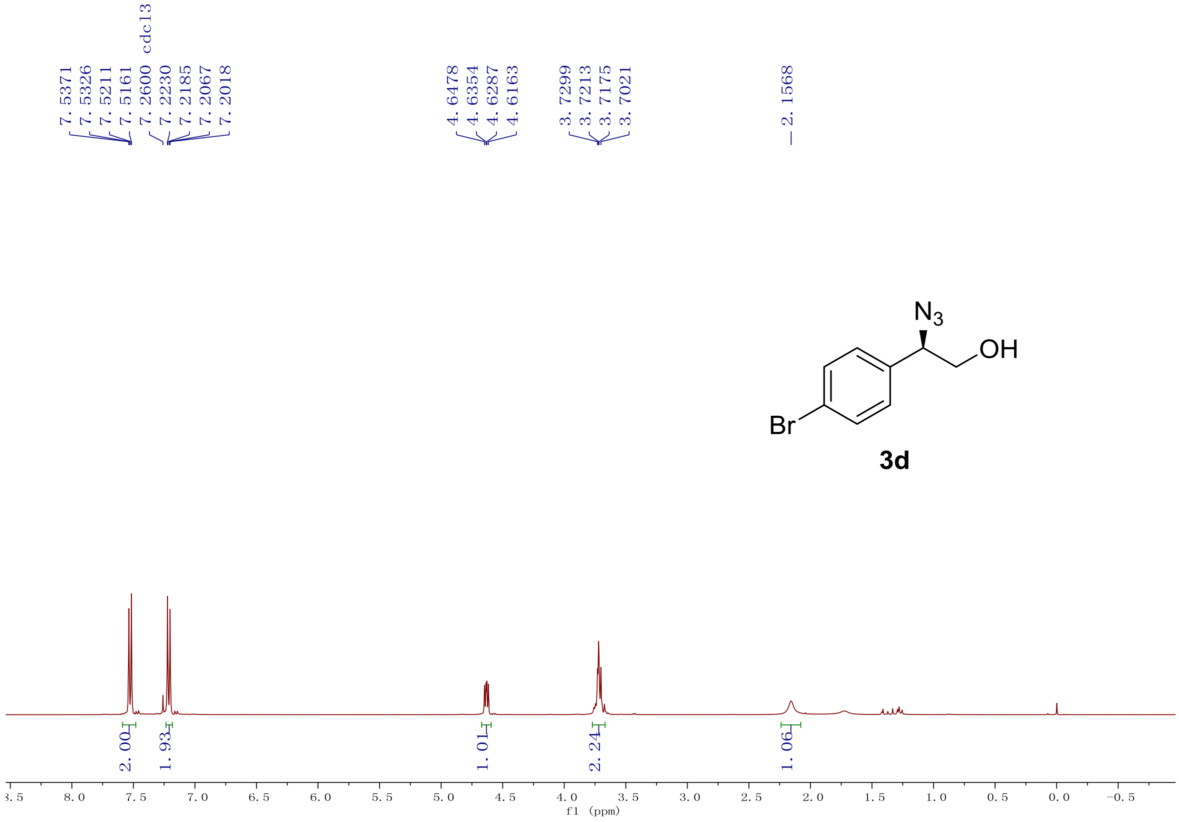
7.5371
7.5326
7.5211
7.5161
7.2600 cdcl3
7.2230
7.2185
7.2067
7.2018
4.6478
4.6354
4.6287
4.6163
3.7299
3.7213
3.7175
3.7021
2.1568
N3
OH
Br
3d
2.00
1.93
1.01
2.24
1.06
f1 (ppm)

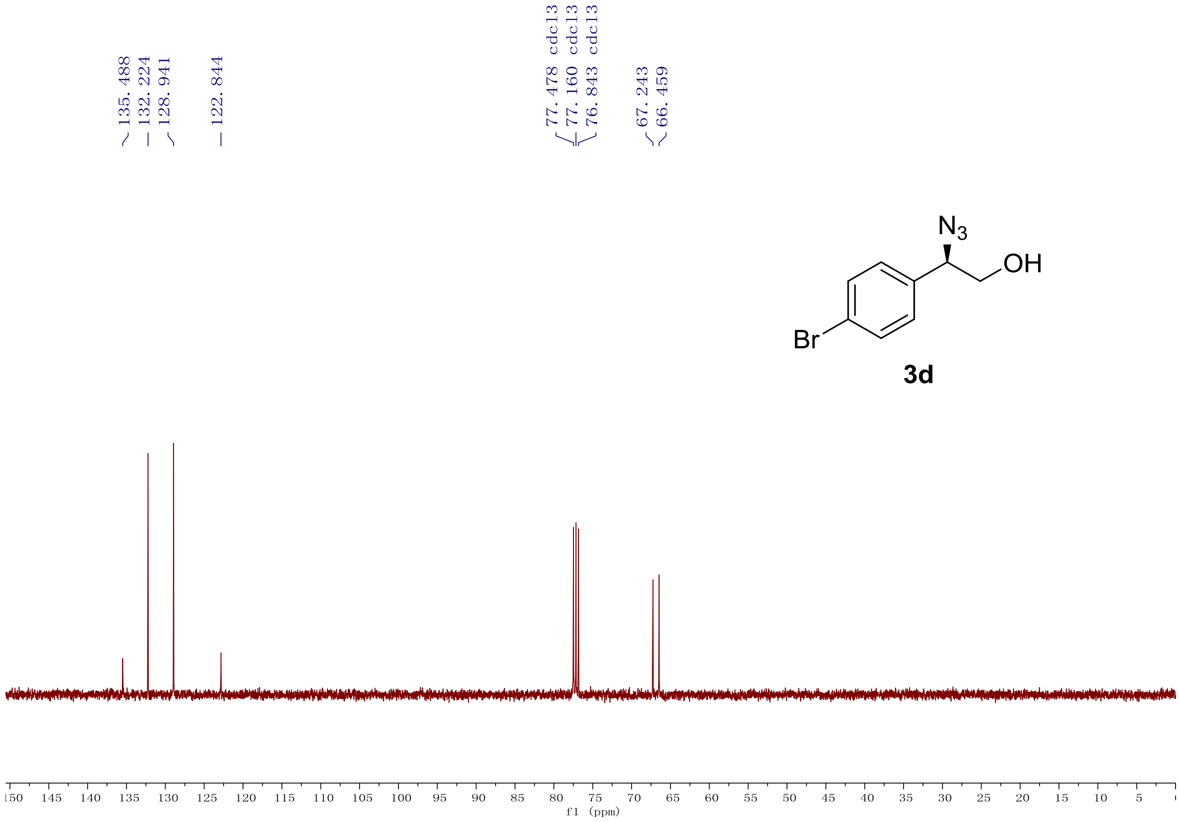
135.488
132.224
128.941
122.844
77.478 cdcl3
77.160 cdcl3
76.843 cdcl3
67.243
66.459
N3
OH
Br
3d
f1 (ppm)

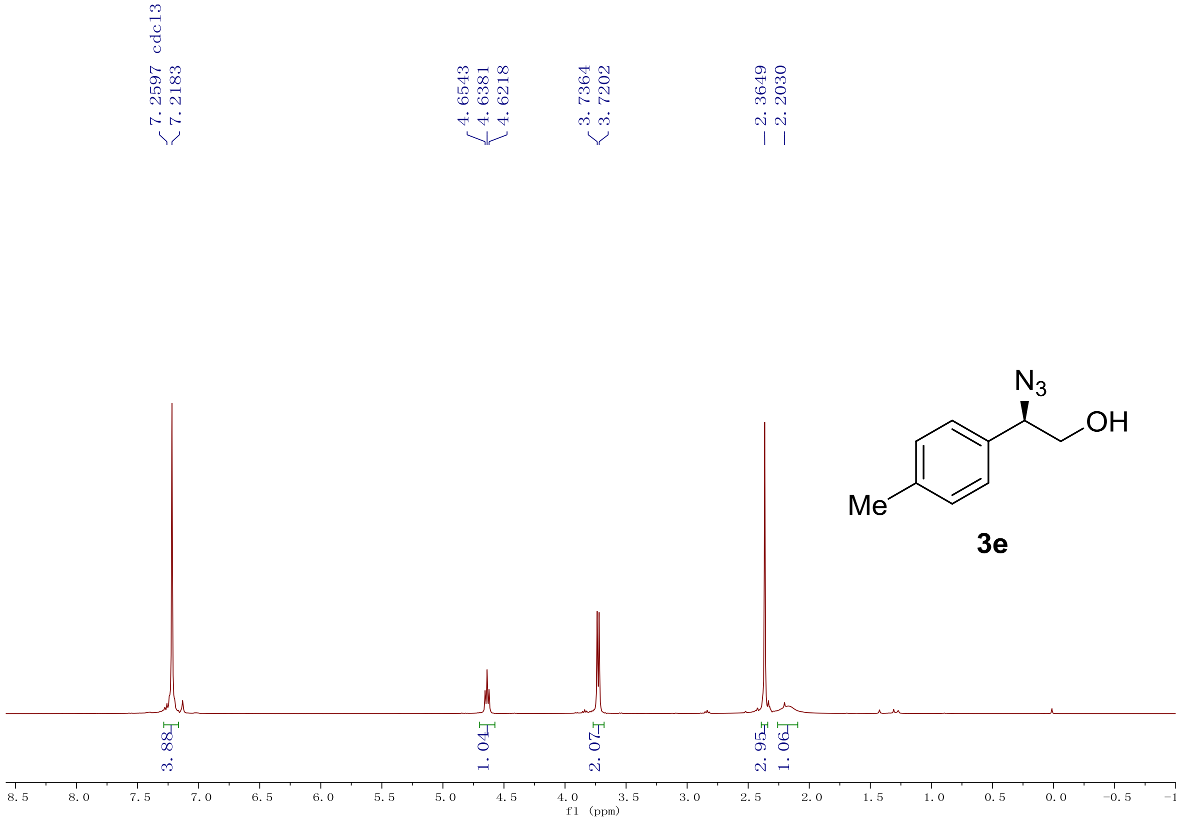
7.2597 cdcl3
7.2183
4.6543
4.6381
4.6218
3.7364
3.7202
2.3649
2.2030
3.88
1.04
2.07
2.95
1.06
8.5 8.0 7.5 7.0 6.5 6.0 5.5 5.0 4.5 4.0 3.5 3.0 2.5 2.0 1.5 1.0 0.5 0.0 -0.5
f1 (ppm)
N3
OH
Me
3e

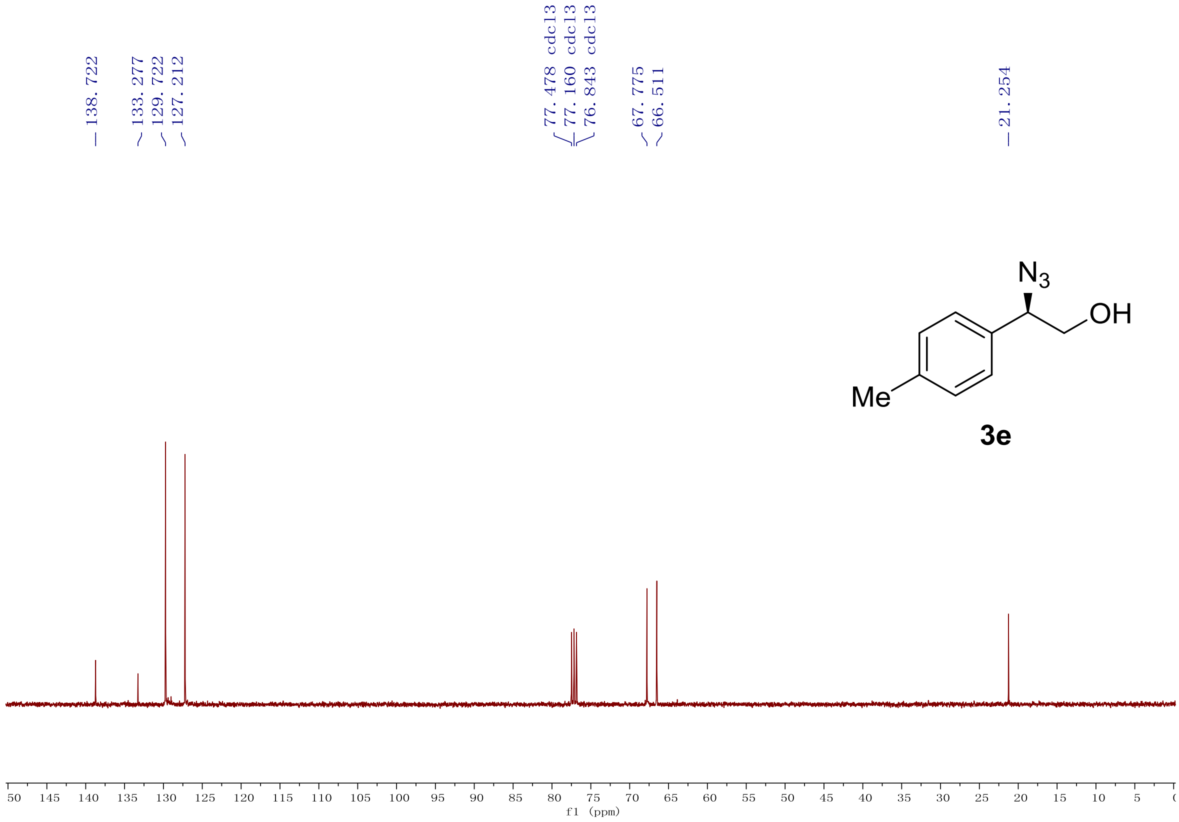
138.722
133.277
129.722
127.212
77.478 cdcl3
77.160 cdcl3
76.843 cdcl3
67.775
66.511
21.254
145 140 135 130 125 120 115 110 105 100 95 90 85 80 75 70 65 60 55 50 45 40 35 30 25 20 15 10 5
f1 (ppm)
N3
OH
Me
3e

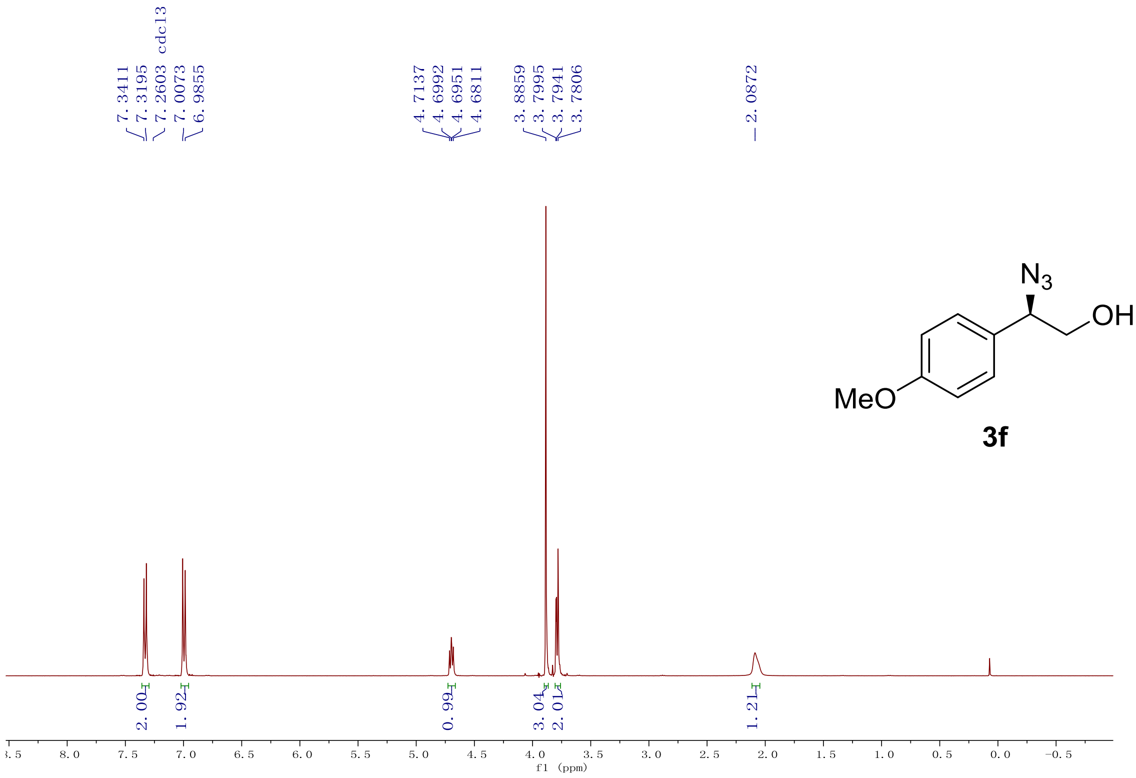
7.3411
7.3195
7.2603 cdcl3
7.0073
6.9855
4.7137
4.6992
4.6951
4.6811
3.8859
3.7995
3.7941
3.7806
2.0872
2.00
1.92
0.99
3.04
2.01
1.21
f1 (ppm)
N3
OH
MeO
3f

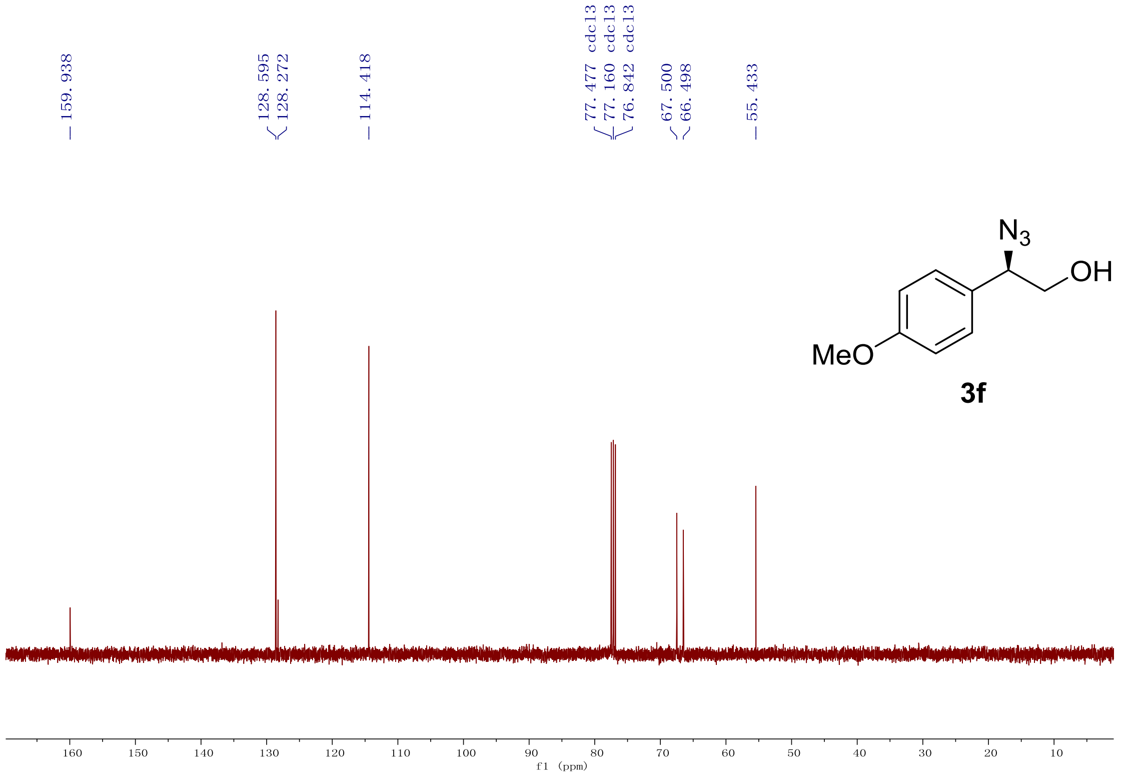
159.938
128.595
128.272
114.418
77.477 cdcl3
77.160 cdcl3
76.842 cdcl3
67.500
66.498
55.433
f1 (ppm)
N3
OH
MeO
3f

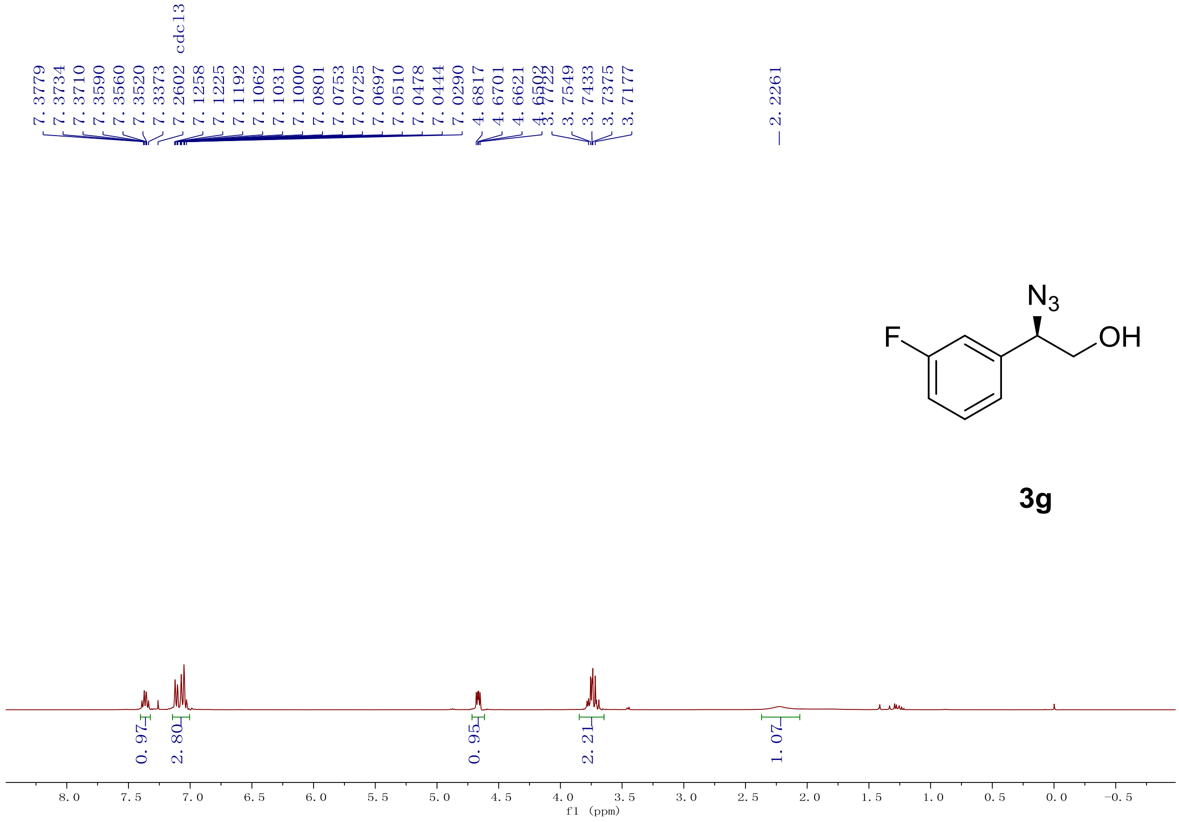
7.3779
7.3734
7.3710
7.3590
7.3560
7.3520
7.3373
7.2602 cdcl3
7.1258
7.1225
7.1192
7.1062
7.1031
7.1000
7.0801
7.0753
7.0725
7.0697
7.0510
7.0478
7.0444
7.0290
4.6817
4.6701
4.6621
4.6502
3.7722
3.7549
3.7433
3.7375
3.7177
2.2261
N3
F
OH
3g
0.97
2.80
0.95
2.21
1.07
8.0 7.5 7.0 6.5 6.0 5.5 5.0 4.5 4.0 3.5 3.0 2.5 2.0 1.5 1.0 0.5 0.0 -0.5
f1 (ppm)

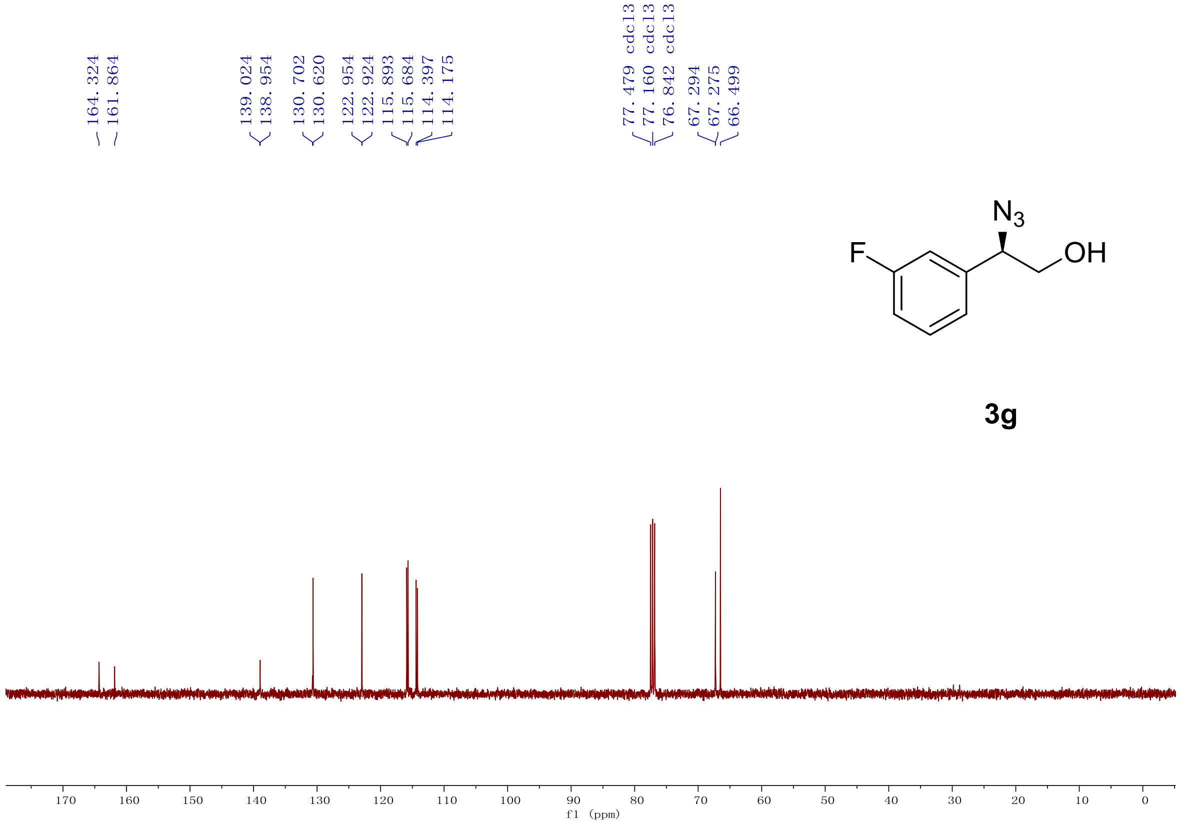
164.324
161.864
139.024
138.954
130.702
130.620
122.954
122.924
115.893
115.684
114.397
114.175
77.479 cdcl3
77.160 cdcl3
76.842 cdcl3
67.294
67.275
66.499
N3
F
OH
3g
170 160 150 140 130 120 110 100 90 80 70 60 50 40 30 20 10 0
f1 (ppm)

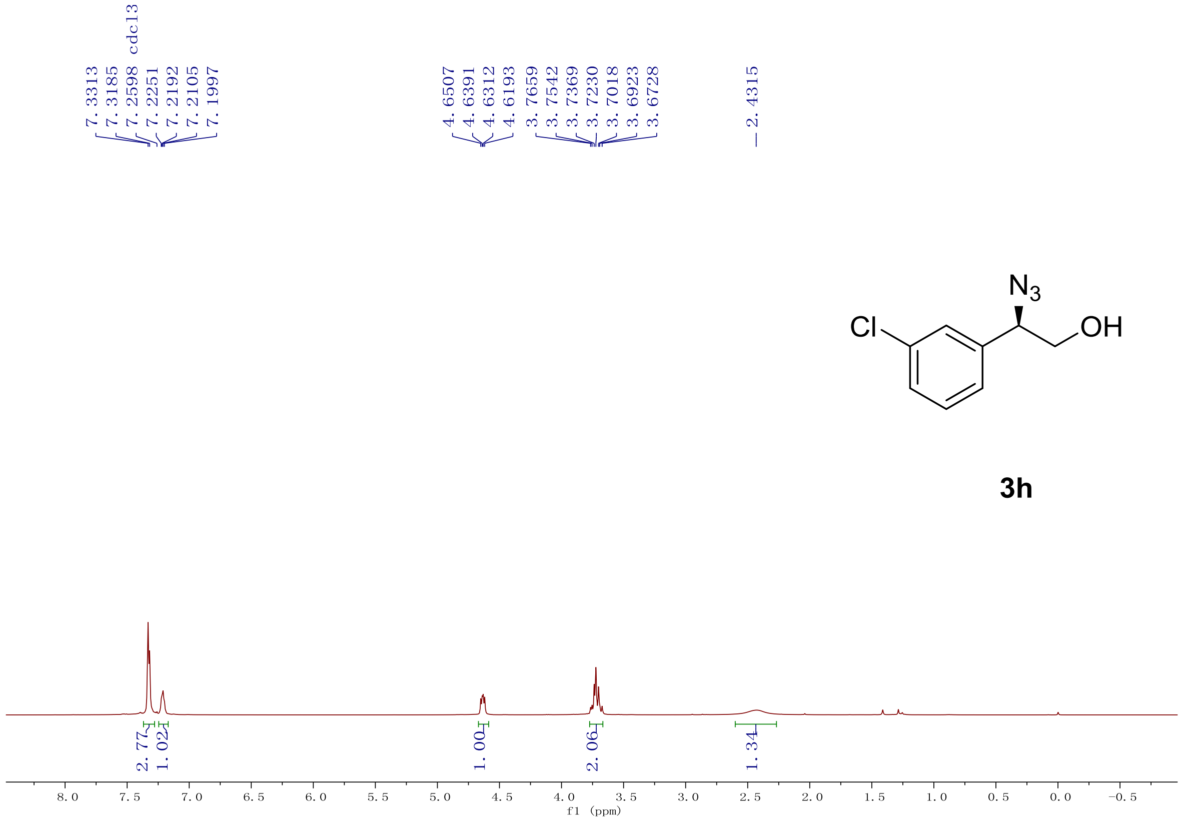
7.3313
7.3185
7.2598 cdcl3
7.2251
7.2192
7.2105
7.1997
4.6507
4.6391
4.6312
4.6193
3.7659
3.7542
3.7369
3.7230
3.7018
3.6923
3.6728
2.4315
2.77
1.02
1.00
2.06
1.34
f1 (ppm)
N3
Cl
OH

3h

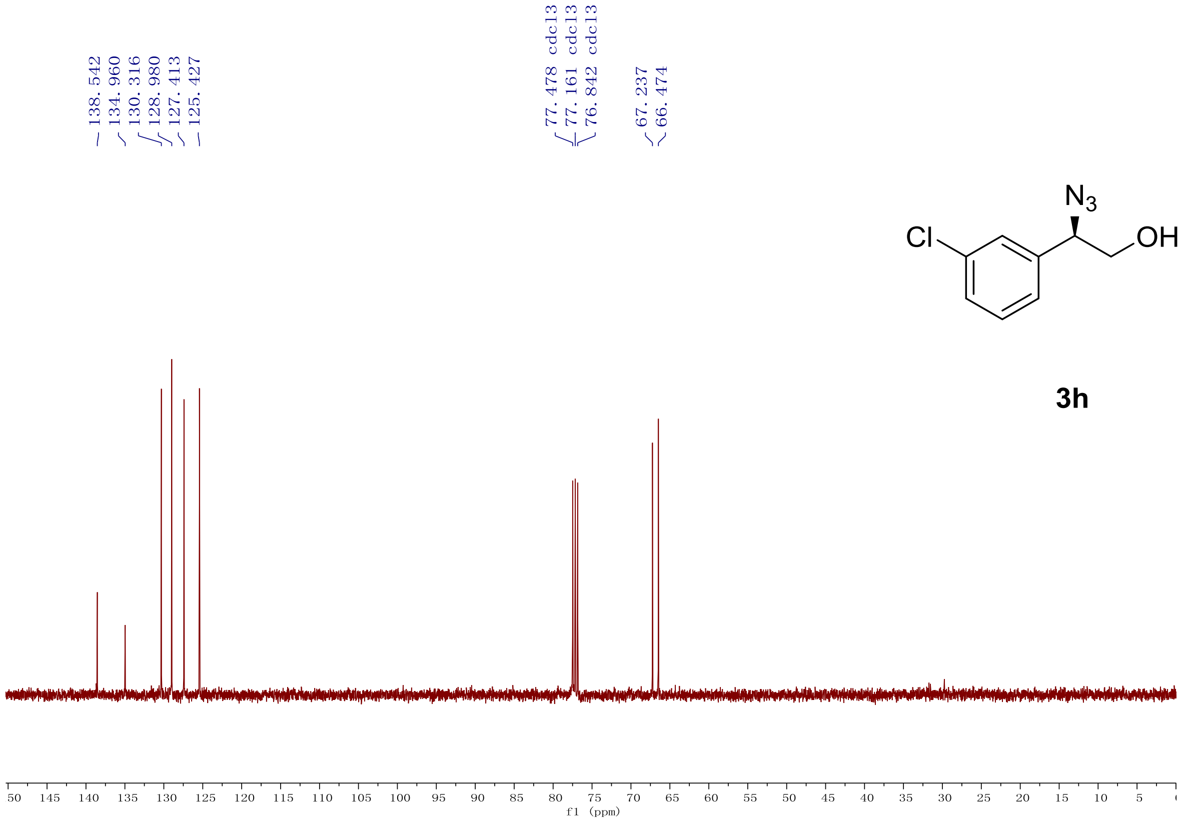
138.542
134.960
130.316
128.980
127.413
125.427
77.478 cdcl3
77.161 cdcl3
76.842 cdcl3
67.237
66.474
f1 (ppm)
N3
Cl
OH

3h

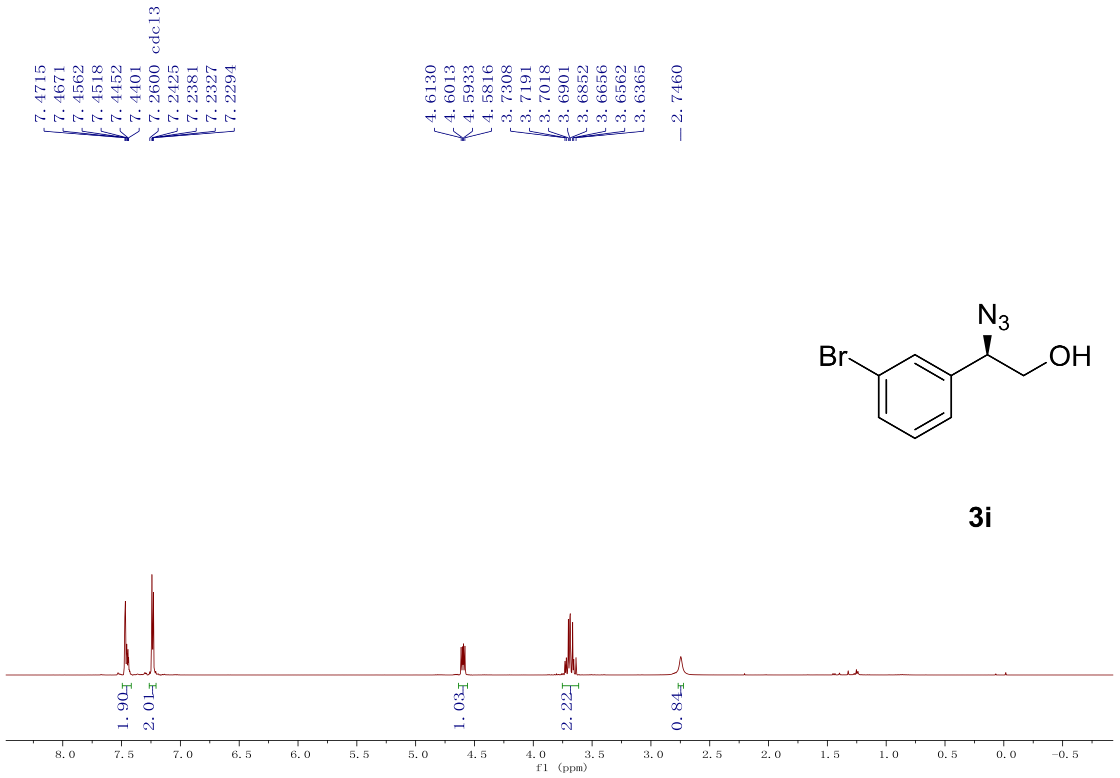
7.4715
7.4671
7.4562
7.4518
7.4452
7.4401
7.2600 cdcl3
7.2425
7.2381
7.2327
7.2294
4.6130
4.6013
4.5933
4.5816
3.7308
3.7191
3.7018
3.6901
3.6852
3.6656
3.6562
3.6365
2.7460
N3
Br
OH
1.90
2.01
1.03
2.22
0.84
f1 (ppm)

3i

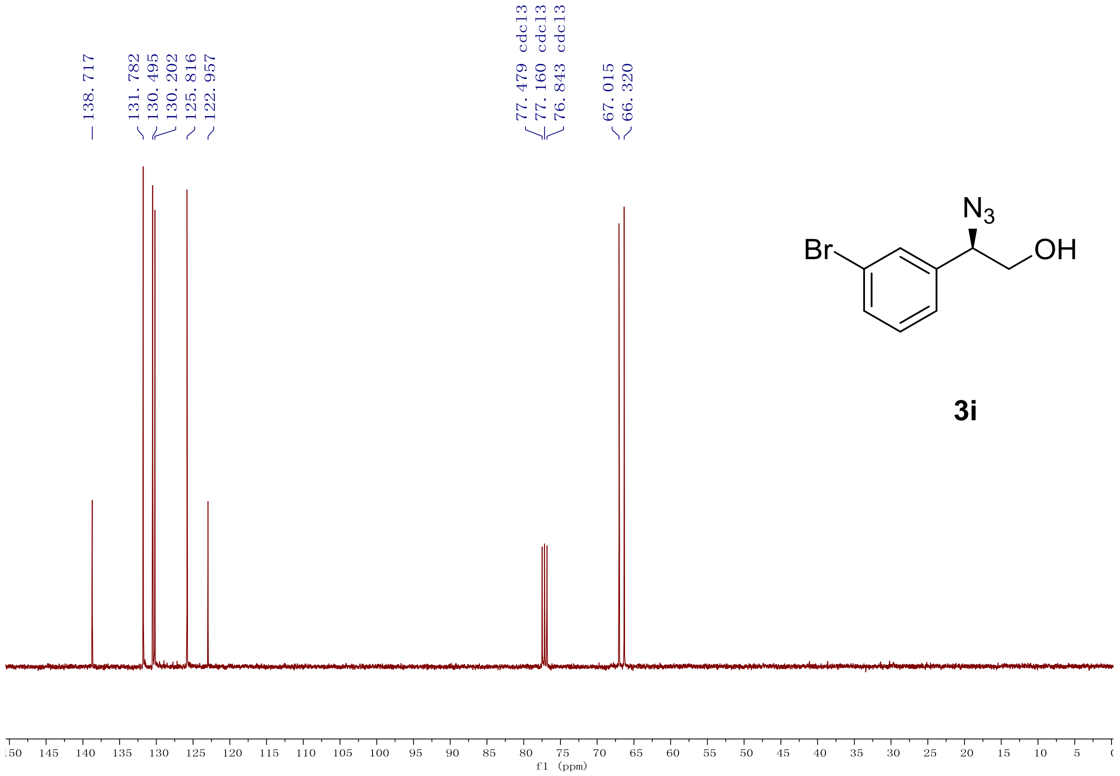
138.717
131.782
130.495
130.202
125.816
122.957
77.479 cdcl3
77.160 cdcl3
76.843 cdcl3
67.015
66.320
N3
Br
OH
f1 (ppm)

3i

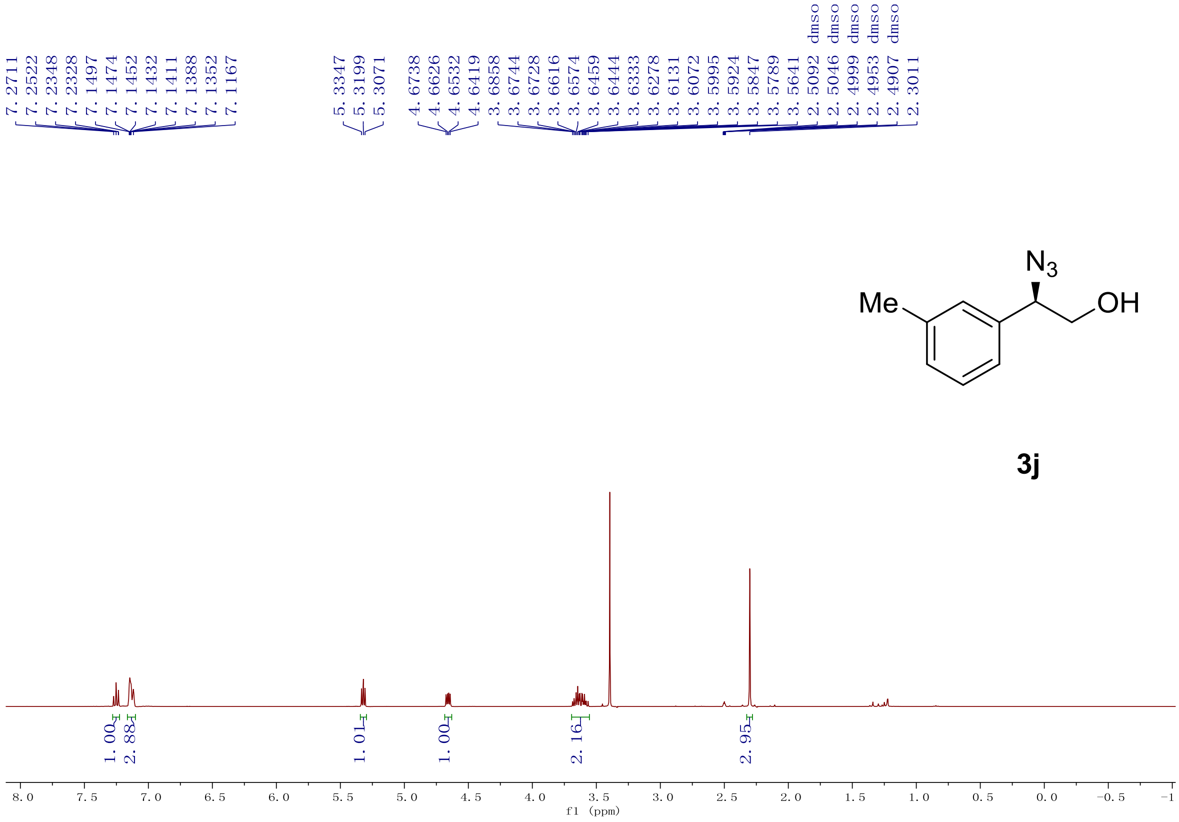
7.2711
7.2522
7.2348
7.2328
7.1497
7.1474
7.1452
7.1432
7.1411
7.1388
7.1352
7.1167
5.3347
5.3199
5.3071
4.6738
4.6626
4.6532
4.6419
3.6858
3.6744
3.6728
3.6616
3.6574
3.6459
3.6444
3.6333
3.6278
3.6131
3.6072
3.5995
3.5924
3.5847
3.5789
3.5641
2.5092 dmso
2.5046 dmso
2.4999 dmso
2.4953 dmso
2.4907 dmso
2.3011
N_3
Me
OH
3j
1.00
2.88
1.01
1.00
2.16
2.95
f1 (ppm)

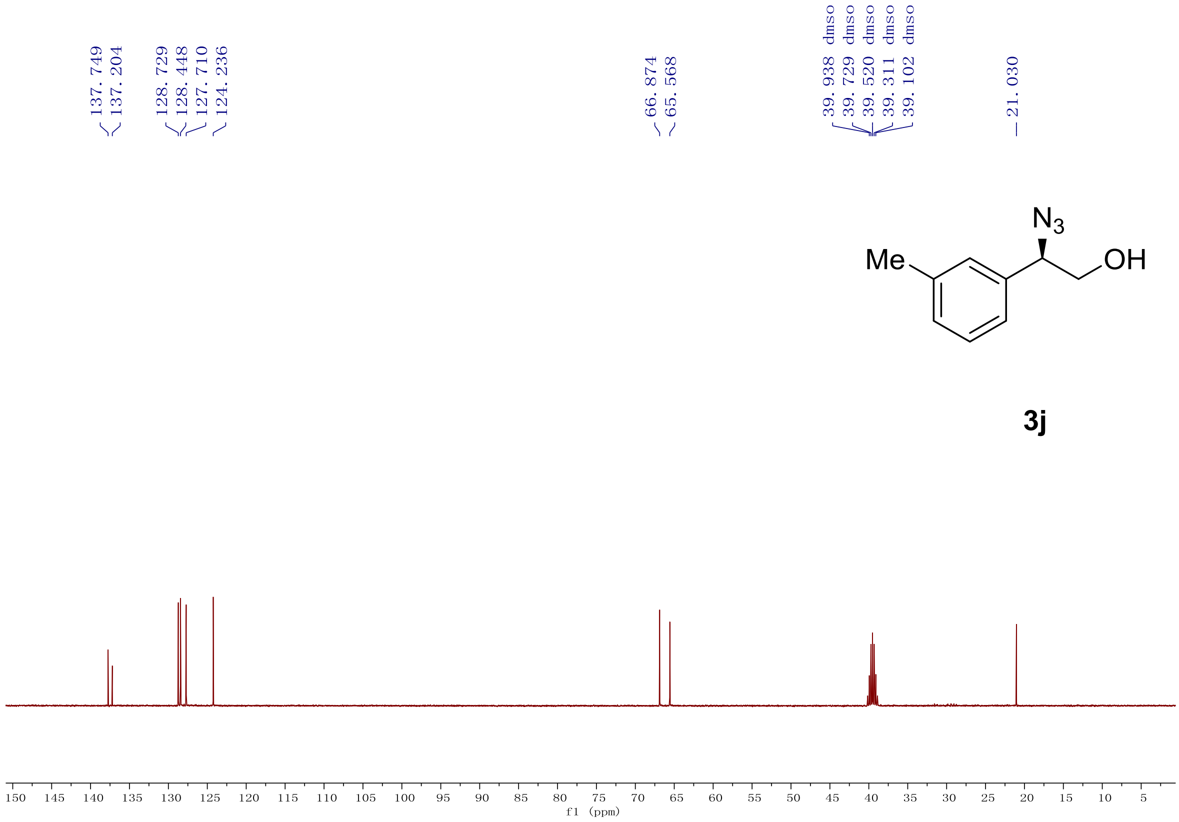
137.749
137.204
128.729
128.448
127.710
124.236
66.874
65.568
39.938 dmso
39.729 dmso
39.520 dmso
39.311 dmso
39.102 dmso
21.030
N_3
Me
OH
3j
f1 (ppm)

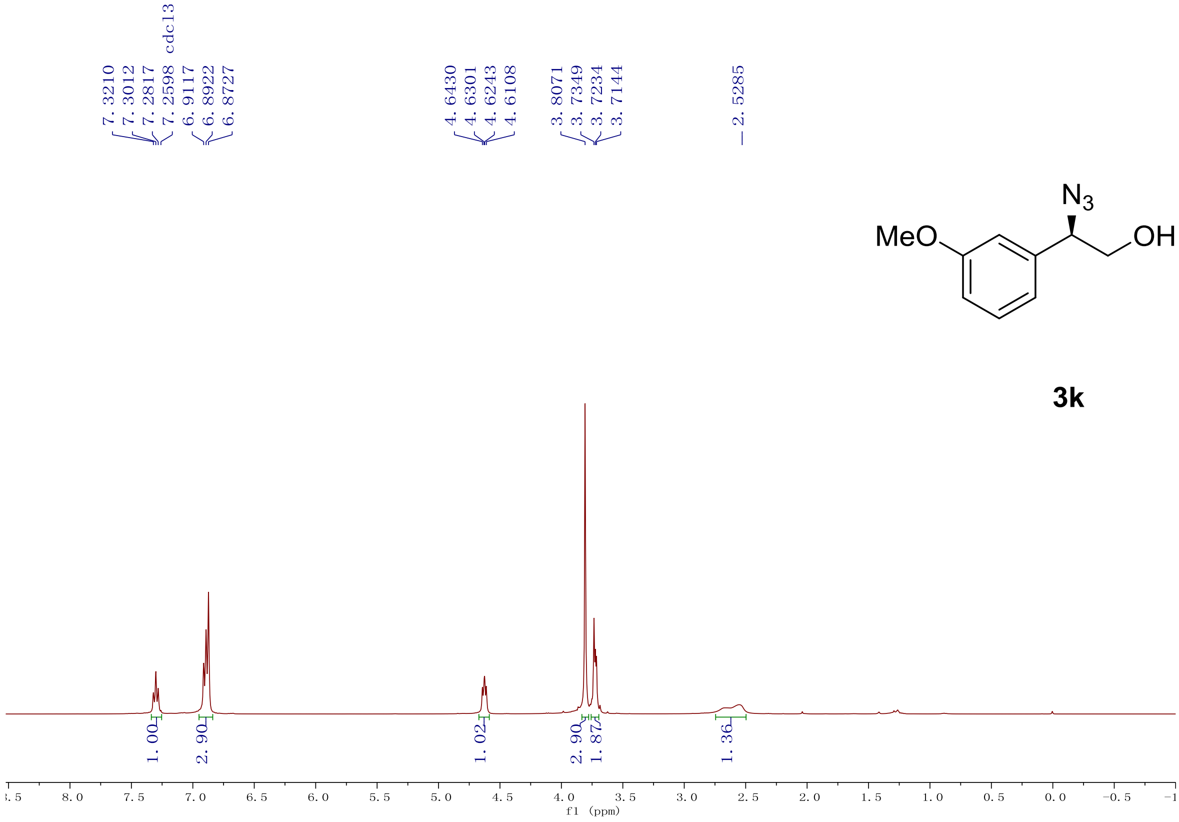

7.3210
7.3012
7.2817
7.2598 cdcl3
6.9117
6.8922
6.8727
4.6430
4.6301
4.6243
4.6108
3.8071
3.7349
3.7234
3.7144
2.5285
MeO
N3
OH
3k
1.00
2.90
1.02
2.90
1.87
1.36
f1 (ppm)

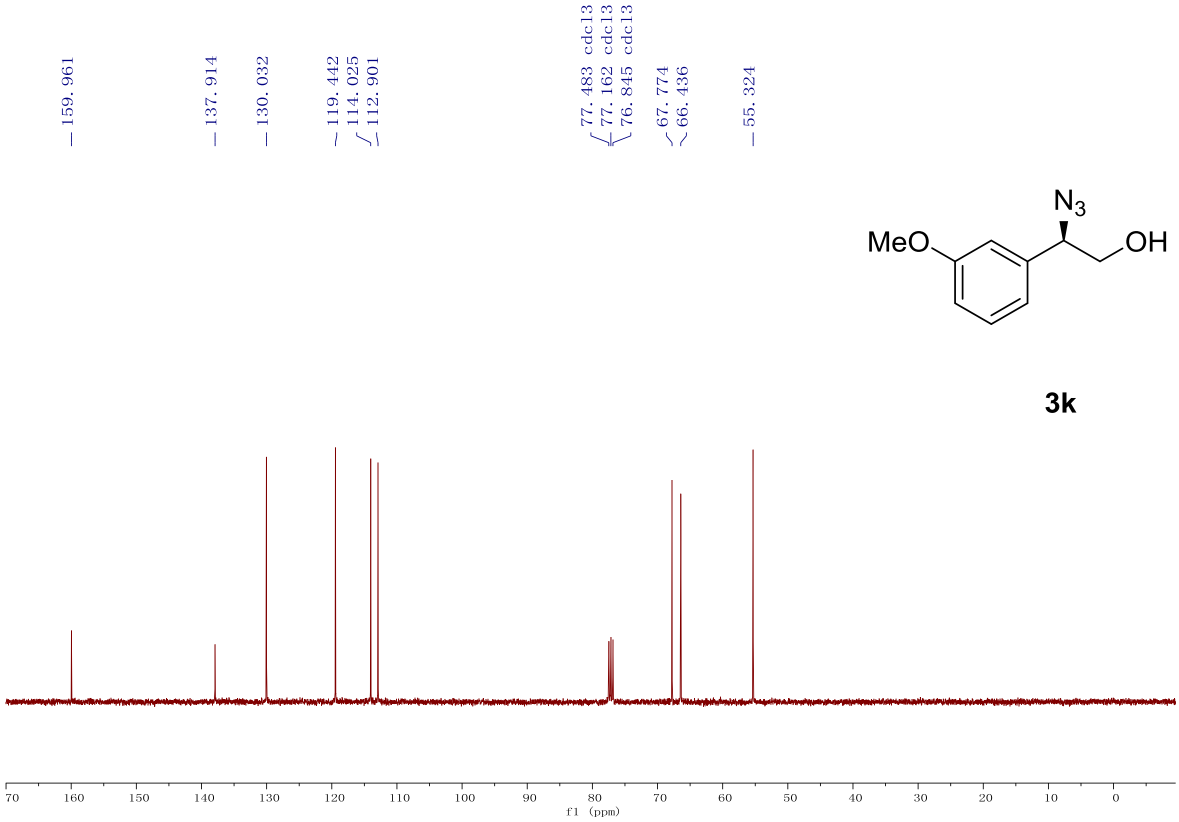

159.961
137.914
130.032
119.442
114.025
112.901
77.483 cdcl3
77.162 cdcl3
76.845 cdcl3
67.774
66.436
55.324
MeO
N3
OH
3k
f1 (ppm)

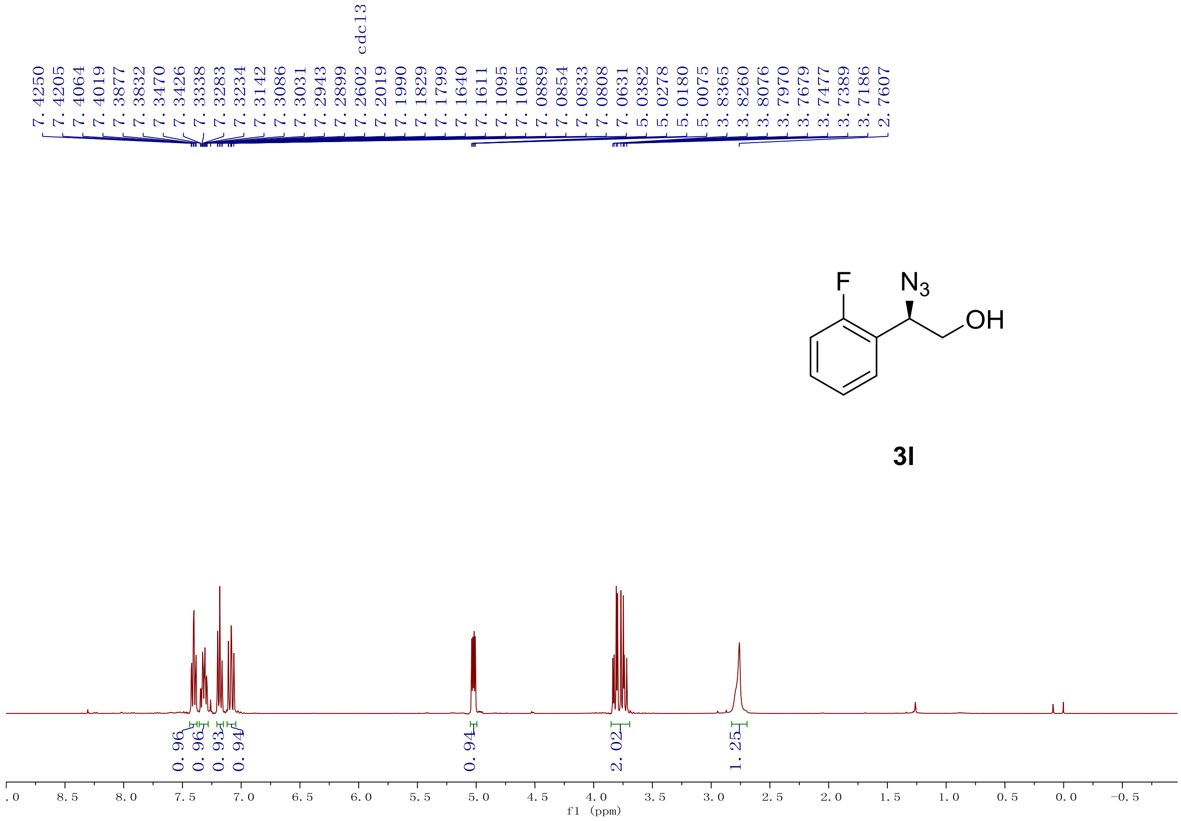

7.4250
7.4205
7.4064
7.4019
7.3877
7.3832
7.3470
7.3426
7.3338
7.3283
7.3234
7.3142
7.3086
7.3031
7.2943
7.2899
7.2602 cdcl3
7.2019
7.1990
7.1829
7.1799
7.1640
7.1611
7.1095
7.1065
7.0889
7.0854
7.0833
7.0808
7.0631
5.0382
5.0278
5.0180
5.0075
3.8365
3.8260
3.8076
3.7970
3.7679
3.7477
3.7389
3.7186
2.7607
F
N3
OH
0.96
0.96
0.93
0.94
0.94
2.02
1.25
f1 (ppm)

3l

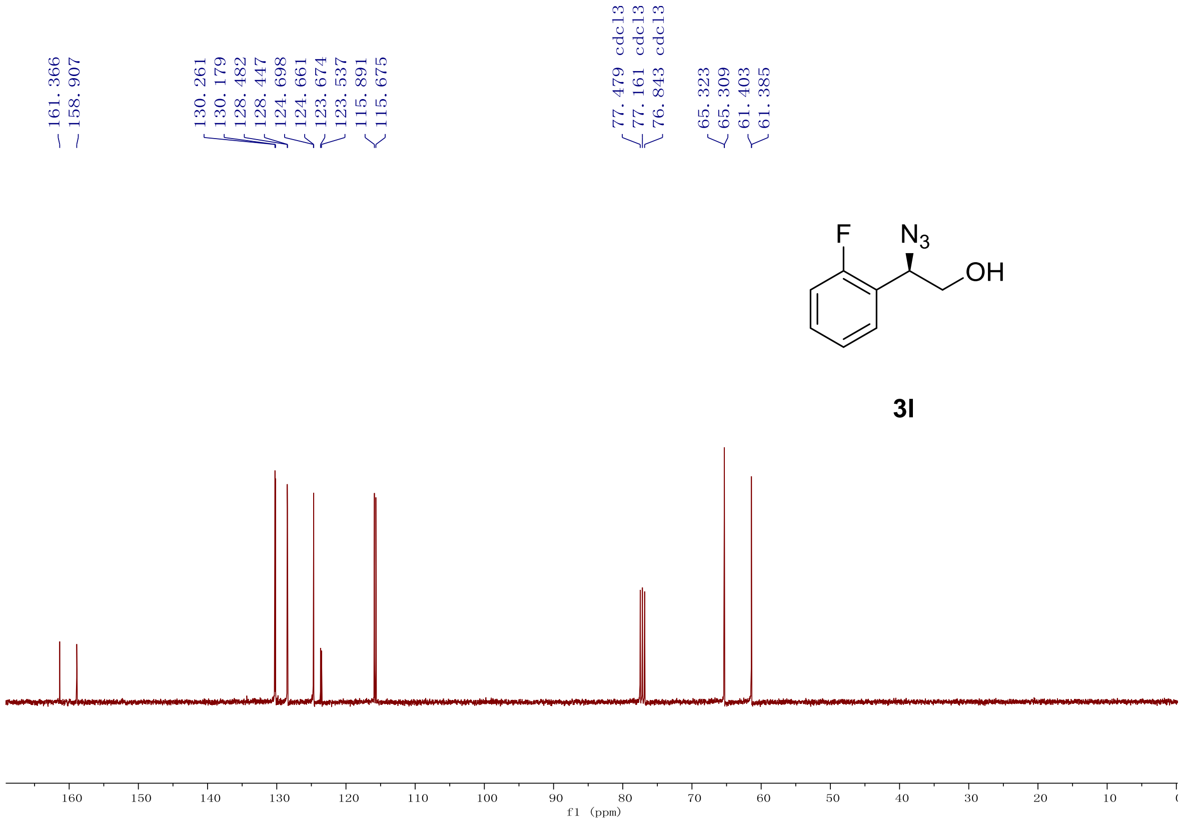

161.366
158.907
130.261
130.179
128.482
128.447
124.698
124.661
123.674
123.537
115.891
115.675
77.479 cdcl3
77.161 cdcl3
76.843 cdcl3
65.323
65.309
61.403
61.385
F
N3
OH
f1 (ppm)

3l

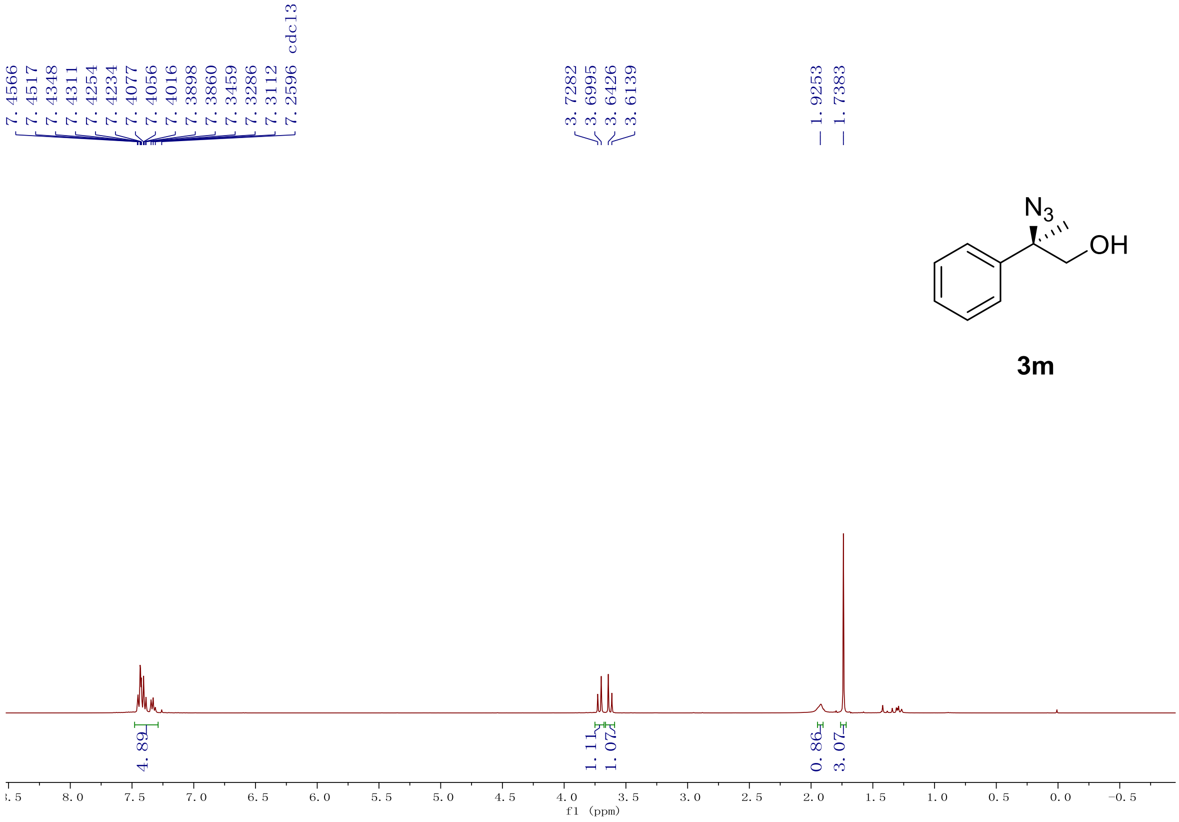
7.4566
7.4517
7.4348
7.4311
7.4254
7.4234
7.4077
7.4056
7.4016
7.3898
7.3860
7.3459
7.3286
7.3112
7.2596 cdcl3
3.7282
3.6995
3.6426
3.6139
1.9253
1.7383
N_3
OH
3m
4.89
1.11
1.07
0.86
3.07
f1 (ppm)

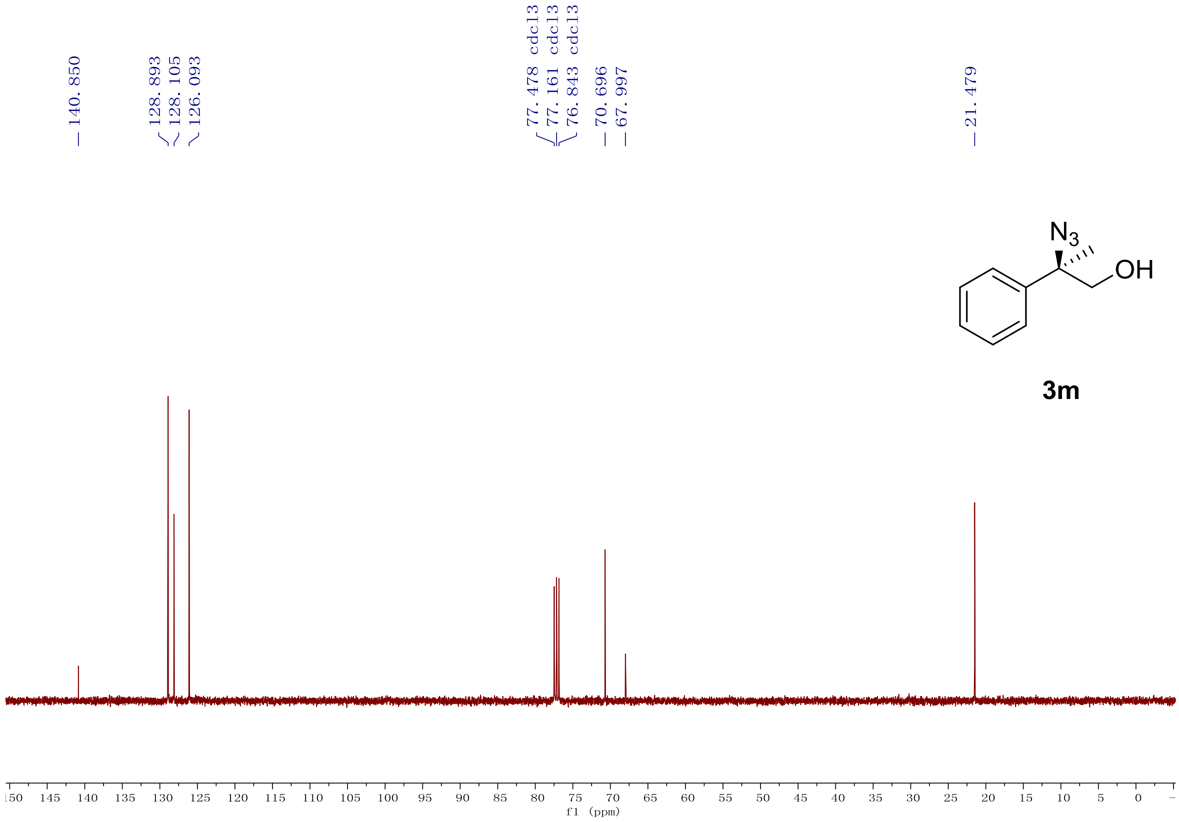
140.850
128.893
128.105
126.093
77.478 cdcl3
77.161 cdcl3
76.843 cdcl3
70.696
67.997
21.479
N_3
OH
3m
f1 (ppm)

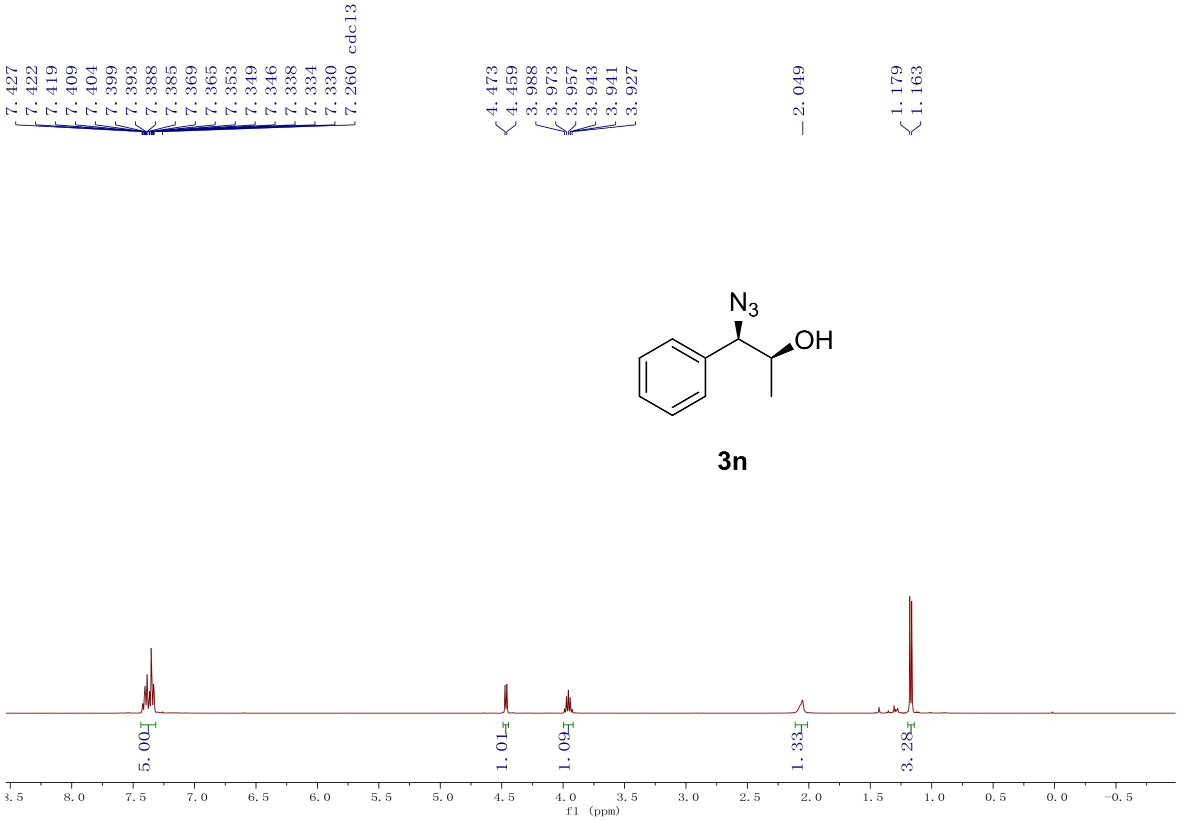
7.427
7.422
7.419
7.409
7.404
7.399
7.393
7.388
7.385
7.369
7.365
7.353
7.349
7.346
7.338
7.334
7.330
7.260 cdcl3
4.473
4.459
3.988
3.973
3.957
3.943
3.941
3.927
2.049
1.179
1.163
N3
OH
5.00
1.01
1.09
1.33
3.28
f1 (ppm)

3n

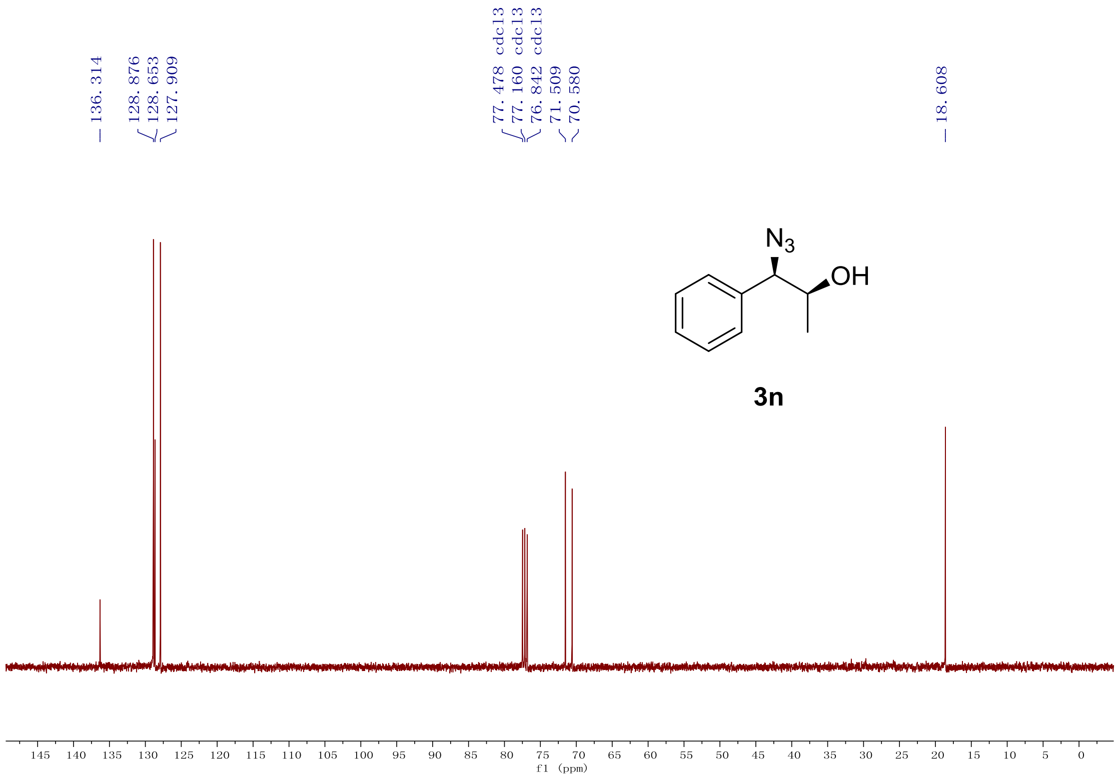
136.314
128.876
128.653
127.909
77.478 cdcl3
77.160 cdcl3
76.842 cdcl3
71.509
70.580
18.608
N3
OH
f1 (ppm)

3n

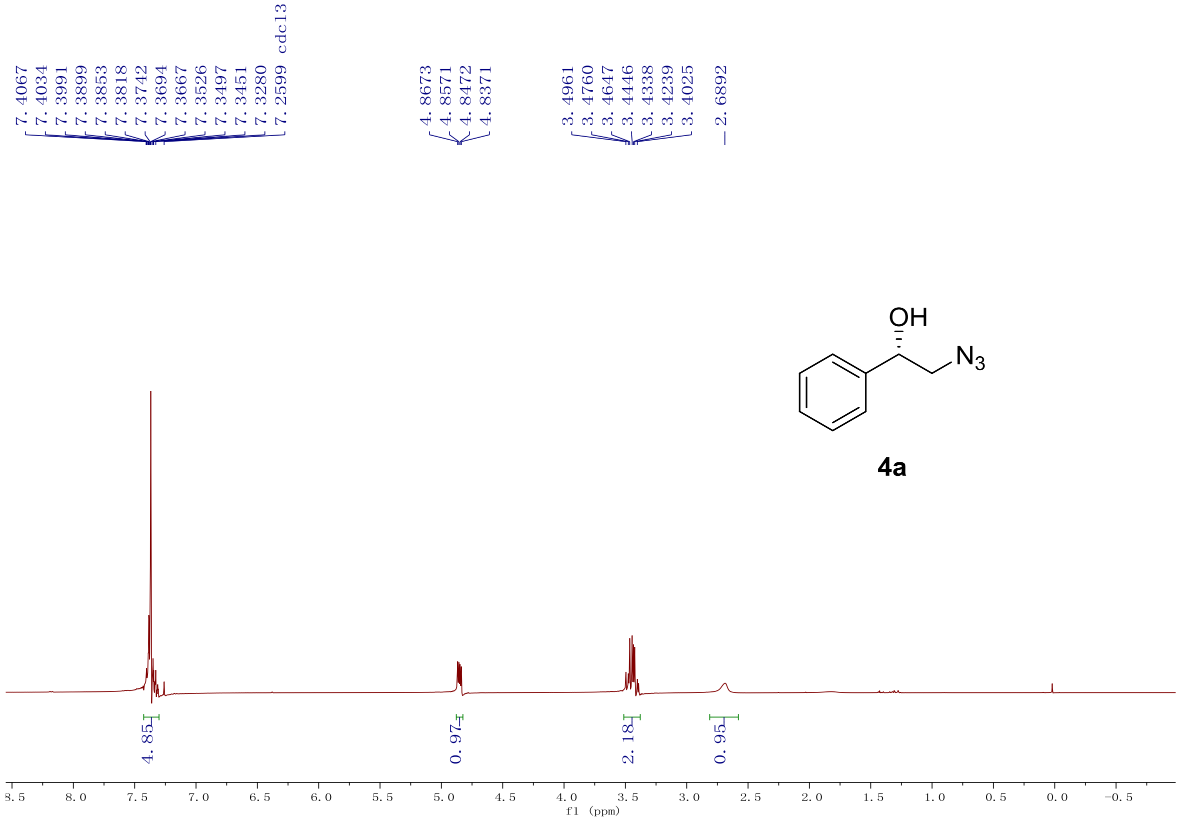
7.4067
7.4034
7.3991
7.3899
7.3853
7.3818
7.3742
7.3694
7.3667
7.3526
7.3497
7.3451
7.3280
7.2599 cdcl3
4.8673
4.8571
4.8472
4.8371
3.4961
3.4760
3.4647
3.4446
3.4338
3.4239
3.4025
2.6892
OH
N3
4a
4.85
0.97
2.18
0.95
8.5 8.0 7.5 7.0 6.5 6.0 5.5 5.0 4.5 4.0 3.5 3.0 2.5 2.0 1.5 1.0 0.5 0.0 -0.5
f1 (ppm)

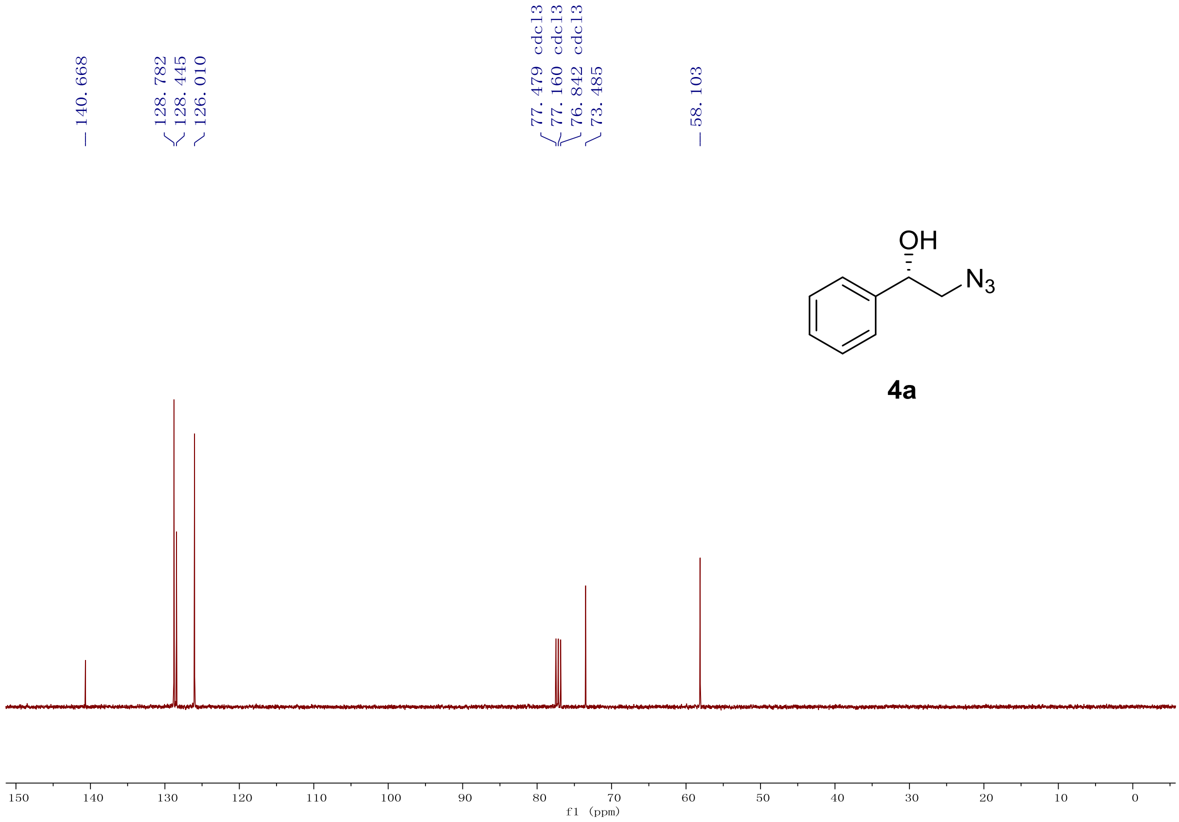
140.668
128.782
128.445
126.010
77.479 cdcl3
77.160 cdcl3
76.842 cdcl3
73.485
58.103
OH
N3
4a
150 140 130 120 110 100 90 80 70 60 50 40 30 20 10 0
f1 (ppm)

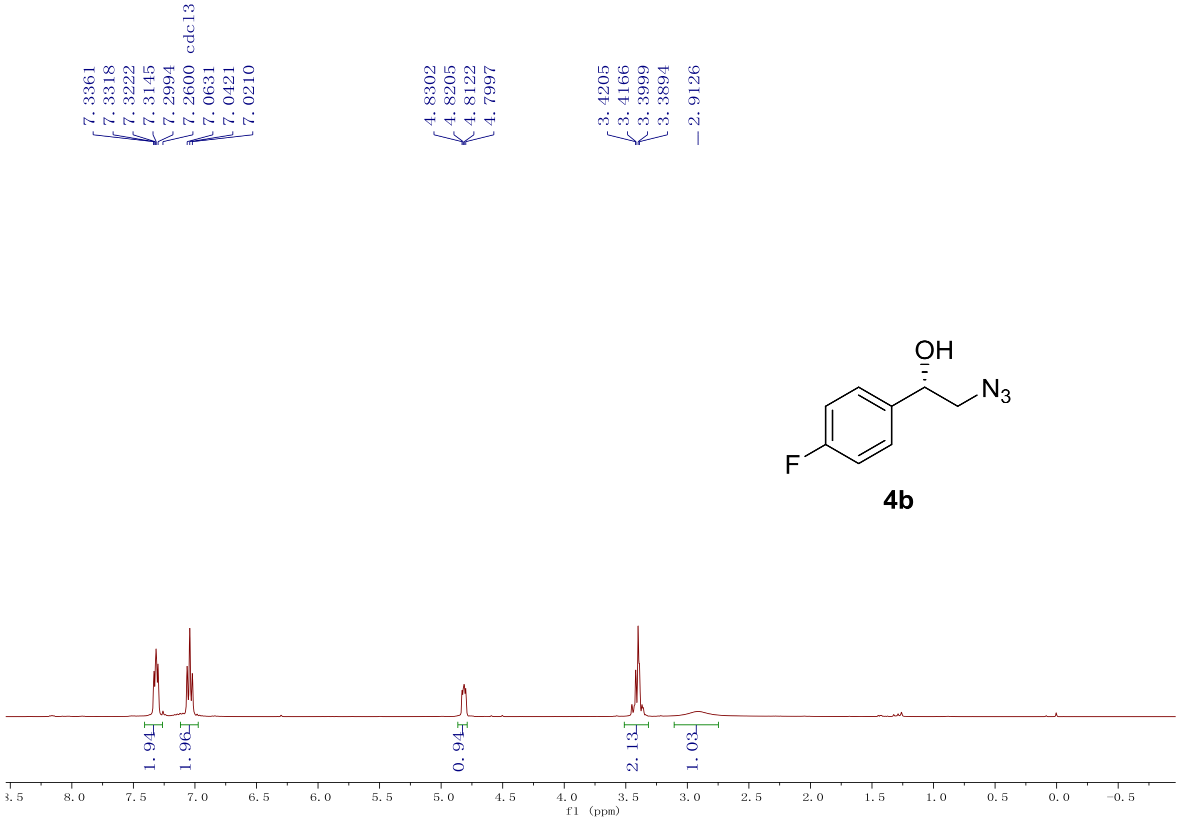
7.3361
7.3318
7.3222
7.3145
7.2994
7.2600 cdcl3
7.0631
7.0421
7.0210
4.8302
4.8205
4.8122
4.7997
3.4205
3.4166
3.3999
3.3894
2.9126
OH
N3
F
4b
1.94
1.96
0.94
2.13
1.03
f1 (ppm)

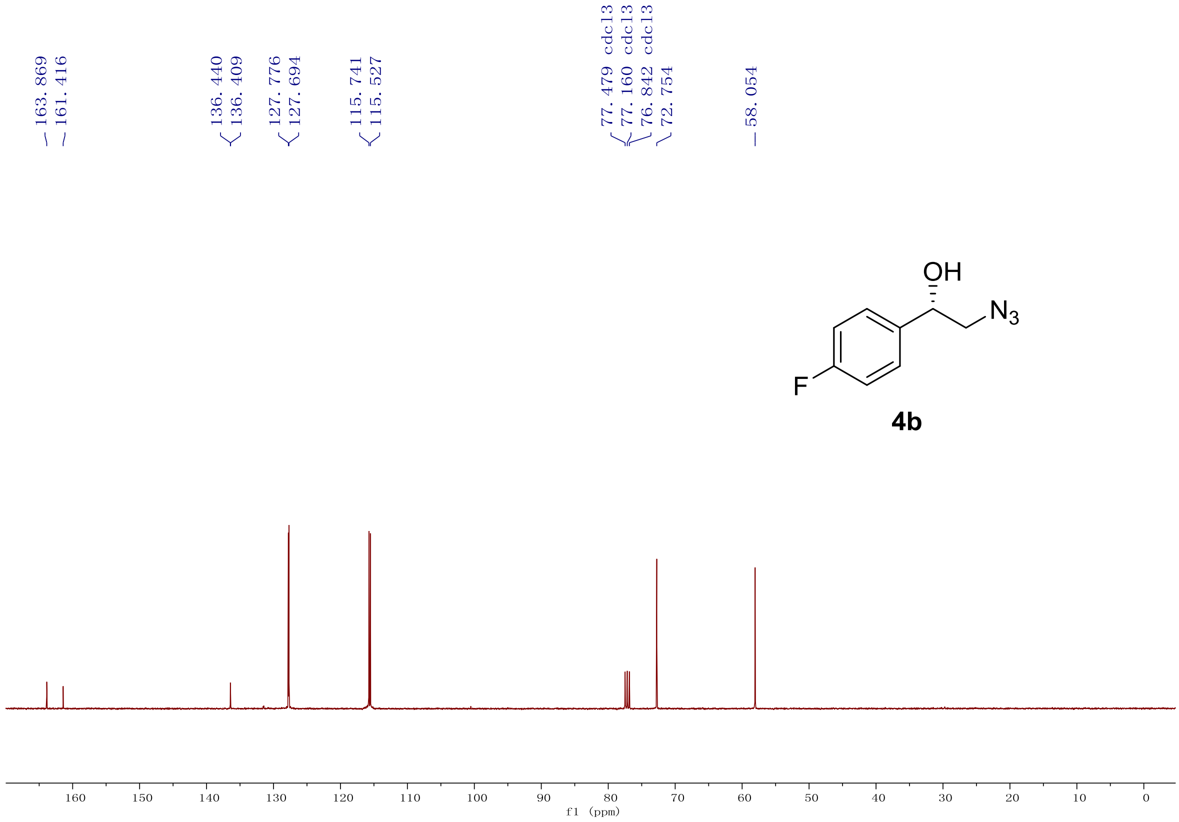
163.869
161.416
136.440
136.409
127.776
127.694
115.741
115.527
77.479 cdcl3
77.160 cdcl3
76.842 cdcl3
72.754
58.054
OH
N3
F
4b
f1 (ppm)

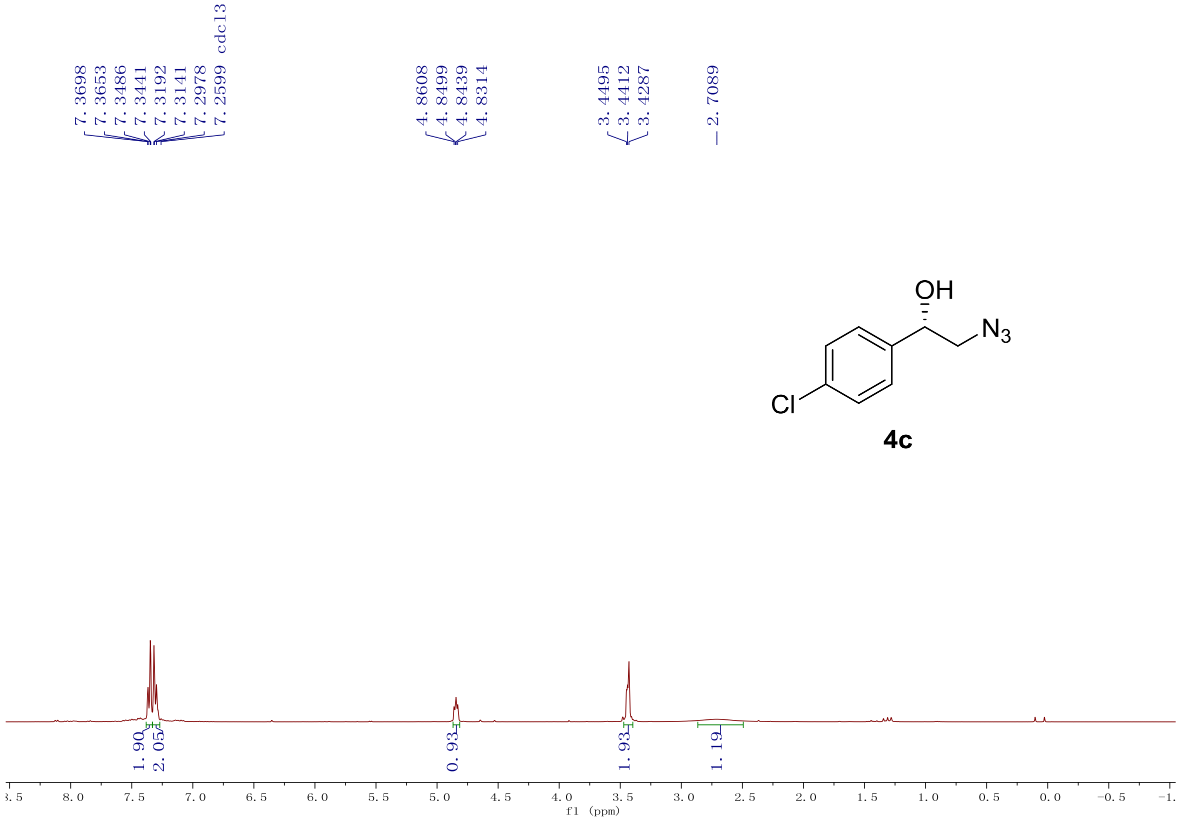

7.3698
7.3653
7.3486
7.3441
7.3192
7.3141
7.2978
7.2599 cdcl3
4.8608
4.8499
4.8439
4.8314
3.4495
3.4412
3.4287
2.7089
OH
N3
Cl
4c
1.90
2.05
0.93
1.93
1.19
f1 (ppm)

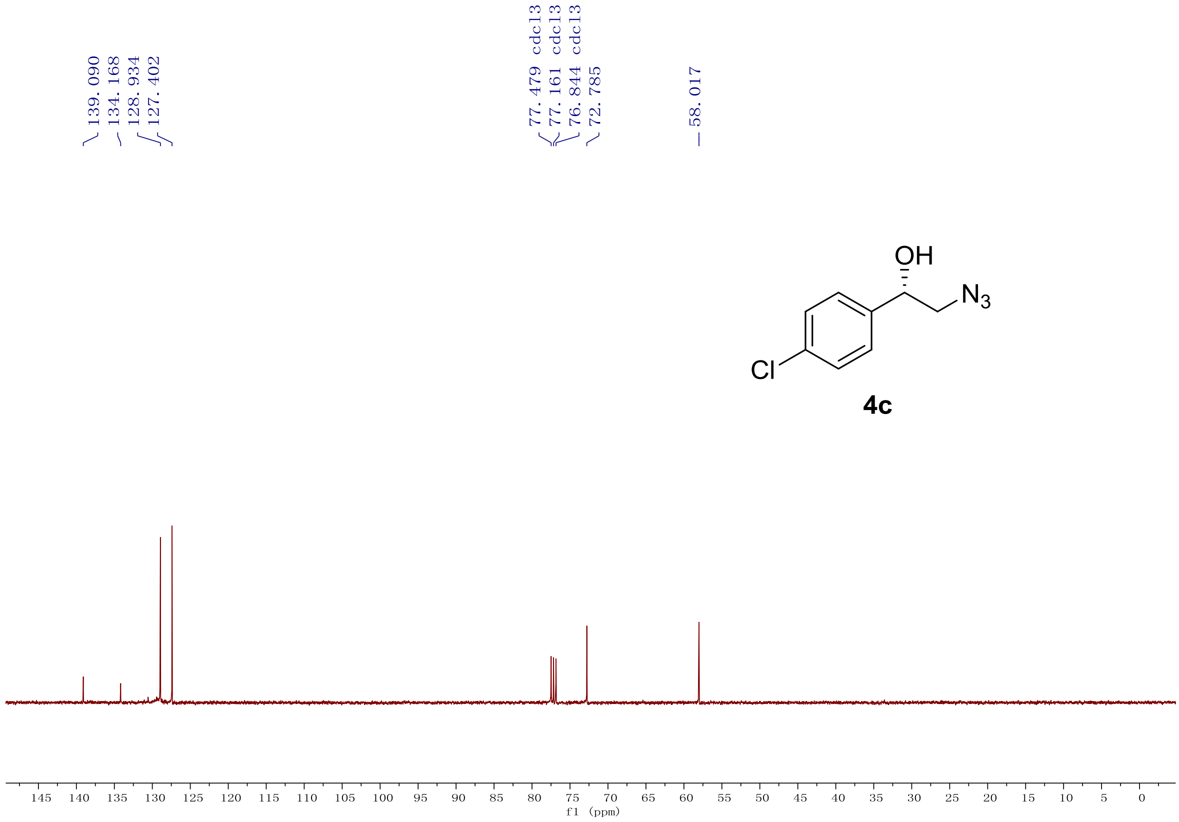

139.090
134.168
128.934
127.402
77.479 cdcl3
77.161 cdcl3
76.844 cdcl3
72.785
58.017
OH
N3
Cl
4c
f1 (ppm)

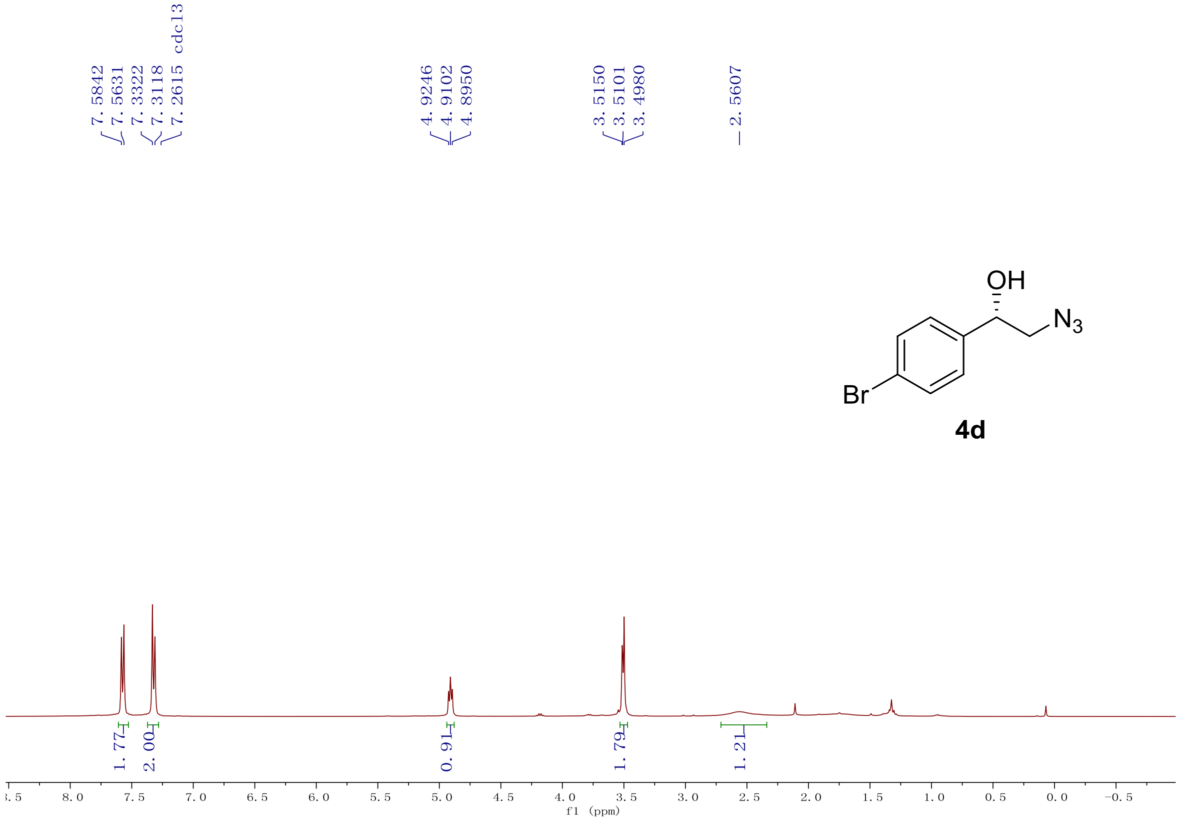
7.5842
7.5631
7.3322
7.3118
7.2615 cdcl3
4.9246
4.9102
4.8950
3.5150
3.5101
3.4980
2.5607
OH
N3
Br
4d
1.77
2.00
0.91
1.79
1.21
f1 (ppm)

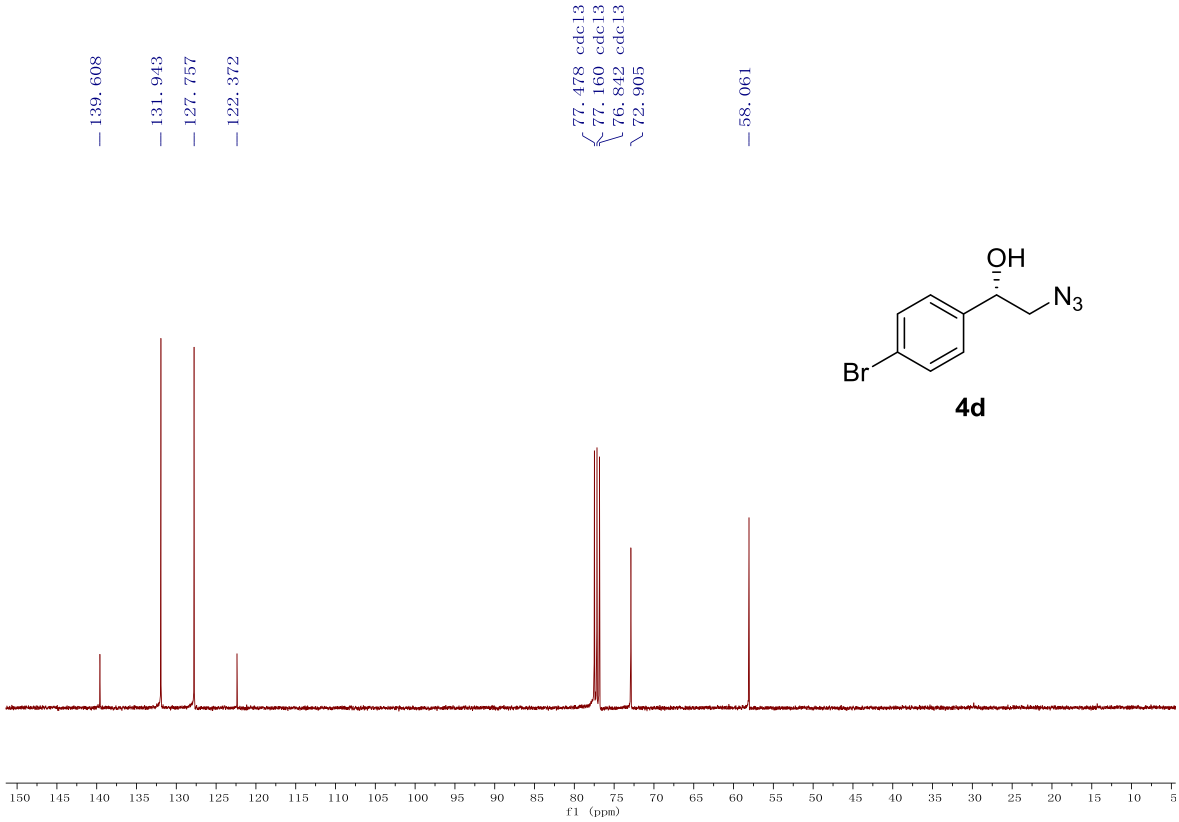
139.608
131.943
127.757
122.372
77.478 cdcl3
77.160 cdcl3
76.842 cdcl3
72.905
58.061
OH
N3
Br
4d
f1 (ppm)

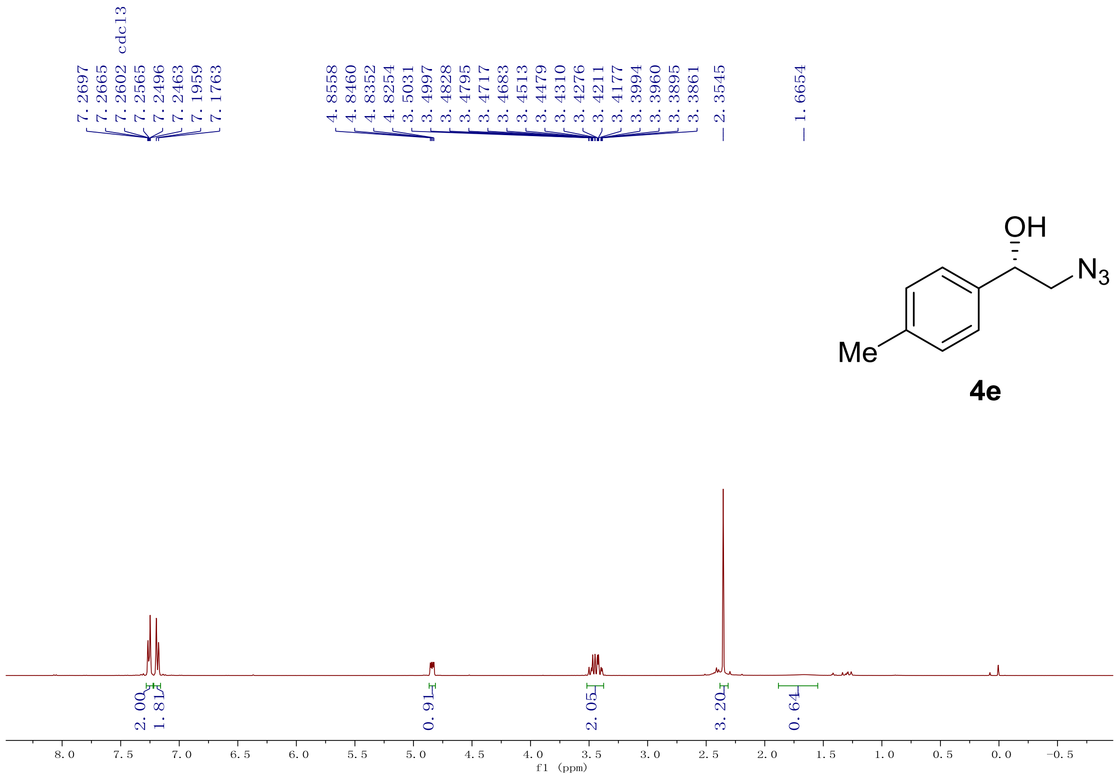
7.2697
7.2665
7.2602 cdcl3
7.2565
7.2496
7.2463
7.1959
7.1763
4.8558
4.8460
4.8352
4.8254
3.5031
3.4997
3.4828
3.4795
3.4717
3.4683
3.4513
3.4479
3.4310
3.4276
3.4211
3.4177
3.3994
3.3960
3.3895
3.3861
2.3545
1.6654
OH
N_3
Me
4e
2.00
1.81
0.91
2.05
3.20
0.64
f1 (ppm)

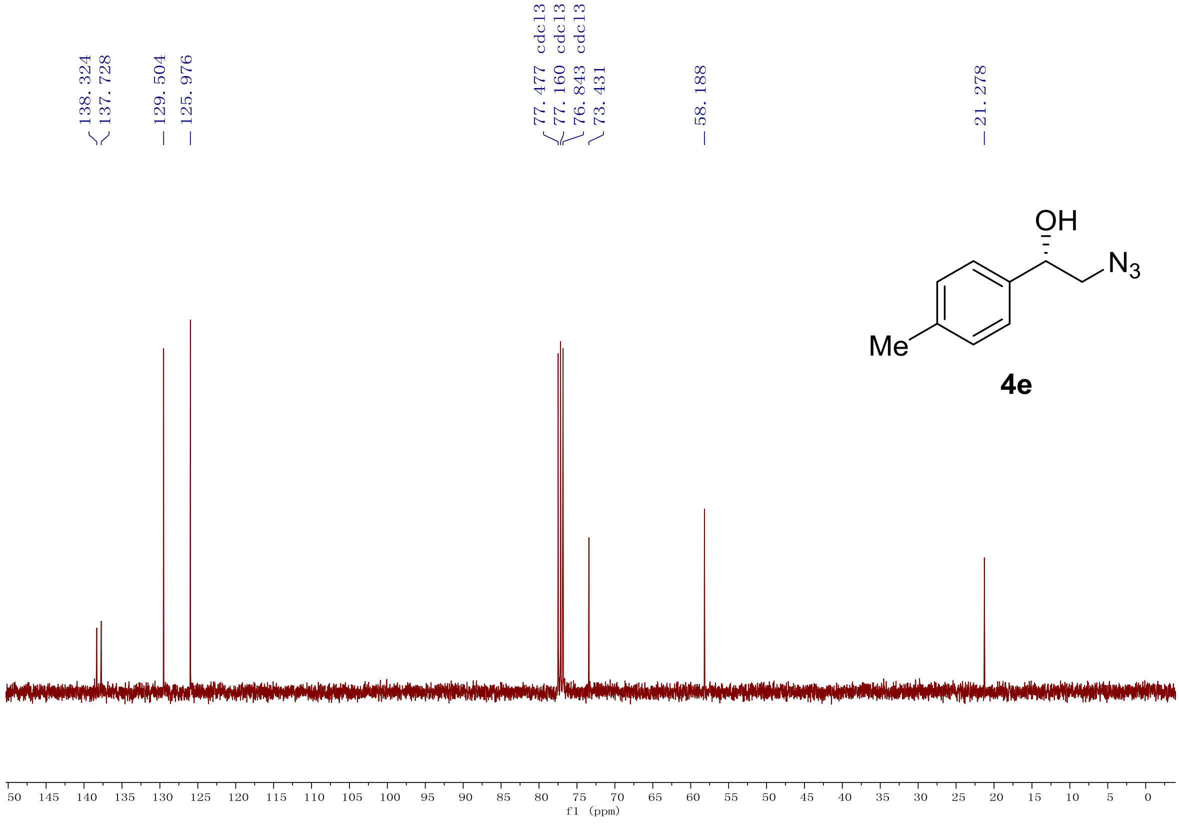
138.324
137.728
129.504
125.976
77.477 cdcl3
77.160 cdcl3
76.843 cdcl3
73.431
58.188
21.278
OH
N_3
Me
4e
f1 (ppm)

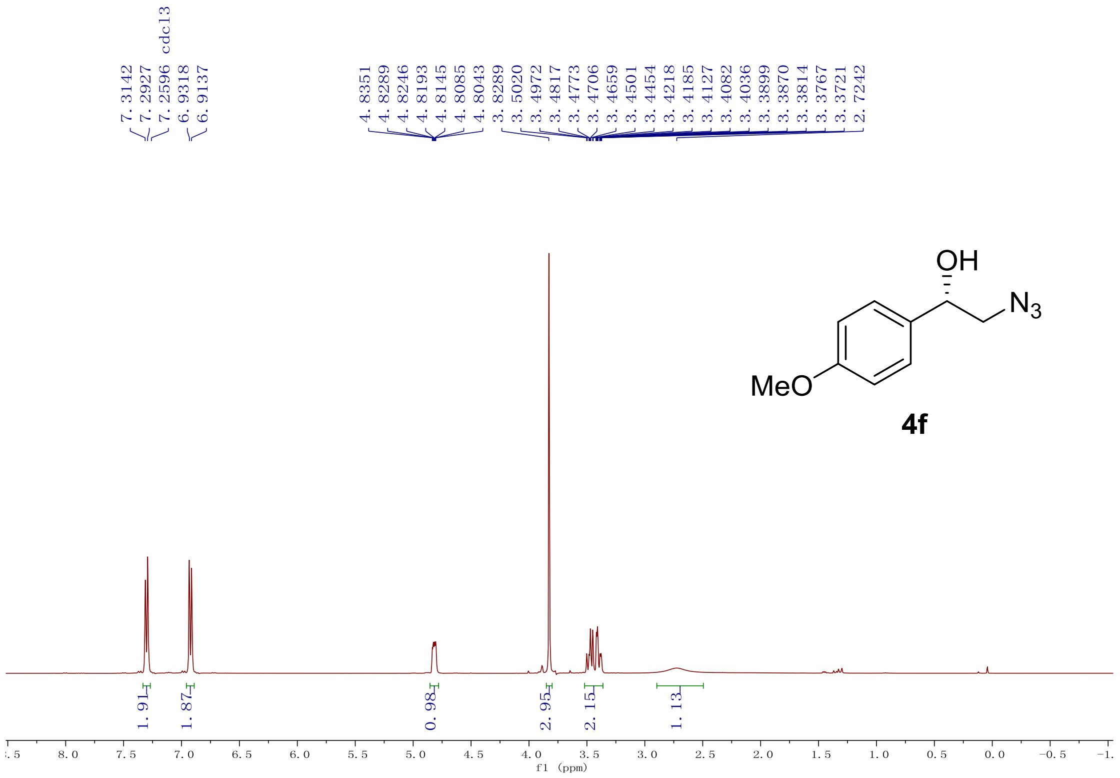
7.3142
7.2927
7.2596 cdcl3
6.9318
6.9137
4.8351
4.8289
4.8246
4.8193
4.8145
4.8085
4.8043
3.8289
3.5020
3.4972
3.4817
3.4773
3.4706
3.4659
3.4501
3.4454
3.4218
3.4185
3.4127
3.4082
3.4036
3.3899
3.3870
3.3814
3.3767
3.3721
2.7242
OH
N3
MeO
4f
1.91
1.87
0.98
2.95
2.15
1.13
f1 (ppm)

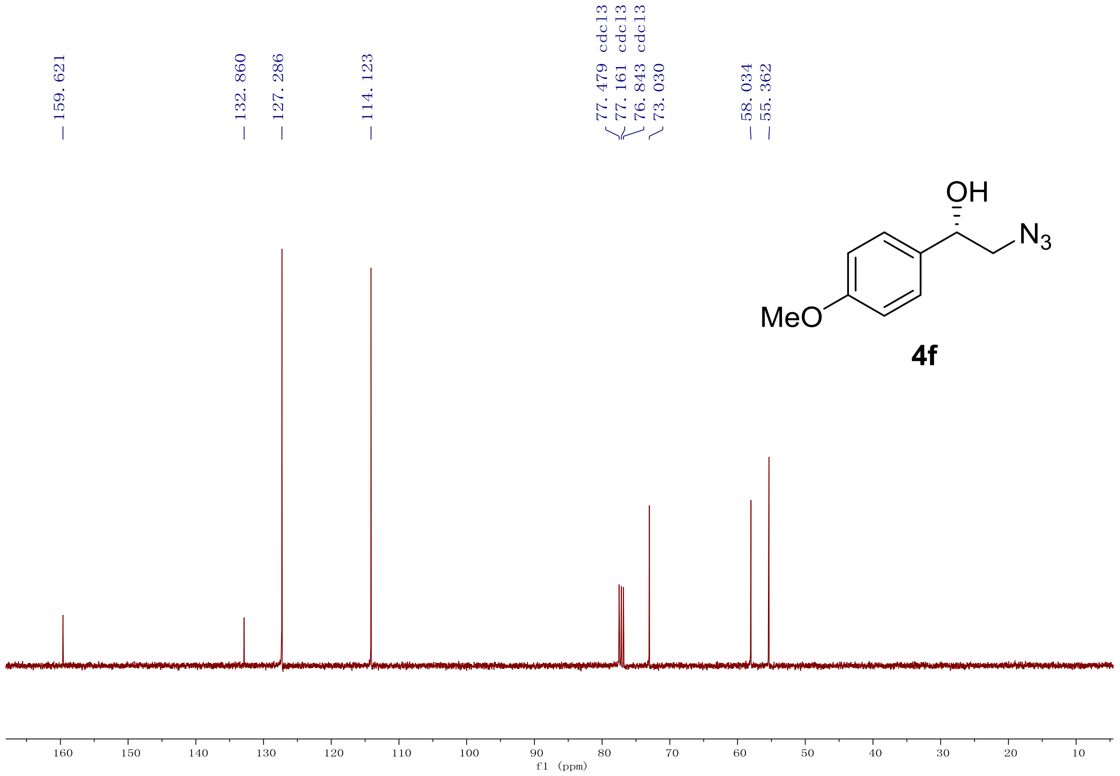
159.621
132.860
127.286
114.123
77.479 cdcl3
77.161 cdcl3
76.843 cdcl3
73.030
58.034
55.362
OH
N3
MeO
4f
f1 (ppm)

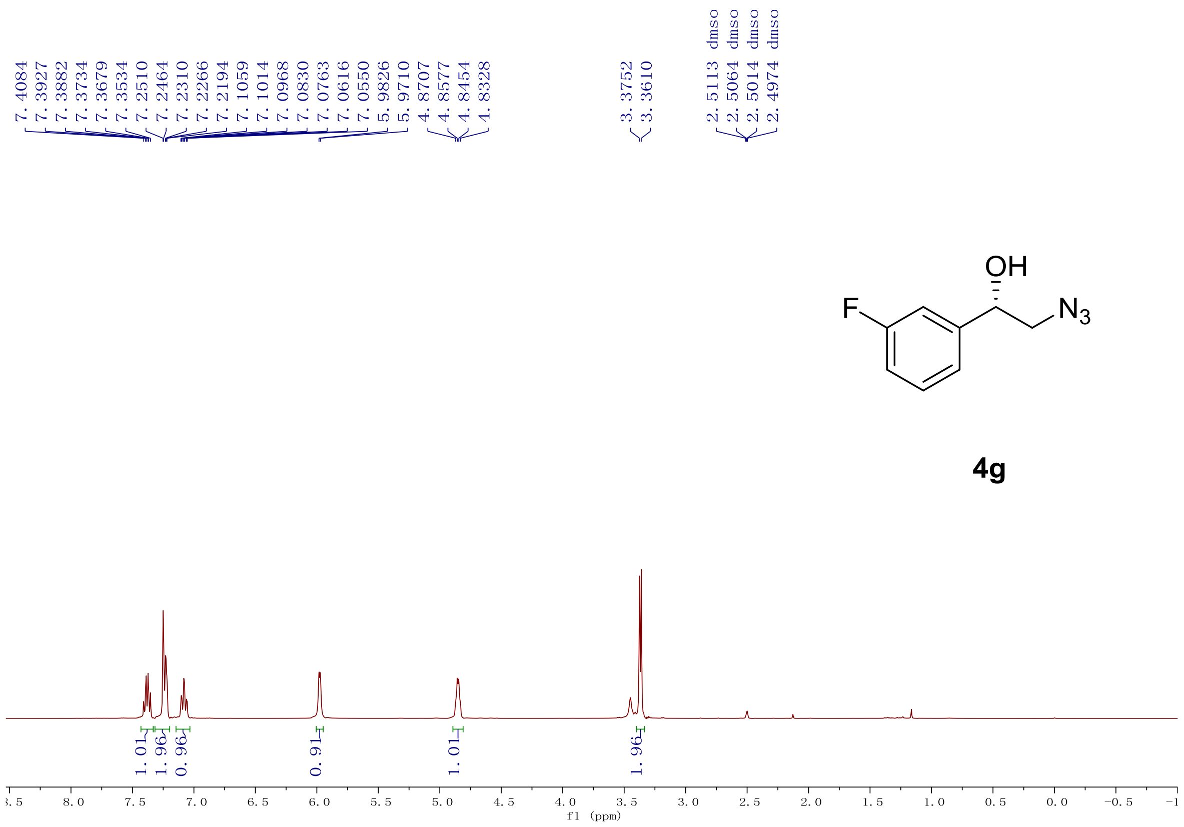
7.4084
7.3927
7.3882
7.3734
7.3679
7.3534
7.2510
7.2464
7.2310
7.2266
7.2194
7.1059
7.1014
7.0968
7.0830
7.0763
7.0616
7.0550
5.9826
5.9710
4.8707
4.8577
4.8454
4.8328
3.3752
3.3610
2.5113 dmso
2.5064 dmso
2.5014 dmso
2.4974 dmso
OH
F
N3
1.01
1.96
0.96
0.91
1.01
1.96
f1 (ppm)

4g

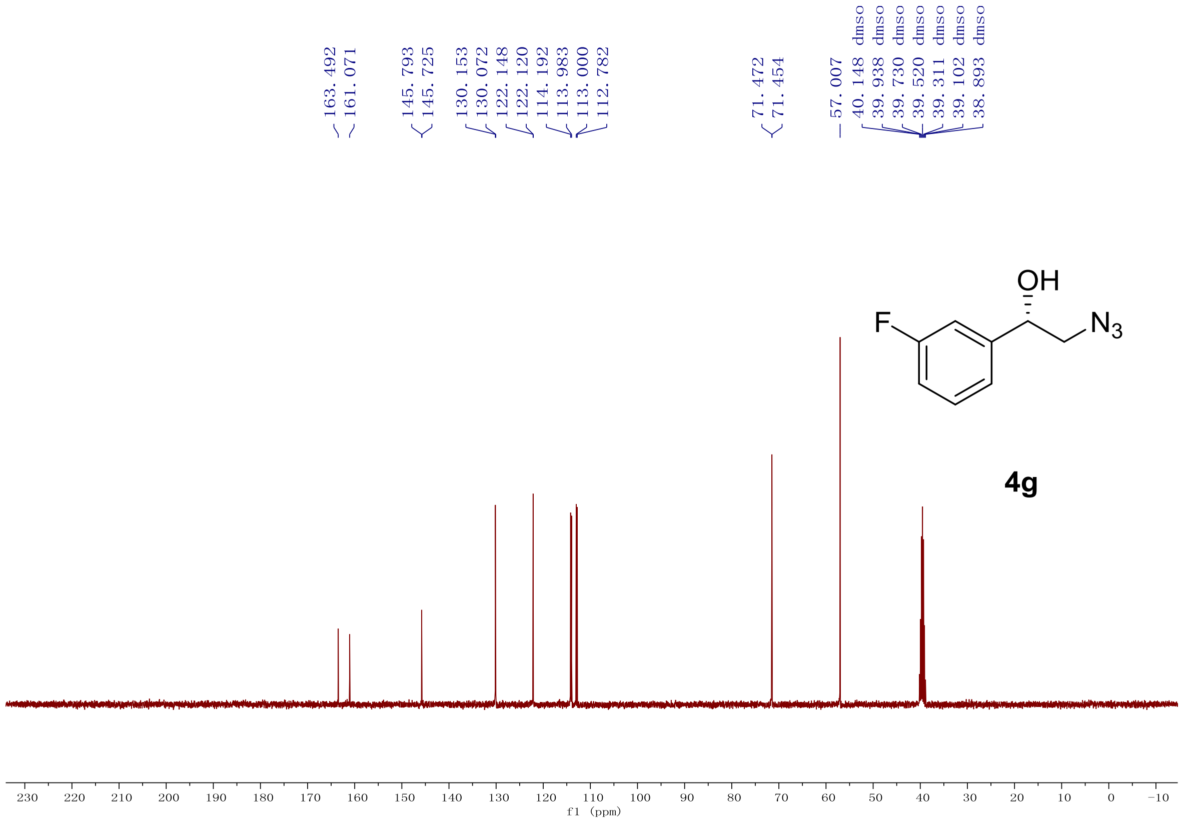
163.492
161.071
145.793
145.725
130.153
130.072
122.148
122.120
114.192
113.983
113.000
112.782
71.472
71.454
57.007
40.148 dmso
39.938 dmso
39.730 dmso
39.520 dmso
39.311 dmso
39.102 dmso
38.893 dmso
OH
F
N3
f1 (ppm)

4g

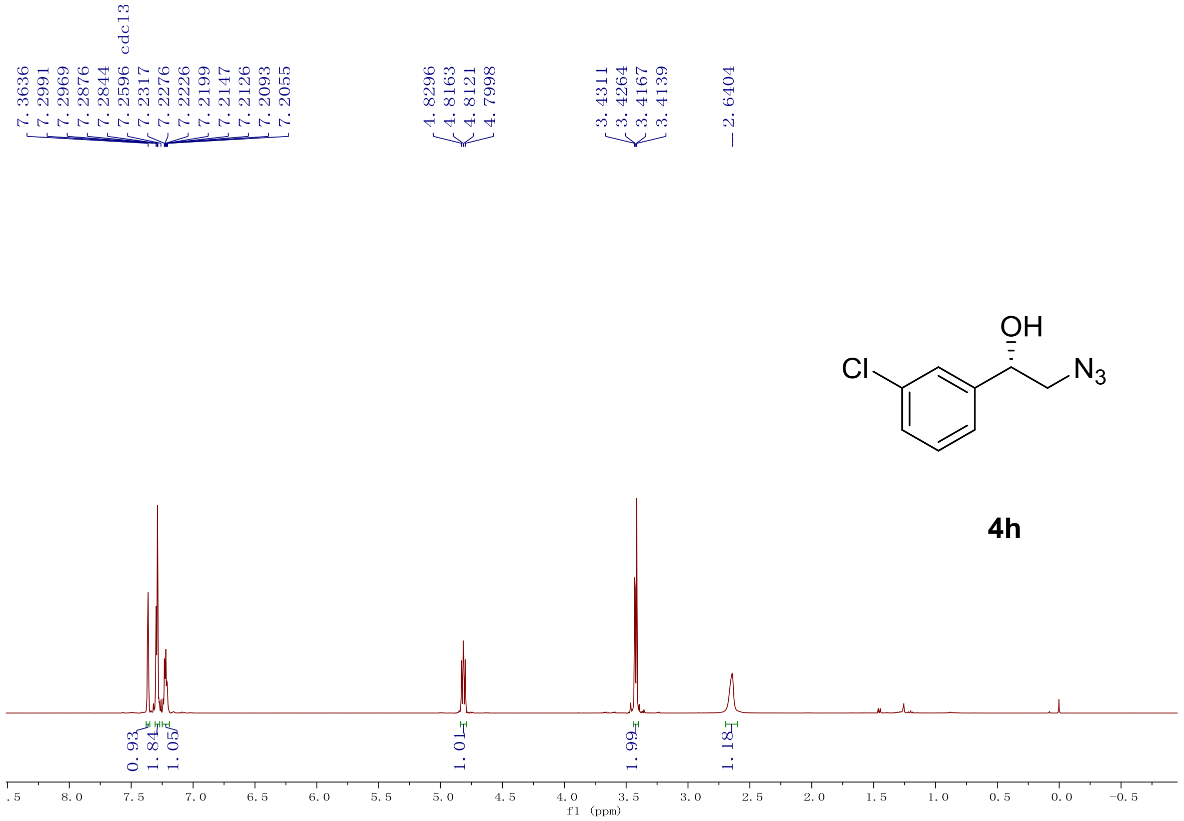
7.3636
7.2991
7.2969
7.2876
7.2844
7.2596 cdcl3
7.2317
7.2276
7.2226
7.2199
7.2147
7.2126
7.2093
7.2055
4.8296
4.8163
4.8121
4.7998
3.4311
3.4264
3.4167
3.4139
2.6404
OH
Cl
N3
4h
0.93
1.84
1.05
1.01
1.99
1.18
f1 (ppm)

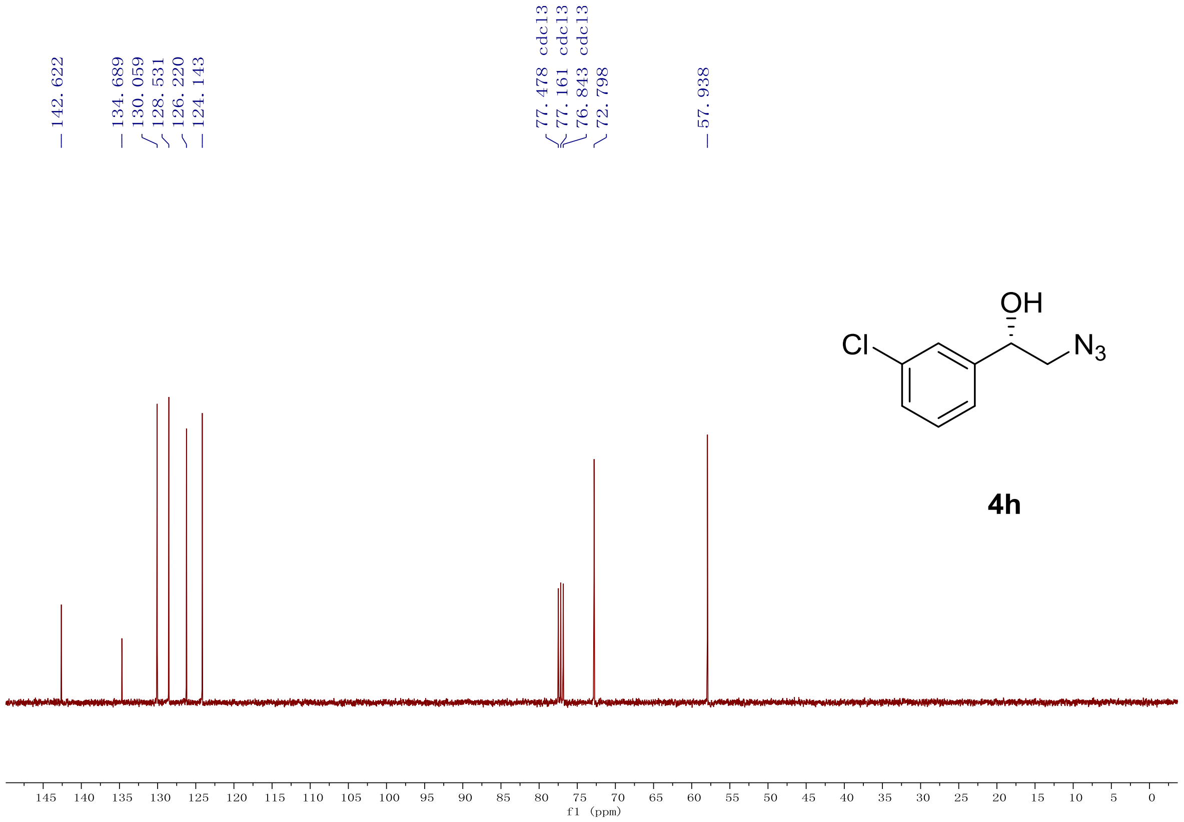
142.622
134.689
130.059
128.531
126.220
124.143
77.478 cdcl3
77.161 cdcl3
76.843 cdcl3
72.798
57.938
OH
Cl
N3
4h
f1 (ppm)

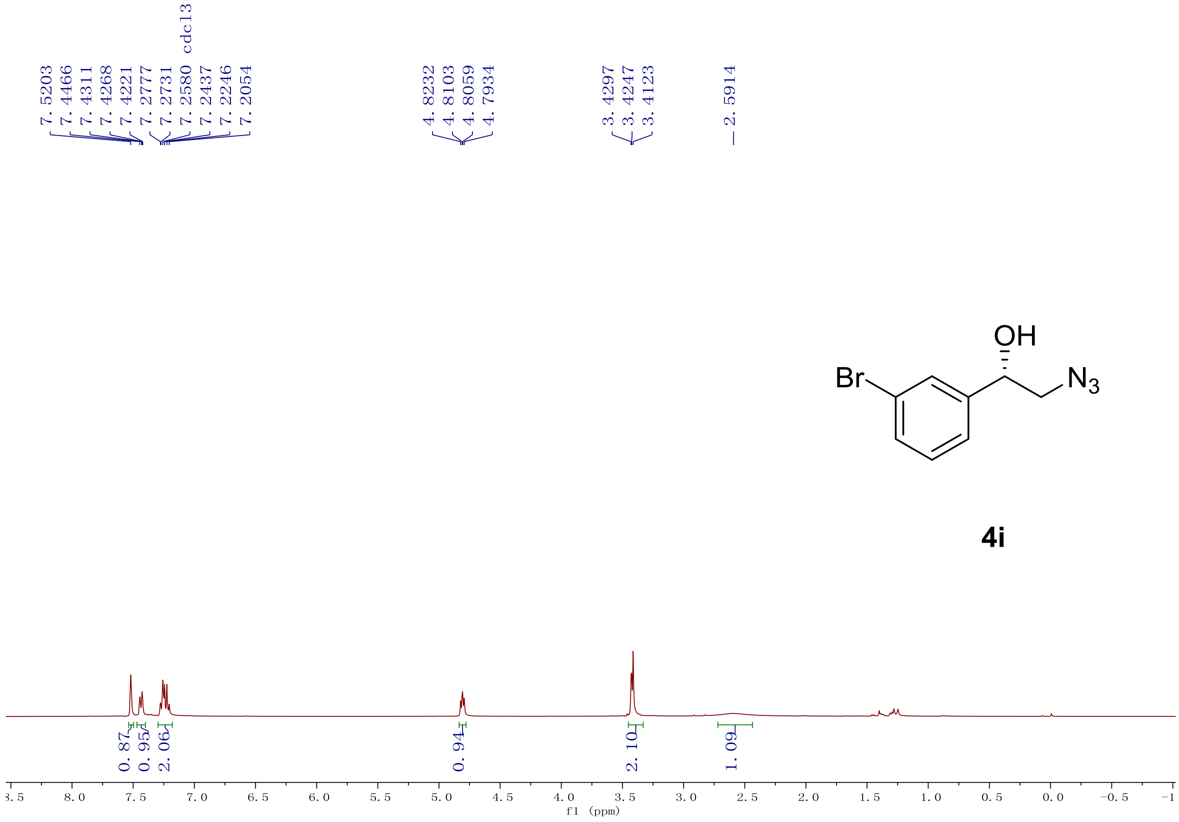
7.5203
7.4466
7.4311
7.4268
7.4221
7.2777
7.2731
7.2580 cdcl3
7.2437
7.2246
7.2054
4.8232
4.8103
4.8059
4.7934
3.4297
3.4247
3.4123
2.5914
0.87
0.95
2.06
0.94
2.10
1.09
8.5 8.0 7.5 7.0 6.5 6.0 5.5 5.0 4.5 4.0 3.5 3.0 2.5 2.0 1.5 1.0 0.5 0.0 -0.5 -1
f1 (ppm)
OH
Br
N3

4i

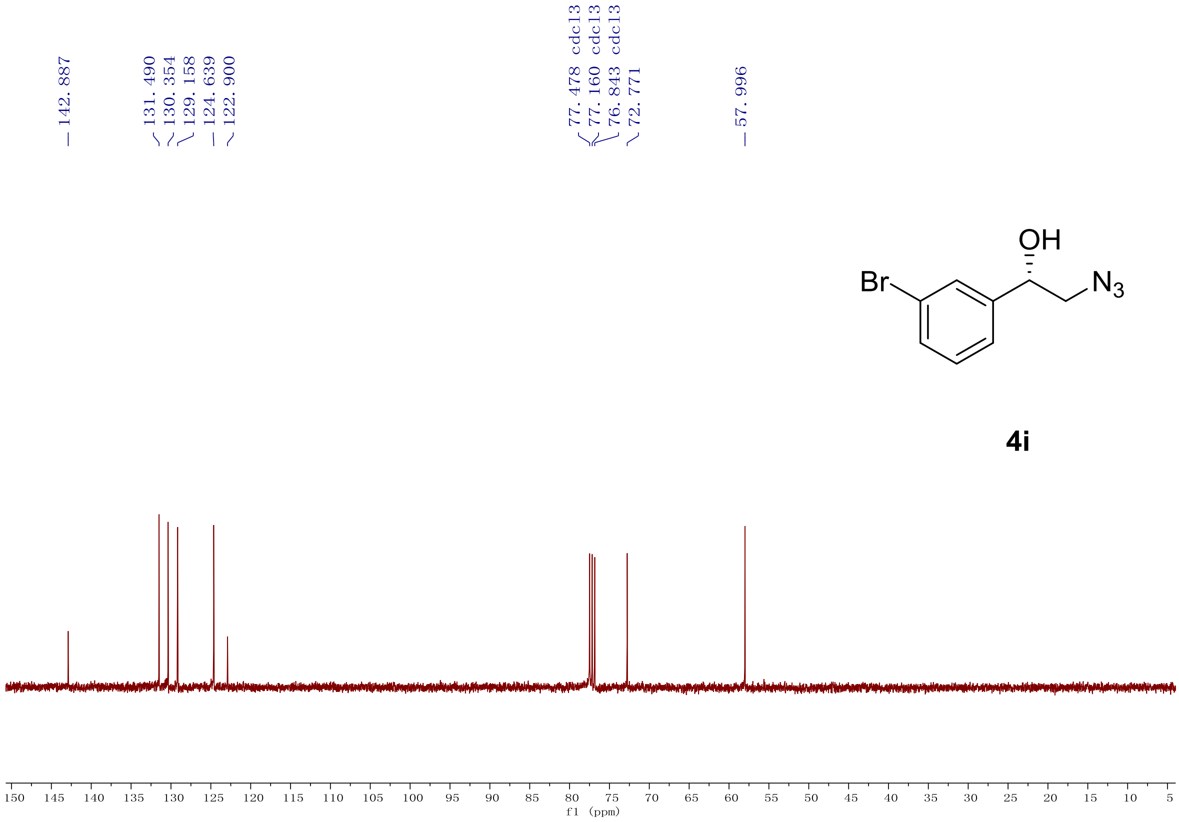
142.887
131.490
130.354
129.158
124.639
122.900
77.478 cdcl3
77.160 cdcl3
76.843 cdcl3
72.771
57.996
150 145 140 135 130 125 120 115 110 105 100 95 90 85 80 75 70 65 60 55 50 45 40 35 30 25 20 15 10 5
f1 (ppm)
OH
Br
N3

4i

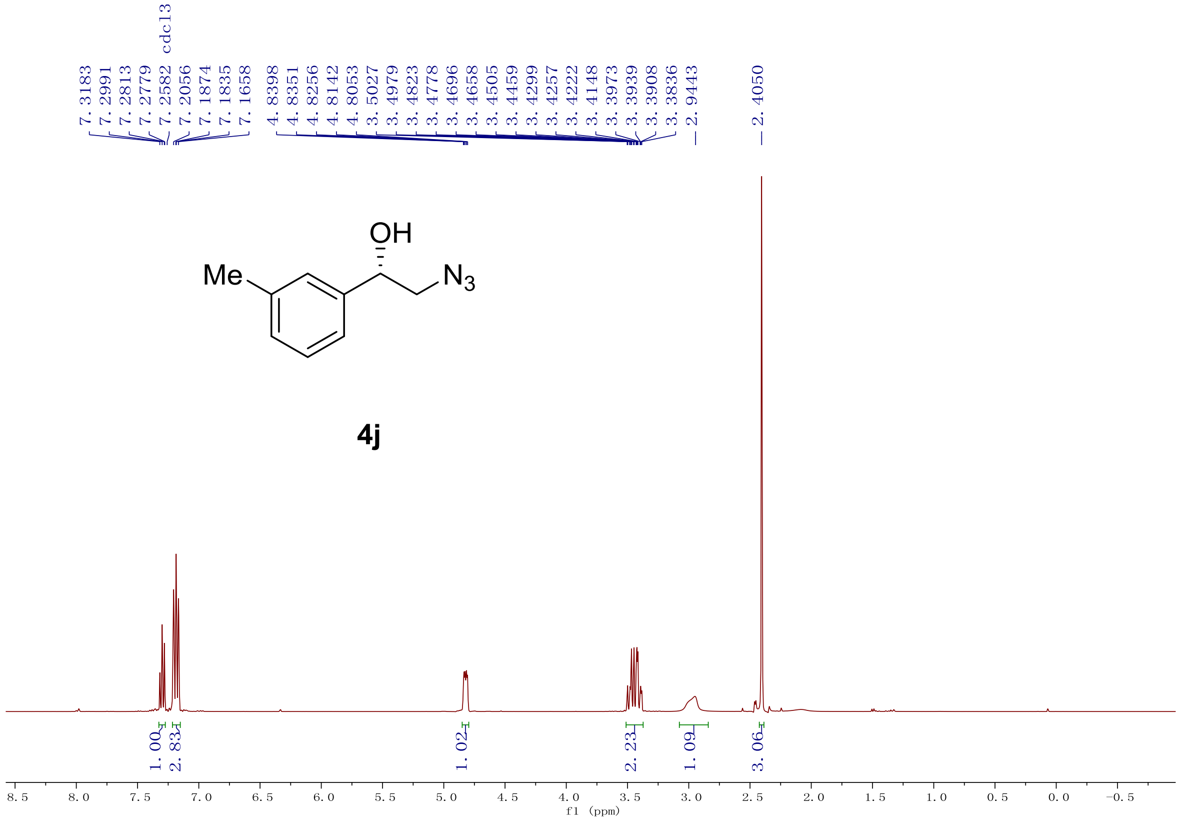
7.3183
7.2991
7.2813
7.2779
7.2582 cdcl3
7.2056
7.1874
7.1835
7.1658
4.8398
4.8351
4.8256
4.8142
4.8053
3.5027
3.4979
3.4823
3.4778
3.4696
3.4658
3.4505
3.4459
3.4299
3.4257
3.4222
3.4148
3.3973
3.3939
3.3908
3.3836
2.9443
2.4050
OH
Me
N3
4j
1.00
2.83
1.02
2.23
1.09
3.06
f1 (ppm)

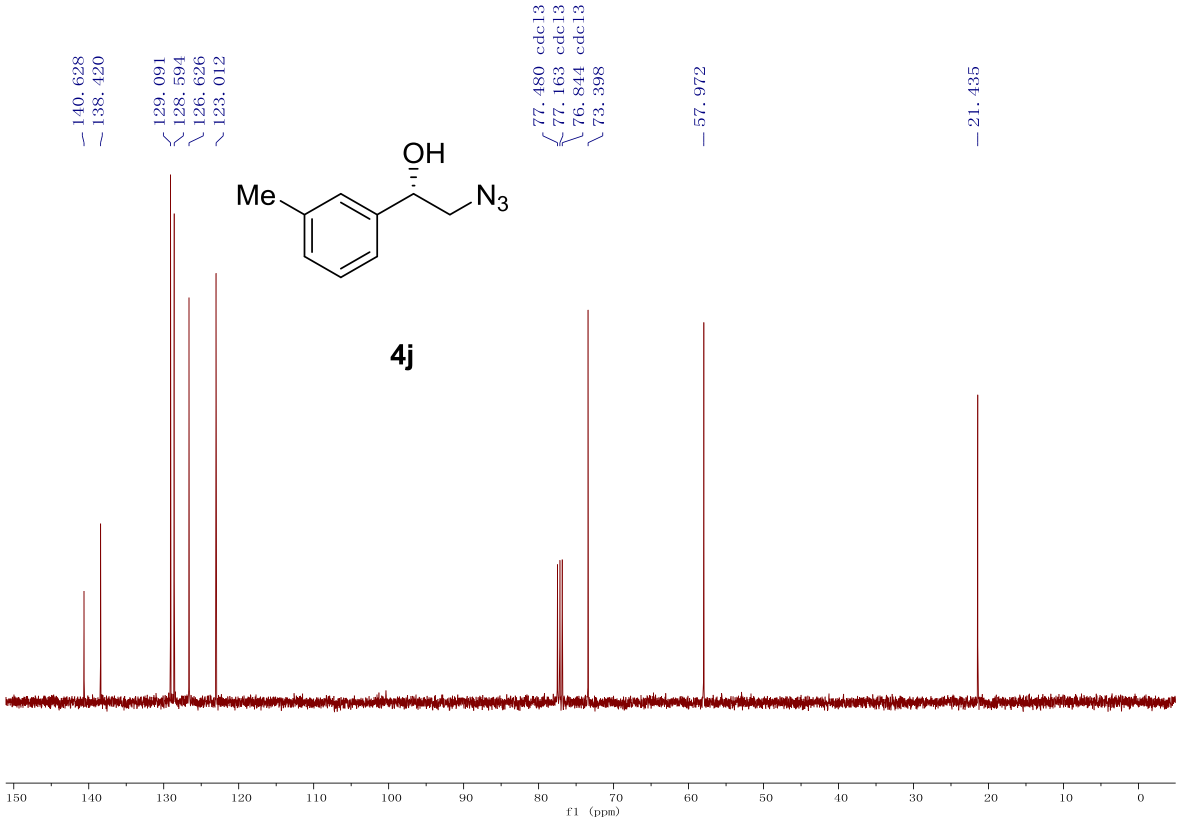
140.628
138.420
129.091
128.594
126.626
123.012
77.480 cdcl3
77.163 cdcl3
76.844 cdcl3
73.398
57.972
21.435
OH
Me
N3
4j
f1 (ppm)

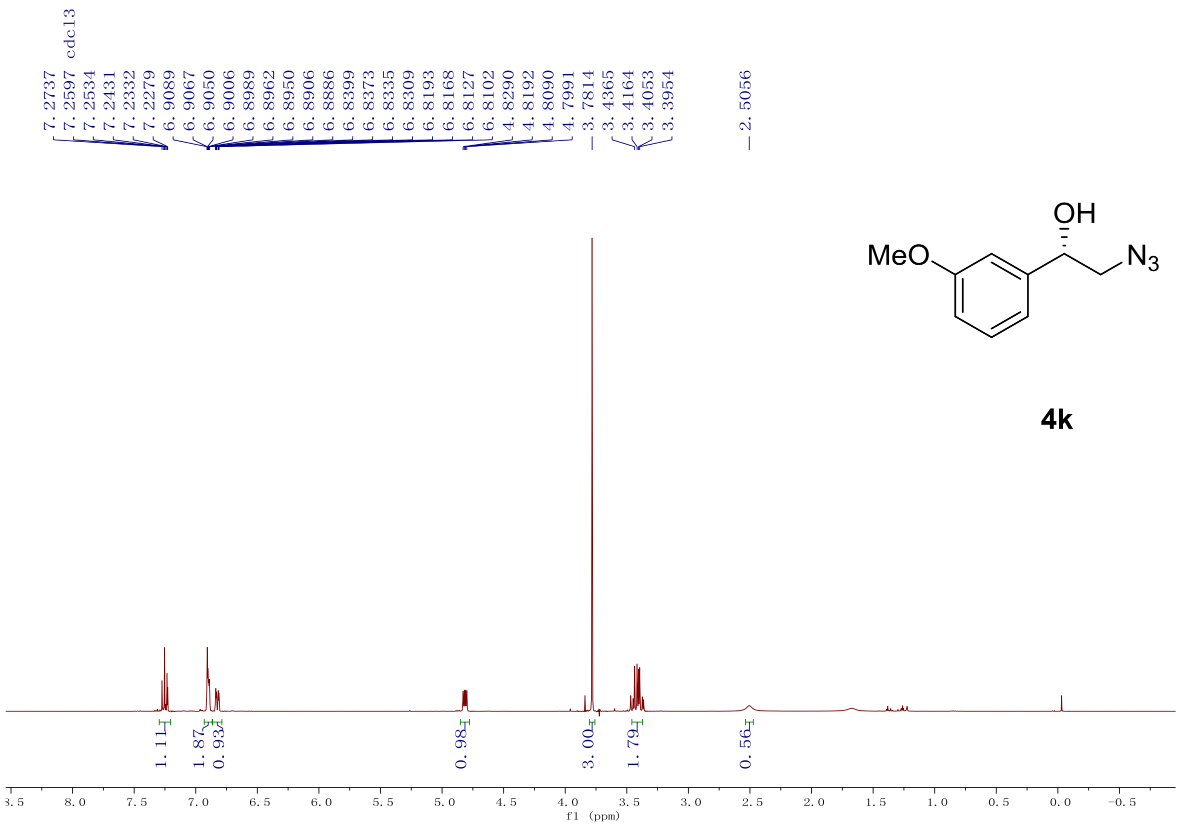
7.2737 cdcl3
7.2597
7.2534
7.2431
7.2332
7.2279
6.9089
6.9067
6.9050
6.9006
6.8989
6.8962
6.8950
6.8906
6.8886
6.8399
6.8373
6.8335
6.8309
6.8193
6.8168
6.8127
6.8102
4.8290
4.8192
4.8090
4.7991
3.7814
3.4365
3.4164
3.4053
3.3954
2.5056
MeO
OH
N3
1.11
1.87
0.93
0.98
3.00
1.79
0.56
f1 (ppm)

4k

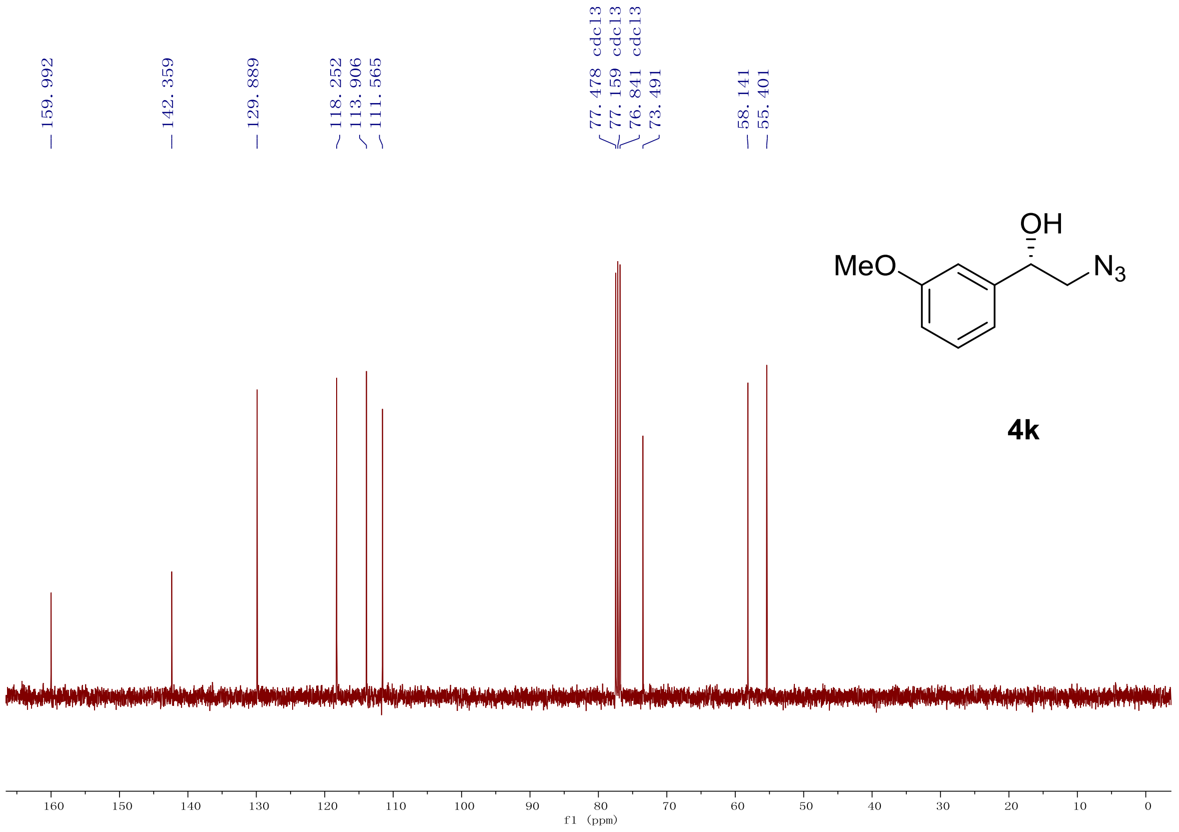
159.992
142.359
129.889
118.252
113.906
111.565
77.478 cdcl3
77.159 cdcl3
76.841 cdcl3
73.491
58.141
55.401
MeO
OH
N3
f1 (ppm)

4k

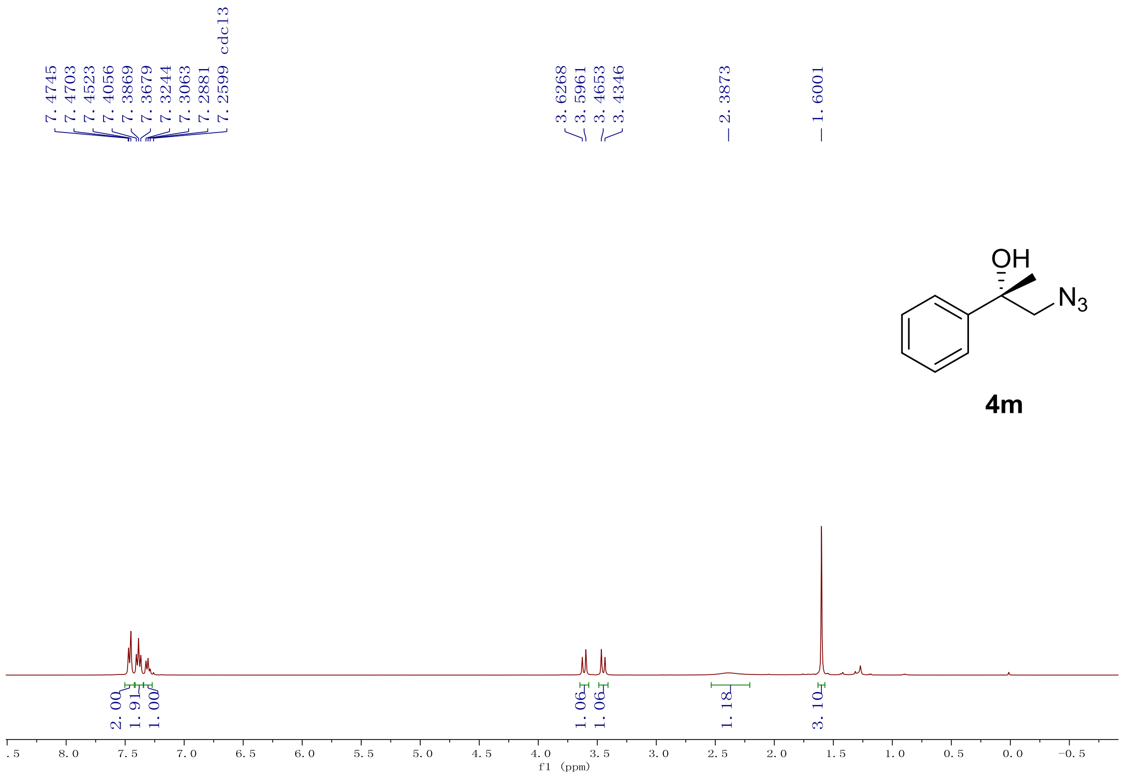
7.4745
7.4703
7.4523
7.4056
7.3869
7.3679
7.3244
7.3063
7.2881
7.2599 cdcl3
3.6268
3.5961
3.4653
3.4346
2.3873
1.6001
OH
N3
4m
2.00
1.91
1.00
1.06
1.06
1.18
3.10
f1 (ppm)

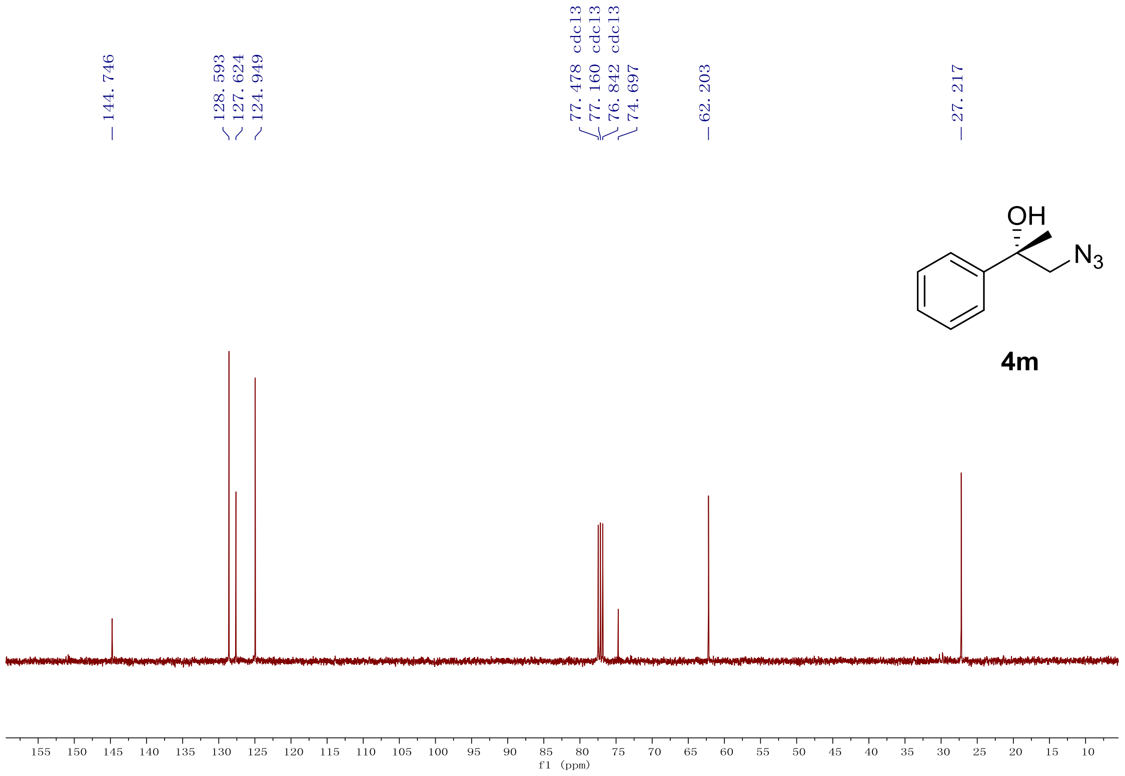
144.746
128.593
127.624
124.949
77.478 cdcl3
77.160 cdcl3
76.842 cdcl3
74.697
62.203
27.217
OH
N3
4m
f1 (ppm)

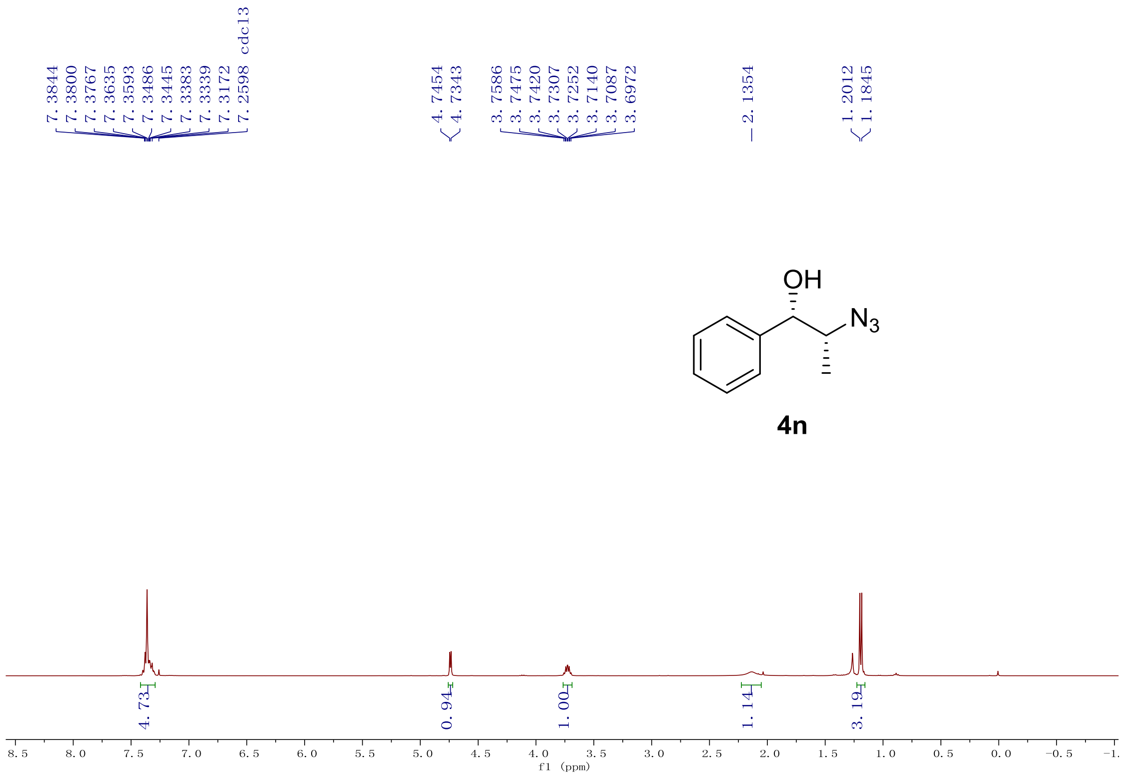
7.3844
7.3800
7.3767
7.3635
7.3593
7.3486
7.3445
7.3383
7.3339
7.3172
7.2598 cdcl3
4.7454
4.7343
3.7586
3.7475
3.7420
3.7307
3.7252
3.7140
3.7087
3.6972
2.1354
1.2012
1.1845
OH
N3
4n
4.73
0.94
1.00
1.14
3.19
f1 (ppm)

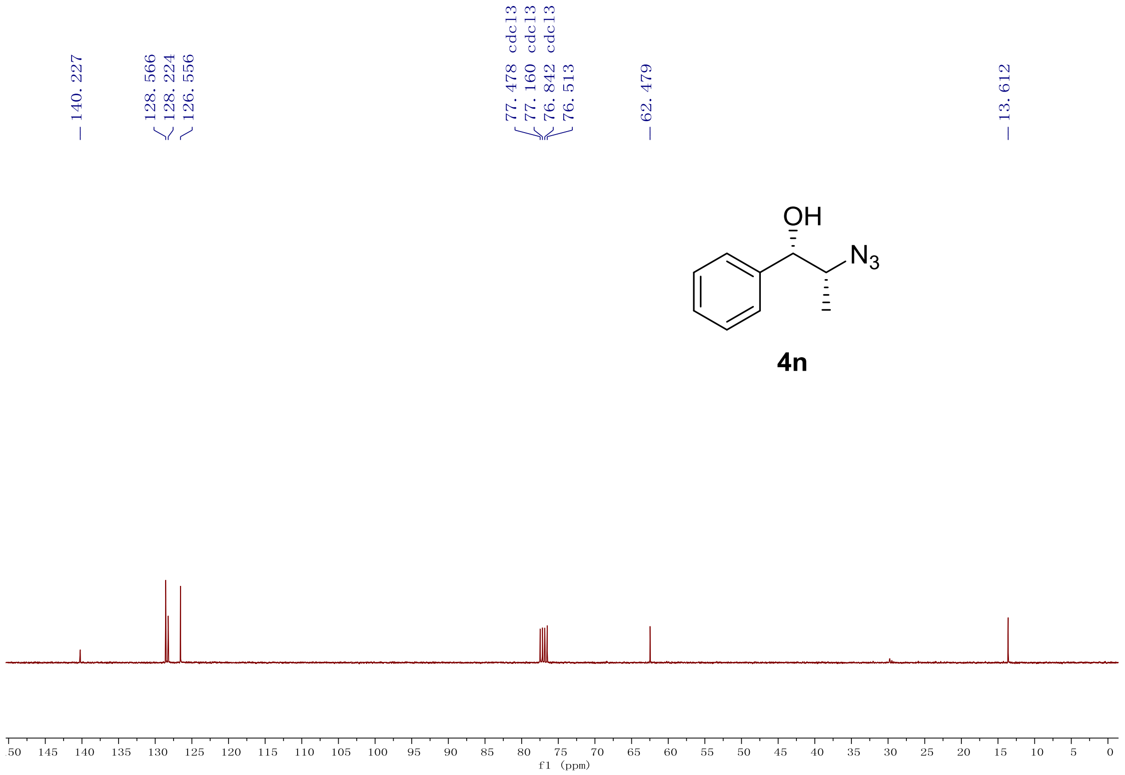
140.227
128.566
128.224
126.556
77.478 cdcl3
77.160 cdcl3
76.842 cdcl3
76.513
62.479
13.612
OH
N3
4n
f1 (ppm)

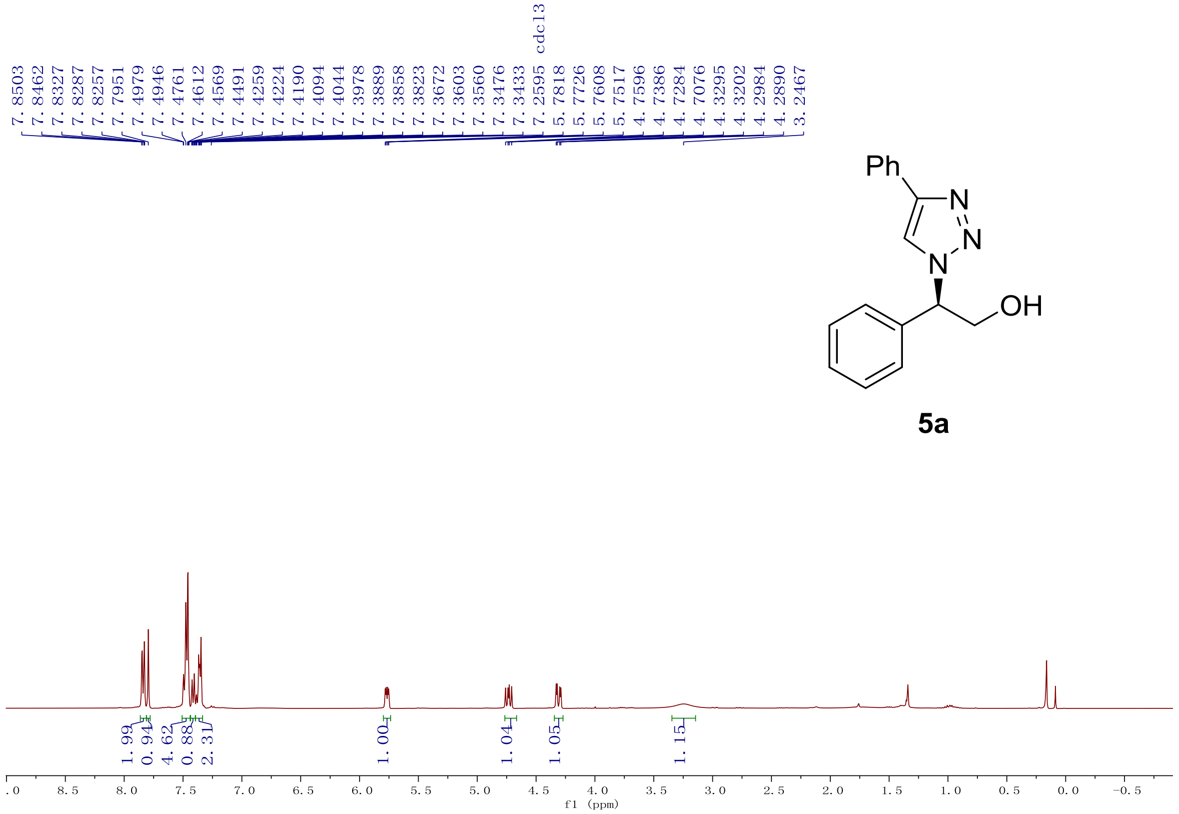
7.8503
7.8462
7.8327
7.8287
7.8257
7.7951
7.4979
7.4946
7.4761
7.4612
7.4569
7.4491
7.4259
7.4224
7.4190
7.4094
7.4044
7.3978
7.3889
7.3858
7.3823
7.3672
7.3603
7.3560
7.3476
7.3433
7.2595 cdcl3
5.7818
5.7726
5.7608
5.7517
4.7596
4.7386
4.7284
4.7076
4.3295
4.3202
4.2984
4.2890
3.2467
Ph
N
N
N
OH
5a
1.99
0.94
4.62
0.88
2.31
1.00
1.04
1.05
1.15
f1 (ppm)

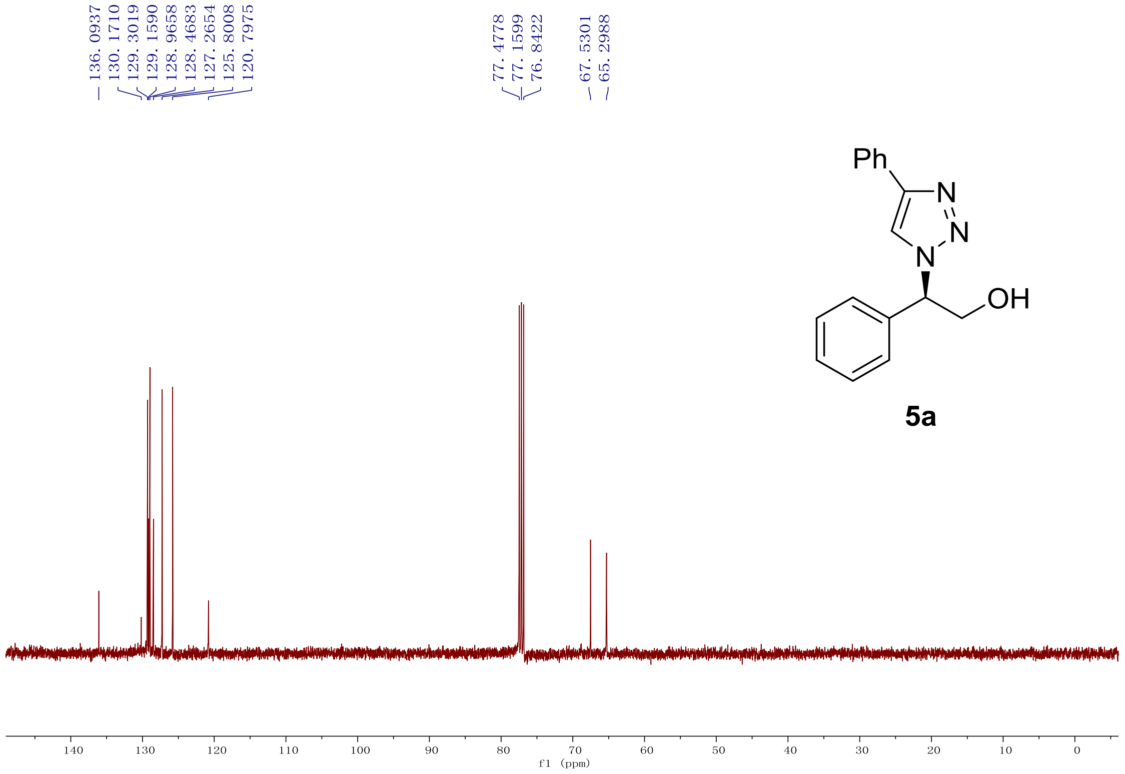
136.0937
130.1710
129.3019
129.1590
128.9658
128.4683
127.2654
125.8008
120.7975
77.4778
77.1599
76.8422
67.5301
65.2988
Ph
N
N
N
OH
5a
f1 (ppm)

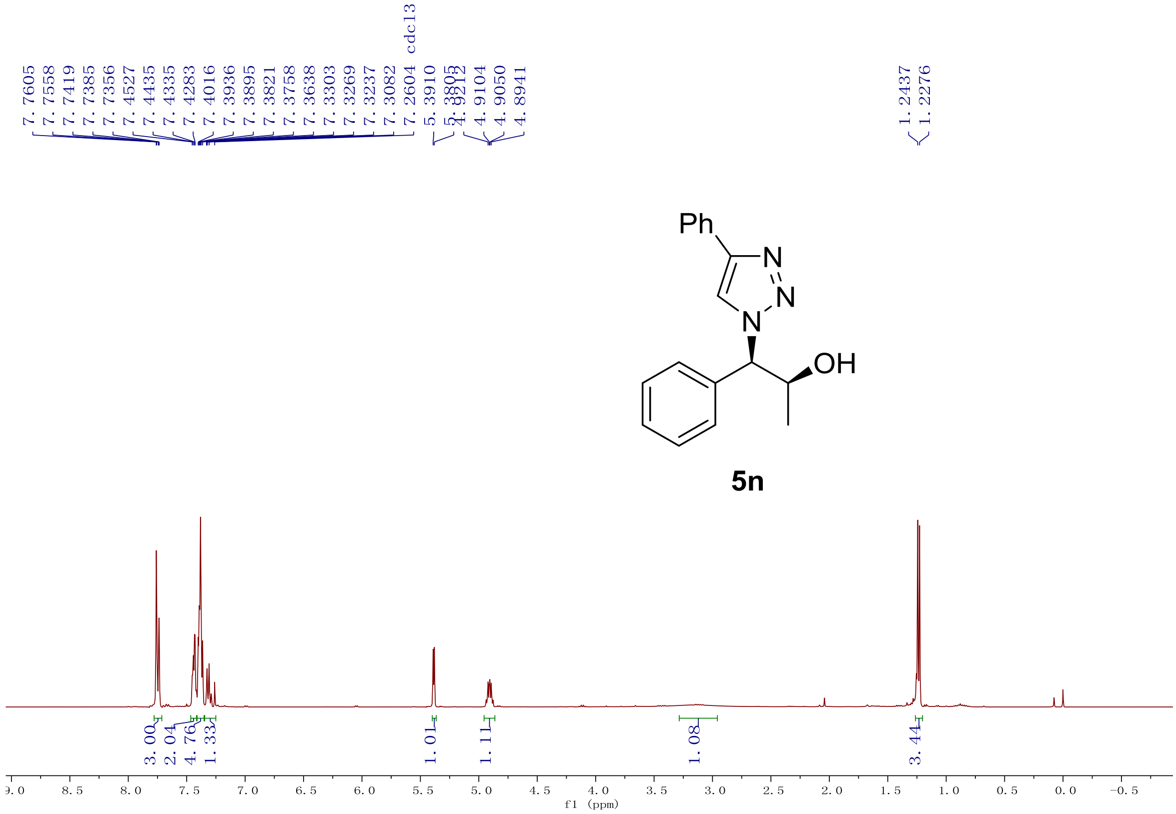
7.7605
7.7558
7.7419
7.7385
7.7356
7.4527
7.4435
7.4335
7.4283
7.4016
7.3936
7.3895
7.3821
7.3758
7.3638
7.3303
7.3269
7.3237
7.3082
7.2604 cdcl3
5.3910
5.3805
4.9212
4.9104
4.9050
4.8941
1.2437
1.2276
Ph
N
N
N
OH
3.00
2.04
4.76
1.33
1.01
1.11
1.08
3.44
f1 (ppm)

5n

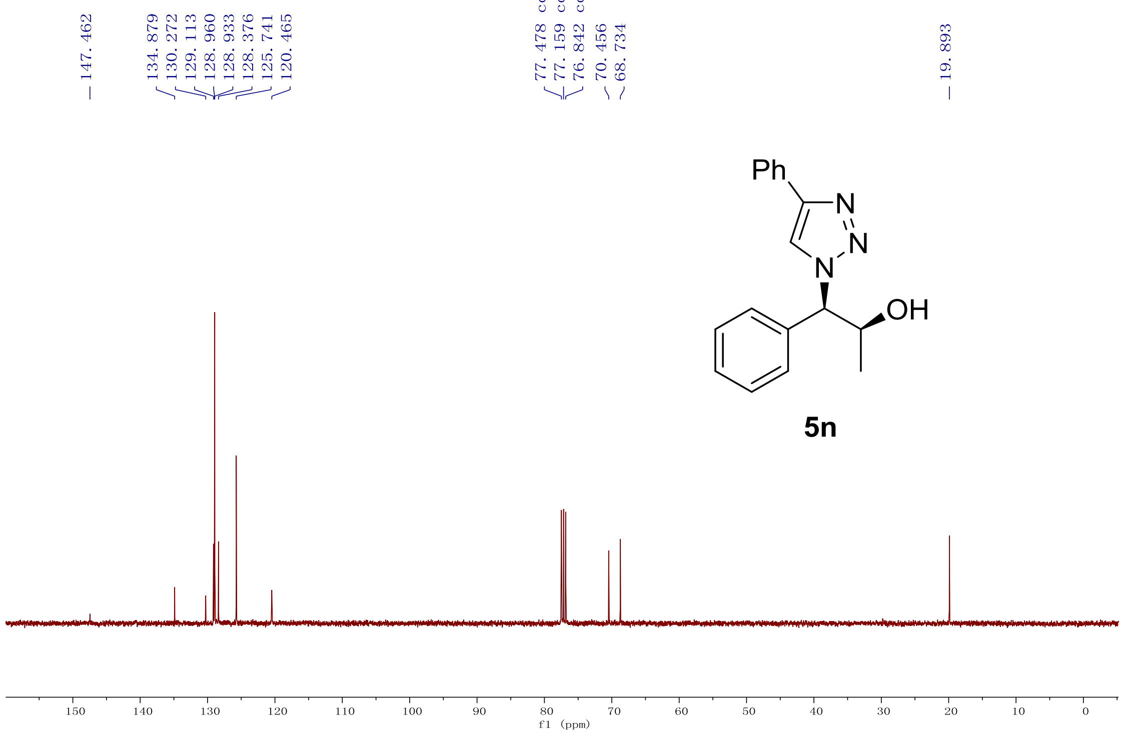
147.462
134.879
130.272
129.113
128.960
128.933
128.376
125.741
120.465
77.478 cdcl3
77.159 cdcl3
76.842 cdcl3
70.456
68.734
19.893
Ph
N
N
N
OH
f1 (ppm)

5n

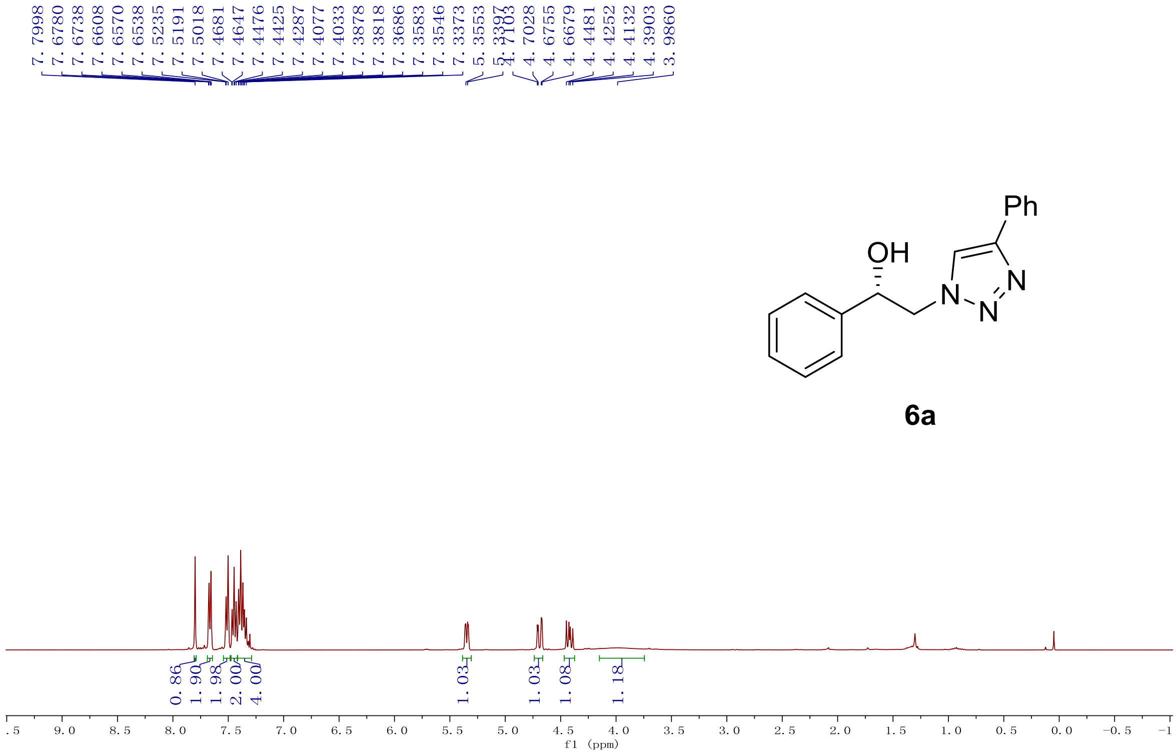
7.7998
7.6780
7.6738
7.6608
7.6570
7.6538
7.5235
7.5191
7.5018
7.4681
7.4647
7.4476
7.4425
7.4287
7.4077
7.4033
7.3878
7.3818
7.3686
7.3583
7.3546
7.3373
5.3553
5.3397
4.7103
4.7028
4.6755
4.6679
4.4481
4.4252
4.4132
4.3903
3.9860
Ph
OH
N
N
N
6a
0.86
1.90
1.98
2.00
4.00
1.03
1.03
1.08
1.18
f1 (ppm)

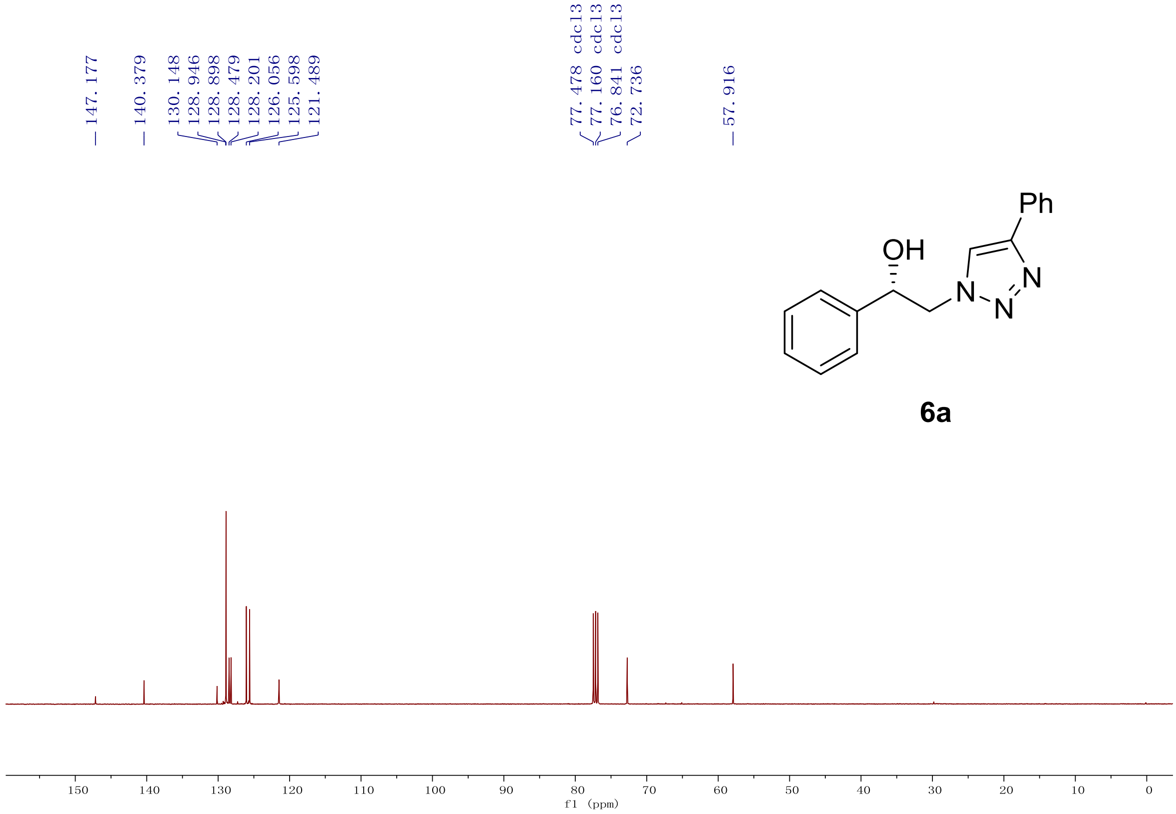
147.177
140.379
130.148
128.946
128.898
128.479
128.201
126.056
125.598
121.489
77.478 cdcl3
77.160 cdcl3
76.841 cdcl3
72.736
57.916
Ph
OH
N
N
N
6a
f1 (ppm)

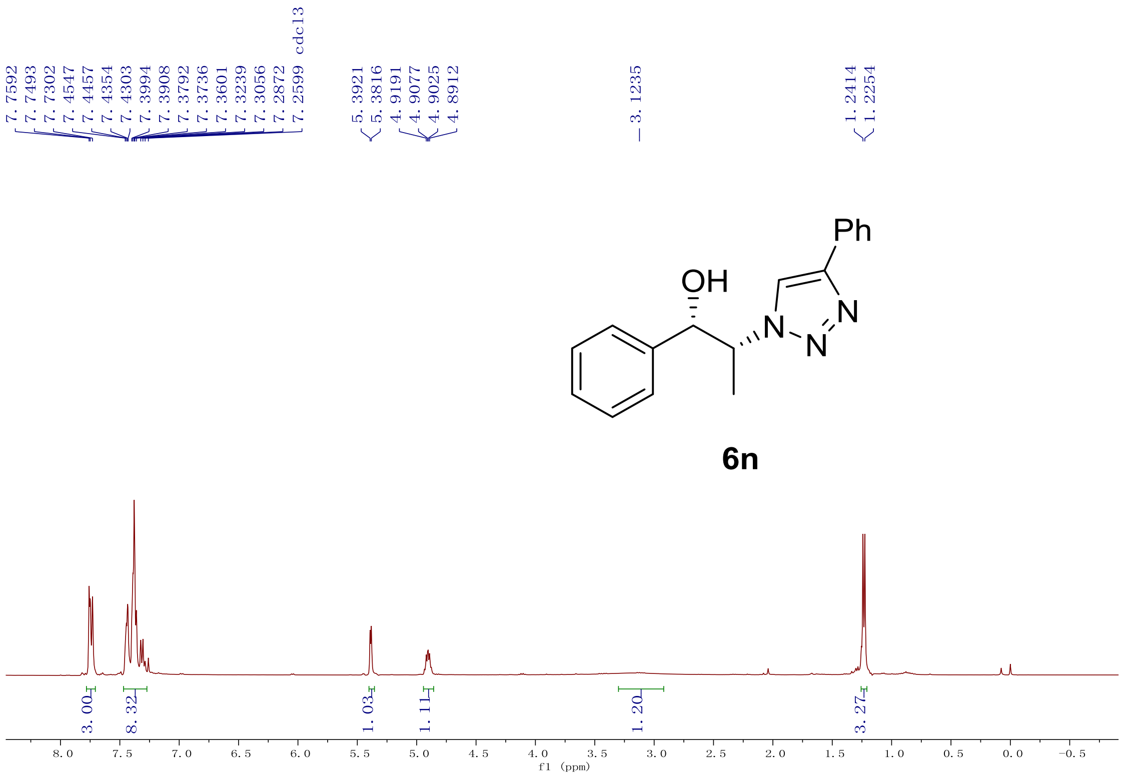
7.7592
7.7493
7.7302
7.4547
7.4457
7.4354
7.4303
7.3994
7.3908
7.3792
7.3736
7.3601
7.3239
7.3056
7.2872
7.2599 cdcl3
5.3921
5.3816
4.9191
4.9077
4.9025
4.8912
3.1235
1.2414
1.2254
OH
Ph
N
3.00
8.32
1.03
1.11
1.20
3.27
f1 (ppm)

6n

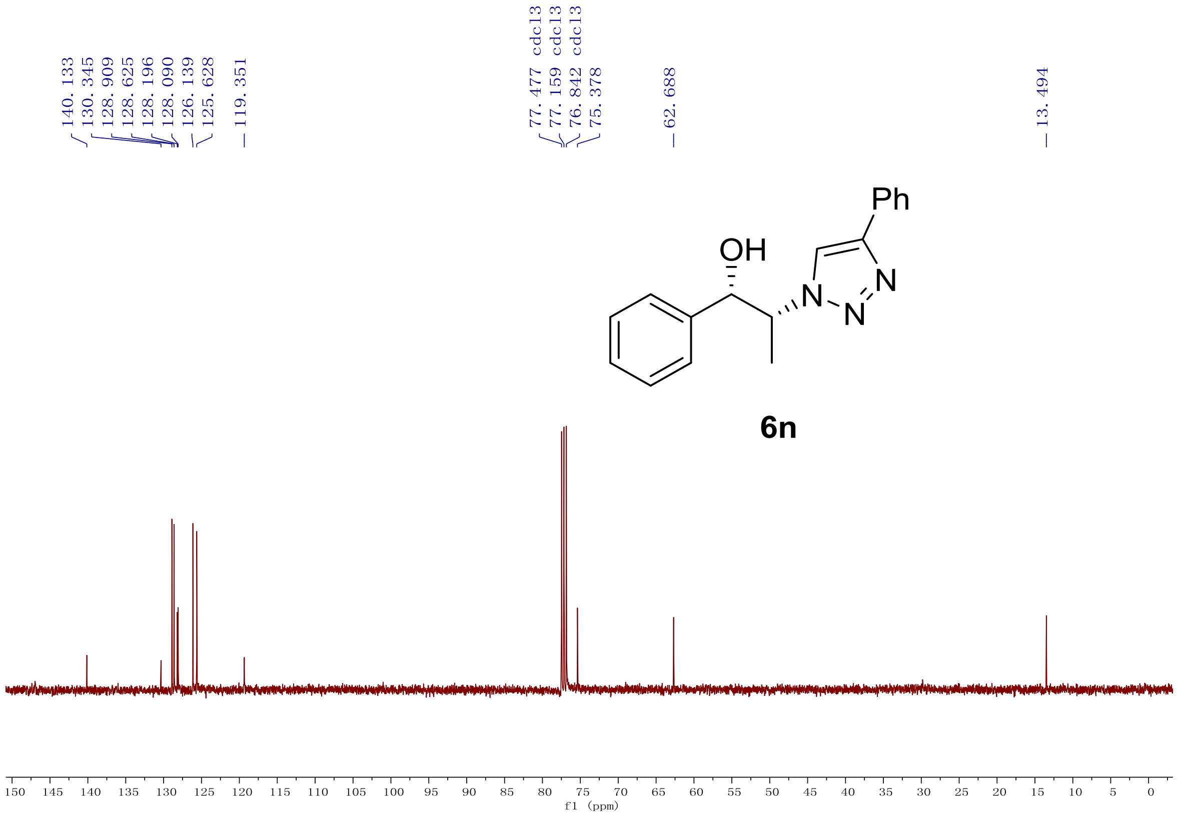
140.133
130.345
128.909
128.625
128.196
128.090
126.139
125.628
119.351
77.477 cdcl3
77.159 cdcl3
76.842 cdcl3
75.378
62.688
13.494
OH
Ph
N
f1 (ppm)

6n

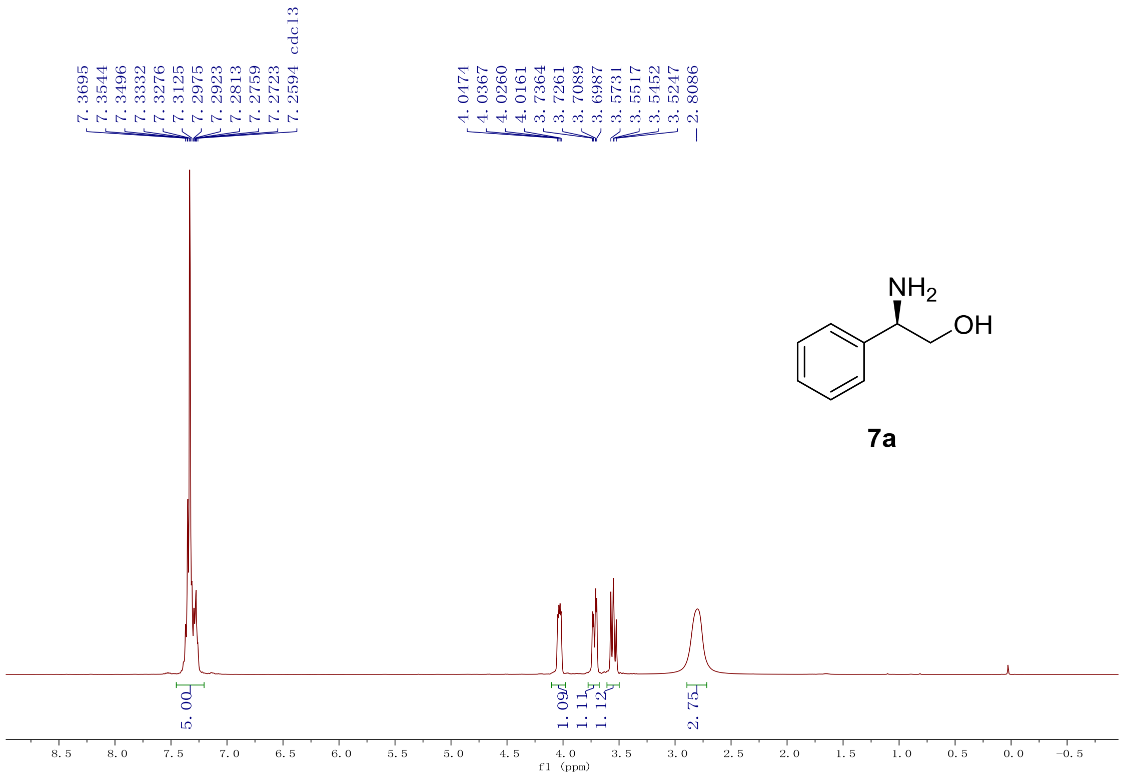

7a

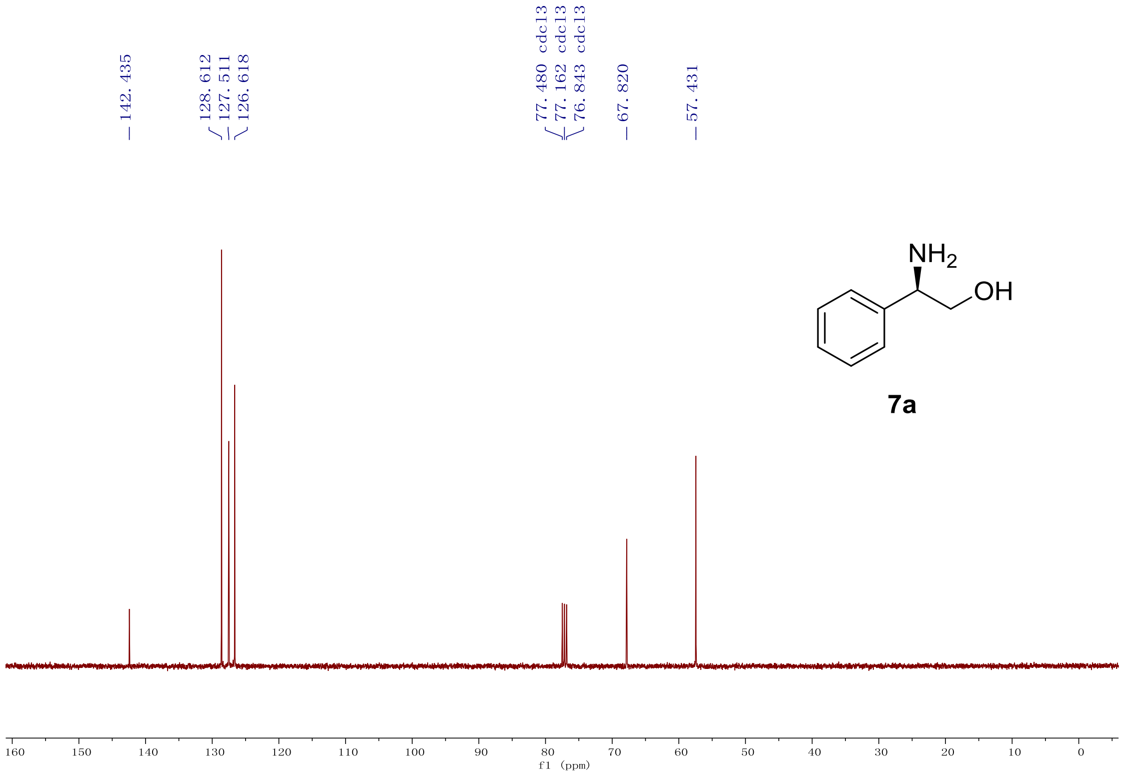

7a

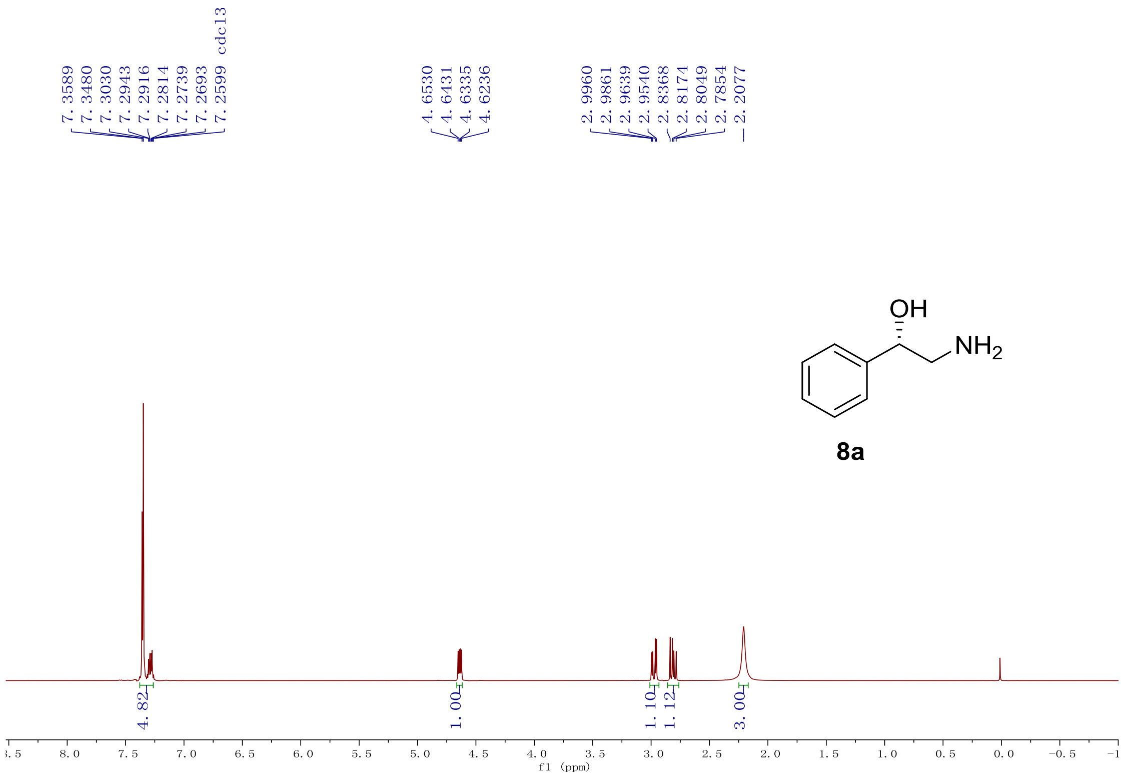
7.3589
7.3480
7.3030
7.2943
7.2916
7.2814
7.2739
7.2693
7.2599 cdcl3
4.6530
4.6431
4.6335
4.6236
2.9960
2.9861
2.9639
2.9540
2.8368
2.8174
2.8049
2.7854
2.2077
OH
NH2
8a
4.82
1.00
1.10
1.12
3.00
f1 (ppm)

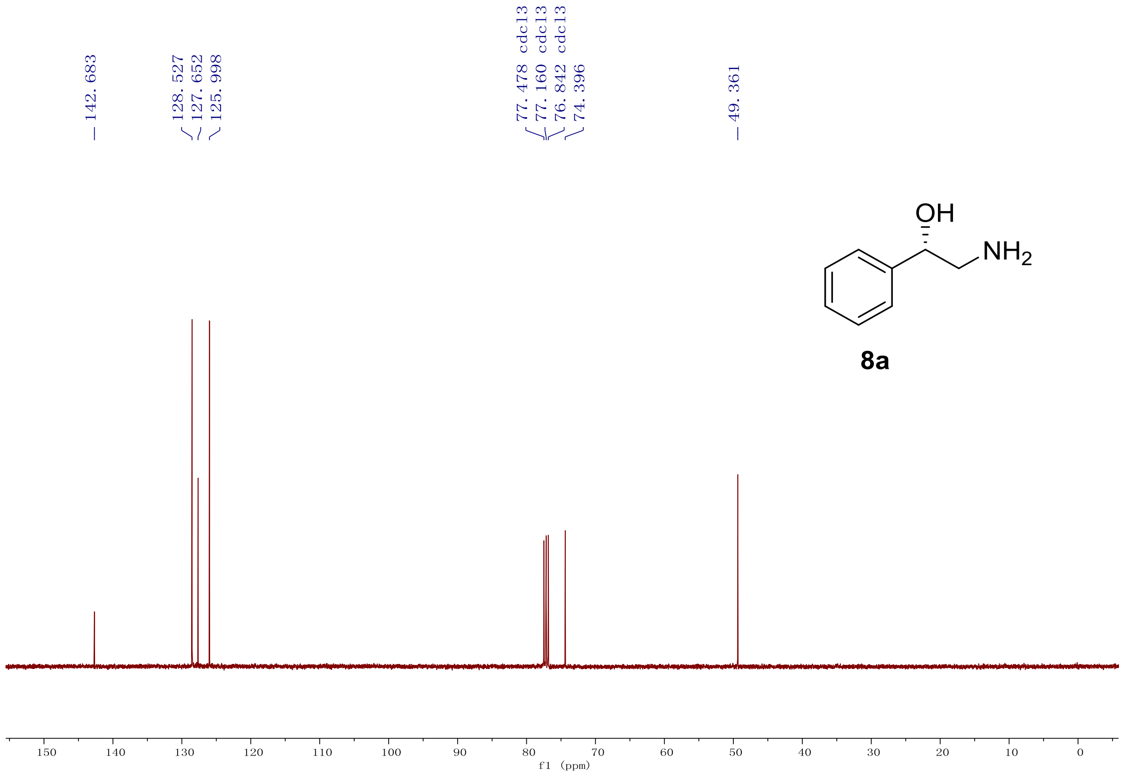
142.683
128.527
127.652
125.998
77.478 cdcl3
77.160 cdcl3
76.842 cdcl3
74.396
49.361
OH
NH2
8a
f1 (ppm)
